# Synthesis of Dithioester Derivatives by Base-Mediated Fragmentation of 1,3-Dithiolanes

**DOI:** 10.1021/acs.orglett.5c00666

**Published:** 2025-04-14

**Authors:** Hasan Pelit, Mehmet Aytug Sinmaz, Oyku Acelya Ildem, Zeynep Mert, Yigit Efe Turhan, Elif Aydin, Senem Dila Yilmaz, Andrea Mentese, Baris Yucel

**Affiliations:** Istanbul Technical University, Science Faculty, Department of Chemistry, Istanbul 34469, Türkiye

## Abstract

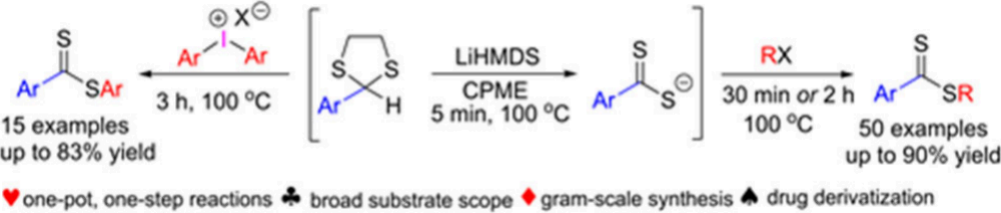

Dithioesters are important agents for chain transfer in polymer chemistry and precursors in the synthesis of heterocycles. Straightforward approaches to their synthesis are therefore in demand. Outlined herein is a method to access such compounds in one-pot. 2-Aryl-1,3-dithiolanes undergo ring fragmentation with LiHMDS in CPME to generate aryl-dithiocarboxylates in 5 min at 100 °C. These anions are subsequently captured in the second step by addition of various alkyl halides and diaryliodonium salts to furnish a large library of dithioesters in good yields. The method can be also employed in a one-pot, one-step manner for alkyl bromides and allows the synthesis of dithioesters in gram scale.

Dithioester derivatives particularly receive attention as RAFT agents in polymer chemistry.^1^ They are also used as precursors for the synthesis of various heterocyles,^2^ including penam-type antibiotics and pyrazolopyrimidines, potent tyrosine kinase inhibitors.^3^ Thus, a number of approaches have been described for the synthesis of dithioesters, each with its own set of advantages and disadvantages.^1,4^ The most common method for their synthesis is the alkylation of carbodithioate salts generated by the reaction of Grignard reagents with carbon disulfide (Scheme 1a).^5^ Limitations in the preparation of air-sensitive Grignard reagents and the high toxicity of volatile carbon disulfide are the main concerns of this method. Another widely applied method for dithioester synthesis is the use of thionating reagents such as P_4_S_10_, Lawesson’s reagent and other organophosphorus sulfides with thioesters and carboxylic acids (Scheme 1b).^6^ However, this method requires either high temperatures or long reaction times. Moreover, commercially available Lawessons’s reagent is unstable at high temperatures and P_4_S_10_ needs to be purified prior to use to ensure reliable results.^7^

**Scheme 1:**
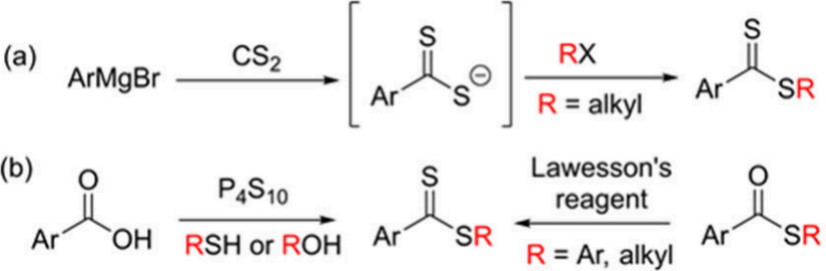
Common Synthesis Methods for Dithioester Derivatives

It is well-known that 1,3-dithiane derivatives are easily metalated with an appropriate base, such as *n-*BuLi, at the C(2) position and thus hold an indispensable place as masked acyl anion equivalents in organic synthesis.^8^ In contrast to 1,3-dithianes, their 5-membered analogs, 1,3-dithiolanes, readily undergo ring fragmentation when they are deprotonated at the same position, resulting in dithiocarboxylate anion and ethylene gas (Scheme 2a).^9^ Moreover, depending on the reaction conditions, (nature of the solvent, base, and substituents on the ring), 1,3-dithiolanes can also undergo deprotonation at C(4) to furnish thioaldehydes and vinyl thiolates (Scheme 2a).^9^ To fully harness the synthetic potential of base-induced fragmentation of 1,3-dithiolane rings, the reaction conditions must be carefully tuned.

**Scheme 2:**
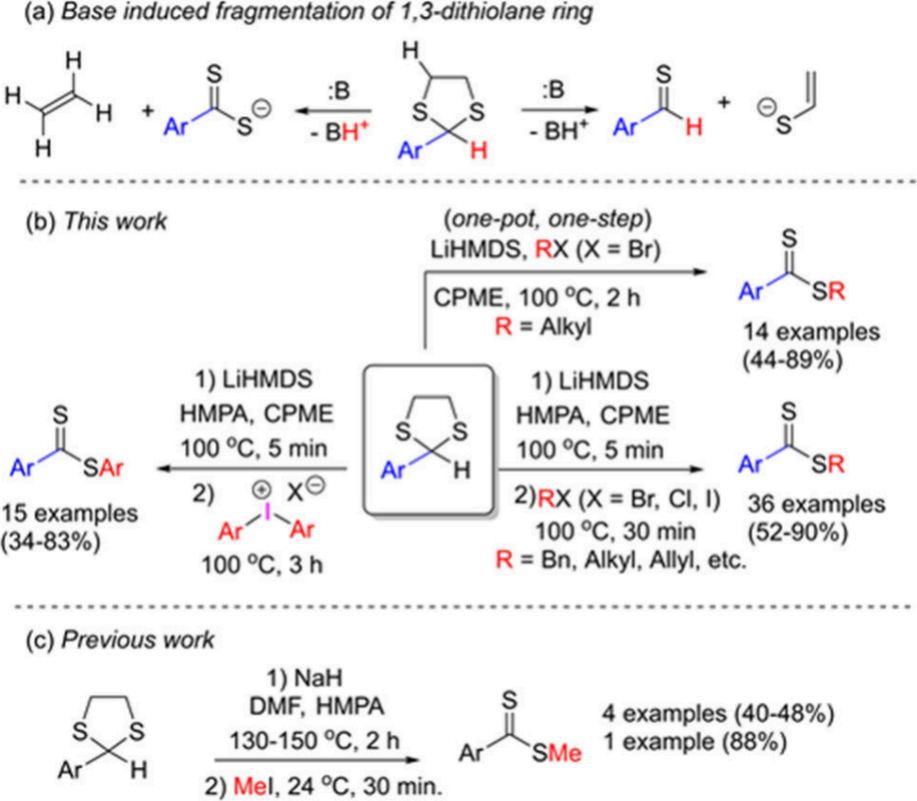
Base Induced Fragmentation of 1,3-Dithiolane Ring and Synthesis of Dithioesters

We viewed this fragmentation as having the potential for a one-pot synthesis of dithioesters. Under the optimized conditions, we found that it is possible to selectively deprotonate the dithiolane ring at the C(2) position by using LiHMDS in CPME (cyclopentyl methyl ether) at 100 °C in only 5 min (Scheme 2b). The corresponding dithiocarboxylates were subsequently captured by various alkyl halides and diaryliodonium salts to provide an array of dithioesters.

The same strategy was applied for the synthesis of methyl dithioesters by Cava et. al in 1979 and stayed untouched so far (Scheme 2c).^10^ They reported 5 examples of the synthesis of methyl dithioesters with an average yield of 52%. They used NaH as a base in the mixture of DMF and HMPA at temperatures between 130 and 150 °C in 2 h to provide the fragmentation of 2-aryl-1,3-dithiolanes. In the second step, intermediate dithiocarboxylates were methylated by addition of methyl iodide at room temperature.

With the goal of developing a general synthesis of dithioesters, we initiated our study by screening the one-pot reactions of **1a** with five different bases (KO*t*Bu, NaH, NaHMDS, KHMDS, and LiHMDS) in CPME at different temperatures with ethyl bromide (entries 1–8, Table 1). Among them, 77% yield was achieved by 1.0 equiv of LiHMDS in 2 h at 100 °C (entry 5). We next tested the reaction of **1a** with LiHMDS in various solvents, finding that CPME was the best choice (entries 9–12). The equivalents of base (entries 13–15) appeared to be the key factor in the reaction’s success, rather than the reaction concentration (entries 15–16) or the presence of HMPA as additive (entry 17). Based on these findings, we established that the optimal reaction conditions were those described in entry 16.

**Table 1:**
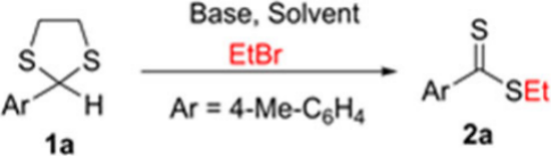
Optimization Table

Entry	Base (equiv)	Solvent (mL)	Temp./Time (°C/h)	Yield **2a** (%)
1	KO*t*Bu (2.0)	CPME (1.0)	100 °C/2 h	-
2^a^	NaH (2.0)	CPME (1.0)	100 °C/2 h	-
3	NaHMDS (1.0)	CPME (2.0)	100 °C/2 h	59%
4	KHMDS (1.0)	CPME (2.0)	100 °C/2 h	54%
5	LiHMDS (1.0)	CPME (2.0)	100 °C/2 h	77%
6	LiHMDS (1.0)	CPME (2.0)	60 °C/2 h	trace
7	LiHMDS (1.0)	CPME (2.0)	80 °C/2 h	trace
8	LiHMDS (1.0)	CPME (1.0)	100 °C/0.5 h	29%
9	LiHMDS (1.0)	DME (2.0)	100 °C/2 h	51%
10	LiHMDS (1.0)	Dioxane (2.0)	100 °C/2 h	47%
11	LiHMDS (1.0)	Toluene (2.0)	100 °C/2 h	39%
12	LiHMDS (1.0)	THF (2.0)	100 °C/2 h	53%
13	LiHMDS (2.0)	CPME (1.0)	100 °C/2 h	51%
14	LiHMDS (1.2)	CPME (2.0)	100 °C/2 h	60%
15	LiHMDS (1.1)	CPME (2.0)	100 °C/2 h	80%
**16**	**LiHMDS (1.1)**	**CPME (1.0)**	**100 ^o^C**/**2 h**	**80%**
17^a^	LiHMDS (1.1)	CPME (1.0)	100 °C/2 h	78%

a HMPA (0.5 equiv) was used as additive

Under the optimized conditions, we next performed the reaction of variously substituted 2-aryl-1,3-dithiolanes with ethyl bromide to explore the scope and limitations of this one-pot, one-step approach to dithioester derivatives (Scheme 3). Except for **2h** and **2l** having 4-(trifluoromethyl)phenyl and furyl groups, respectively, all dithioester derivatives (**2a**–**g, 2i–k**) with diverse electronic and steric effects could be achieved in good yields (60–89%). On the other hand, the reaction of 2-cyclohexyl-1,3-ditholane (**1m**) did not produce the corresponding dithioester **2m**. The reaction gave a ketene dithioacetal derivative, *α,α*-bis(thioethyl)methylenecyclohexane **2m′**, in 20% yield together with unreacted starting dithiolane (**1m**) (see Scheme S2). The reaction of dithiolane **1n** without an alpha-hydrogen could produce dialkyl-substited dithioester **2n** in 13% yield, yet the prolonged reaction time (18 h) was necessary. When ethyl bromide was replaced with longer alkyl bromides, the reactions of **1a** were also successful, affording dithioesters **3a** and **4a** in 80 and 85% yields, respectively. Interestingly, under the optimized conditions, the reaction of **1a** with benzyl bromides gave the corresponding dithioesters (**5a**–**7a**) in lower yields (31–35%). We envisaged that performing the reaction in two steps might improve the yields. Thus, in the first step, we attempted to generate the dithiocarboxylate anion by the reaction of **1a** with 1.2 equiv of LiHMDS in CPME at 100 °C. After 3 min, addition of benzyl bromide, such as 4-chlorobenzyl bromide in CPME, to the resulting dark red solution at the 100 °C boosted the yield of **6a** to 68%. We found it necessary to use 0.5 equiv of HMPA and prolong the reaction time to 5 min in the first step to achieve **6a** in 80% yield. We realized that in the second step, adding 1.1 equiv of 4-chlorobenzyl bromide and stirring the mixture for 30 min at 100 °C was sufficient to complete the reaction.

**Scheme 3:**
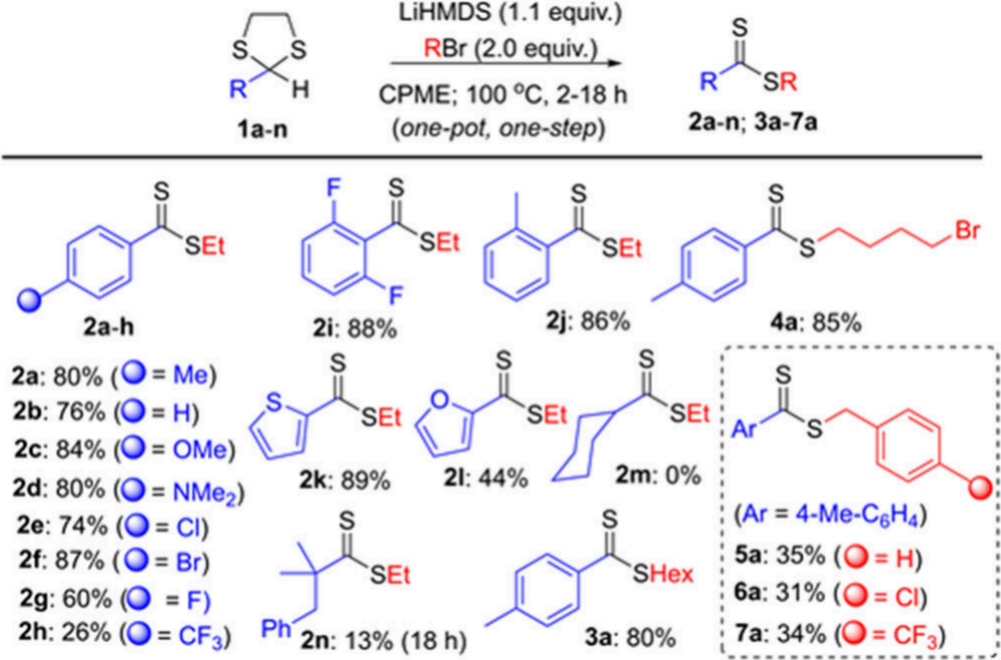
One-Pot, One-Step Synthesis of Dithioesters

We next investigated the scope of the one-pot, two-step protocol with variously substituted benzylic halides. As depicted in Scheme 4, irrespective of electronic properties and the position of the substituents on the benzyl group, dithioester derivatives **5a**–**19a**, **21a** were obtained in 65–84% yields. Heteroaryl benzylic halides reacted well with **1a** under the optimized conditions, affording dithioesters **20a**, **22a**–**25a** in 66–82% yields. Moreover, we screened substrates other than benzylic halides, such as methyl-2-bromopropionate, allyl bromide, and bromoacetonitrile to widen the scope of this method. These alkylating reagents all gave the corresponding dithioesters **16a**, **26a**–**27a** in good yields (66–79%). Under the optimal reaction conditions, this method was also applied to the derivatization of medications gemfibrozil and oxaprozin, providing the corresponding dithioesters **28a**–**29a** in 72 and 52% yields, respectively.

**Scheme 4:**
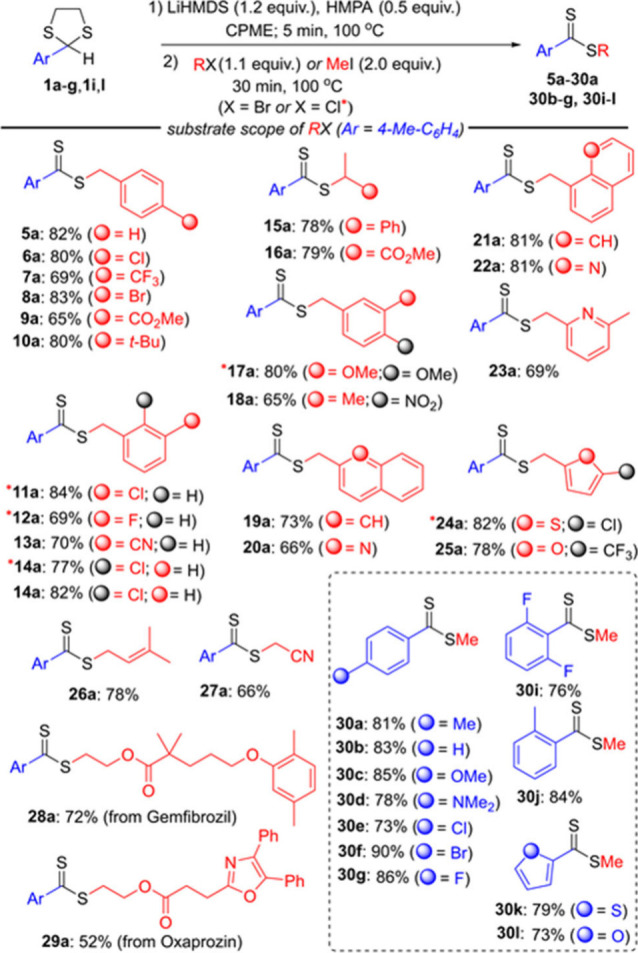
Substrate Scope for Benzylic Halides and MeI

We next explored the scope of 2-aryl-1,3-dithiolanes (**1a**–**g**, **1i**–**l**) by using methyl iodide in the second step. Overall, 1,3-dithiolanes bearing an aryl group with electron-donating, electron-withdrawing substituents and 2-hetaryl-1,3-dithiolanes furnished the methyl dithioesters **30a**–**g** and **30i**–**l** in 73–90% yields within 35 min of reaction time. On the other hand, the reaction of methyl iodide with **1a** gave only 33% yield in the one-pot, one-step procedure described above. In addition, this procedure was tested with Michael acceptors such as acrylonitrile and methyl acrylate to broaden the scope of the method even further, but these reactions produced unsatisfactory results under the optimized conditions.

To demonstrate the synthetic utility of this method, we performed 10.0 mmol scale reactions of **1a** without using a glovebox and employed commercially available LiHMDS solution in THF (Scheme 5). One-pot, one-step reaction of **1a** with ethyl bromide proceeded well in the mixture of CPME and THF, affording dithioester **2a** in 84% yield (1.65 g). We also conducted the reaction of **1a** with benzyl bromide in a one-pot, two-step fashion. In this case, the reaction yielded dithioester **5a** in 68% yield (1.76 g), which was lower than the 82% yield obtained with 0.5 mmol of **1a** in CPME only under optimized conditions. When the same procedure was carried out with methyl iodide, the product **30a** was isolated in an 83% yield (1.51 g).

**Scheme 5:**
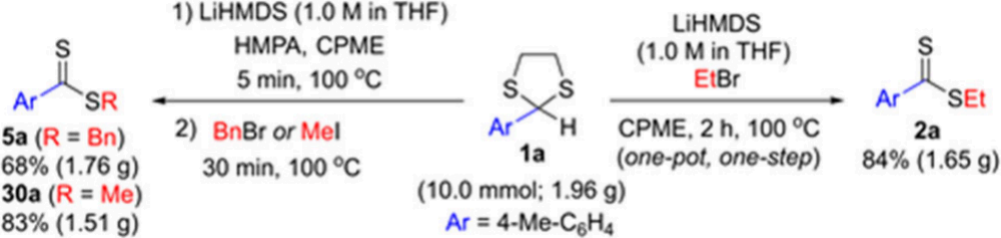
Gram Scale Synthesis of Dithioesters

Finally, considering successful results on arylation of sulfur-centered nucleophiles with diaryliodonium salts,^11^ we attempted to arylate dithiocarboxylate anions to furnish diaryl dithioesters under transition metal-free conditions (Scheme 6). We first performed one-pot, one-step reaction of **1a** with diphenyliodonium triflate in the presence of LiHMDS in CPME at 100 °C for 2h, which resulted in the formation of **31a** in only 20% yield. We then decided to conduct the reaction in two steps, as described for benzyl halides. However, we noticed that the reaction time for the second step must be prolonged to 3 h for the best yields. Since the counteranion influences the reactivity of diaryliodonium salts,^11a,11c^ the reaction of **1a** with diphenyliodonium salts possessing triflate, hexafluorophosphate, chloride, and iodide counteranions were tested. Among them, the best result was achieved by diphenyliodonium hexafluorophosphate, producing dithioester **31a** in 73% yield. To investigate the scope of the reaction, we next coupled 2-aryl-1,3-dithiolanes and commercially available diaryliodonium salts bearing aryls with different electronic and steric properties. Overall, diaryl dithioesters (**31a**–**36a**, **31c**–**d**, **g**, and **31i**–**k**) were produced in moderate to good yields, regardless of the electronic properties of the aryl groups. The reactions of dithiolanes (**1a** and **1e**–**f**) with sterically encumbered di(mesityl)iodonium salt were also successful, producing the corresponding products **37a** in 64% yield and **37e**–**f** in 76 and 83% yields, respectively. On the other hand, dithioester **31j** was isolated in 34% yield when 2-(*o*-tolyl)-1,3-dithiolane reacted with diphenyliodonium triflate. This implies that the steric properties of the aryl group on dithiolane ring are more influential to the reaction’s success.

**Scheme 6:**
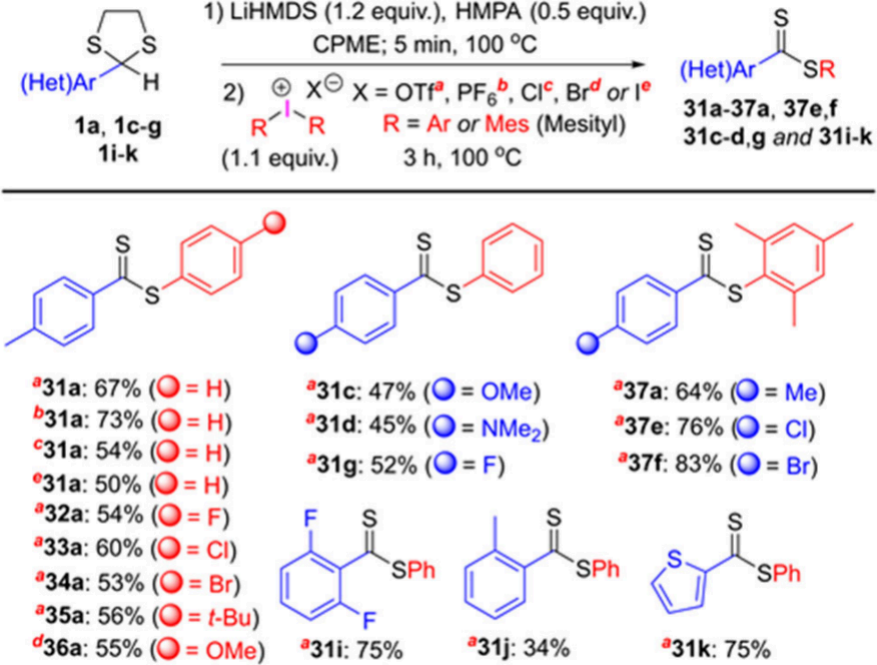
Substrate Scope for Diaryliodonium Salts

In conclusion, we have thoroughly studied previously underexplored base mediated fragmentation of 2-aryl-1,3-dithiolanes and established it as an effective method for the synthesis of a wide range of dithioesters in short reaction times and with generally good yields. One of the key benefits of this method is that the starting compounds 2-aryl-1,3-dithiolanes can be easily prepared, even in a 1 min at room temperature,^12^ using well well-known procedures. Moreover, the presented method is operationally simple and enables the synthesis of dithioesters on gram scales.

## Data Availability

The Data underlying this study are available in the published article and its Supporting Information.

## Supporting Information Available

All experimental procedures and NMR, HRMS spectra data, and copies of ^1^H NMR and ^13^C NMR spectra (PDF)
FAIR data, including the primary NMR FID files, for compounds **2a**–**n**, **3a**–**30a**, **30b**–**g**, **30i**–**l**, **31a**–**37a**, **37e**–**f**, **31c**–**d**, **g, 31i**–**k**, **2m′** (ZIP, ZIP, ZIP)
