# Supplementary material for: Synthesis of Dithioester Derivatives by Base-Mediated Fragmentation of 1,3-Dithiolanes
Source: Org Lett. 2025 Apr 14;27(16):4135–9. doi: 10.1021/acs.orglett.5c00666 (PMC12038844; doi:10.1021/acs.orglett.5c00666)

SUPPORTING INFORMATION

Synthesis of Dithioester Derivatives by Base-mediated Fragmentation of 1,3-Dithiolanes

Hasan Pelit, Mehmet Aytug Sinmaz, Oyku Acelya Ildem, Zeynep Mert, Yigit Efe Turhan, Elif Aydin, Senem Dila Yilmaz, Andrea Mentese, Baris Yucel*

Istanbul Technical University, Science Faculty, Department of Chemistry, Maslak 34469, Istanbul, Türkiye.

TABLE OF CONTENTS

1. General Methods ……………………………………………………………….………….…………….S2

2. General procedure A (for one-pot, one-step reactions of 2-aryl-1,3-dithiolanes with alkyl bromides)….........S3

2.1. Synthesis of dithioesters **2a-n**; **3a-7a**…………………………………………………………...…..S3

2.2. Synthesis of *α,α*-bis(thioethyl)methylenecyclohexane **2m'**……………...…………………….……….S8

3. General procedure B (for one-pot, two-step reactions of 2-aryl-1,3-dithiolanes with alkyl halides)….……...S8

3.1. Synthesis of dithioesters **5a-30a**, **30b-g**, **30i-l**……………………………………………..………...S9

4. General procedure C (for one-pot, two-step reactions of 2-aryl-1,3-dithiolanes with diaryliodonium salts)...S24

4.1. Synthesis of dithioesters **31a-37a, 37e-f, 31c-d, g** and **31i-k** ……………………………………………. S25

5. Gram scale synthesis of ethyl 4-methylbenzodithioate (**2a**)…………………………………………………S31

6. Gram scale synthesis of benzyl 4-methylbenzodithioate (**5a**) and methyl 4-methylbenzodithioate (**31a**)...... S32

7. Synthesis of 2-bromoethyl 3-(4,5-diphenyloxazol-2-yl)propanoate………………………………………S33

8. References……………..…………………………………………………….…………………..S34

9. NMR Spectra of compounds……………………..……………………………….…………………..S35

1. General Methods. All reagents were used as purchased from commercial suppliers without further purification unless otherwise indicated. Air- and moisture-sensitive solutions were handled under nitrogen and transferred via syringe. Tetrahydrofuran (THF) was freshly distilled from sodium/benzophenone ketyl. Anhydrous CPME, DME, Dioxane and Toluene were purchased from Merck and stored in glovebox. All solvents used were stored over activated molecular sieves (3 or 4 Å). Molecular sieves were pre-dried at 300 ºC for 24 h immediately before use. Solvents for column chromatography, ethyl acetate and hexanes were distilled in a rotary evaporator. TLC was performed with Merck TLC Silicagel60 F_{254} plates and detection was under UV light at 254 nm. Chromatographic separations were performed with Merck Silica 60 (200–400 or 70–230 mesh). NMR spectra were recorded with a Varian Inova 500 (500 MHz for ^{1}H and 125 MHz for ^{13}C NMR) instruments. Chemical shifts δ were given in ppm relative to residual peaks of deuterated solvents and coupling constants, *J*, were given in Hertz. The following abbreviations are used to describe spin multiplicities in ^{1}H NMR spectra: s = singlet; bs = broad singlet; d = doublet; t = triplet; q = quartet; dd = doublet of doublets; m = multiplets. Multiplicities in ^{13}C NMR spectra were determined by DEPT (Distortionless Enhancement by Polarization Transfer) or APT (Attached Proton Test) measurements. High resolution mass spectra (HRMS) were obtained on Waters Synapt Q-TOF-MS and Thermo Scientific™ Q Exactive Hybrid Quadrupole-Orbitrap MS spectrometers.

2. General procedure A (for one-pot, one-step reactions of 2-aryl-1,3-dithiolanes with alkyl bromides)

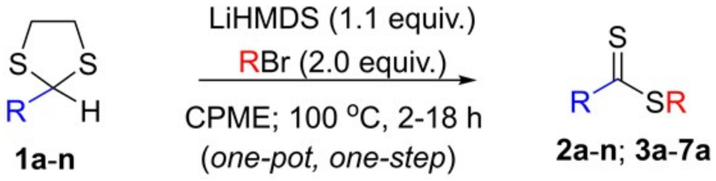

Scheme S1.

An oven-dried 15 mL screw-cap reaction vial equipped with a stirring bar was charged with 2-aryl-1,3-dithiolane derivative (0.5 mmol, 1.0 equiv.) and the vial was brought into a glovebox. The reaction vial was charged with LiHMDS (0.55 mmol, 92.0 mg, 1.1 equiv.), anhydrous CPME (1.0 mL) and then alkyl bromide (1.0 mmol, 2.0 equiv.) by a micropipette. The vial was tightly closed, wrapped with a strip of Parafilm, and taken out of the glovebox. After having stirred the reaction mixture for 2 hours at 100 °C in a pre-heated oil bath, the vial was cooled to room temperature. The reaction mixture was taken into a 50 mL flask and the solvent was removed in a rotatory evaporator. The remaining residue was dissolved in CH_2Cl_2 and mixed with silica gel (about 0.5 g). After evaporating CH_2Cl_2, the remaining silica gel was directly loaded onto a column and purified by flash chromatography on silica gel using hexanes/ethyl acetate mixture as eluent to yield the product.

2.1. Synthesis of dithioesters 2a-n; 3a-7a

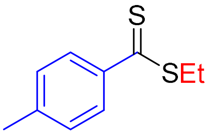

Ethyl 4-methylbenzodithioate (**2a**)[1]: Following General Procedure A, 2-(*p* -tolyl)-1,3-dithiolane (**1a**, 0.5 mmol, 98 mg), LiHMDS (0.55 mmol, 92 mg) and ethyl bromide (1.0 mmol, 76 μL) were reacted in 1.0 mL CPME at 100 °C for 2 h. Product **2a** was isolated (78.5 mg, 80%) as red oil by flash chromatography on silica gel using hexanes as eluent. ^{1}H NMR (500 MHz, $CDCl_3$): δ 7.94 (d, *J* = 7.9 Hz, 2H), 7.18 (d, *J* = 7.9 Hz, 2H), 3.38 (q, *J* = 7.4 Hz, 2H), 2.38 (s, 3H), 1.42 (t, *J* = 7.4 Hz, 3H) ppm; ^{13}C-APT NMR (125 MHz, $CDCl_3$): δ 227.9, 143.1, 142.6, 128.9, 126.8, 31.3, 21.4, 12.3 ppm; HRMS [TOF MS ES+]: m/z $[M + H]^+$ calcd. for $C_{10}H_{13}S_2$ 197.0459, found 197.0450 (−4.6 ppm).

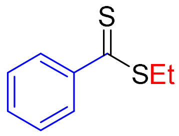

Ethyl benzodithioate (**2b**)[2]: Following General Procedure A, 2-phenyl-1,3-dithiolane (**1b**, 0.5 mmol, 91 mg), LiHMDS (0.55 mmol, 92 mg) and ethyl bromide (1.0 mmol, 76 µL) were reacted in 1.0 mL CPME at 100 ºC for 2 h. Product **2b** was isolated (69 mg, 76%) as red oil by flash chromatography on silica gel using hexanes as eluent. ^{1}H NMR (500 MHz, $CDCl_3$): δ 8.00 (d, *J* = 8.4 Hz, 2H), 7.54–7.51 (m, 1H), 7.40–7.37 (m, 2H), 3.38 (q, *J* = 7.5 Hz, 2H), 1.42 (t, *J* = 7.5 Hz, 3H) ppm; ^{13}C-APT NMR (125 MHz, $CDCl_3$): δ 228.5, 145.1, 132.2, 128.2, 126.7, 31.5, 12.2 ppm; HRMS [FT MS ESI+]: m/z $[M + H]^+$ calcd. for $C_9H_{11}S_2$ 183.0297, found 183.0295 (−1.1 ppm).

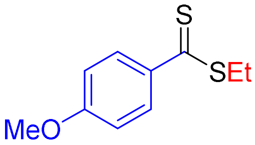

Ethyl 4-methoxybenzodithioate (**2c**)[3]: Following General Procedure A, 2-(4-methoxyphenyl)-1,3-dithiolane (**1c**, 0.5 mmol, 106 mg), LiHMDS (0.55 mmol, 92 mg) and ethyl bromide (1.0 mmol, 76 µL) were reacted in 1.0 mL CPME at 100 ºC for 2 h. Product **2c** was isolated (89 mg, 84%) as red oil by flash chromatography on silica gel using 30:1 hexanes/ethyl acetate as eluent. ^{1}H NMR (500 MHz, $CDCl_3$): δ 8.08 (d, *J* = 8.9 Hz, 2H), 6.87 (d, *J* = 8.9 Hz, 2H), 3.86 (s, 3H), 3.37 (q, *J* = 7.4 Hz, 2H), 1.41 (t, *J* = 7.4 Hz, 3H) ppm; ^{13}C-APT NMR (125 MHz, $CDCl_3$): δ 226.1, 163.4, 138.1, 128.8, 113.3, 55.5, 31.2, 12.4 ppm; HRMS [FT MS ESI+]: m/z $[M + H]^+$ calcd. for $C_{10}H_{13}OS_2$ 213.0402, found 213.0400 (−0.9 ppm).

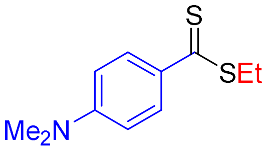

Ethyl 4-(dimethylamino)benzodithioate (**2d**): Following General Procedure A, 4-(1,3-dithiolan-2-yl)-*N,N*-dimethylaniline (**1d**, 0.5 mmol, 113 mg), LiHMDS (0.55 mmol, 92 mg) and ethyl bromide (1.0 mmol, 76 µL) were reacted in 1.0 mL CPME at 100 ºC for 2 h. Product **2d** was isolated (90 mg, 80%) as orange-red solid by flash chromatography on silica gel using 20:1 hexanes/ethyl acetate as eluent. ^{1}H NMR (500 MHz, $CDCl_3$): δ 8.14 (d, *J* = 9.2 Hz, 2H), 6.58 (d, *J* = 9.2 Hz, 2H), 3.38 (q, *J* = 7.5 Hz, 2H), 3.04 (s, 6H), 1.40 (t, *J* = 7.5 Hz, 3H) ppm; ^{13}C-APT NMR (125 MHz, $CDCl_3$): δ 223.4, 153.6, 133.4, 129.2, 110.3, 40.1, 30.4, 12.8 ppm; HRMS [FT MS ESI+]: m/z $[M + H]^+$ calcd. for $C_{11}H_{16}NS_2$ 226.0719, found 226.0716 (−1.3 ppm).

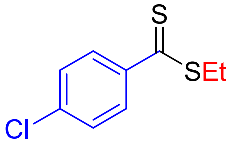

Ethyl 4-chlorobenzodithioate (2e)[4]: Following General Procedure A, 2-(4-chlorophenyl)-1,3-dithiolane (**1e**, 0.5 mmol, 108 mg), LiHMDS (0.55 mmol, 92 mg) and ethyl bromide (1.0 mmol, 76 μL) were reacted in 1.0 mL CPME at 100 ºC for 2 h. Product **2e** was isolated (80 mg, 74%) as red oil by flash chromatography on silica gel using hexanes as eluent. ^{1}H NMR (500 MHz, $CDCl_3$): δ 7.94 (d, J = 8.4 Hz, 2H), 7.34 (d, J = 8.4 Hz, 2H), 3.36 (q, J = 7.5 Hz, 2H), 1.41 (t, J = 7.5 Hz, 3H) ppm; ^{13}C-APT NMR (125 MHz, $CDCl_3$): δ 226.2, 143.1, 138.6, 128.4, 128.0, 31.6, 12.2 ppm; HRMS [FT MS ESI+]: m/z $[M + H]^+$ calcd. for $C_9H_{10}ClS_2$ 216.9907, found 216.9905 (−0.9 ppm).

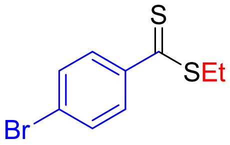

Ethyl 4-bromobenzodithioate (2f)[4]: Following General Procedure A, 2-(4-bromophenyl)-1,3-dithiolane (**1f**, 0.5 mmol, 130.6 mg), LiHMDS (0.55 mmol, 92 mg) and ethyl bromide (1.0 mmol, 76 μL) were reacted in 1.0 mL CPME at 100 ºC for 2 h. Product **2f** was isolated (114 mg, 87%) as red oil by flash chromatography on silica gel using hexanes as eluent. ^{1}H NMR (500 MHz, $CDCl_3$): δ 7.86 (d, J = 8.6 Hz, 2H), 7.50 (d, J = 8.6 Hz, 2H), 3.36 (q, J = 7.5 Hz, 2H), 1.41 (t, J = 7.5 Hz, 3H) ppm; ^{13}C-APT NMR (125 MHz, $CDCl_3$): δ 226.3, 143.6, 131.6, 128.1, 127.2, 31.6, 12.2 ppm; HRMS [FT MS ESI+]: m/z $[M + H]^+$ calcd. for $C_9H_{10}BrS_2$ 260.9402, found 260.9396 (−2.3 ppm).

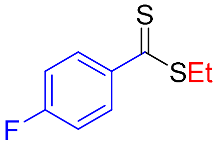

Ethyl 4-fluorobenzodithioate (2g)[5]: Following General Procedure A, 2-(4-fluorophenyl)-1,3-dithiolane (**1g**, 0.5 mmol, 100 mg), LiHMDS (0.55 mmol, 92 mg) and ethyl bromide (1.0 mmol, 76 μL) were reacted in 1.0 mL CPME at 100 ºC for 2 h. Product **2f** was isolated (60 mg, 60%) as red oil by flash chromatography on silica gel using hexanes as eluent. ^{1}H NMR (500 MHz, $CDCl_3$): δ 8.06–8.02 (m, 2H), 7.08–7.03 (m, 2H), 3.37 (q, J = 7.4 Hz, 2H), 1.41 (t, J = 7.4 Hz, 3H) ppm; ^{13}C-APT NMR (125 MHz, $CDCl_3$): δ 226.2, 165.5 (d, J = 245 Hz), 141.3 (d, J = 3.3 Hz), 129.0 (d, J = 8.9 Hz), 115.2 (d, J = 21.9 Hz), 31.6, 12.2 ppm; HRMS [FT MS ESI+]: m/z $[M + H]^+$ calcd. for $C_9H_{10}FS_2$ 201.0202, found 201.0199 (−1.5 ppm).

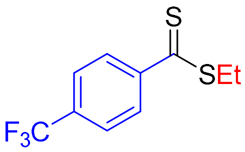

Ethyl 4-(trifluoromethyl)benzodithioate (2h): Following General Procedure A, 2-(4-(trifluoromethyl)phenyl)-1,3-dithiolane (**1h**, 0.5 mmol, 125 mg), LiHMDS (0.55 mmol, 92 mg) and ethyl bromide (1.0 mmol, 76 µL) were reacted in 1.0 mL CPME at 100 ºC for 2 h. Product **2h** was isolated (33 mg, 26%) as red oil by flash chromatography on silica gel using hexanes as eluent. ^{1}H NMR (500 MHz, $CDCl_3$): 8.03 (d, J = 8.1 Hz, 2H), 7.64 (d, J = 8.1 Hz, 2H), 3.39 (q, J = 7.4 Hz, 2H), 1.43 (t, J = 7.4 Hz, 3H) ppm; ^{13}C-APT NMR (125 MHz, $CDCl_3$): δ 226.8, 147.7 (q, J = 1.3 Hz), 133.3 (q, J = 32.6 Hz), 127.0, 125.8 (q, J = 273 Hz), 125.3 (q, J = 3.8 Hz), 31.8, 12.1 ppm; HRMS [FT MS ESI+]: m/z $[M + H]^+$ calcd. for $C_{10}H_{10}F_3S_2$ 251.0171, found 251.0166 (−2.0 ppm).

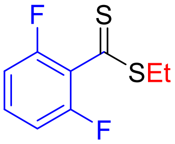

Ethyl 2,6-difluorobenzodithioate (2i): Following General Procedure A, 2-(2,6-difluorophenyl)-1,3-dithiolane (**1i**, 0.5 mmol, 109 mg), LiHMDS (0.55 mmol, 92 mg) and ethyl bromide (1.0 mmol, 76 µL) were reacted in 1.0 mL CPME at 100 ºC for 2 h. Product **2i** was isolated (96 mg, 88%) as red oil by flash chromatography on silica gel using hexanes as eluent. ^{1}H NMR (500 MHz, $CDCl_3$): δ 7.33–7.28 (m, 1H), 6.93 (t, J = 8.0 Hz, 2H), 3.39 (q, J = 7.5 Hz, 2H), 1.42 (t, J = 7.5 Hz, 3H) ppm; ^{13}C-APT NMR (125 MHz, $CDCl_3$): δ 217.1, 157.5 (dd, J = 5.5, 252 Hz), 130.3 (t, J = 10 Hz), 124.2 (t, J = 19.5 Hz), 111.7 (dd, J = 4.8, 20.8 Hz), 31.5, 11.8 ppm; HRMS [FT MS ESI+]: m/z $[M + H]^+$ calcd. for $C_9H_9F_2S_2$ 219.0108, found 219.0106 (−0.9 ppm).

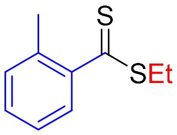

Ethyl 2-methylbenzodithioate (2j): Following General Procedure A, 2-(*o*-tolyl)-1,3-dithiolane (**1j**, 0.5 mmol, 98 mg), LiHMDS (0.55 mmol, 92 mg) and ethyl bromide (1.0 mmol, 76 µL) were reacted in 1.0 mL CPME at 100 ºC for 2 h. Product **2j** was isolated (84 mg, 86%) as orange-red oil by flash chromatography on silica gel using hexanes as eluent. ^{1}H NMR (500 MHz, $CDCl_3$): δ 7.31–7.21 (m, 4 H), 3.37 (q, J = 7.5 Hz, 2H), 2.39 (s, 3H), 1.44 (t, J = 7.5 Hz, 3H) ppm; ^{13}C-APT NMR (125 MHz, $CDCl_3$): δ 233.2, 143.4, 133.1, 130.7, 129.0, 126.3, 125.5, 31.4, 19.3, 12.1 ppm; HRMS [FT MS ESI+]: m/z $[M + H]^+$ calcd. for $C_{10}H_{13}S_2$ 197.0453, found 197.0452 (−0.5 ppm).

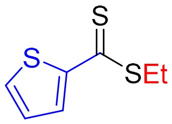

Ethyl thiophene-2-carbodithioate (**2k**): Following General Procedure A, 2-(thiophen-2-yl)-1,3-dithiolane (**1k**, 0.5 mmol, 94 mg), LiHMDS (0.55 mmol, 92 mg) and ethyl bromide (1.0 mmol, 76 μL) were reacted in 1.0 mL CPME at 100 °C for 2 h. Product **2k** was isolated (84 mg, 89%) as red oil by flash chromatography on silica gel using hexanes as eluent. ^{1}H NMR (500 MHz, $CDCl_3$): δ 7.81 (d, J = 3.9 Hz, 1H), 7.61 (d, J = 5.0 Hz, 1H), 7.09 (t, J = 5.0 Hz, 1H), 3.37 (q, J = 7.4 Hz, 2H), 1.40 (t, J = 7.4 Hz, 3H) ppm; ^{13}C-APT NMR (125 MHz, $CDCl_3$): δ 214.1, 151.6, 134.5, 128.3, 126.5, 31.5, 12.7 ppm; HRMS [FT MS ESI+]: m/z $[M + H]^+$ calcd. for $C_7H_9S_3$ 188.9861, found 188.9858 (−1.6 ppm).

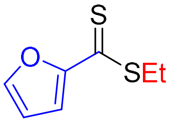

Ethyl furan-2-carbodithioate (**2l**): Following General Procedure A, 2-(furan-2-yl)-1,3-dithiolane (**1l**, 0.5 mmol, 86 mg), LiHMDS (0.55 mmol, 92 mg) and ethyl bromide (1.0 mmol, 76 μL) were reacted in 1.0 mL CPME at 100 °C for 2 h. Product **2l** was isolated (38 mg, 44%) as red oil by flash chromatography on silica gel using hexanes as eluent. ^{1}H NMR (500 MHz, $CDCl_3$): δ 7.62 (dd, J = 1.0, 1.8 Hz, 1H), 7.35 (d, J = 3.6 Hz, 1H), 6.51 (dd, J = 1.8, 3.6 Hz, 1H), 3.35 (q, J = 7.4 Hz, 2H), 1.38 (t, J = 7.4 Hz, 3H) ppm; ^{13}C-APT NMR (125 MHz, $CDCl_3$): δ 207.4, 157.8, 146.3, 115.1, 113.2, 28.8, 11.6 ppm; HRMS [FT MS ESI+]: m/z $[M + H]^+$ calcd. for $C_7H_9OS_2$ 173.0089, found 173.0088 (−0.6 ppm).

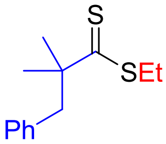

Ethyl 2,2-dimethyl-3-phenylpropanedithioate (**2n**): Following General Procedure A, 2-(2-methyl-1-phenylpropan-2-yl)-1,3-dithiolane (**1n**, 1.0 mmol, 238 mg), LiHMDS (1.10 mmol, 184 mg) and ethyl bromide (2.0 mmol, 152 μL) were reacted in 2.0 mL CPME at 100 °C for 18 h. Product **2n** was isolated (31 mg, 13%) as yellow oil by flash chromatography on silica gel using hexanes as eluent. ^{1}H NMR (500 MHz, $CDCl_3$): δ 7.27–7.21 (m, 3H), 7.15–7.14 (m, 2H), 3.19 (q, J = 7.5 Hz, 2H), 3.12 (s, 2H), 1.47 (s, 6H), 1.32 (t, J = 7.5 Hz, 3H) ppm; ^{13}C-APT NMR (125 MHz, $CDCl_3$): δ 248.9, 137.9, 130.4, 127.7, 126.4, 55.8, 50.9, 31.1, 28.7, 11.9 ppm; HRMS [FT MS ESI+]: m/z $[M + H]^+$ calcd. for $C_{13}H_{19}S_2$ 239.0923, found 239.0918 (−2.1 ppm).

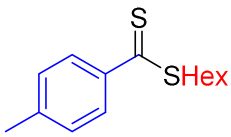

Hexyl 4-methylbenzodithioate (**3a**): Following General Procedure A, 2-(*p* -tolyl)-1,3-dithiolane (**1a**, 0.5 mmol, 98 mg), LiHMDS (0.55 mmol, 92 mg) and hexyl bromide (1.0 mmol, 140 µL) were reacted in 1.0 mL CPME at 100 °C for 2 h. Product **3a** was isolated (101 mg, 80%) as red oil by flash chromatography on silica gel using hexanes as eluent. ^{1}H NMR (500 MHz, $CDCl_3$): δ 7.95 (d, J = 8.0 Hz, 2H), 7.18 (d, J = 8.0 Hz, 2H), 3.37 (t, J = 7.5 Hz, 2H), 2.38 (s, 3H), 1.77 (p, J = 7.5 Hz, 2H), 1.51–1.45 (m, 2H), 1.37–1.33 (m, 4H), 0.94–0.91 (m, 3H) ppm; ^{13}C-APT NMR (125 MHz, $CDCl_3$): δ 228.1, 143.0, 142.7, 128.8, 126.8, 37.2, 31.3, 28.8, 27.3, 22.5, 21.4, 14.0 ppm; HRMS [FT MS ESI+]: m/z [M + H]$^+$ calcd. for $C_{14}H_{21}S_2$ 253.1079 found 253.1074 (−2.0 ppm).

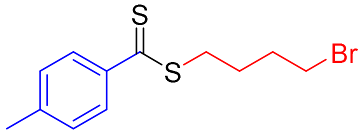

4-Bromobutyl 4-methylbenzodithioate (**4a**): Following General Procedure A, 2-(*p*-tolyl)-1,3-dithiolane (**1a**, 0.5 mmol, 98 mg), LiHMDS (0.55 mmol, 92 mg) and 1,4-dibromobutane (1.0 mmol, 119.5 µL) were reacted in 1.0 mL CPME at 100 °C for 2 h. Product **4a** was isolated (129 mg, 85%) as red oil by flash chromatography on silica gel using hexanes as eluent. ^{1}H NMR (500 MHz, $CDCl_3$): δ 7.94 (d, J = 8.0 Hz, 2H), 7.18 (d, J = 8.0 Hz, 2H), 3.45 (t, J = 6.5 Hz, 2H), 3.40 (t, J = 7.2 Hz, 2H), 2.38 (s, 3H), 2.06–2.00 (m, 2H), 1.95–1.89 (m, 2H) ppm; ^{13}C-APT NMR (125 MHz, $CDCl_3$): δ 227.4, 143.3, 142.5, 128.9, 126.8, 35.8, 32.8, 31.9, 28.8, 26.2, 21.4 ppm; HRMS [TOF MS ES+]: m/z [M + H]$^+$ calcd. for $C_{12}H_{16}S_2{}^{81}Br$ 304.9851, found 304.9855 (1.3 ppm).

2.2. Synthesis of *α*,*α*-bis(thioethyl)methylenecyclohexane 2m'

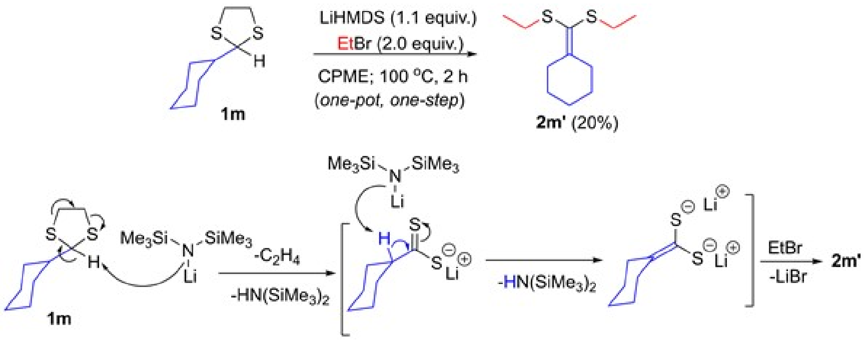

Scheme S2.

Following General Procedure A, 2-cyclohexyl-1,3-dithiolane (**1m**, 0.5 mmol, 94 mg), LiHMDS (0.55 mmol, 92 mg) and ethyl bromide (1.0 mmol, 76 µL) were reacted in 1.0 mL CPME at 100 °C for 2 h. Product **2m'** was isolated (22 mg, 20%) as colorless oil by flash chromatography on silica gel using hexanes as eluent. ^{1}H NMR (500 MHz, $CDCl_3$): δ 2.69 (q, *J* = 7.3 Hz, 4H), 2.66–2.63 (m, 4H), 1.59–1.51 (m, 6H), 1.17 (t, *J* = 7.3 Hz, 6H) ppm; ^{13}C-APT NMR (125 MHz, $CDCl_3$): δ 155.0, 119.5, 34.2, 28.2, 27.1, 26.7, 14.8 ppm; HRMS [FT MS ESI+]: m/z $[M + H]^+$ calcd. for $C_{11}H_{21}S_2$ 217.1079, found 217.1075 (−1.8 ppm).

3. General procedure B (for one-pot, two-step reactions of 2-aryl-1,3-dithiolanes with alkyl halides)

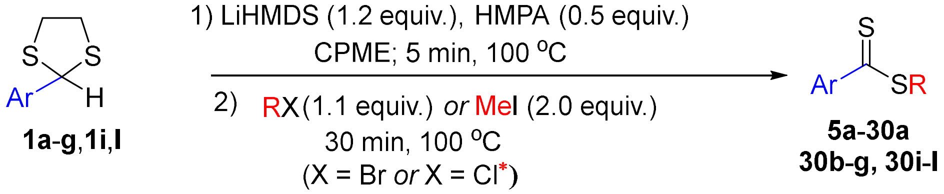

Scheme S3.

An oven-dried 15 mL screw-cap reaction vial equipped with a stirring bar was charged with 2-aryl-1,3-dithiolane derivative (0.5 mmol, 1.0 equiv.). A vial (preferentially a conical bottom vial) was charged with alkyl halide (0.55 mmol, 1.1 equiv.), if it was solid or high boiling point liquid (above 120 °C), and then both vials were put into a glovebox. The reaction vial was charged with LiHMDS (0.6 mmol, 100 mg, 1.2 equiv.), anhydrous CPME (1.0 mL) and then HMPA (0.5 equiv. 0.25 mmol, 44.0 µL) by a micropipette. The solution's color changed to a deep red after HMPA was added. The conical bottom vial containing solid or high boiling point liquid alkyl bromide was charged with CPME (1.0 mL). If alkyl halide was low boiling point liquid such as MeI, the empty conical bottom vial was charged with alkyl halide [(0.55 mmol, 1.1 equiv.; *MeI (1.0 mmol, 2.0 equiv.)*] by a micropipette and CPME (1.0 mL). Both vials were sealed with a rubber septum wrapped with a strip of Parafilm and removed from the glovebox. After having stirred the reaction mixture for 5 min. at 100 °C in a pre-heated oil bath, the solution (1.0 mL CPME) of alkyl halide was added into the reaction mixture by a syringe at 100 °C. After the resulting reaction mixture was stirred for 30 min. at 100 °C, the reaction vial was cooled to room temperature. The reaction mixture was taken into a 50

mL flask and the solvent was removed in a rotatory evaporator. The remaining residue was dissolved in CH_2Cl_2 and mixed with silica gel (about 0.5 g). After evaporating CH_2Cl_2, the remaining silica gel was directly loaded onto a column and purified by flash chromatography on silica gel using hexanes/ethyl acetate mixture as eluent to yield the product.

3.1. Synthesis of dithioesters 5a-30a, 30b-g, 30i-l

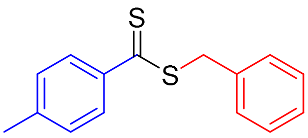

Benzyl 4-methylbenzodithioate (**5a**)[6]: Following General Procedure B, 2-(*p* -tolyl)-1,3-dithiolane (**1a**, 0.5 mmol, 98 mg), LiHMDS (0.60 mmol, 100 mg), HMPA (0.25 mmol, 44 µL) in 1.0 mL CPME were reacted at 100 for 5 minutes, and then benzyl bromide (0.55 mmol, 65 µL) in 1.0 mL CPMA was added into the reaction mixture by a syringe at 100 °C. The resulting mixture was stirred at 100 °C for 30 min. Product **5a** was isolated (106 mg, 82%) as red oil by flash chromatography on silica gel using hexanes as eluent. ^{1}H NMR (500 MHz, $CDCl_3$): δ 7.97 (d, *J* = 8.0 Hz, 2H), 7.42–7.40 (m, 2H), 7.37–7.29 (m, 3H), 7.19 (d, *J* = 8.0 Hz, 2H), 4.61 (s, 2H), 2.39 (s, 3H) ppm; ^{13}C-APT NMR (125 MHz, $CDCl_3$): δ 227.0, 143.4, 142.2, 135.1, 129.3, 129.0, 128.6, 127.6, 126.9, 42,1 21.5 ppm; HRMS [TOF MS ES+]: m/z $[M + H]^+$ calcd. for $C_{15}H_{15}S_2$ 259.0610, found 259.0615 (1.9 ppm).

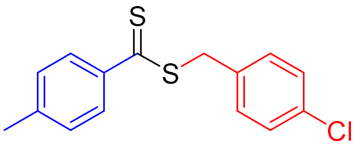

4-Chlorobenzyl 4-methylbenzodithioate (**6a**): Following General Procedure B, 2-(*p* -tolyl)-1,3-dithiolane (**1a**, 0.5 mmol, 98 mg), LiHMDS (0.60 mmol, 100 mg), HMPA (0.25 mmol, 44 µL) in 1.0 mL CPME were reacted at 100 for 5 minutes, and then 1-(bromomethyl)-4-chlorobenzene (0.55 mmol, 113 mg) in 1.0 mL CPMA was added into the reaction mixture by a syringe at 100 °C. The resulting mixture was stirred at 100 °C for 30 min. Product **6a** was isolated (117 mg, 80%) as red oil by flash chromatography on silica gel using hexanes as eluent. ^{1}H NMR (500 MHz, $CDCl_3$): δ 7.95 (d, *J* = 8.3 Hz, 2H), 7.34–7.29 (m, 4H), 7.18 (d, *J* = 8.3 Hz, 2H), 4.57 (s, 2H), 2.38 (s, 3H) ppm; ^{13}C-APT NMR (125 MHz, $CDCl_3$): δ 226.4, 143.6, 142.1, 133.8, 133.5, 130.1, 129.0 128.8, 126.9, 41.0 21.5 ppm; HRMS [FT MS ESI+]: m/z $[M + H]^+$ calcd. for $C_{15}H_{14}ClS_2$ 293.0220, found 293.0215 (−1.7 ppm).

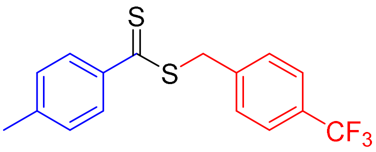

4-(Trifluoromethyl)benzyl 4-methylbenzodithioate (7a): Following General Procedure B, 2-(*p* -tolyl)-1,3-dithiolane (**1a**, 0.5 mmol, 98 mg), LiHMDS (0.60 mmol, 100 mg), HMPA (0.25 mmol, 44 μL) in 1.0 mL CPME were reacted at 100 for 5 minutes, and then 1-(bromomethyl)-4-(trifluoromethyl)benzene (0.55 mmol, 108 mg) in 1.0 mL CPMA was added into the reaction mixture by a syringe at 100 °C. The resulting mixture was stirred at 100 °C for 30 min. Product **7a** was isolated (113 mg, 69%) as pink solid by flash chromatography on silica gel using hexanes as eluent. ^{1}H NMR (500 MHz, $CDCl_3$): δ 7.96 (d, J = 8.0 Hz, 2H), 7.60–7.50 (AB_{system}, δ_A = 7.60, δ_B = 7.51, J_{AB} = 8.0 Hz, 4H), 7.20 (d, J = 8.0 Hz, 2H), 4.66 (s, 2H), 2.39 (s, 3H) ppm; ^{13}C-APT NMR (125 MHz, $CDCl_3$): δ 226.1, 143.7, 142.0, 139.6 (q, J = 1.4 Hz), 129.8 (q, J = 32.4 Hz), 129.5, 129.1 127.0, 125.5 (q, J = 3.8 Hz), 124 (q, J = 272 Hz), 40.8, 21.5 ppm; HRMS [TOF MS ES+]: m/z $[M + H]^+$ calcd. for $C_{16}H_{14}F_3S_2$ 327.0484, found 327.0489 (1.5 ppm).

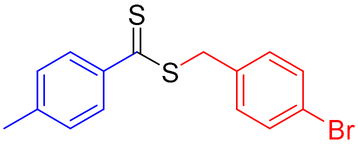

4-Bromobenzyl 4-methylbenzodithioate (8a): Following General Procedure B, 2-(*p* -tolyl)-1,3-dithiolane (**1a**, 0.5 mmol, 98 mg), LiHMDS (0.60 mmol, 100 mg), HMPA (0.25 mmol, 44 μL) in 1.0 mL CPME were reacted at 100 for 5 minutes, and then 1-bromo-4-(bromomethyl)benzene (0.55 mmol, 138 mg) in 1.0 mL CPMA was added into the reaction mixture by a syringe at 100 °C. The resulting mixture was stirred at 100 °C for 30 min. Product **8a** was isolated (140 mg, 83%) as orange-reddish solid by flash chromatography on silica gel using hexanes as eluent. ^{1}H NMR (500 MHz, $CDCl_3$): δ 7.95 (d, J = 8.0 Hz, 2H), 7.46 (d, J = 8.0 Hz, 2H), 7.27 (d, J = 8.3 Hz, 2H), 7.19 (d, J = 8.0 Hz, 2H), 4.56 (s, 2H), 2.39 (s, 3H) ppm; ^{13}C-APT NMR (125 MHz, $CDCl_3$): δ 226.4, 143.6, 142.0, 134.3, 131.7, 130.9, 129.0, 126.9, 121.6, 41.0 21.5 ppm; HRMS [TOF MS ES+]: m/z $[M + H]^+$ calcd. for $C_{15}H_{14}S_2{}^{81}Br$ 338.9700, found 338.9710 (3.0 ppm).

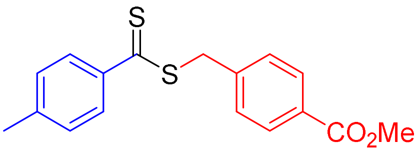

Methyl 4-(((4-methylphenylcarbonothioyl)thio)methyl)-benzoate (9a): Following General Procedure B, 2-(*p*-tolyl)-1,3-dithiolane (**1a**, 0.5 mmol, 98 mg), LiHMDS (0.60 mmol, 100 mg), HMPA (0.25 mmol, 44 μL) in 1.0 mL CPME were reacted at 100 for 5 minutes, and then

methyl 4-(bromomethyl)benzoate (0.55 mmol, 126 mg) in 1.0 mL CPMA was added into the reaction mixture by a syringe at 100 ºC. The resulting mixture was stirred at 100 ºC for 30 min. Product **9a** was isolated (103 mg, 65%) as red solid by flash chromatography on silica gel using 10:1 hexanes/ethyl acetate as eluent. ^{1}H NMR (500 MHz, $CDCl_3$): δ 8.00 (d, *J* = 8.2 Hz, 2H), 7.94 (d, *J* = 8.2 Hz, 2H), 7.46 (d, *J* = 8.2 Hz, 2H), 7.18 (d, *J* = 8.2 Hz, 2H), 4.64 (s, 2H), 3.91 (s, 3H), 2.38 (s, 3H) ppm; ^{13}C-APT NMR (125 MHz, $CDCl_3$): δ 226.3, 166.7, 143.7, 142.1, 140.7, 129.9, 129.5, 129.3, 129.1, 127.0, 52.1, 41.3 21.5 ppm; HRMS [TOF MS ES+]: m/z $[M + H]^+$ calcd. for $C_{17}H_{17}O_2S_2$ 317.0664, found 317.0669 (1.6 ppm).

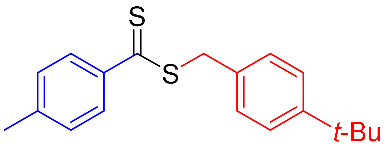

4-(*tert*-Butyl)benzyl 4-methylbenzodithioate (**10a**): Following General Procedure B, 2-(*p* -tolyl)-1,3-dithiolane (**1a**, 0.5 mmol, 98 mg), LiHMDS (0.60 mmol, 100 mg), HMPA (0.25 mmol, 44 µL) in 1.0 mL CPME were reacted at 100 for 5 minutes, and then 1-(bromomethyl)-4-(*tert*-butyl)benzene (0.55 mmol, 101 µL) in 1.0 mL CPMA was added into the reaction mixture by a syringe at 100 ºC. The resulting mixture was stirred at 100 ºC for 30 min. Product **10a** was isolated (126 mg, 80%) as red oil by flash chromatography on silica gel using hexanes as eluent. ^{1}H NMR (500 MHz, $CDCl_3$): δ 7.97 (d, *J* = 8.0 Hz, 2H), 7.40–7.34 (m, 4H), 7.19 (d, *J* = 8.0 Hz, 2H), 4.59 (s, 2H), 2.39 (s, 3H), 1.35 (s, 9H) ppm; ^{13}C-APT NMR (125 MHz, $CDCl_3$): δ 227.2, 150.7, 143.3, 142.2, 131.9, 129.0, 128.9, 126.9, 125.6, 41.8, 34.5, 31.3 21.5 ppm; HRMS [TOF MS ES+]: m/z $[M + H]^+$ calcd. for $C_{19}H_{23}S_2$ 315.1236, found 315.1243 (2.2 ppm).

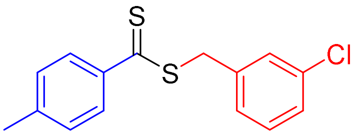

3-Chlorobenzyl 4-methylbenzodithioate (**11a**): Following General Procedure B, 2-(*p*-tolyl)-1,3-dithiolane (**1a**, 0.5 mmol, 98 mg), LiHMDS (0.60 mmol, 100 mg), HMPA (0.25 mmol, 44 µL) in 1.0 mL CPME were reacted at 100 for 5 minutes, and then 1-chloro-3-(chloromethyl)benzene (0.55 mmol, 70 µL) in 1.0 mL CPMA was added into the reaction mixture by a syringe at 100 ºC. The resulting mixture was stirred at 100 ºC for 30 min. Product **11a** was isolated (123 mg, 84%) as red oil by flash chromatography on silica gel using hexanes as eluent. ^{1}H NMR (500 MHz, $CDCl_3$): δ 7.97 (d, *J* = 8.0 Hz, 2H), 7.41 (s, 1H), 7.31–7.27 (m, 3H), 7.20 (d, *J* = 8.0 Hz, 2H), 4.59 (s, 2H), 2.40 (s, 3H) ppm; ^{13}C-

APT NMR (125 MHz, $CDCl_3$): δ 226.2, 143.6, 142.0, 137.3, 134.4, 129.8, 129.2, 129.0, 127.8, 127.4, 126.9, 41.0, 21.5 ppm; HRMS [TOF MS ES+]: m/z $[M + H]^+$ calcd. for $C_{15}H_{14}S_2Cl$ 293.0220, found 293.0226 (2.0 ppm).

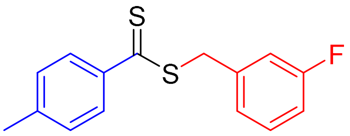

3-Fluorobenzyl 4-methylbenzodithioate (**12a**): Following General Procedure B, 2-(*p*-tolyl)-1,3-dithiolane (**1a**, 0.5 mmol, 98 mg), LiHMDS (0.60 mmol, 100 mg), HMPA (0.25 mmol, 44 μL) in 1.0 mL CPME were reacted at 100 for 5 minutes, and then 1-(chloromethyl)-3-fluorobenzene (0.55 mmol, 66 μL) in 1.0 mL CPMA was added into the reaction mixture by a syringe at 100 °C. The resulting mixture was stirred at 100 °C for 30 min. Product **12a** was isolated (95 mg, 69%) as red oil by flash chromatography on silica gel using hexanes as eluent. ^1H NMR (500 MHz, $CDCl_3$): δ 7.97 (d, *J* = 8.4 Hz, 2H), 7.32–7.28 (m, 1H), 7.20–7.17 (m, 3H), 7.13–7.11 (m, 1H), 7.02–6.98 (m, 1H), 4.60 (s, 2H), 2.39 (s, 3H) ppm; ^{13}C-APT NMR (125 MHz, $CDCl_3$): δ 226.3, 162.7 (d, *J* = 247 Hz), 143.6, 142.0, 137.7 (d, *J* = 7.6 Hz), 130.1 (q, *J* = 8.1 Hz), 129.0, 126.9, 124.9 (d, *J* = 2.9 Hz), 116.1 (d, *J* = 21.6 Hz), 114.6 (d, *J* = 21.0 Hz), 41.1 (d, *J* = 1.9 Hz), 21.5 ppm; HRMS [TOF MS ES+]: m/z $[M + H]^+$ calcd. for $C_{15}H_{14}FS_2$ 277.0515, found 277.0522 (2.5 ppm).

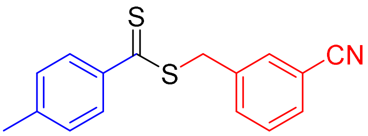

3-Cyanobenzyl 4-methylbenzodithioate (**13a**): Following General Procedure B, 2-(*p*-tolyl)-1,3-dithiolane (**1a**, 0.5 mmol, 98 mg), LiHMDS (0.60 mmol, 100 mg), HMPA (0.25 mmol, 44 μL) in 1.0 mL CPME were reacted at 100 for 5 minutes, and then 3-(bromomethyl)benzonitrile (0.55 mmol, 108 mg) in 1.0 mL CPMA was added into the reaction mixture by a syringe at 100 °C. The resulting mixture was stirred at 100 °C for 30 min. Product **13a** was isolated (99 mg, 70%) as pink-reddish solid by flash chromatography on silica gel using 20:1 hexanes/ethyl acetate as eluent. ^1H NMR (500 MHz, $CDCl_3$): δ 7.95 (d, *J* = 8.0 Hz, 2H), 7.68 (s, 1H), 7.62 (d, *J* = 7.8 Hz, 1H), 7.56 (d, *J* = 7.8 Hz, 1H), 7.43 (t, *J* = 7.8 Hz, 1H), 7.19 (d, *J* = 8.0 Hz, 2H), 4.62 (s, 2H), 2.39 (s, 3H) ppm; ^{13}C-APT NMR (125 MHz, $CDCl_3$): δ 225.5, 143.8, 141.7, 137.3, 133.6, 132.5, 131.1, 129.3, 129.0, 126.9, 118.3, 112.5, 40.1, 21.4 ppm; HRMS [TOF MS ES+]: m/z $[M + H]^+$ calcd. for $C_{16}H_{14}NS_2$ 284.0562, found 284.0565 (1.0 ppm).

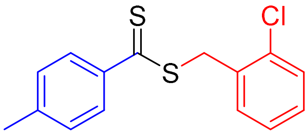

2-Chlorobenzyl 4-methylbenzodithioate (14a): Following General Procedure B, 2-(*p*-tolyl)-1,3-dithiolane (**1a**, 0.5 mmol, 98 mg), LiHMDS (0.60 mmol, 100 mg), HMPA (0.25 mmol, 44 µL) in 1.0 mL CPME were reacted at 100 for 5 minutes, and then 1-chloro-2-(chloromethyl)benzene (0.55 mmol, 68 µL) *or* 1-bromo-2-(chloromethyl)benzene (0.55 mmol, 71 µL) in 1.0 mL CPMA was added into the reaction mixture by a syringe at 100 °C. The resulting mixture was stirred at 100 °C for 30 min. Product **14a** was isolated (112 mg, 77% *or* 124 mg 82% when 1-bromo-2-(chloromethyl)benzene was used) as red oil by flash chromatography on silica gel using hexanes as eluent. ^{1}H NMR (500 MHz, $CDCl_3$): δ 7.98 (d, *J* = 8.3 Hz, 2H), 7.55–7.53 (m, 1H), 7.49–7.42 (m, 1H), 7.28–7.23 (m, 2H), 7.20 (d, *J* = 8.3 Hz, 2H), 4.76 (s, 2H), 2.40 (s, 3H) ppm; ^{13}C-APT NMR (125 MHz, $CDCl_3$): δ 226.5, 143.4, 142.1, 134.5, 133.2, 131.3, 129.6, 129.1, 128.9, 126.93, 126.91, 39.6, 21.5 ppm; HRMS [FT MS ESI+]: m/z [M + H]$^+$ calcd. for $C_{15}H_{14}ClS_2$ 293.0220, found 293.0215 (−1.7 ppm).

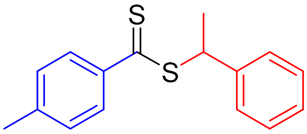

1-Phenylethyl 4-methylbenzodithioate (15a)[7]: Following General Procedure B, 2-(*p*-tolyl)-1,3-dithiolane (**1a**, 0.5 mmol, 98 mg), LiHMDS (0.60 mmol, 100 mg), HMPA (0.25 mmol, 44 µL) in 1.0 mL CPME were reacted at 100 for 5 minutes, and then (1-bromoethyl)benzene (0.55 mmol, 75 µL) in 1.0 mL CPMA was added into the reaction mixture by a syringe at 100 °C. The resulting mixture was stirred at 100 °C for 30 min. Product **15a** was isolated (106 mg, 78%) as red solid by flash chromatography on silica gel using hexanes as eluent. ^{1}H NMR (500 MHz, $CDCl_3$): δ 7.94 (d, *J* = 8.0 Hz, 2H), 7.48 (d, *J* = 7.5 Hz, 2H), 7.39–7.36 (m, 2H), 7.32–7.30 (m, 1H), 7.18 (d, *J* = 8.0 Hz, 2H), 5.32 (q, *J* = 7.1 Hz, 1H), 2.39 (s, 3H), 1.84 (d, *J* = 7.1 Hz, 3H) ppm; ^{13}C-APT NMR (125 MHz, $CDCl_3$): δ 226.1, 143.2, 142.4, 141.3, 128.9, 128.6, 127.8, 127.6, 126.9, 45.0, 21.4, 20.8 ppm; HRMS [TOF MS ES+]: m/z [M + H]$^+$ calcd. for $C_{16}H_{17}S_2$ 273.0766, found 273.0759 (−2.6 ppm).

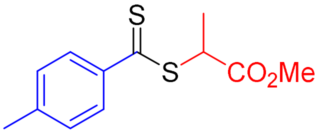

Methyl 2-((4-methylphenylcarbonothioyl)thio)propanoate (16a): Following General Procedure B, 2-(*p*-tolyl)-1,3-dithiolane (**1a**, 0.5 mmol, 98 mg), LiHMDS (0.60 mmol, 100 mg), HMPA (0.25 mmol, 44 µL) in 1.0 mL CPME were reacted at 100 for 5 minutes, and then methyl 2-bromopropanoate (0.55

mmol, 62 μL) in 1.0 mL CPMA was added into the reaction mixture by a syringe at 100 °C. The resulting mixture was stirred at 100 °C for 30 min. Product **16a** was isolated (100 mg, 79%) as red oil by flash chromatography on silica gel using hexanes as eluent. ^{1}H NMR (500 MHz, $CDCl_3$): δ 7.93 (d, *J* = 8.0 Hz, 2H), 7.17 (d, *J* = 8.0 Hz, 2H), 4.77 (q, *J* = 7.4 Hz, 1H), 3.76 (s, 3H), 2.37 (s, 3H), 1.66 (d, *J* = 7.4 Hz, 3H) ppm; ^{13}C-APT NMR (125 MHz, $CDCl_3$): δ 225.0, 171.7, 143.7, 141.7, 129.0, 126.9, 52.8, 48.1, 21.5, 16.5 ppm; HRMS [TOF MS ES+]: m/z $[M + H]^+$ calcd. for $C_{12}H_{15}O_2S_2$ 255.0508, found 255.0516 (3.1 ppm).

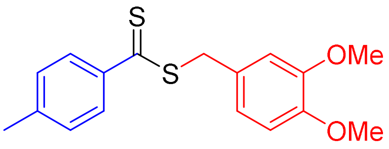

3,4-Dimethoxybenzyl 4-methylbenzodithioate (**17a**): Following General Procedure B, 2-(*p*-tolyl)-1,3-dithiolane (**1a**, 0.5 mmol, 98 mg), LiHMDS (0.60 mmol, 100 mg), HMPA (0.25 mmol, 44 μL) in 1.0 mL CPME were reacted at 100 for 5 minutes, and then 4-(chloromethyl)-1,2-dimethoxybenzene (0.55 mmol, 103 mg) in 1.0 mL CPMA was added into the reaction mixture by a syringe at 100 °C. The resulting mixture was stirred at 100 °C for 30 min. Product **17a** was isolated (127 mg, 80%) as orange reddish solid by flash chromatography on silica gel using 10:1 hexanes/ethyl acetate as eluent. ^{1}H NMR (500 MHz, $CDCl_3$): δ 7.94 (d, *J* = 8.4 Hz, 2H), 7.17 (d, *J* = 8.0 Hz, 2H), 6.96–6.91 (m, 2H), 6.82 (d, *J* = 8.2 Hz, 2H), 4.55 (s, 2H), 3.87 (s, 6H), 2.37 (s, 3H) ppm; ^{13}C-APT NMR (125 MHz, $CDCl_3$): δ 227.3, 149.0, 148.6, 143.4, 142.3, 129.0, 127.3, 126.9, 121.6, 112.3, 111.2, 55.9, 55.8, 42.2, 21.5 ppm; HRMS [TOF MS ES+]: m/z $[M + H]^+$ calcd. for $C_{17}H_{19}O_2S_2$ 319.0826, found 319.0837 (3.4 ppm).

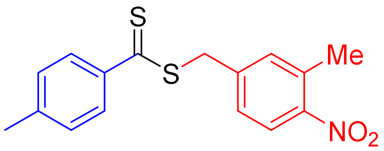

3-Methyl-4-nitrobenzyl 4-methylbenzodithioate (**18a**): Following General Procedure B, 2-(*p*-tolyl)-1,3-dithiolane (**1a**, 0.5 mmol, 98 mg), LiHMDS (0.60 mmol, 100 mg), HMPA (0.25 mmol, 44 μL) in 1.0 mL CPME were reacted at 100 for 5 minutes, and then 4-(bromomethyl)-2-methyl-1-nitrobenzene (0.55 mmol, 127 mg) in 1.0 mL CPMA was added into the reaction mixture by a syringe at 100 °C. The resulting mixture was stirred at 100 °C for 30 min. Product **18a** was isolated (103 mg, 65%) as orange reddish solid by flash chromatography on silica gel using 10:1 hexanes/ethyl acetate as eluent. ^{1}H NMR (500 MHz, $CDCl_3$): δ 7.95–7.93 (m, 3H), 7.35–7.34 (m, 2H), 7.19 (d, *J* =

8.2 Hz, 2H), 4.62 (s, 2H), 2.59 (s, 3H), 2.38 (s, 3H) ppm; ^{13}C-APT NMR (125 MHz, $CDCl_3$): δ 225.6, 148.2, 143.9, 141.8, 141.4, 134.0, 133.4, 129.1, 127.6, 127.0, 125.0, 40.3, 21.5, 20.5 ppm; HRMS [TOF MS ES+]: m/z $[M + H]^+$ calcd. for $C_{16}H_{16}NO_2S_2$ 318.0617, found 318.0610 (1.6 ppm).

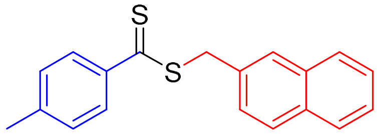

Naphthalen-2-ylmethyl 4-methylbenzodithioate (**19a**): Following General Procedure B, 2-(*p*-tolyl)-1,3-dithiolane (**1a**, 0.5 mmol, 98 mg), LiHMDS (0.60 mmol, 100 mg), HMPA (0.25 mmol, 44 μL) in 1.0 mL CPME were reacted at 100 for 5 minutes, and then 2-(bromomethyl)naphthalene (0.55 mmol, 122 mg) in 1.0 mL CPMA was added into the reaction mixture by a syringe at 100 ºC. The resulting mixture was stirred at 100 ºC for 30 min. Product **19a** was isolated (113 mg, 73%) as pink solid by flash chromatography on silica gel using hexanes as eluent. ^{1}H NMR (500 MHz, $CDCl_3$): δ 7.97 (d, *J* = 8.0 Hz, 2H), 7.87 (s, 1H), 7.84–7.81 (m, 3H), 7.52–7.46 (m, 3H), 7.19 (d, *J* = 8.0 Hz, 2H), 4.78 (s, 2H), 2.38 (s, 3H) ppm; ^{13}C-APT NMR (125 MHz, $CDCl_3$): δ 226.9, 143.4, 142.3, 133.3, 132.8, 132.6, 129.0, 128.5, 128.2, 127.7, 127.6, 127.1, 126.9, 126.3, 126.1, 42.3, 21.5 ppm; HRMS [TOF MS ES+]: m/z $[M + H]^+$ calcd. for $C_{19}H_{17}S_2$ 309.0766, found 309.0776 (3.2 ppm).

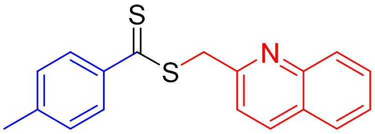

Quinolin-2-ylmethyl 4-methylbenzodithioate (**20a**): Following General Procedure B, 2-(*p*-tolyl)-1,3-dithiolane (**1a**, 0.5 mmol, 98 mg), LiHMDS (0.60 mmol, 100 mg), HMPA (0.25 mmol, 44 μL) in 1.0 mL CPME were reacted at 100 for 5 minutes, and then 2-(bromomethyl)quinoline (0.55 mmol, 122 mg) in 1.0 mL CPMA was added into the reaction mixture by a syringe at 100 ºC. The resulting mixture was stirred at 100 ºC for 30 min. Product **20a** was isolated (102 mg, 66%) as red oil by flash chromatography on silica gel using 10:1 hexanes/ethyl acetate as eluent. ^{1}H NMR (500 MHz, $CDCl_3$): δ 8.11 (d, *J* = 8.5 Hz, 1H), 8.08 (d, *J* = 8.6 Hz, 1H), 7.98 (d, *J* = 8.3 Hz, 2H), 7.80 (d, *J* = 8.2 Hz, 1H), 7.74–7.70 (m, 1H), 7.55–7.51 (m, 2H), 7.18 (d, *J* = 7.8 Hz, 2H), 4.99 (s, 2H), 2.37 (s, 3H) ppm; ^{13}C-APT NMR (125 MHz, $CDCl_3$): δ 226.5, 156.2, 147.8, 143.6, 142.2, 136.9, 129.8, 129.0, 128.9, 127.5, 127.2, 127.1, 126.6, 121.4, 44.0, 21.5 ppm; HRMS [FT MS ESI+]: m/z $[M + H]^+$ calcd. for $C_{18}H_{16}NS_2$ 310.0719, found 310.0714 (−1.6 ppm).

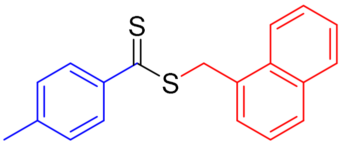

Naphthalen-1-ylmethyl 4-methylbenzodithioate (**21a**): Following General Procedure B, 2-(*p*-tolyl)-1,3-dithiolane (**1a**, 0.5 mmol, 98 mg), LiHMDS (0.60 mmol, 100 mg), HMPA (0.25 mmol, 44 μL) in 1.0 mL CPME were reacted at 100 for 5 minutes, and then 1-(bromomethyl)naphthalene (0.55 mmol, 122 mg) in 1.0 mL CPMA was added into the reaction mixture by a syringe at 100 °C. The resulting mixture was stirred at 100 °C for 30 min. Product **21a** was isolated (125 mg, 81%) as orange solid by flash chromatography on silica gel using hexanes as eluent. ^{1}H NMR (500 MHz, $CDCl_3$): δ 8.02 (d, J = 8.0 Hz, 1H), 7.97 (d, J = 8.3 Hz, 2H), 7.92–7.90 (m, 1H), 7.85 (d, J = 8.4 Hz, 1H), 7.62 (d, J = 7.0 Hz, 1H), 7.58–7.52 (m, 2H), 7.45 (dd, J = 7.0, 8.3 Hz, 1H), 7.17 (d, J = 8.0 Hz, 2H), 5.06 (s, 2H), 2.37 (s, 3H) ppm; ^{13}C-APT NMR (125 MHz, $CDCl_3$): δ 227.1, 143.4, 142.1, 133.8, 131.8, 130.6, 129.0, 128.84, 128.81, 128.2, 127.0, 126.6, 126.0, 125.4, 123.6, 40.4, 21.5 ppm; HRMS [TOF MS ES+]: m/z $[M + H]^+$ calcd. for $C_{19}H_{17}S_2$ 309.0766, found 309.0772 (1.9 ppm).

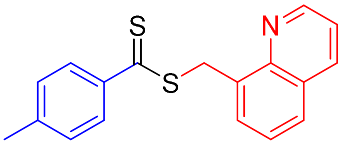

Quinolin-8-ylmethyl 4-methylbenzodithioate (**22a**): Following General Procedure B, 2-(*p*-tolyl)-1,3-dithiolane (**1a**, 0.5 mmol, 98 mg), LiHMDS (0.60 mmol, 100 mg), HMPA (0.25 mmol, 44 μL) in 1.0 mL CPME were reacted at 100 for 5 minutes, and then 8-(bromomethyl)quinoline (0.55 mmol, 122 mg) in 1.0 mL CPMA was added into the reaction mixture by a syringe at 100 °C. The resulting mixture was stirred at 100 °C for 30 min. Product **22a** was isolated (125 mg, 81%) as red solid by flash chromatography on silica gel using 10:1 hexanes/ethyl acetate as eluent. ^{1}H NMR (500 MHz, $CDCl_3$): δ 8.97 (dd, J = 1.7, 4.2 Hz, 1H), 8.12 (dd, J = 1.8, 8.2 Hz, 1H), 7.94 (d, J = 8.4 Hz, 2H), 7.91 (d, J = 7.2 Hz, 1H), 7.74 (dd, J = 1.7, 8.3 Hz, 1H), 7.47 (dd, J = 7.0, 8.2 Hz, 1H), 7.41 (dd, J = 4.2, 8.3 Hz, 1H), 7.12 (d, J = 7.9 Hz, 2H), 5.33 (s, 2H), 2.33 (s, 3H) ppm; ^{13}C-APT NMR (125 MHz, $CDCl_3$): δ 227.6, 149.7, 146.5, 142.9, 142.4, 136.2, 134.1, 130.2, 128.7, 128.3, 127.9, 127.1, 126.9, 126.1, 121.2, 37.7, 21.3 ppm; HRMS [TOF MS ES+]: m/z $[M + H]^+$ calcd. for $C_{18}H_{16}NS_2$ 310.0719, found 310.0711 (−2.6 ppm).

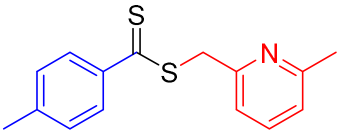

(6-Methylpyridin-2-yl)methyl 4-methylbenzodithioate (23a): Following General Procedure B, 2-(*p*-tolyl)-1,3-dithiolane (**1a**, 0.5 mmol, 98 mg), LiHMDS (0.60 mmol, 100 mg), HMPA (0.25 mmol, 44 μL) in 1.0 mL CPME were reacted at 100 for 5 minutes, and then 2-(bromomethyl)-6-methylpyridine (0.55 mmol, 102 mg) in 1.0 mL CPMA was added into the reaction mixture by a syringe at 100 °C. The resulting mixture was stirred at 100 °C for 30 min. Product **23a** was isolated (94 mg, 69%) as red oil by flash chromatography on silica gel using 10:1 hexanes/ethyl acetate as eluent. ^{1}H NMR (500 MHz, $CDCl_3$): δ 7.94 (d, *J* = 8.3 Hz, 2H), 7.49 (t, *J* = 7.7 Hz, 1H), 7.20 (d, *J* = 7.7 Hz, 1H), 7.14 (d, *J* = 7.8 Hz, 2H), 7.02 (d, *J* = 7.7 Hz, 1H), 4.72 (s, 2H), 2.54 (s, 3H), 2.34 (s, 3H) ppm; ^{13}C-APT NMR (125 MHz, $CDCl_3$): δ 226.5, 158.3, 154.6, 143.3, 142.1, 136.8, 128.9, 126.9, 122.0, 120.5, 43.6, 24.3, 21.4 ppm; HRMS [TOF MS ES+]: m/z $[M + H]^+$ calcd. for $C_{15}H_{16}NS_2$ 274.0719, found 274.0712 (−2.6 ppm).

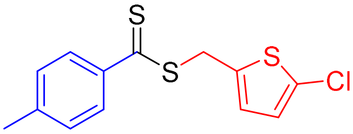

(5-Chlorothiophen-2-yl)methyl 4-methylbenzodithioate (24a): Following General Procedure B, 2-(*p*-tolyl)-1,3-dithiolane (**1a**, 0.5 mmol, 98 mg), LiHMDS (0.60 mmol, 100 mg), HMPA (0.25 mmol, 44 μL) in 1.0 mL CPME were reacted at 100 for 5 minutes, and then 2-chloro-5-(chloromethyl)thiophene (0.55 mmol, 66 μL) in 1.0 mL CPMA was added into the reaction mixture by a syringe at 100 °C. The resulting mixture was stirred at 100 °C for 30 min. Product **24a** was isolated (123 mg, 82%) as orange solid by flash chromatography on silica gel using hexanes as eluent. ^{1}H NMR (500 MHz, $CDCl_3$): δ 7.94 (d, *J* = 8.2 Hz, 2H), 7.19 (d, *J* = 8.2 Hz, 2H), 6.84 (d, *J* = 3.7 Hz, 1H), 6.74 (d, *J* = 3.7 Hz, 1H), 4.73 (s, 2H), 2.39 (s, 3H) ppm; ^{13}C-APT NMR (125 MHz, $CDCl_3$): δ 225.6, 143.7, 141.9, 136.8, 129.5, 129.0, 127.0, 125.6, 36.1, 21.5 ppm; HRMS [TOF MS ES+]: m/z $[M + H]^+$ calcd. for $C_{13}H_{12}S_3Cl$ 298.9784, found 298.9786 (0.7 ppm).

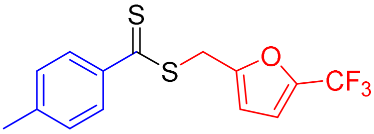

(5-(Trifluoromethyl)furan-2-yl)methyl 4-methylbenzodithioate (25a): Following General Procedure B, 2-(*p*-tolyl)-1,3-dithiolane (**1a**, 0.5 mmol, 98 mg), LiHMDS (0.60 mmol, 100 mg), HMPA (0.25 mmol, 44 μL) in 1.0 mL CPME were reacted at 100 for 5 minutes, and then 2-(bromomethyl)-5-

(trifluoromethyl)furan (0.55 mmol, 74 μL) in 1.0 mL CPMA was added into the reaction mixture by a syringe at 100 ºC. The resulting mixture was stirred at 100 ºC for 30 min. Product **25a** was isolated (123 mg, 78%) as pink reddish solid by flash chromatography on silica gel using hexanes as eluent. ^{1}H NMR (500 MHz, $CDCl_3$): δ 7.96 (d, *J* = 8.2 Hz, 2H), 7.20 (d, *J* = 8.2 Hz, 2H), 6.73 (d, *J* = 3.5 Hz, 1H), 6.42 (d, *J* = 3.5 Hz, 1H), 4.68 (s, 2H), 2.39 (s, 3H) ppm; ^{13}C-APT NMR (125 MHz, $CDCl_3$): δ 225.1, 152.0 (q, *J* = 1.5 Hz), 143.9, 141.9, 141.4 (q, *J* = 43.0 Hz), 129.1, 127.0, 119.0 (q, *J* = 267 Hz), 112.5 (q, *J* = 2.9 Hz), 109.7, 33.1, 21.4 ppm; HRMS [TOF MS ES+]: m/z $[M + H]^+$ calcd. for $C_{14}H_{12}OF_3S_2$ 317.0276, found 317.0287 (3.5 ppm).

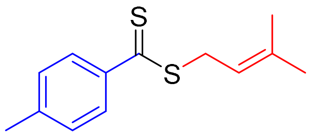

3-Methylbut-2-en-1-yl 4-methylbenzodithioate (**26a**): Following General Procedure B, 2-(*p*-tolyl)-1,3-dithiolane (**1a**, 0.5 mmol, 98 mg), LiHMDS (0.60 mmol, 100 mg), HMPA (0.25 mmol, 44 μL) in 1.0 mL CPME were reacted at 100 for 5 minutes, and then 1-bromo-3-methylbut-2-ene (0.55 mmol, 63.5 μL) in 1.0 mL CPMA was added into the reaction mixture by a syringe at 100 ºC. The resulting mixture was stirred at 100 ºC for 30 min. Product **26a** was isolated (92 mg, 78%) as red oil by flash chromatography on silica gel using hexanes as eluent. ^{1}H NMR (500 MHz, $CDCl_3$): δ 7.95 (d, *J* = 8.0 Hz, 2H), 7.18 (d, *J* = 8.0 Hz, 2H), 5.41–5.37 (m, 1H), 4.00 (q, *J* = 7.8 Hz, 2H), 2.38 (s, 3H), 1.79 (s, 3H), 1.76 (s, 3H) ppm; ^{13}C-APT NMR (125 MHz, $CDCl_3$): δ 228.2, 143.1, 142.4, 138.8, 128.9, 126.8, 116.1, 36.2, 25.7, 21.4, 18.1 ppm; HRMS [FT MS ESI+]: m/z $[M + H]^+$ calcd. for $C_{13}H_{17}S_2$ 237.0766, found 237.0760 (−2.5 ppm).

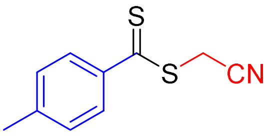

Cyanomethyl 4-methylbenzodithioate (**27a**): Following General Procedure B, 2-(*p*-tolyl)-1,3-dithiolane (**1a**, 0.5 mmol, 98 mg), LiHMDS (0.60 mmol, 100 mg), HMPA (0.25 mmol, 44 μL) in 1.0 mL CPME were reacted at 100 for 5 minutes, and then 2-bromoacetonitrile (0.55 mmol, 38 μL) in 1.0 mL CPMA was added into the reaction mixture by a syringe at 100 ºC. The resulting mixture was stirred at 100 ºC for 30 min. Product **27a** was isolated (68 mg, 66%) as red oil by flash chromatography on silica gel using hexanes as eluent. ^{1}H NMR (500 MHz, $CDCl_3$): δ 7.93 (d, *J* = 8.0 Hz, 2H), 7.22 (d, *J* = 8.0 Hz, 2H), 4.16 (s, 2H), 2.39 (s, 3H) ppm; ^{13}C-APT NMR (125 MHz, $CDCl_3$): δ 221.5, 144.7, 140.9, 129.3,

127.0, 114.7, 21.8, 21.6 ppm; HRMS [FT MS ESI+]: m/z $[M + H]^+$ calcd. for $C_{10}H_{10}NS_2$ 208.0249, found 208.0247 (−1.0 ppm).

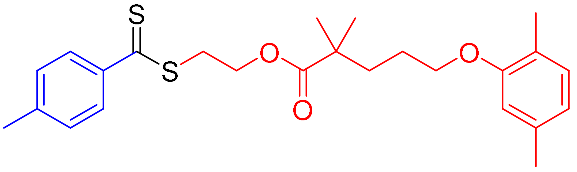

2-((4-methylphenylcarbonothioyl)thio)ethyl5-(2,5-dimethylphenoxy)-2,2-dimethylpentanoate (28a): Following General Procedure B, 2-(*p*-tolyl)-1,3-dithiolane (**1a**, 0.5 mmol, 98 mg), LiHMDS (0.60 mmol, 100 mg), HMPA (0.25 mmol, 44 µL) in 1.0 mL CPME were reacted at 100 for 5 minutes, and then 2-bromoethyl 5-(2,5-dimethylphenoxy)-2,2-dimethylpentanoate[14] (0.55 mmol, 197 mg) in 1.0 mL CPMA was added into the reaction mixture by a syringe at 100 °C. The resulting mixture was stirred at 100 °C for 30 min. Product **28a** was isolated (160 mg, 72%) as red oil by flash chromatography on silica gel using 60:1 hexanes/ethyl acetate as eluent. ^{1}H NMR (500 MHz, $CDCl_3$): δ 7.93 (d, J = 8.1 Hz, 2H), 7.15 (d, J = 8.1 Hz, 2H), 7.01 (d, J = 7.5 Hz, 1H), 6.67 (d, J = 8.0 Hz, 1H), 6.01 (s, 1H), 4.39 (t, J = 6.2 Hz, 2H), 3.95–3.91 (m, 2H), 3.71 (t, J = 6.2 Hz, 2H), 2.37 (s, 3H), 2.32 (s, 3H), 2.19 (s, 3H), 1.78–1.73 (m, 4H), 1.25 (s, 6H) ppm; ^{13}C-APT NMR (125 MHz, $CDCl_3$): δ 226.6, 177.4, 156.9, 143.6, 142.4, 136.4, 130.2, 129.0, 126.7, 123.5, 120.6, 111.9, 67.8, 61.3, 42.1, 37.0, 35.4, 25.2, 25.1, 21.5, 21.4 15.7 ppm.

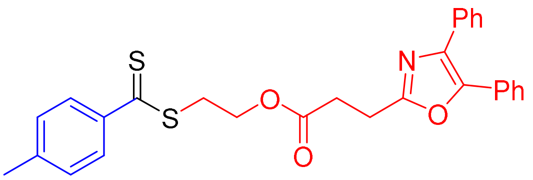

2-((4-methylphenylcarbonothioyl)thio)ethyl 3-(4,5-diphenyloxazol-2-yl)propanoate (29a): Following General Procedure B, 2-(*p*-tolyl)-1,3-dithiolane (**1a**, 0.5 mmol, 98 mg), LiHMDS (0.60 mmol, 100 mg), HMPA (0.25 mmol, 44 µL) in 1.0 mL CPME were reacted at 100 for 5 minutes, and then 2-bromoethyl 3-(4,5-diphenyloxazol-2-yl)propanoate[14] (0.55 mmol, 220 mg) in 1.0 mL CPMA was added into the reaction mixture by a syringe at 100 °C. The resulting mixture was stirred at 100 °C for 30 min. Product **29a** was isolated (127 mg, 52%) as red oil by flash chromatography on silica gel using 20:1 hexanes/ethyl acetate as eluent. ^{1}H NMR (500 MHz, $CDCl_3$): δ 7.91 (d, J = 8.0 Hz, 2H), 7.65–7.56 (m, 4H), 7.37–7.30 (m, 6H), 7.15 (d, J = 8.0 Hz, 2H), 4.43 (t, J = 6.3 Hz, 2H), 3.69 (t, J = 6.3 Hz, 2H), 3.21 (t, J = 7.5 Hz, 2H), 2.96 (t, J = 7.5 Hz, 2H), 2.37 (s, 3H) ppm; ^{13}C-APT NMR (125 MHz, $CDCl_3$): δ 226.5, 171.6, 161.5, 145.1, 143.6, 142.3, 135.1,

132.4, 129.0, 128.9, 128.6, 128.5, 128.4, 128.0, 127.8, 126.9, 126.4, 61.7, 35.1, 31.0, 23.4, 21.5 ppm; HRMS [FT MS ESI+]: m/z $[M + H]^+$ calcd. for $C_{28}H_{26}O_3NS_2$ 488.1349, found 488.1343 (−1.2 ppm).

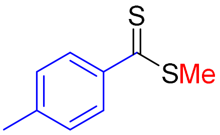

Methyl 4-methylbenzodithioate (**30a**)[8]: Following General Procedure B, 2-(*p* -tolyl)-1,3-dithiolane (**1a**, 0.5 mmol, 98 mg), LiHMDS (0.60 mmol, 100 mg), HMPA (0.25 mmol, 44 μL) in 1.0 mL CPME were reacted at 100 for 5 minutes, and then methyl iodide (1.00 mmol, 62 μL) in 1.0 mL CPMA was added into the reaction mixture by a syringe at 100 °C. The resulting mixture was stirred at 100 °C for 30 min. Product **30a** was isolated (74 mg, 81%) as red oil by flash chromatography on silica gel using hexanes as eluent. ^{1}H NMR (500 MHz, $CDCl_3$): δ 7.96 (d, *J* = 8.0 Hz, 2H), 7.19 (d, *J* = 8.0 Hz, 2H), 2.78 (s, 3H), 2.38 (s, 3H) ppm; ^{13}C-APT NMR (125 MHz, $CDCl_3$): δ 229.0, 143.2, 142.5, 128.9, 126.8, 21.5, 20.5 ppm; HRMS [FT MS ESI+]: m/z $[M + H]^+$ calcd. for $C_9H_{11}S_2$ 183.0297, found 183.0295 (−1.1 ppm).

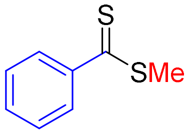

Methyl benzodithioate (**30b**)[8]: Following General Procedure B, 2-phenyl-1,3-dithiolane (**1b**, 0.5 mmol, 91 mg), LiHMDS (0.60 mmol, 100 mg), HMPA (0.25 mmol, 44 μL) in 1.0 mL CPME were reacted at 100 for 5 minutes, and then methyl iodide (1.00 mmol, 62 μL) in 1.0 mL CPMA was added into the reaction mixture by a syringe at 100 °C. The resulting mixture was stirred at 100 °C for 30 min. Product **30b** was isolated (70 mg, 83%) as red oil by flash chromatography on silica gel using hexanes as eluent. ^{1}H NMR (500 MHz, $CDCl_3$): 8.04–8.02 (m, 2H), 7.56–7.52 (m, 1H), 7.45–7.38 (m, 2H), 2.79 (s, 3H) ppm; ^{13}C-APT NMR (125 MHz, $CDCl_3$): δ 229.1, 143.9, 132.2, 128.2, 126.7, 20.6 ppm; HRMS [TOF MS ES+]: m/z $[M]^+$ calcd. for $C_8H_8S_2$ 168.0062, found 168.0059 (–1.8 ppm).

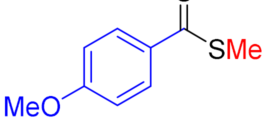

Methyl 4-methoxybenzodithioate (**30c**)[9]: Following General Procedure B, 2-(4-methoxyphenyl)-1,3-dithiolane (**1c**, 0.5 mmol, 106 mg), LiHMDS (0.60 mmol, 100 mg), HMPA (0.25 mmol, 44 μL) in 1.0 mL CPME were reacted at 100 for 5 minutes, and then methyl iodide (1.00 mmol, 62 μL) in 1.0 mL CPMA was added into the reaction mixture by a syringe at 100 °C. The resulting mixture was stirred at 100 °C for 30 min. Product **30c** was isolated (84 mg, 85%) as red solid by flash chromatography on silica gel using hexanes as eluent. ^{1}H NMR (500 MHz, $CDCl_3$): δ 8.10 (d, *J* = 9.0 Hz, 2H), 6.87 (d, *J* = 9.0 Hz, 2H),

3.85 (s, 3H), 2.76 (s, 3H) ppm; ^{13}C-APT NMR (125 MHz, $CDCl_3$): δ 226.7, 163.4, 138.0, 128.9, 113.4, 59.4, 20.3 ppm; HRMS [TOF MS ES+]: m/z $[M + H]^+$ calcd. for $C_9H_{11}OS_2$ 199.0246, found 199.0246 (0.0 ppm).

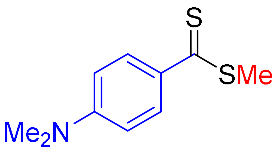

Methyl 4-(dimethylamino)benzodithioate (**30d**)[8]: Following General Procedure B, 2-(4-dimethylaminophenyl)-1,3-dithiolane (**1d**, 0.5 mmol, 113 mg), LiHMDS (0.60 mmol, 100 mg), HMPA (0.25 mmol, 44 µL) in 1.0 mL CPME were reacted at 100 for 5 minutes, and then methyl iodide (1.00 mmol, 62 µL) in 1.0 mL CPMA was added into the reaction mixture by a syringe at 100 ºC. The resulting mixture was stirred at 100 ºC for 30 min. Product **30d** was isolated (82 mg, 78%) as orange solid by flash chromatography on silica gel using hexanes as eluent. ^{1}H NMR (500 MHz, $CDCl_3$): δ 8.15 (d, J = 9.1 Hz, 2H), 6.61 (d, J = 9.1 Hz, 2H), 3.06 (s, 6H), 2.76 (s, 3H) ppm; ^{13}C-APT NMR (125 MHz, $CDCl_3$): δ 224.3, 153.6, 133.6, 129.2, 110.5, 40.1, 19.7 ppm; HRMS [TOF MS ES+]: m/z $[M + H]^+$ calcd. for $C_{10}H_{14}NS_2$ 212.0562, found 212.0567 (2.4 ppm).

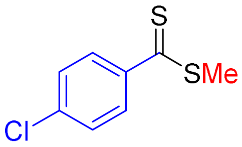

Methyl 4-chlorobenzodithioate (**30e**)[8]: Following General Procedure B, 2-(4-chlorophenyl)-1,3-dithiolane (**1e**, 0.5 mmol, 108 mg), LiHMDS (0.60 mmol, 100 mg), HMPA (0.25 mmol, 44 µL) in 1.0 mL CPME were reacted at 100 for 5 minutes, and then methyl iodide (1.00 mmol, 62 µL) in 1.0 mL CPMA was added into the reaction mixture by a syringe at 100 ºC. The resulting mixture was stirred at 100 ºC for 30 min. Product **30e** was isolated (74 mg, 73%) as red oil by flash chromatography on silica gel using hexanes as eluent. ^{1}H NMR (500 MHz, $CDCl_3$): δ 7.96 (d, J = 8.7 Hz, 2H), 7.36 (d, J = 8.7 Hz, 2H), 2.77 (s, 3H) ppm; ^{13}C-APT NMR (125 MHz, $CDCl_3$): δ 227.0, 143.0, 138.7, 128.5, 128.0, 20.7 ppm; HRMS [FT MS ESI+]: m/z $[M + H]^+$ calcd. for $C_8H_8ClS_2$ 202.9751, found 202.9748 (−1.5 ppm).

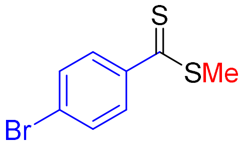

Methyl 4-bromobenzodithioate (**30f**)[8]: Following General Procedure B, 2-(4-bromophenyl)-1,3-dithiolane (**1f**, 0.5 mmol, 131 mg), LiHMDS (0.60 mmol, 100 mg), HMPA (0.25 mmol, 44 µL) in 1.0 mL CPME were reacted at 100 for 5 minutes, and then methyl iodide (1.00 mmol, 62 µL) in 1.0 mL CPMA was added into the reaction mixture by a syringe at 100 ºC. The resulting mixture was stirred at 100 ºC for 30 min. Product **30f**

was isolated (111 mg, 90%) as red solid by flash chromatography on silica gel using hexanes as eluent. ^{1}H NMR (500 MHz, $CDCl_3$): δ 7.87 (d, *J* = 8.6 Hz, 2H), 7.51 (d, *J* = 8.6 Hz, 2H), 2.76 (s, 3H) ppm; ^{13}C-APT NMR (125 MHz, $CDCl_3$): δ 226.9, 143.3, 131.4, 128.1, 127.3, 20.6 ppm; HRMS [TOF MS ES+]: m/z $[M + H]^+$ calcd. for $C_8H_8S_2{}^{81}Br$ 248.9230, found 248.9218 (−4.8 ppm).

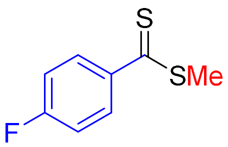

Methyl 4-fluorobenzodithioate (**30g**)[8]: Following General Procedure B, 2-(4-fluorophenyl)-1,3-dithiolane (**1g**, 0.5 mmol, 100 mg), LiHMDS (0.60 mmol, 100 mg), HMPA (0.25 mmol, 44 µL) in 1.0 mL CPME were reacted at 100 for 5 minutes, and then methyl iodide (1.00 mmol, 62 µL) in 1.0 mL CPMA was added into the reaction mixture by a syringe at 100 °C. The resulting mixture was stirred at 100 °C for 30 min. Product **30g** was isolated (80 mg, 86%) as red oil by flash chromatography on silica gel using hexanes as eluent. ^{1}H NMR (500 MHz, $CDCl_3$): δ 8.08–8.04 (m, 2H), 7.09–7.04 (m, 2H), 2.77 (s, 3H) ppm; ^{13}C-APT NMR (125 MHz, $CDCl_3$): δ 226.9, 165.5 (d, *J* = 254.6 Hz), 141.2 (d, *J* = 3.3 Hz), 129.0 (d, *J* = 8.6 Hz), 115.2 (d, *J* = 21.9 Hz), 20.7 ppm; HRMS [TOF MS ES+]: m/z $[M + H]^+$ calcd. for $C_8H_8FS_2$ 187.0046, found 187.0042 (−2.1 ppm).

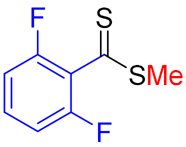

Methyl 2,6-difluorobenzodithioate (**30i**): Following General Procedure B, 2-(2,6-difluorophenyl)-1,3-dithiolane (**1i**, 0.5 mmol, 109 mg), LiHMDS (0.60 mmol, 100 mg), HMPA (0.25 mmol, 44 µL) in 1.0 mL CPME were reacted at 100 for 5 minutes, and then methyl iodide (1.00 mmol, 62 µL) in 1.0 mL CPMA was added into the reaction mixture by a syringe at 100 °C. The resulting mixture was stirred at 100 °C for 30 min. Product **30i** was isolated (78 mg, 76%) as orange solid by flash chromatography on silica gel using hexanes as eluent. ^{1}H NMR (500 MHz, $CDCl_3$): δ 7.35–7.29 (m, 1H), 6.96–6.92 (m, 2H), 2.81 (s, 3H) ppm; ^{13}C-APT NMR (125 MHz, $CDCl_3$): δ 217.6, 157.6 (dd, *J* = 5.0, 252.0 Hz), 130.5 (t, *J* = 10.0 Hz), 124.2 (d, *J* = 19.0 Hz), 111.7 (dd, *J* = 4.6, 21.0 Hz), 20.6 ppm; HRMS [TOF MS ES+]: m/z $[M + H]^+$ calcd. for $C_8H_7F_2S_2$ 204.9957, found 204.9963 (2.9 ppm).

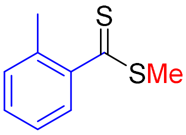

Methyl 2-methylbenzodithioate (**30j**): Following General Procedure B, 2-(*o*-tolyl)-1,3-dithiolane (**1j**, 0.5 mmol, 98 mg), LiHMDS (0.60 mmol, 100 mg), HMPA (0.25

mmol, 44 μL) in 1.0 mL CPME were reacted at 100 for 5 minutes, and then methyl iodide (1.00 mmol, 62 μL) in 1.0 mL CPMA was added into the reaction mixture by a syringe at 100 °C. The resulting mixture was stirred at 100 °C for 30 min. Product **30j** was isolated (77 mg, 84%) as red oil by flash chromatography on silica gel using hexanes as eluent. ^{1}H NMR (500 MHz, $CDCl_3$): δ 7.32–7.21 (m, 4H), 2.78 (m, 3H), 2.38 (s, 3H) ppm; ^{13}C-APT NMR (125 MHz, $CDCl_3$): δ 233.8, 147.4, 133.3, 130.8, 129.2, 126.4, 125.5, 20.7, 19.5 ppm; HRMS [FT MS ESI+]: m/z $[M + H]^+$ calcd. for $C_9H_{11}S_2$ 183.0297, found 183.0295 (−1.1 ppm).

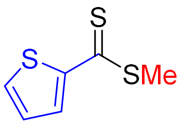

Methyl thiophene-2-carbodithioate (**30k**)[10]: Following General Procedure B, 2-(thiophen-2-yl)-1,3-dithiolane (**1k**, 0.5 mmol, 94 mg), LiHMDS (0.60 mmol, 100 mg), HMPA (0.25 mmol, 44 μL) in 1.0 mL CPME were reacted at 100 for 5 minutes, and then methyl iodide (1.00 mmol, 62 μL) in 1.0 mL CPMA was added into the reaction mixture by a syringe at 100 °C. The resulting mixture was stirred at 100 °C for 30 min. Product **30k** was isolated (69 mg, 79%) as red oil by flash chromatography on silica gel using hexanes as eluent. ^{1}H NMR (500 MHz, $CDCl_3$): δ 7.82 (dd, J = 1.1, 4.0 Hz, 1H), 7.61 (dd, J = 1.1, 5.1 Hz, 1H), 7.11 (dd, J = 4.0, 5.1 Hz, 1H), 2.76 (s, 3H) ppm; ^{13}C-APT NMR (125 MHz, $CDCl_3$): δ 214.9, 151.4, 133.4, 128.4, 126.6, 19.5 ppm; HRMS [FT MS ESI+]: m/z $[M + H]^+$ calcd. for $C_6H_7S_3$ 174.9704, found 174.9703 (−0.6 ppm).

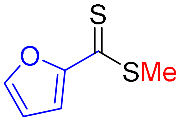

Methyl furan-2-carbodithioate (**30l**)[10]: Following General Procedure B, 2-(1,3-dithiolan-2-yl)furan (**1l**, 0.5 mmol, 86 mg), LiHMDS (0.60 mmol, 100 mg), HMPA (0.25 mmol, 44 μL) in 1.0 mL CPME were reacted at 100 for 5 minutes, and then methyl iodide (1.00 mmol, 62 μL) in 1.0 mL CPMA was added into the reaction mixture by a syringe at 100 °C. The resulting mixture was stirred at 100 °C for 30 min. Product **30l** was isolated (57 mg, 73%) as red oil by flash chromatography on silica gel using hexanes as eluent. ^{1}H NMR (500 MHz, $CDCl_3$): δ 7.63–7.62 (m, 1H), 7.36 (d, J = 3.6 Hz, 1H), 6.51 (dd, J = 1.8, 3.6 Hz, 1H), 2.72 (s, 3H) ppm; ^{13}C-APT NMR (125 MHz, $CDCl_3$): δ 208.1, 157.9, 146.3, 115.1, 113.2, 18.0 ppm; HRMS [FT MS ES+]: m/z $[M + H]^+$ calcd. for $C_6H_7OS_2$ 158.9933, found 158.9932 (−0.6 ppm).

4. General procedure C (for one-pot, two-step reactions of 2-aryl-1,3-dithiolanes with diaryliodonium salts)

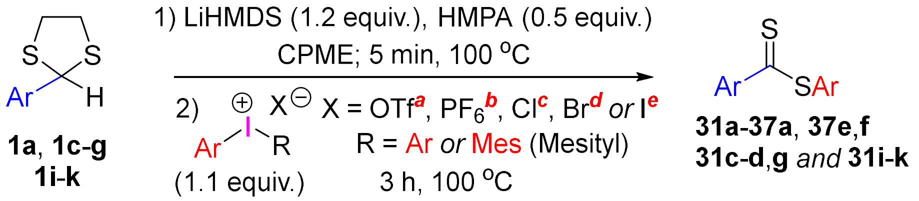

Scheme S4.

An oven-dried 15 mL screw-cap reaction vial equipped with a stirring bar was charged with 2-aryl-1,3-dithiolane derivative (0.5 mmol, 1.0 equiv.) and the vial was brought into a glovebox. The reaction vial was charged with LiHMDS (0.6 mmol, 100 mg, 1.2 equiv.), anhydrous CPME (1.0 mL) and then HMPA (0.5 equiv. 0.25 mmol, 44.0 μL) by a micropipette. The solution's color changed to a deep red after HMPA was added. A 4.0 mL screw-cap vial was charged with diaryliodonium salt (0.55 mmol, 1.1 equiv.) and then both vials were closed with their caps and removed from the glovebox. *Diaryliodonium salts have very low solubility in CPME.* After having stirred the reaction mixture for 5 min. at 100 °C in a pre-heated oil bath, the reaction vial was taken from oil bath and opened to air; then diaryliodonium salt was added into the reaction vial. After the resulting reaction mixture was stirred for 3 h at 100 °C, the reaction vial was cooled to room temperature. The reaction mixture was taken into a 50 mL flask and the solvent was removed in a rotatory evaporator. The remaining residue was dissolved in CH_2Cl_2 and mixed with silica gel (about 0.5 g). After evaporating CH_2Cl_2, the remaining silica gel was directly loaded onto a column and purified by flash chromatography on silica gel using hexanes/ethyl acetate mixture as eluent to yield the product.

4.1. Synthesis of dithioesters 31a-37a, 37e-f, 31c-d, g and 31i-k

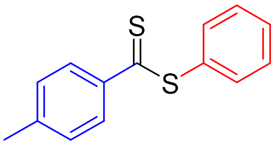

Phenyl 4-methylbenzodithioate (31a)[11]: Following General Procedure C, 2-(*p*-tolyl)-1,3-dithiolane (**1a**, 0.5 mmol, 98 mg), LiHMDS (0.60 mmol, 100 mg), HMPA (0.25 mmol, 44 μL) in 1.0 mL CPME were reacted at 100 for 5 minutes, and then diaryliodonium salt 0.55 mmol ([a]diphenyliodonium triflate, 237 mg; [b]diphenyliodonium hexafluorophosphate, 234 mg; [c]diphenyliodonium chloride, 174 mg;

[e]diphenyliodonium iodide, 224 mg) was added into the reaction mixture. The resulting mixture was stirred at 100 ºC for 3 hours. Product **31a** was isolated ([a]82 mg, 67%; [b]89 mg, 73%; [c]66 mg, 54%; [e]61 mg, 50%) as purple reddish solid by flash chromatography on silica gel using hexanes as eluent. ^{1}H NMR (500 MHz, $CDCl_3$): δ 8.06 (d, *J* = 8.0 Hz, 2H), 7.54–7.49 (m, 5H), 7.24 (d, *J* = 8.0 Hz, 2H), 2.42 (s, 3H) ppm; ^{13}C-APT NMR (125 MHz, $CDCl_3$): δ 227.8, 143.6, 142.0, 135.4, 131.4, 130.2, 129.5, 129.0, 127.0, 21.5 ppm; HRMS [TOF MS ES+]: m/z $[M + H]^+$ calcd. for $C_{14}H_{13}S_2$ 245.0453, found 245.0457 (1.6 ppm).

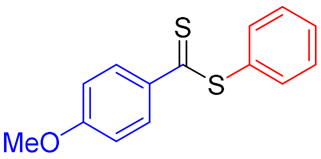

Phenyl 4-methoxybenzodithioate (31c)[11]: Following General Procedure C, 2-(4-methoxyphenyl)-1,3-dithiolane (**1c**, 0.5 mmol, 106 mg), LiHMDS (0.60 mmol, 100 mg), HMPA (0.25 mmol, 44 µL) in 1.0 mL CPME were reacted at 100 for 5 minutes, and then diphenyliodonium triflate (0.55 mmol, 237 mg) was added into the reaction mixture. The resulting mixture was stirred at 100 ºC for 3 hours. Product **31c** was isolated (61 mg, 47%) as red solid by flash chromatography on silica gel using hexanes as eluent. ^{1}H NMR (500 MHz, $CDCl_3$): δ 8.23 (d, *J* = 8.9 Hz, 2H), 7.54–7.50 (m, 5H), 6.94 (d, *J* = 8.9 Hz, 2H), 3.90 (s, 3H) ppm; ^{13}C-APT NMR (125 MHz, $CDCl_3$): δ 225.7, 163.8, 137.5, 135.6, 131.4, 130.1, 129.5, 129.2, 113.5, 55.5 ppm; HRMS [TOF MS ES+]: m/z $[M + H]^+$ calcd. for $C_{14}H_{13}OS_2$ 261.0402, found 261.0408 (2.3 ppm).

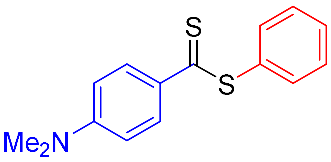

Phenyl 4-(dimethylamino)benzodithioate (31d): Following General Procedure C, 2-(4-dimethylaminophenyl)-1,3-dithiolane (**1d**, 0.5 mmol, 113 mg), LiHMDS (0.60 mmol, 100 mg), HMPA (0.25 mmol, 44 µL) in 1.0 mL CPME were reacted at 100 for 5 minutes, and then diphenyliodonium triflate (0.55 mmol, 237 mg) was added into the reaction mixture. The resulting mixture was stirred at 100 ºC for 3 hours. Product **31d** was isolated (53 mg, 45%) as orange solid by flash chromatography on silica gel using hexanes as eluent. ^{1}H NMR (500 MHz, $CDCl_3$): δ 8.25 (d, *J* = 9.0 Hz, 2H), 7.50 (s, 5H), 6.62 (d, *J* = 9.0 Hz, 2H), 3.09 (s, 6H) ppm; ^{13}C-APT NMR (125 MHz, $CDCl_3$): δ 222.7, 153.9, 135.9, 132.8, 131.8, 129.8, 129.6, 129.2, 110.4, 40.1 ppm; HRMS [TOF MS ES+]: m/z $[M + H]^+$ calcd. for $C_{15}H_{16}NS_2$ 274.0719, found 274.0724 (1.8 ppm).

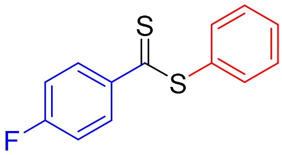

Phenyl 4-fluorobenzodithioate (**31g**): Following General Procedure C, 2-(4-fluorophenyl)-1,3-dithiolane (**1g**, 0.5 mmol, 100 mg), LiHMDS (0.60 mmol, 100 mg), HMPA (0.25 mmol, 44 μL) in 1.0 mL CPME were reacted at 100 for 5 minutes, and then diphenyliodonium triflate (0.55 mmol, 237 mg) was added into the reaction mixture. The resulting mixture was stirred at 100 °C for 3 hours. Product **31g** was isolated (65 mg, 52%) as red solid by flash chromatography on silica gel using hexanes as eluent. ^{1}H NMR (500 MHz, $CDCl_3$): δ 8.19–8.14 (m, 2H), 7.55–7.48 (m, 5H), 7.14–7.09 (m, 2H) ppm; ^{13}C-APT NMR (125 MHz, $CDCl_3$): δ 226.1, 165.7 (d, J = 255.0 Hz), 140.7, (d, J = 3.1 Hz), 135.6, 131.2, 130.4, 129.6, 129.3 (d, J = 9.0 Hz), 115.4 (d, J = 22.0 Hz) ppm; HRMS [TOF MS ES+]: m/z $[M + H]^+$ calcd. for $C_{13}H_{10}FS_2$ 249.0202, found 249.0203 (0.4 ppm).

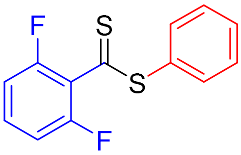

Phenyl 2,6-difluorobenzodithioate (**31i**): Following General Procedure C, 2-(2,6-difluorophenyl)-1,3-dithiolane (**1i**, 0.5 mmol, 109 mg), LiHMDS (0.60 mmol, 100 mg), HMPA (0.25 mmol, 44 μL) in 1.0 mL CPME were reacted at 100 for 5 minutes, and then diphenyliodonium triflate (0.55 mmol, 237 mg) was added into the reaction mixture. The resulting mixture was stirred at 100 °C for 3 hours. Product **31i** was isolated (100 mg, 75%) as orange reddish solid by flash chromatography on silica gel using hexanes as eluent. ^{1}H NMR (500 MHz, $CDCl_3$): δ 7.55–7.47 (m, 5H), 7.33–7.27 (m, 1H), 6.92 (t, J = 8.0 Hz, 2H) ppm; ^{13}C-APT NMR (125 MHz, $CDCl_3$): δ 218.9, 157.3 (dd, J = 5.6, 252.0 Hz), 134.7, 130.8, 130.6 (dd, J = 2.5, 9.9 Hz), 129.6, 123.9 (t, J = 19.6 Hz), 111.7 (dd, J = 4.5, 20.5 Hz) ppm; HRMS [TOF MS ES+]: m/z $[M + H]^+$ calcd. for $C_{13}H_9F_2S_2$ 267.0108, found 267.0110 (0.7 ppm).

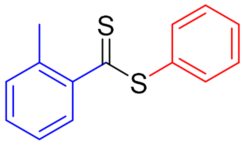

Phenyl 2-methylbenzodithioate (**31j**)[12]: Following General Procedure C, 2-(*o*-tolyl)-1,3-dithiolane (**1j**, 0.5 mmol, 98 mg), LiHMDS (0.60 mmol, 100 mg), HMPA (0.25 mmol, 44 μL) in 1.0 mL CPME were reacted at 100 for 5 minutes, and then diphenyliodonium triflate (0.55 mmol, 237 mg) was added into the reaction mixture. The resulting mixture was stirred at 100 °C for 3 hours. Product **31j** was isolated (42 mg, 34%) as orange solid by flash chromatography on silica gel using hexanes as eluent. ^{1}H NMR (500 MHz, $CDCl_3$): δ 7.53 (bs, 5H), 7.38 (d, J = 7.4 Hz, 1H), 7.33–7.30 (m, 1H), 7.25 (t, J = 7.4 Hz, 2H), 2.47 (s,

3H) ppm; ^{13}C-APT NMR (125 MHz, $CDCl_3$): δ 233.4, 146.7, 134.7, 133.3, 131.3, 130.8, 130.4, 129.7, 129.3, 126.7, 125.6, 19.5 ppm; HRMS [TOF MS ES+]: m/z $[M + H]^+$ calcd. for $C_{14}H_{13}S_2$ 245.0453, found 245.0458 (2.0 ppm).

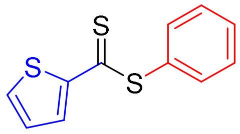

Phenyl thiophene-2-carbodithioate (**31k**)[13]: Following General Procedure C, 2-(thiophen-2-yl)-1,3-dithiolane (**1k**, 0.5 mmol, 94 mg), LiHMDS (0.60 mmol, 100 mg), HMPA (0.25 mmol, 44 µL) in 1.0 mL CPME were reacted at 100 for 5 minutes, and then diphenyliodonium triflate (0.55 mmol, 237 mg) was added into the reaction mixture. The resulting mixture was stirred at 100 °C for 3 hours. Product **31k** was isolated (88 mg, 75%) as red oil by flash chromatography on silica gel using hexanes as eluent. ^{1}H NMR (500 MHz, $CDCl_3$): δ 7.98 (d, J = 3.9 Hz, 1H), 7.68 (d, J = 5.2 Hz, 1H), 7.53–7.51 (m, 5H), 7.16–7.18 (m, 1H) ppm; ^{13}C-APT NMR (125 MHz, $CDCl_3$): δ 213.7, 151.1, 135.7, 135.5, 130.4, 130.3, 129.5, 128.5, 127.0 ppm; HRMS [TOF MS ES+]: m/z $[M + H]^+$ calcd. for $C_{11}H_9S_3$ 236.9861, found 236.9865 (1.7 ppm).

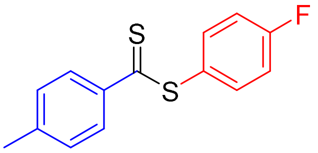

4-Fluorophenyl 4-methylbenzodithioate (**32a**): Following General Procedure C, 2-(*p*-tolyl)-1,3-dithiolane (**1a**, 0.5 mmol, 98 mg), LiHMDS (0.60 mmol, 100 mg), HMPA (0.25 mmol, 44 µL) in 1.0 mL CPME were reacted at 100 for 5 minutes, and then bis(4-fluorophenyl)iodonium trifluoromethanesulfonate (0.55 mmol, 256 mg) was added into the reaction mixture. The resulting mixture was stirred at 100 °C for 3 hours. Product **32a** was isolated (71 mg, 54%) as red solid by flash chromatography on silica gel using hexanes as eluent. ^{1}H NMR (500 MHz, $CDCl_3$): δ 8.06–8.03 (m, 2H), 7.48–7.44 (m, 2H), 7.24–7.18 (m, 4H), 2.42 (s, 3H) ppm; ^{13}C-APT NMR (125 MHz, $CDCl_3$): δ 227.6 (d, J = 1.8 Hz), 164.0 (d, J = 251.5 Hz), 143.8, 141.8, 137.6 (d, J = 8.9 Hz), 129.1, 127.0, 116.9 (d, J = 12.0 Hz), 21.5 ppm; HRMS [TOF MS ES+]: m/z $[M + H]^+$ calcd. for $C_{14}H_{12}FS_2$ 263.0359, found 263.0364 (1.9 ppm).

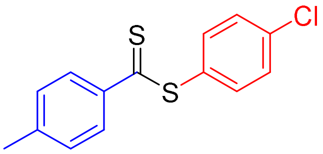

4-Chlorophenyl 4-methylbenzodithioate (**33a**)[12]: Following General Procedure C, 2-(*p*-tolyl)-1,3-dithiolane (**1a**, 0.5 mmol, 98 mg), LiHMDS (0.60 mmol, 100 mg), HMPA (0.25 mmol, 44 µL) in 1.0 mL CPME were reacted at 100 for 5 minutes, and then bis(4-chlorophenyl)iodonium trifluoromethanesulfonate

(0.55 mmol, 275 mg) was added into the reaction mixture. The resulting mixture was stirred at 100 °C for 3 hours. Product **33a** was isolated (80 mg, 60%) as red solid by flash chromatography on silica gel using hexanes as eluent. ^{1}H NMR (500 MHz, $CDCl_3$): δ 8.04 (d, J = 8.0 Hz, 2H), 7.49–7.40 (AB_{system}, δ_A = 7.48, δ_B = 7.41, J_{AB} = 8.5 Hz, 4H), 7.23 (d, J = 8.0 Hz, 2H), 2.41 (s, 3H) ppm; ^{13}C-APT NMR (125 MHz, $CDCl_3$): δ 226.8, 143.9, 141.8, 136.73, 136.70, 129.8, 129.7, 129.1, 127.0, 21.6 ppm; HRMS [TOF MS ES+]: m/z $[M + H]^+$ calcd. for $C_{14}H_{12}S_2Cl$ 279.0063, found 279.0069 (2.2 ppm).

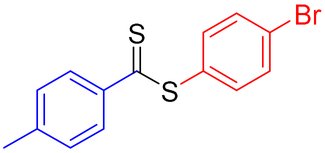

4-Bromophenyl 4-methylbenzodithioate (**34a**): Following General Procedure C, 2-(*p*-tolyl)-1,3-dithiolane (**1a**, 0.5 mmol, 98 mg), LiHMDS (0.60 mmol, 100 mg), HMPA (0.25 mmol, 44 μL) in 1.0 mL CPME were reacted at 100 for 5 minutes, and then bis(4-bromophenyl)iodonium trifluoromethanesulfonate (0.55 mmol, 323 mg) was added into the reaction mixture. The resulting mixture was stirred at 100 °C for 3 hours. Product **34a** was isolated (86 mg, 53%) as pink reddish solid by flash chromatography on silica gel using hexanes as eluent. ^{1}H NMR (500 MHz, $CDCl_3$): δ 8.04 (d, J = 8.0 Hz, 2H), 7.63 (d, J = 8.4 Hz, 2H), 7.34 (d, J = 8.4 Hz, 2H), 7.23 (d, J = 8.0 Hz, 2H), 2.41 (s, 3H) ppm; ^{13}C-APT NMR (125 MHz, $CDCl_3$): δ 226.7, 143.9, 141.8, 137.0, 132.8, 130.4, 129.1, 127.1, 125.1, 21.6 ppm; HRMS [TOF MS ES+]: m/z $[M + H]^+$ calcd. for $C_{14}H_{12}BrS_2$ 322.9558, found 322.9567 (2.8 ppm).

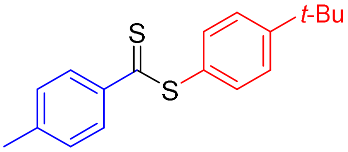

4-(*tert*-Butyl)phenyl 4-methylbenzodithioate (**35a**): Following General Procedure C, 2-(*p*-tolyl)-1,3-dithiolane (**1a**, 0.5 mmol, 98 mg), LiHMDS (0.60 mmol, 100 mg), HMPA (0.25 mmol, 44 μL) in 1.0 mL CPME were reacted at 100 for 5 minutes, and then bis(4-tertbutylphenyl)iodonium trifluoromethanesulfonate (0.55 mmol, 298 mg) was added into the reaction mixture. The resulting mixture was stirred at 100 °C for 3 hours. Product **35a** was isolated (84 mg, 56%) as pink reddish solid by flash chromatography on silica gel using hexanes as eluent. ^{1}H NMR (500 MHz, $CDCl_3$): δ 8.06 (d, J = 8.0 Hz, 2H), 7.54 (d, J = 8.5 Hz, 2H), 7.43 (d, J = 8.5 Hz, 2H), 7.23 (d, J = 8.0 Hz, 2H), 2.42 (s, 3H), 1.40 (s, 9H) ppm; ^{13}C-APT NMR (125 MHz, $CDCl_3$): δ 228.0, 153.5, 143.5, 142.1, 134.9, 129.0, 128.0, 127.0, 126.6, 34.9, 31.2, 21.5 ppm; HRMS [TOF MS ES+]: m/z $[M + H]^+$ calcd. for $C_{18}H_{21}S_2$ 301.1079, found 301.1085 (2.0 ppm).

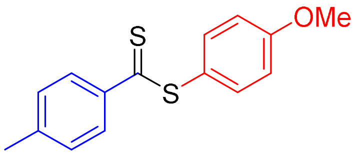

4-Methoxyphenyl 4-methylbenzodithioate (**36a**)[11]: Following General Procedure C, 2-(*p*-tolyl)-1,3-dithiolane (**1a**, 0.5 mmol, 98 mg), LiHMDS (0.60 mmol, 100 mg), HMPA (0.25 mmol, 44 μL) in 1.0 mL CPME were reacted at 100 for 5 minutes, and then bis(4-methoxyphenyl)iodonium bromide (0.55 mmol, 232 mg) was added into the reaction mixture. The resulting mixture was stirred at 100 °C for 3 hours. Product **36a** was isolated (75 mg, 55%) as red solid by flash chromatography on silica gel using hexanes as eluent. ^{1}H NMR (500 MHz, $CDCl_3$): δ 8.04 (d, J = 8.0 Hz, 2H), 7.39 (d, J = 8.7 Hz, 2H), 7.22 (d, J = 8.0 Hz, 2H), 7.03 (d, J = 8.7 Hz, 2H), 3.87 (s, 3H), 2.41 (s, 3H) ppm; ^{13}C-APT NMR (125 MHz, $CDCl_3$): δ 229.0, 161.2, 143.5, 142.0, 136.9, 129.0, 127.0, 122.2, 115.2, 55.3, 21.5 ppm; HRMS [TOF MS ES+]: m/z $[M + H]^+$ calcd. for $C_{15}H_{15}OS_2$ 275.0559, found 275.0565 (2.2 ppm).

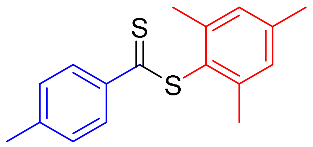

Mesityl 4-methylbenzodithioate (**37a**): Following General Procedure C, 2-(*p*-tolyl)-1,3-dithiolane (**1a**, 0.5 mmol, 98 mg), LiHMDS (0.60 mmol, 100 mg), HMPA (0.25 mmol, 44 μL) in 1.0 mL CPME were reacted at 100 for 5 minutes, and then dimesityliodonium triflate (0.55 mmol, 283 mg) was added into the reaction mixture. The resulting mixture was stirred at 100 °C for 3 hours. Product **37a** was isolated (92 mg, 64%) as purple solid by flash chromatography on silica gel using hexanes as eluent. ^{1}H NMR (500 MHz, $CDCl_3$): δ 8.16 (d, J = 8.3 Hz, 2H), 7.27 (d, J = 8.3 Hz, 2H), 7.11 (s, 2H), 2.46 (s, 3H), 2.41 (s, 3H), 2.38 (s, 6H) ppm; ^{13}C-APT NMR (125 MHz, $CDCl_3$): δ 225.4, 143.3, 142.8, 142.1, 140.7, 129.4, 128.9, 127.1, 127.0, 21.5, 21.3, 20.1 ppm; HRMS [TOF MS ES+]: m/z $[M + H]^+$ calcd. for $C_{17}H_{19}S_2$ 287.0923, found 287.0923 (0.0 ppm).

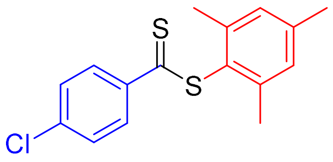

Mesityl 4-chlorobenzodithioate (**37e**): Following General Procedure C, 2-(4-chlorophenyl)-1,3-dithiolane (**1e**, 0.5 mmol, 108 mg), LiHMDS (0.60 mmol, 100 mg), HMPA (0.25 mmol, 44 μL) in 1.0 mL CPME were reacted at 100 for 5 minutes, and then dimesityliodonium triflate (0.55 mmol, 283 mg) was added into the reaction mixture. The resulting mixture was stirred at 100 °C for 3 hours. Product **37e** was isolated (117 mg, 76%) as red solid by flash chromatography on silica gel using hexanes as eluent. ^{1}H NMR (500 MHz, $CDCl_3$): δ 8.16 (d, J = 8.6 Hz, 2H), 7.43 (d, J = 8.6 Hz, 2H), 7.11 (s, 2H),

2.41 (s, 3H), 2.37 (s, 6H) ppm; ^{13}C-APT NMR (125 MHz, $CDCl_3$): δ 223.9, 142.7, 142.6, 140.9, 138.9, 129.5, 128.4, 128.2, 126.7, 21.3, 20.7 ppm; HRMS [TOF MS ES+]: m/z $[M + H]^+$ calcd. for $C_{16}H_{16}S_2Cl$ 307.0376, found 307.0382 (2.0 ppm).

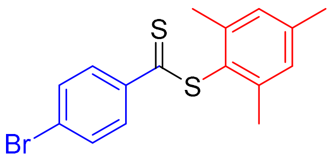

Mesityl 4-bromobenzodithioate (**37f**): Following General Procedure C, 2-(4-bromophenyl)-1,3-dithiolane (**1f**, 0.5 mmol, 131 mg), LiHMDS (0.60 mmol, 100 mg), HMPA (0.25 mmol, 44 μL) in 1.0 mL CPME were reacted at 100 for 5 minutes, and then dimesityliodonium triflate (0.55 mmol, 283 mg) was added into the reaction mixture. The resulting mixture was stirred at 100 °C for 3 hours. Product **37f** was isolated (146 mg, 83%) as red solid by flash chromatography on silica gel using hexanes as eluent. ^{1}H NMR (500 MHz, $CDCl_3$): δ 8.07 (d, *J* = 8.6 Hz, 2H), 7.58 (d, *J* = 8.6 Hz, 2H), 7.10 (s, 2H), 2.40 (s, 3H), 2.36 (s, 6H) ppm; ^{13}C-APT NMR (125 MHz, $CDCl_3$): δ 224.1, 143.0, 142.7, 141.0, 131.4, 129.5, 128.4, 127.5, 126.7, 21.3, 20.8 ppm; HRMS [TOF MS ES+]: m/z $[M + H]^+$ calcd. for $C_{16}H_{16}S_2Br$ 350.9877, found 350.9878 (0.3 ppm).

5. Gram scale synthesis of ethyl 4-methylbenzodithioate (2a)

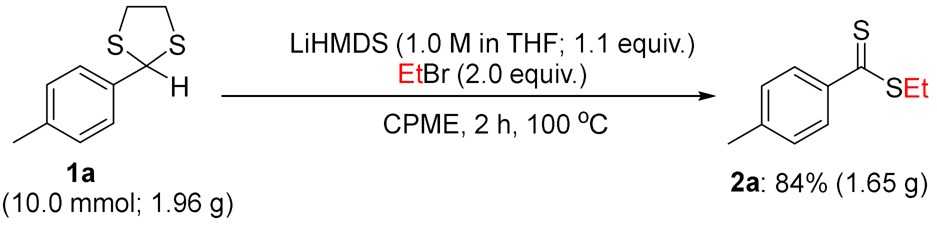

Scheme S5.

An oven-dried 250 mL Schlenk flask equipped with a stirring bar was charged with 2-(*p*-tolyl)-1,3-dithiolane (10.0 mmol, 1.96 g). The open neck was closed with a rubber septum and sealed with a strip of Parafilm. The flask was purged with a stream of nitrogen gas for five minutes. Anhydrous CPME (15 mL), LiHMDS (11.0 mmol, 11.0 mL of a 1.0 M of THF solution) and then ethyl bromide (20 mmol, 1.48 mL) were added by syringe under a nitrogen atmosphere. The flask was removed from the Schlenk line and the reaction mixture was stirred under a nitrogen atmosphere at 100 °C for 2 h. After

cooling the Schlenk flask to room temperature, the reaction mixture was taken into a 250 mL flask and the solvent was removed in a rotatory evaporator. The remaining residue was dissolved in CH_2Cl_2 and mixed with silica gel (about 5.0 g). After evaporating CH_2Cl_2, the remaining silica gel was directly loaded onto a column and purified by flash chromatography on silica gel using hexanes mixture as eluent to yield the product **2a** (1.65 g, 84%) as a red oil.

6. Gram scale synthesis of benzyl 4-methylbenzodithioate (5a) and methyl 4-methylbenzodithioate (31a)

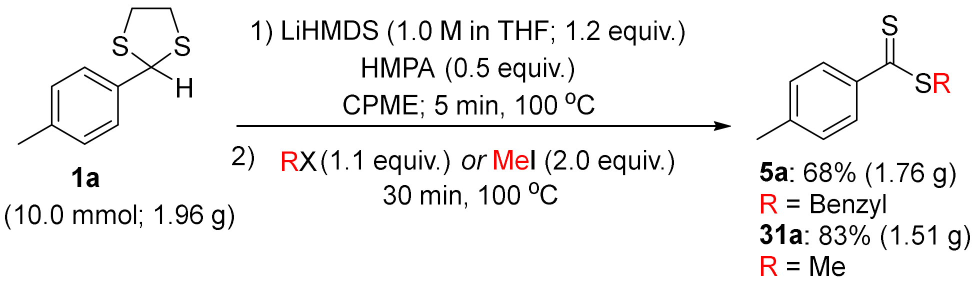

Scheme S6

An oven-dried 250 mL Schlenk flask equipped with a stirring bar was charged with 2-(*p*-tolyl)-1,3-dithiolane (10.0 mmol, 1.96 g). The open neck was closed with a rubber septum and sealed with a strip of Parafilm. The flask was purged with a stream of nitrogen gas for five minutes. Anhydrous CPME (10 mL), LiHMDS (11.0 mmol, 11.0 mL of a 1.0 M of THF solution) and then HMPA (5.0 mmol, 0.87 mL) were added by syringe under a nitrogen atmosphere. The flask was removed from the Schlenk line. After having stirred the resulting deep red reaction mixture for 5 min. at 100 °C, the solution (10.0 mL CPME) of alkyl halide (benzyl bromide: 11.0 mmol, 1.306 mL; methyl iodide: 20.0 mmol, 1.245 mL) was added into the reaction mixture by a syringe at 100 °C. After the reaction mixture was stirred for 30 min. at 100 °C, the Schlenk flask was cooled to room temperature. The reaction mixture was taken into a 250 mL flask and the solvent was removed in a rotatory evaporator. The remaining residue was dissolved in CH_2Cl_2 and mixed with silica gel (about 5.0 g). After evaporating CH_2Cl_2, the remaining silica gel was directly loaded onto a column and purified by flash

chromatography on silica gel using hexanes mixture as eluent to yield the product [**5a** (1.76 g, 68%); **31a** (1.51g, 83%)] as a red oil.

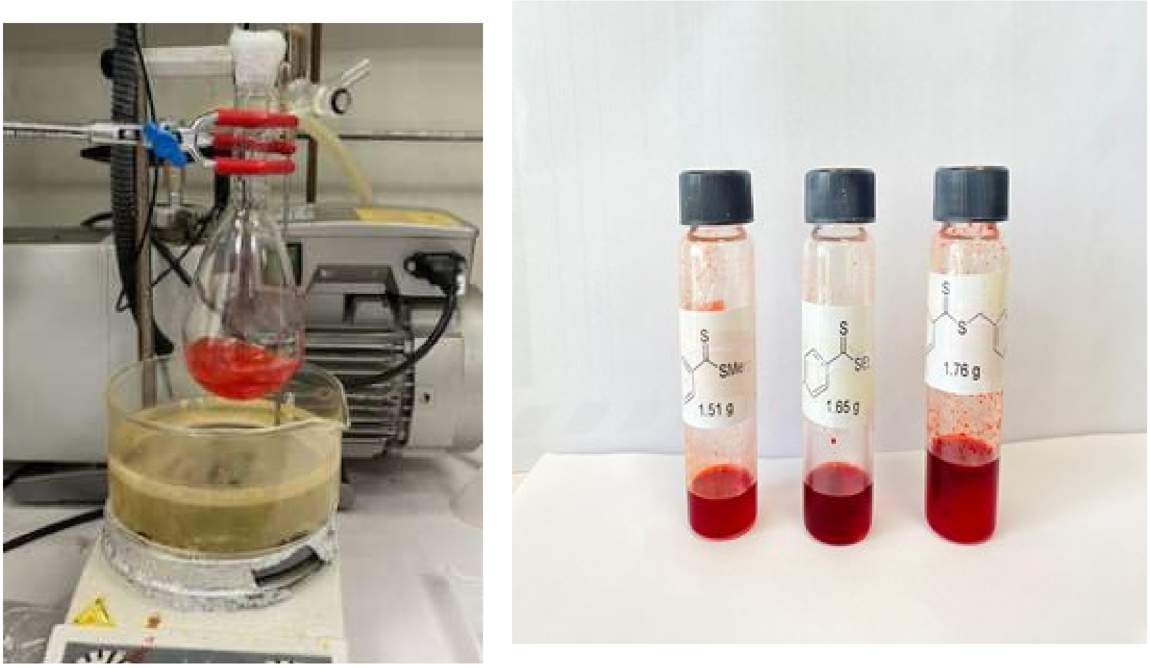

Figure S1.

7. Synthesis of 2-bromoethyl 3-(4,5-diphenyloxazol-2-yl)propanoate

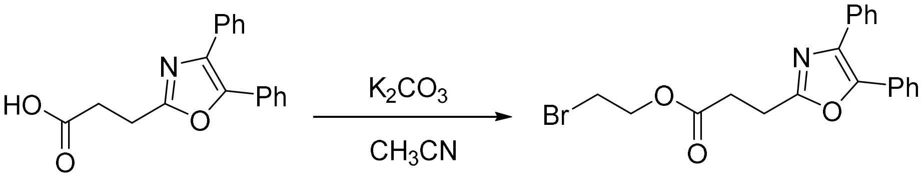

2-Bromoethyl 3-(4,5-diphenyloxazol-2-yl)propanoate was synthesized according to a reported method.[14] To a mixture of oxaprozin (1.0 g, 3.4 mmol) and K_2CO_3 (0.94 g, 6.8 mmol) in CH_3CN, 1,2-dibromoethane (1.46 mL, 17.0 mmol) was added. The resulting mixture was stirred overnight at 80 °C. After cooling to room temperature, the white precipitate was filtered off and the solvent was removed in a rotatory evaporator. The remaining residue was dissolved in CH_2Cl_2 and mixed with silica gel (about 1.0 g). After evaporating CH_2Cl_2, the remaining silica gel was directly loaded onto a column and purified by flash chromatography on silica gel using 20:1 hexanes/ethyl acetate as eluent to isolate product (0.56 g, 41% yield). ^1H NMR (500 MHz, $CDCl_3$): δ 7.63 (d, J = 6.8 Hz, 2H), 7.57 (d, J = 8.7

Hz, 2H), 7.37–7.31 (m, 6H), 4.41 (t, J = 6.1 Hz, 2H), 3.51 (t, J = 6.1 Hz, 2H), 3.21 (t, J = 7.5 Hz, 2H), 2.97 (t, J = 7.5 Hz, 2H) ppm; ^{13}C-APT NMR (125 MHz, $CDCl_3$): δ 171.5, 161.5, 145.5, 135.1, 132.4, 128.9, 128.6, 128.5, 128.4, 128.1, 127.9, 126.5, 64.0, 30.9, 28.5, 23.4 ppm; HRMS [FT MS ES+]: m/z $[M + H]^+$ calcd. for $C_{20}H_{19}BrNO_3$ 400.0548, found 400.0540 (−2.0 ppm).

8. References

1) Kato, S.; Goto, M.; Hattori, R.; Nishiwaki, K.; Mizuta, M.; Ishida, M. *Chem. Ber*. **1985**, *118*, 1668 - 1683.

2) Gonzalo-Barquero, A.; Lepoittevin, B.; Rouden, J.; Baudoux, J. *Molecules* **2023**, *28*, 7333.

3) Ishida, M.; Kaga, K.; Sato, H.; Yokoi, M.; Kato, S. *Bull. Chem. Soc. Jpn.* **1986**, *59*, 1403-1410.

4) Cohen, O.; Mishani, E.; Rozen, S. *Tetrahedron* **2010**, *66*, 3579-3582.

5) Vigante, B. A.; Ozols, Y. Y; Terekhova, M. I.; Petrov, E. S.; Dubur, G. Ya. *J. Heterocycl. Chem.* **1986**, *22*, 401-410.

6) Sudalai, A.; Kanagasabapathy, S.; Benicewicz, B. C. *Org. Lett*. **2000**, *2*, 3213 – 3216.

7) Kanagasabapathy, S.; Sudalai, A.; Benicewicz, B. C. *Tetrahedron Lett*. **2001**, *42*, 3791 – 3794.

8) Söderström, M.; Håkansson, E. O.; Odell, L. R. *Chem. Commun*. **2025**, *61*, 145-148.

9) Olah, G. A.; Bruce, M. R.; Clouet, F. L. *J. Org. Chem*. **1981**, *46*, 438-442.

10) Verkruijsse, H. D.; Brandsma, L. *J. Organomet. Chem*. **1987**, *332*, 95-98.

11) Hazra, G.; Masarwa, A. *Org. Lett*. **2023**, *25*, 6396 –6400.

12) Chen, Z-C.; Jin, Y-Y.; Yang, R-Y. *Synthesis*, **1988**, 723 – 724.

13) Fuchibe, K.; Mukohara, I.; Yamada, A.; Miyazaki, D.; Takayama, R.; Ichikawa, J. *Org. Lett*. **2022**, *24*, 169–174.

14) Falk, E.; Franchino, A.; Horak, T.; Gürtler, L.; Morandi, B. *Organic Lett*. **2023**, *25*, 1695-1700.

Figure S1. ^{1}H NMR (500 MHz, $CDCl_3$) spectrum for **2a**

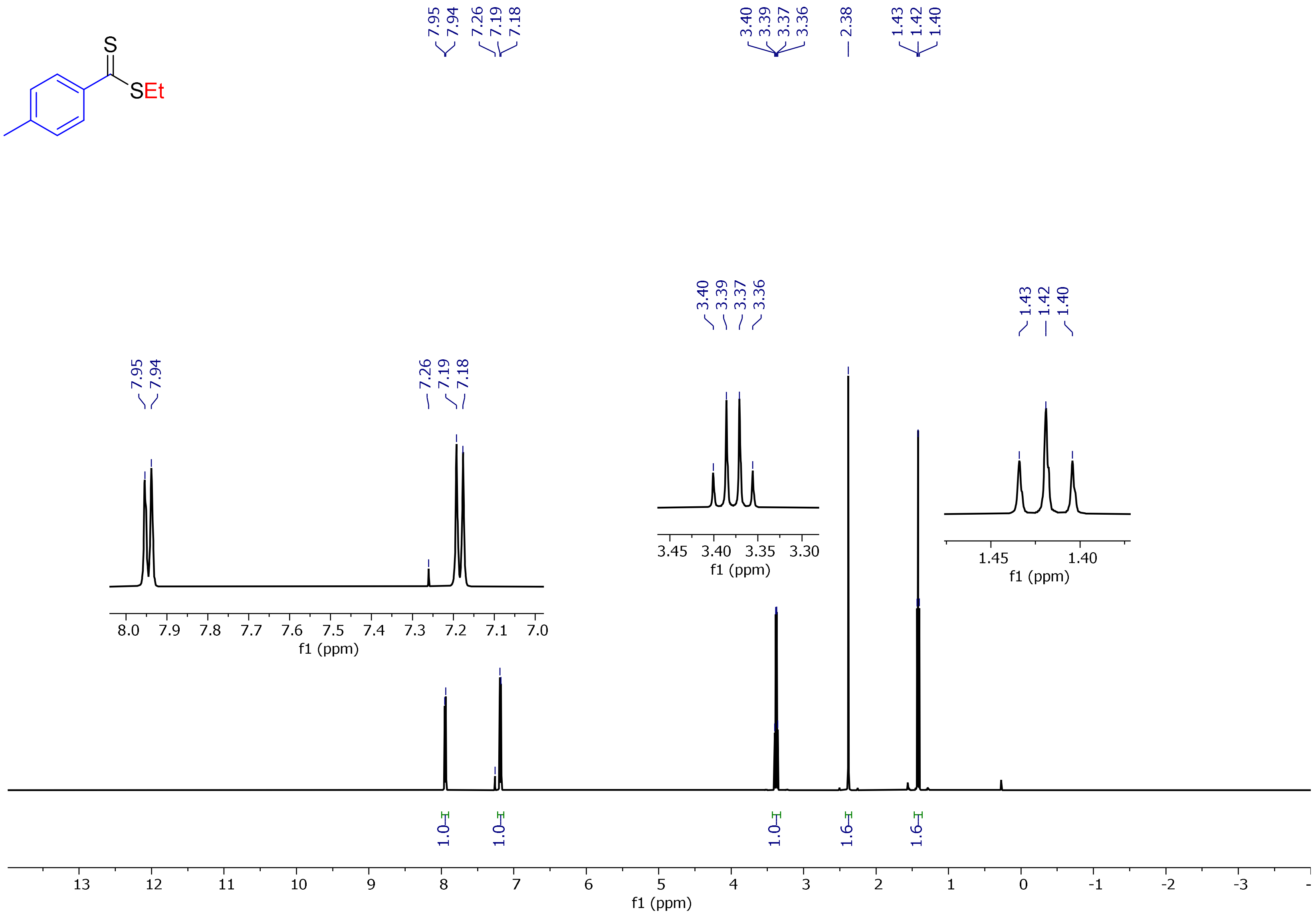

Figure S2. ^{13}C NMR (125 MHz, $CDCl_3$) spectrum for **2a**

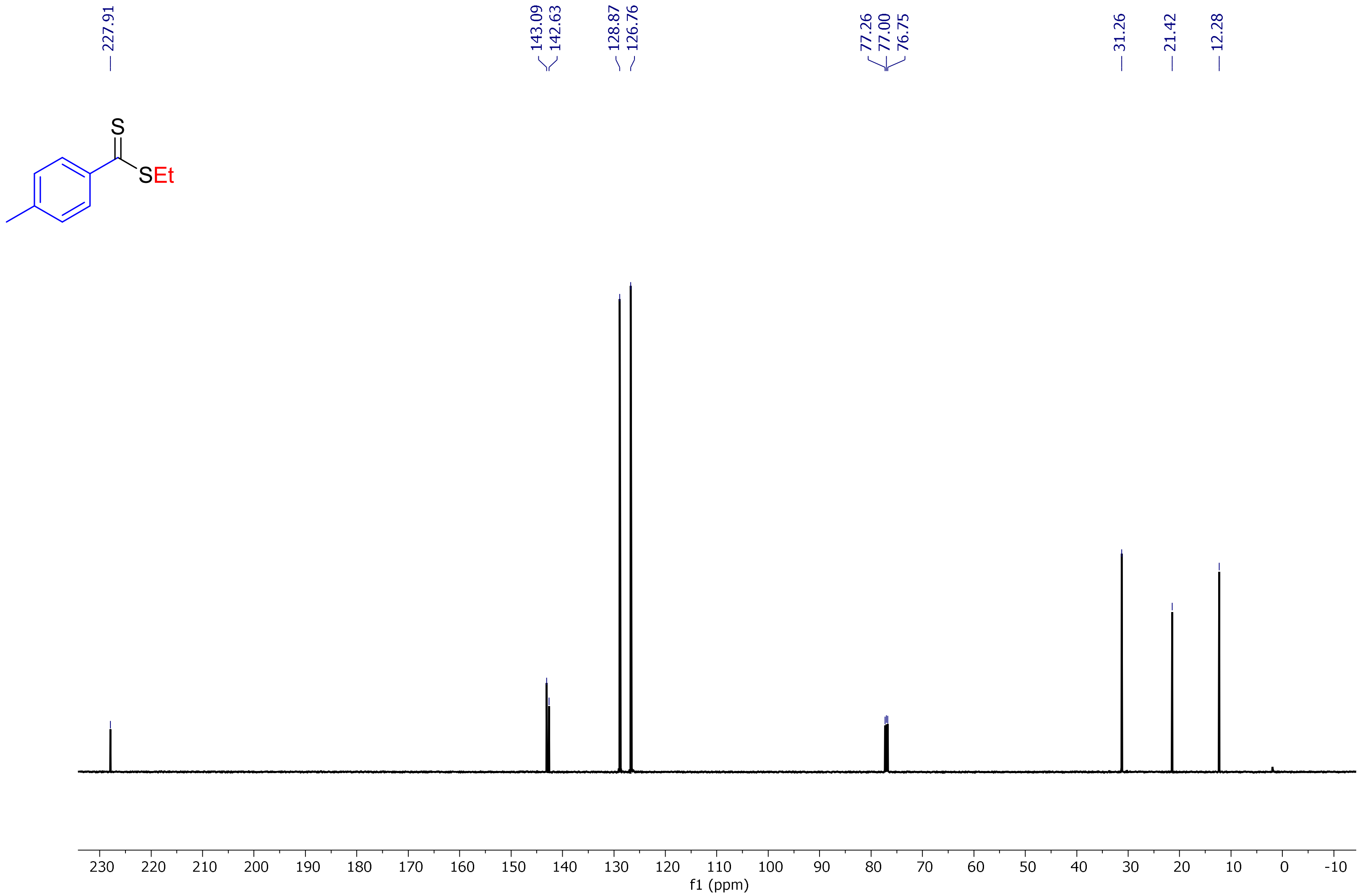

Figure S3. ^{1}H NMR (500 MHz, $CDCl_3$) spectrum for **2b**

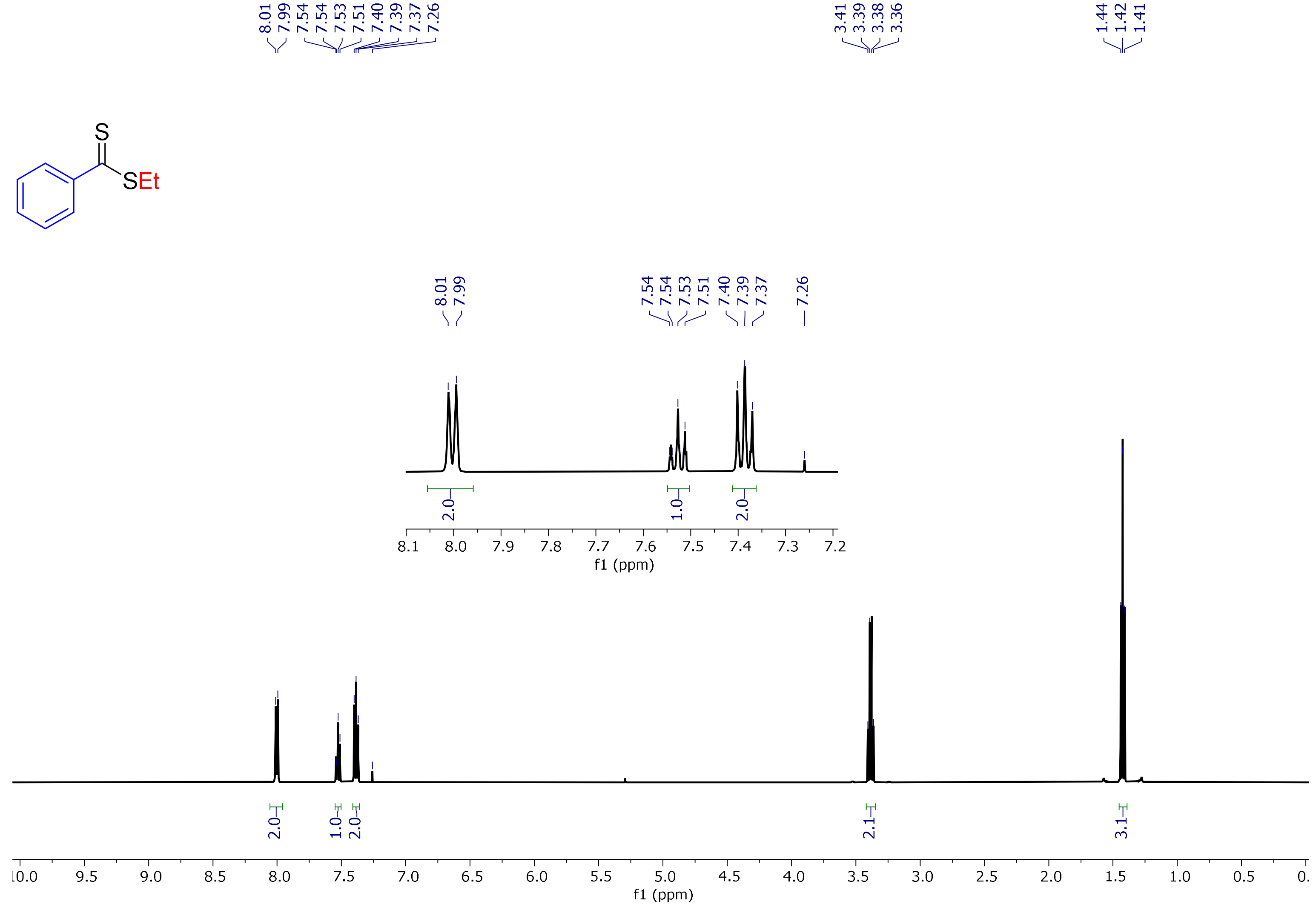

Figure S4. ^{13}C NMR (125 MHz, $CDCl_3$) spectrum for **2b**

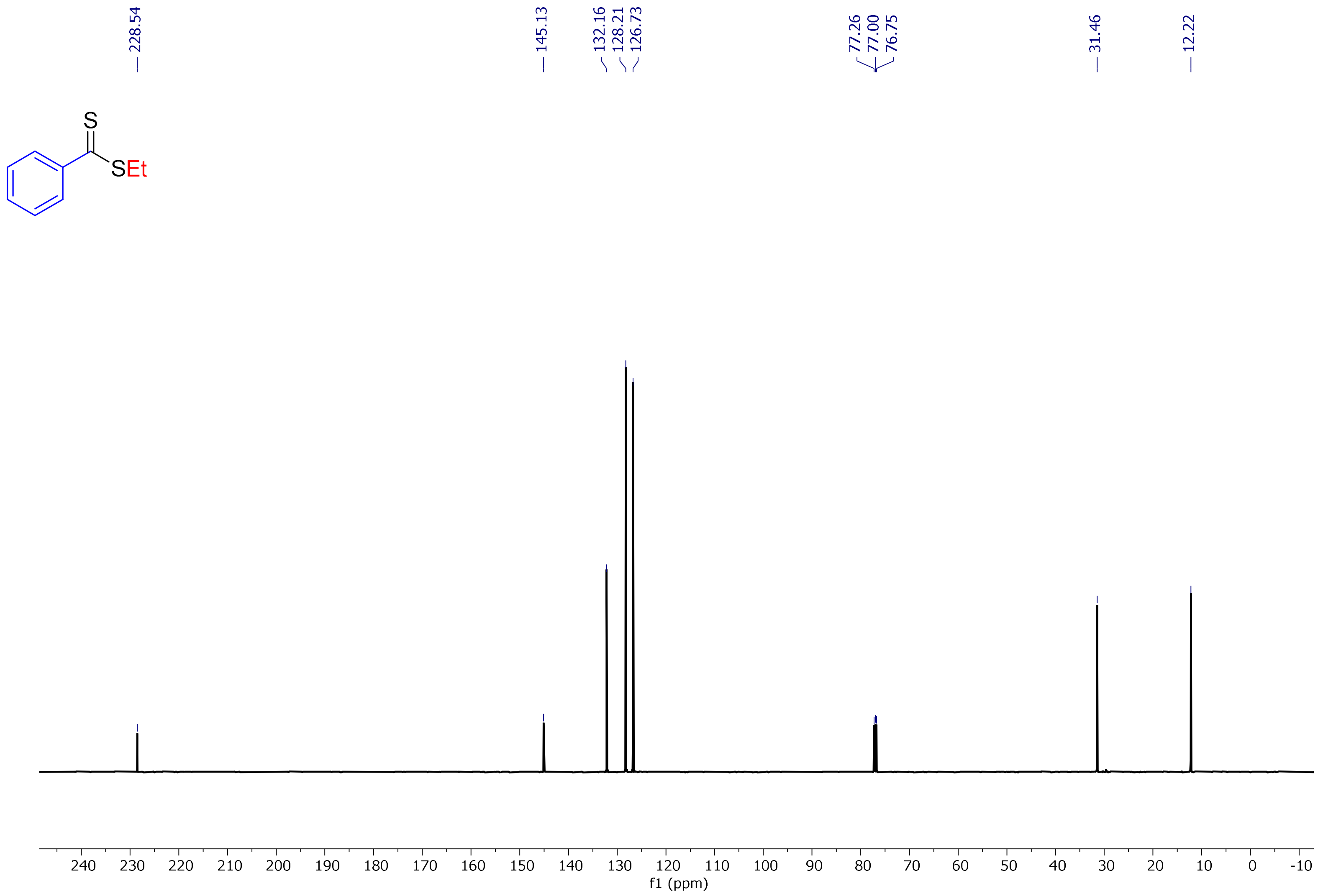

Figure S5. ^{1}H NMR (500 MHz, $CDCl_3$) spectrum for **2c**

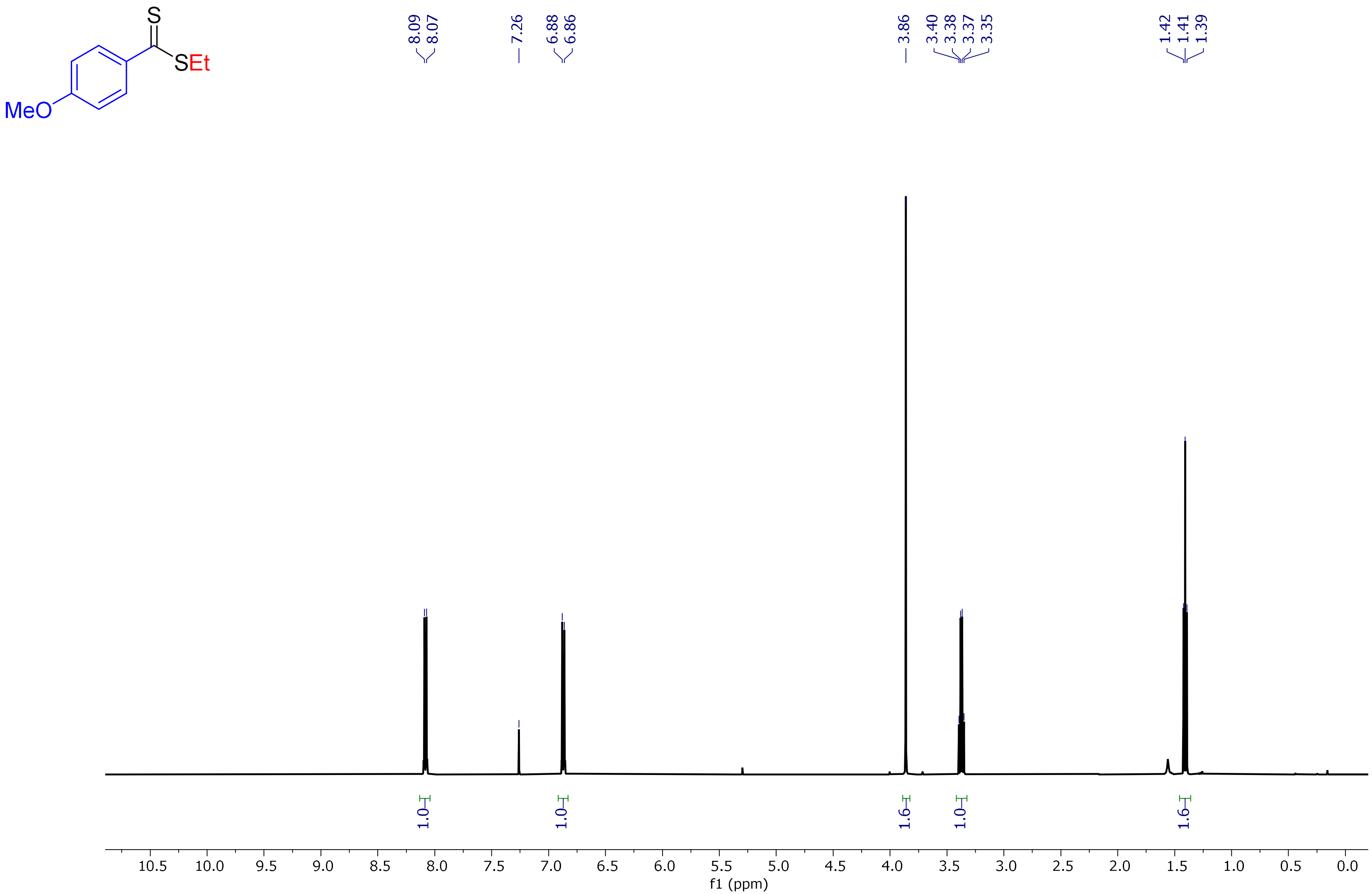

Figure S6. ^{13}C NMR (125 MHz, $CDCl_3$) spectrum for **2c**

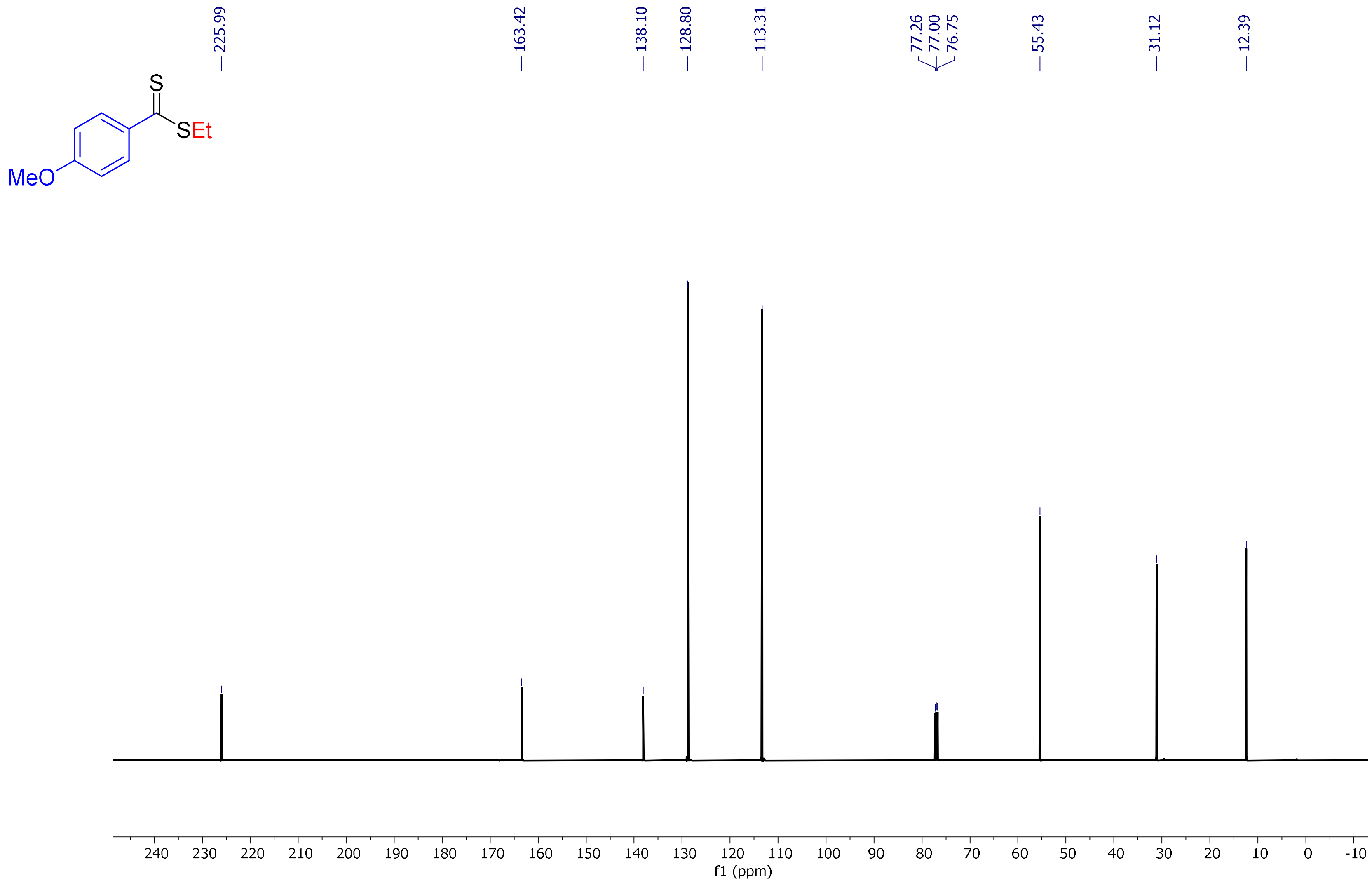

Figure S7. ^{1}H NMR (500 MHz, $CDCl_3$) spectrum for **2d**

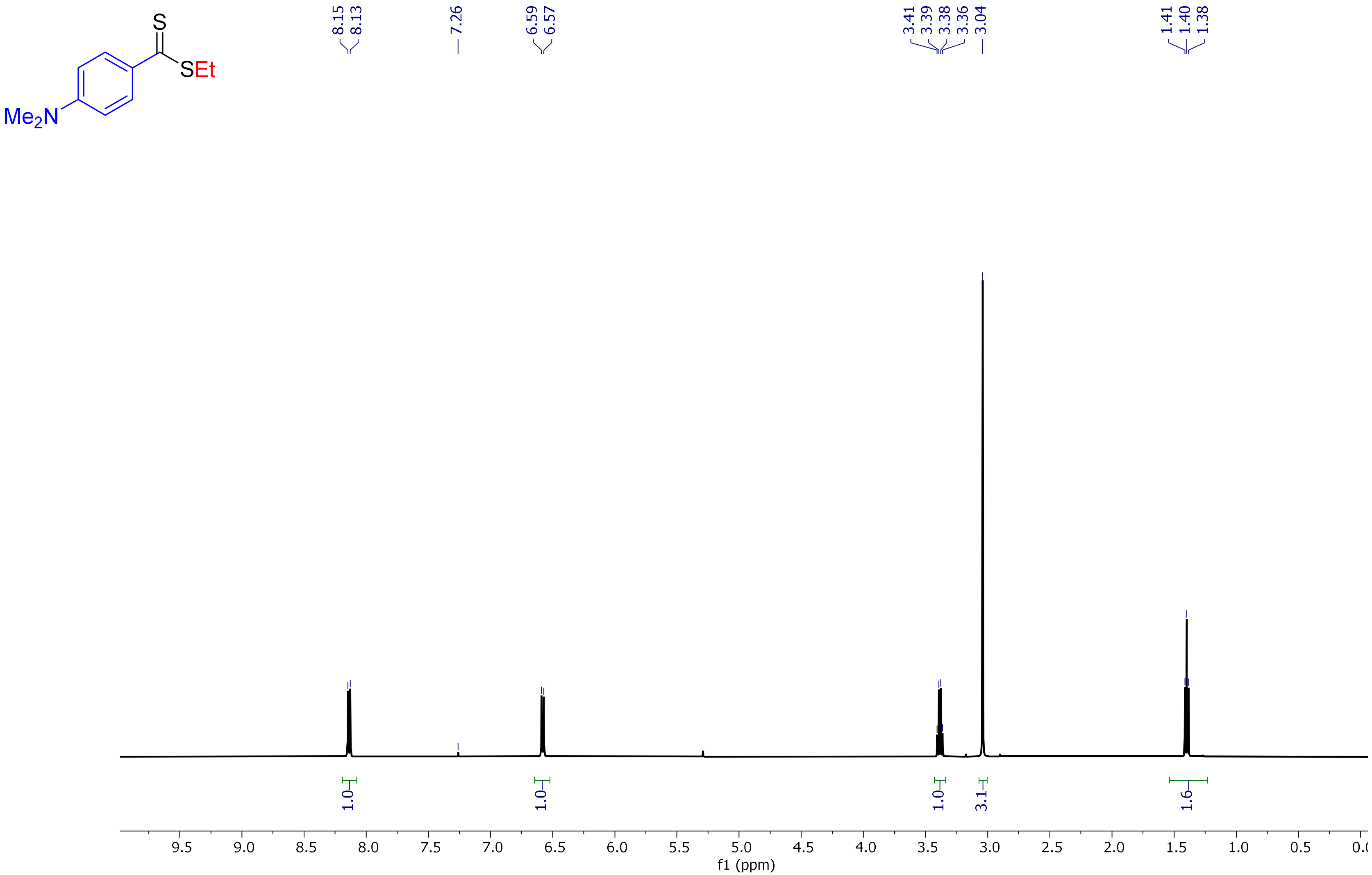

Figure S8. ^{13}C NMR (125 MHz, $CDCl_3$) spectrum for **2d**

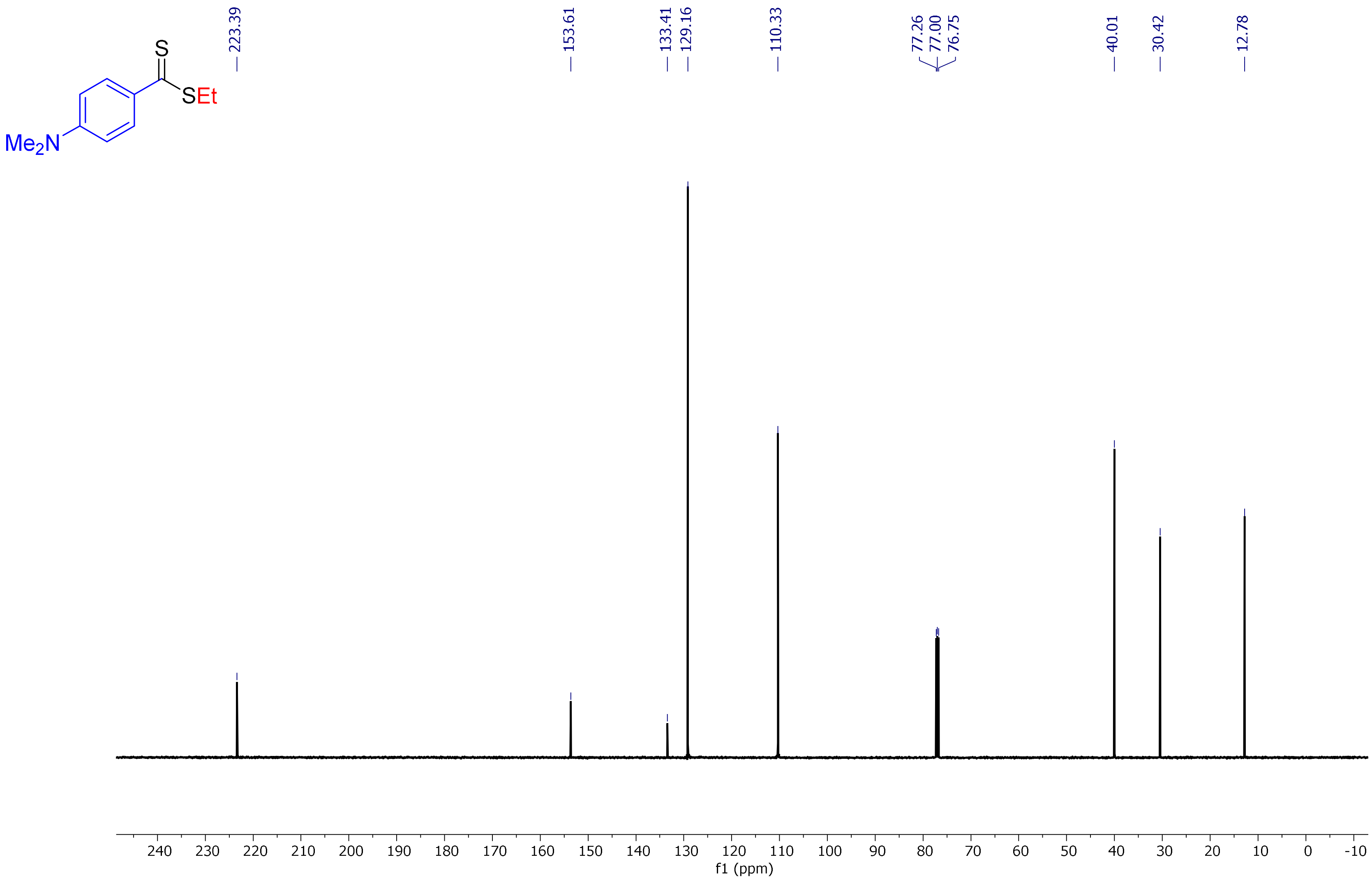

Figure S9. ^{1}H NMR (500 MHz, $CDCl_3$) spectrum for **2e**

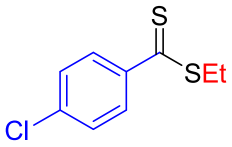

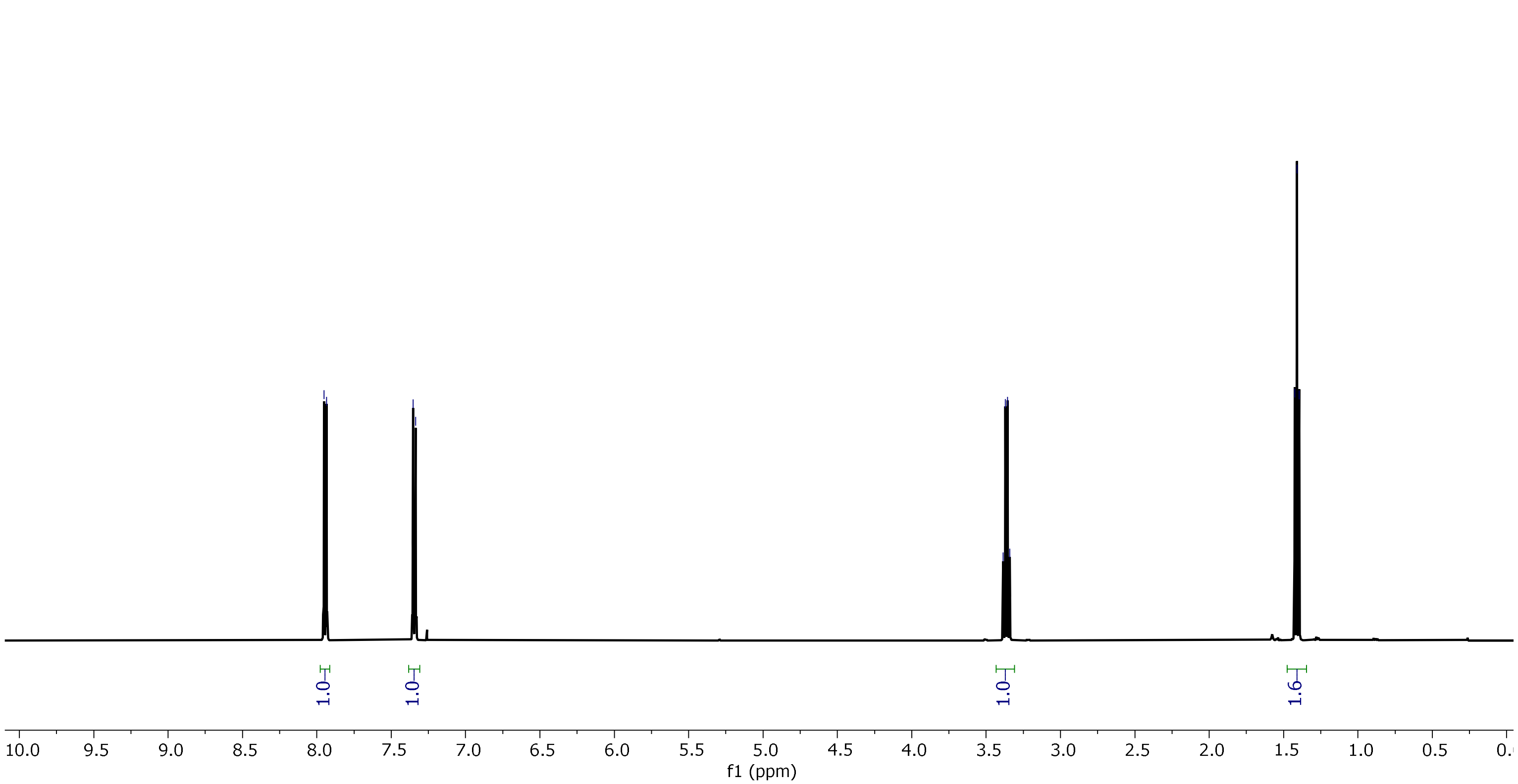

Figure S10. ^{13}C NMR (125 MHz, $CDCl_3$) spectrum for **2e**

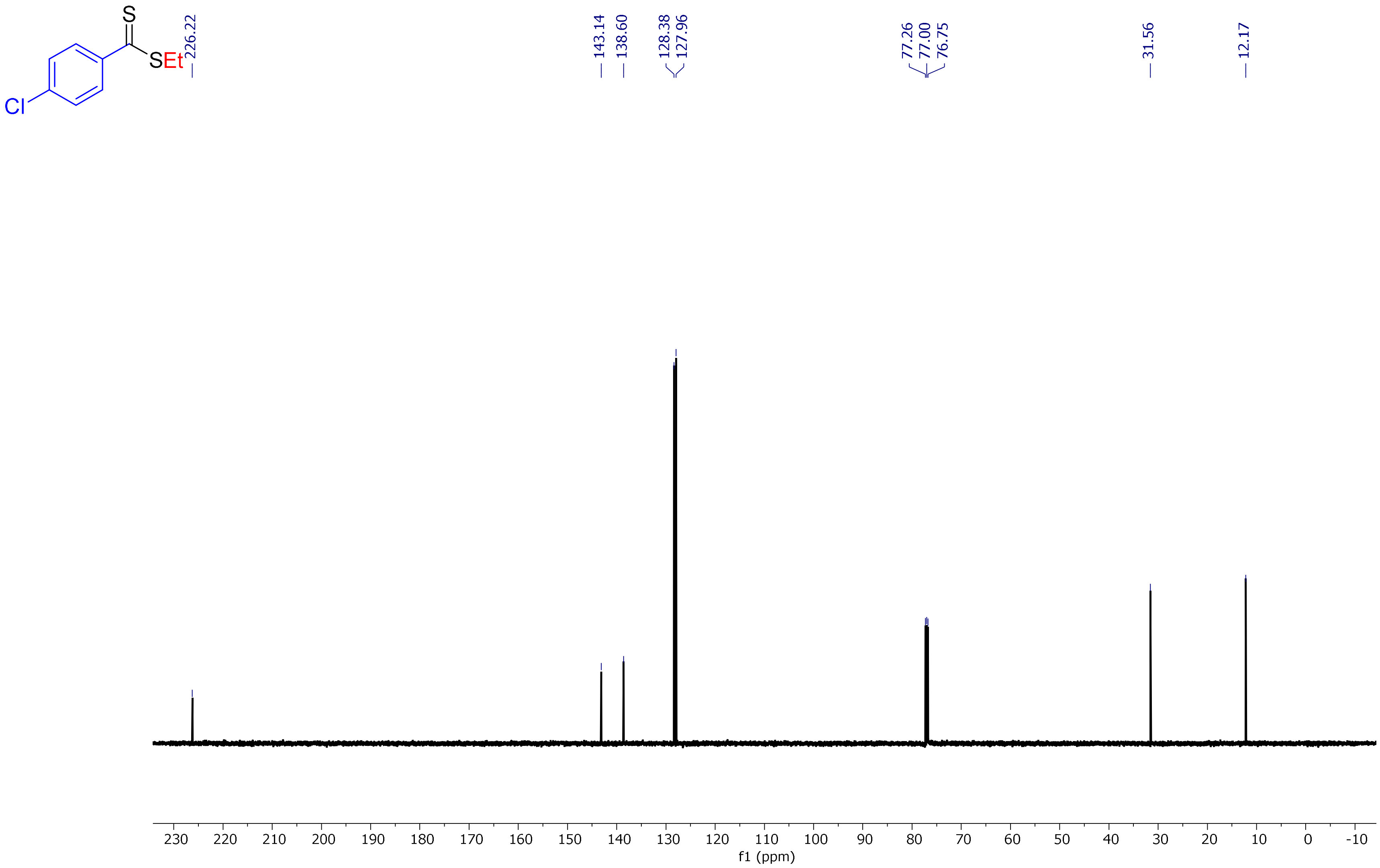

Figure S11. ^{1}H NMR (500 MHz, $CDCl_3$) spectrum for **2f**

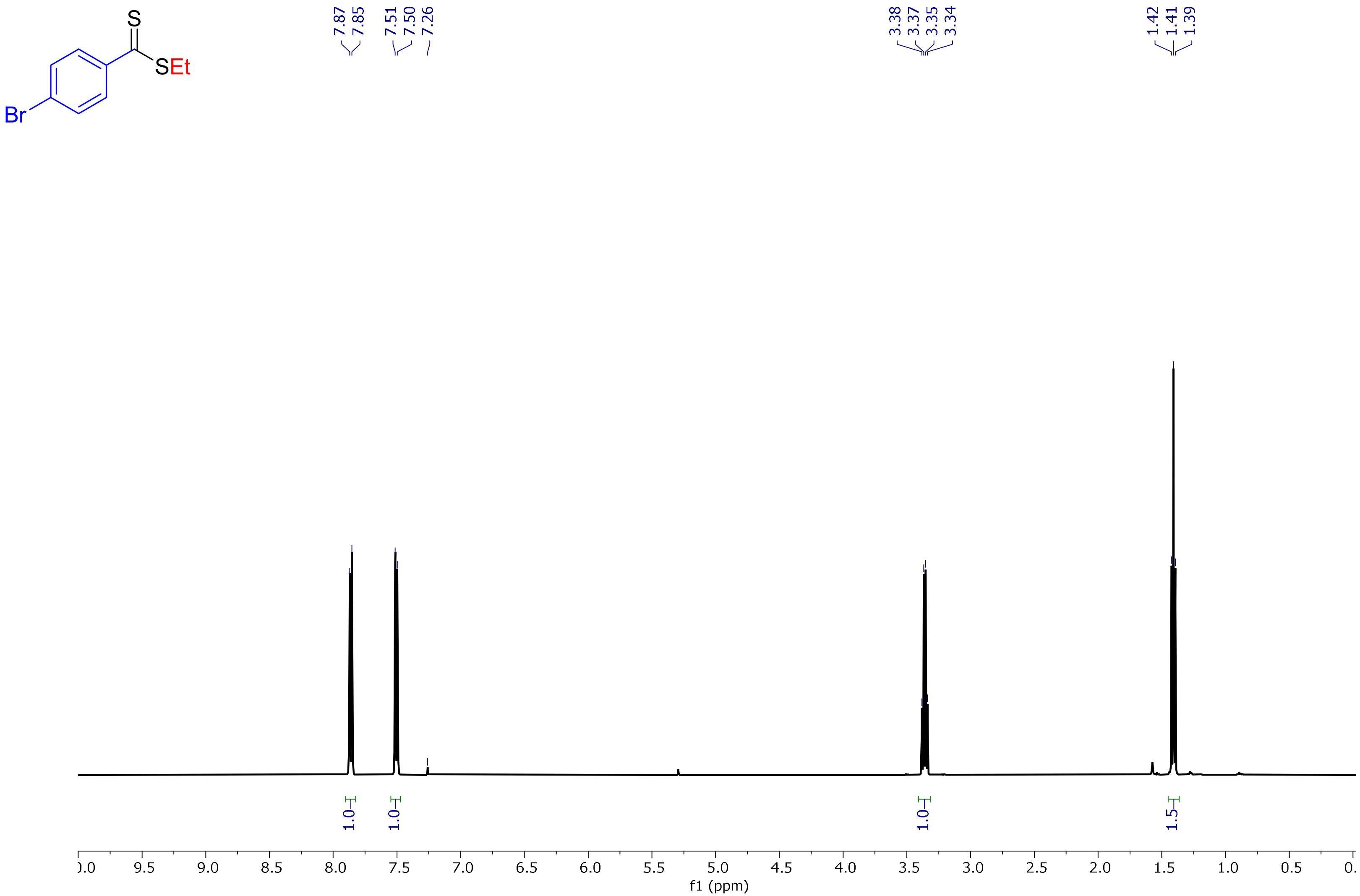

Figure S12. ^{13}C NMR (125 MHz, $CDCl_3$) spectrum for **2f**

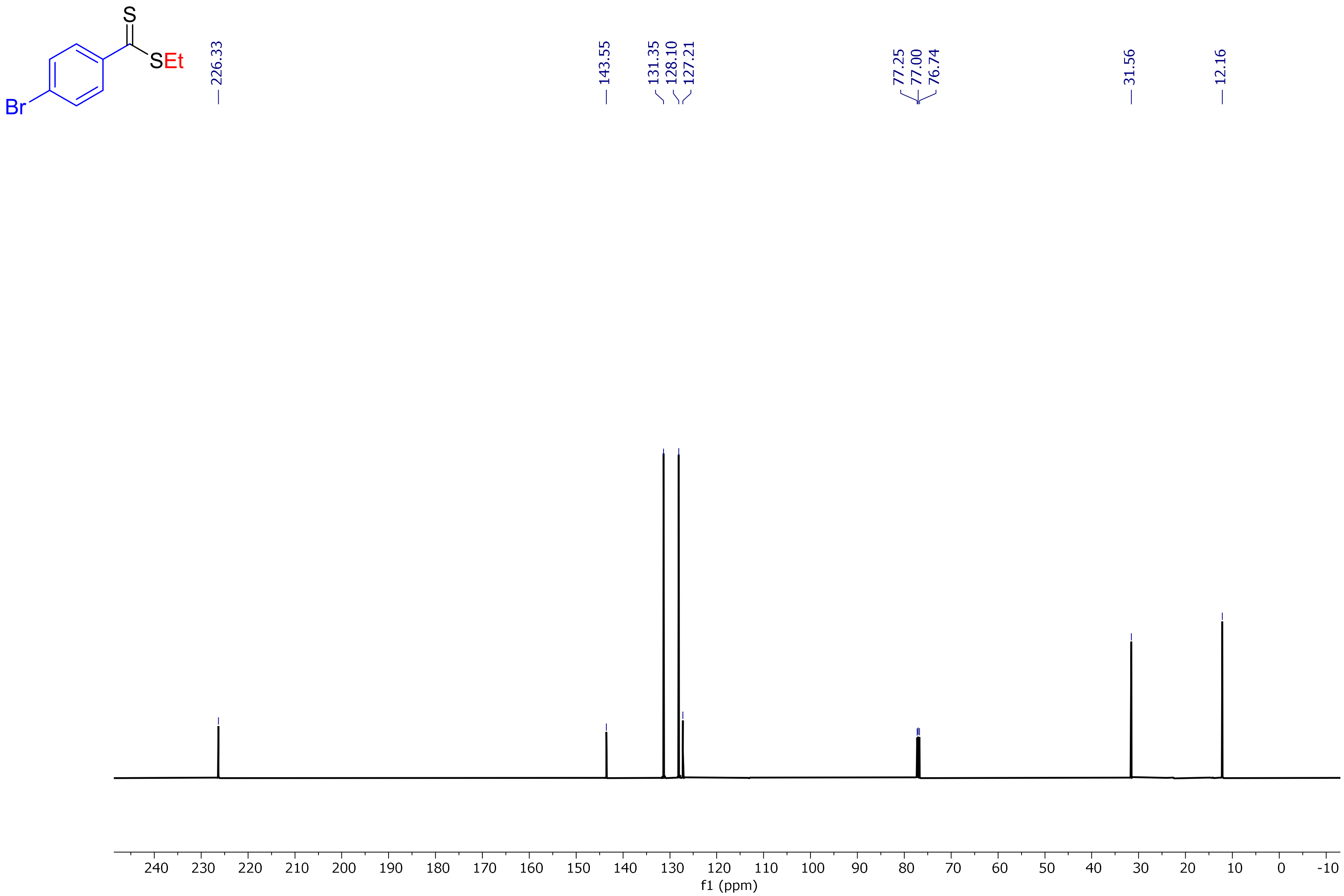

Figure S13. ^{1}H NMR (500 MHz, $CDCl_3$) spectrum for **2g**

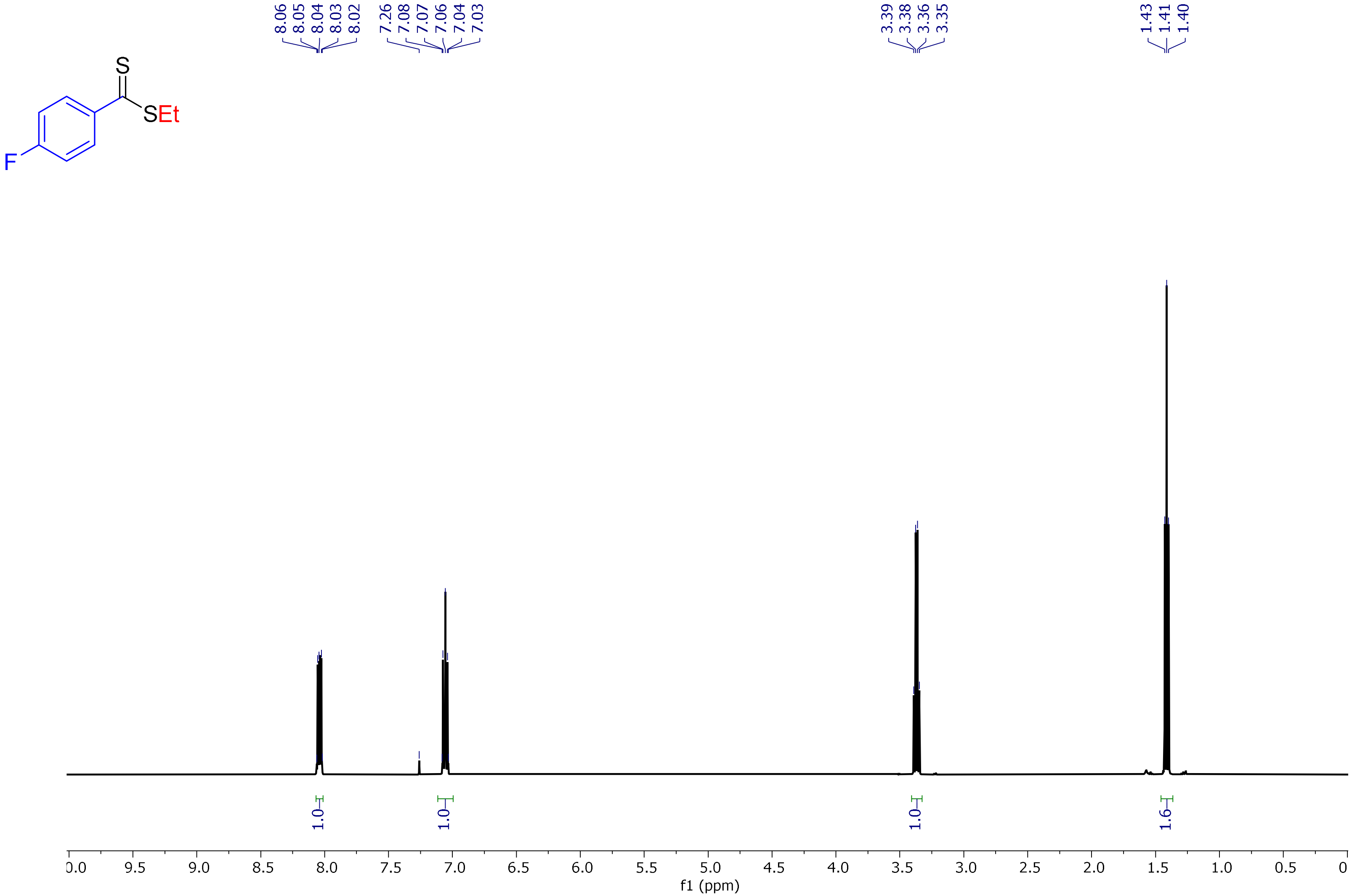

Figure S14. ^{13}C NMR (125 MHz, $CDCl_3$) spectrum for **2g**

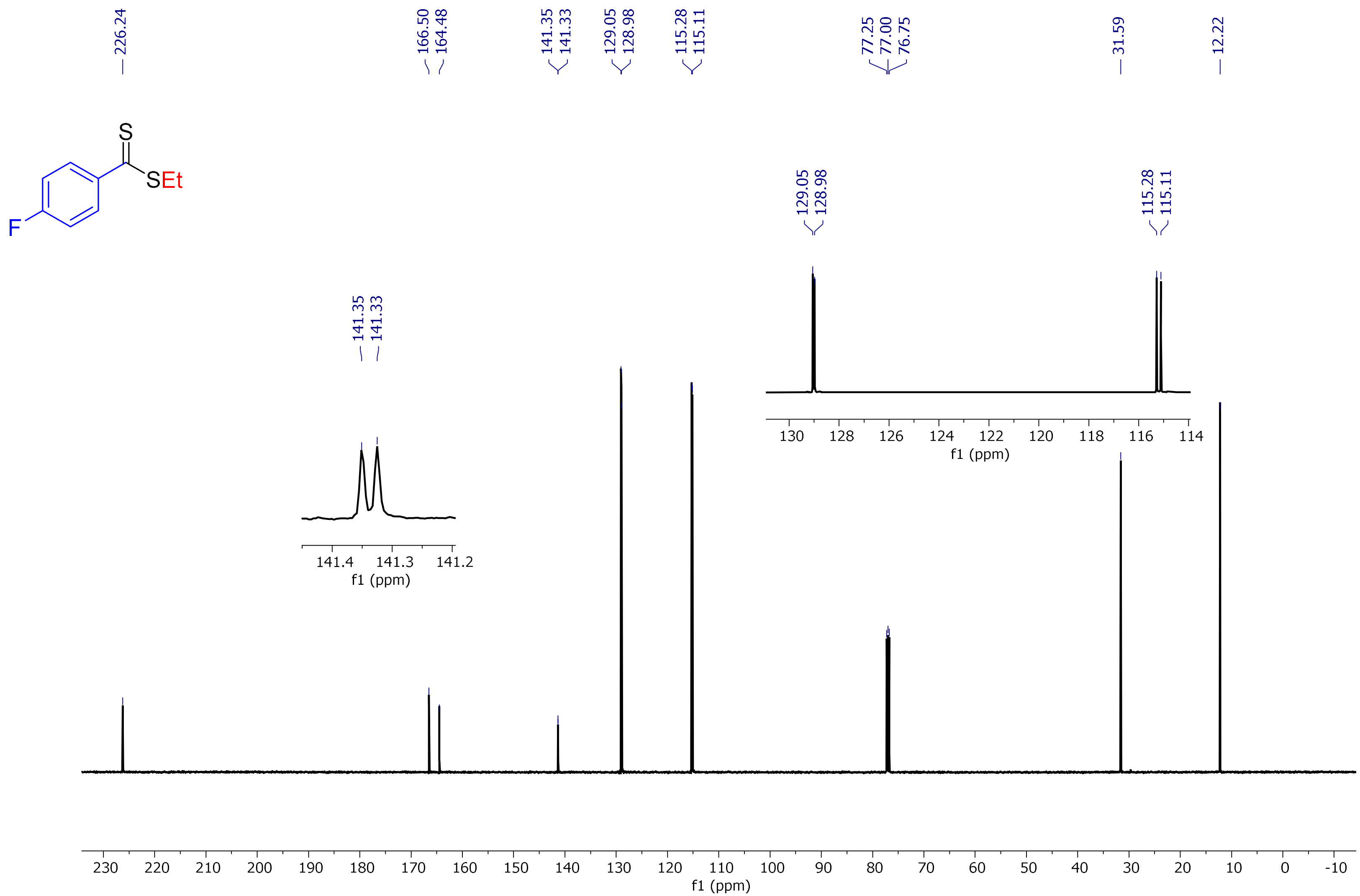

Figure S15. ^{1}H NMR (500 MHz, $CDCl_3$) spectrum for **2h**

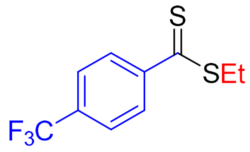

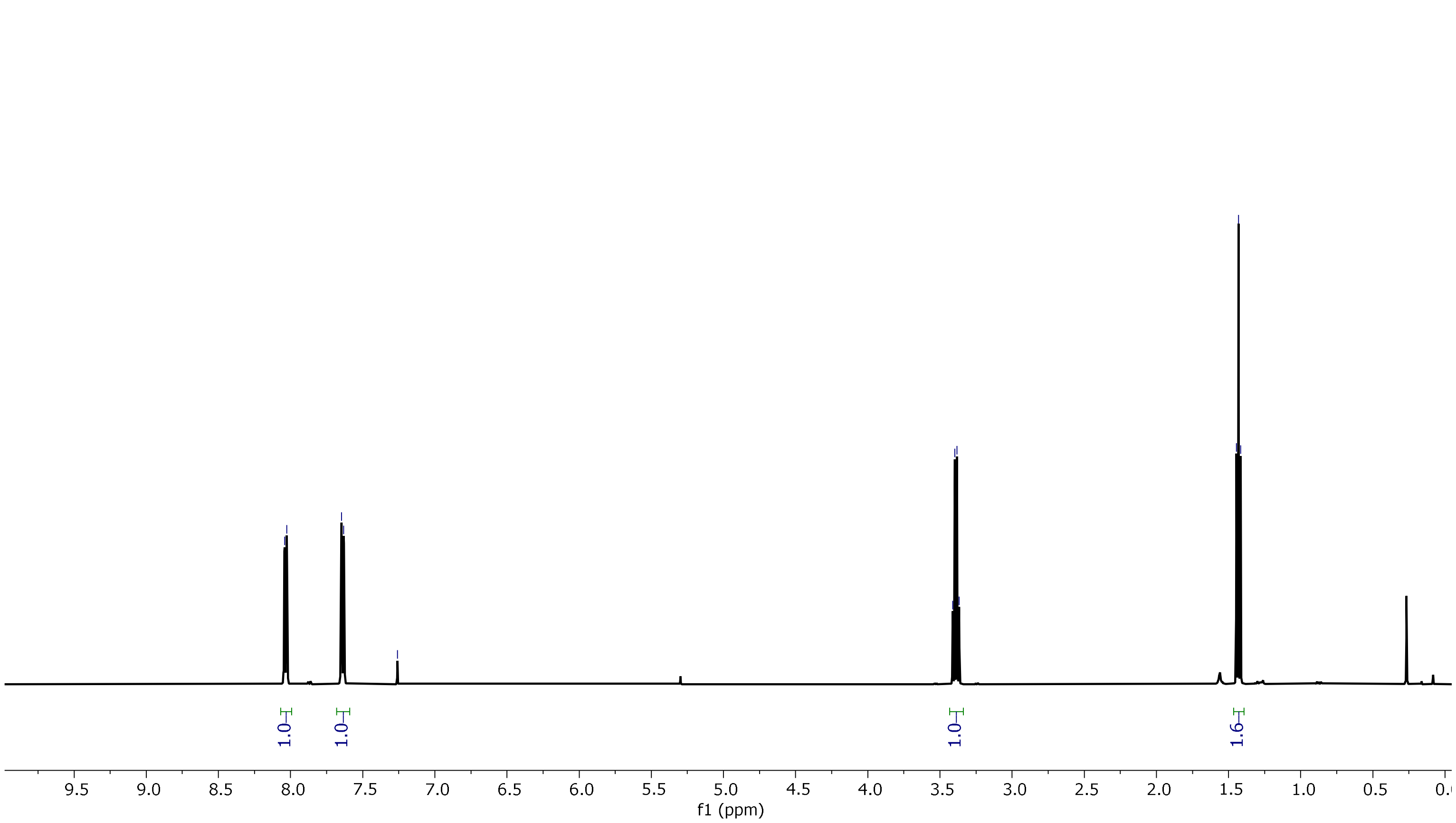

Figure S16. ^{13}C NMR (125 MHz, CDCl$_3$) spectrum for **2h**

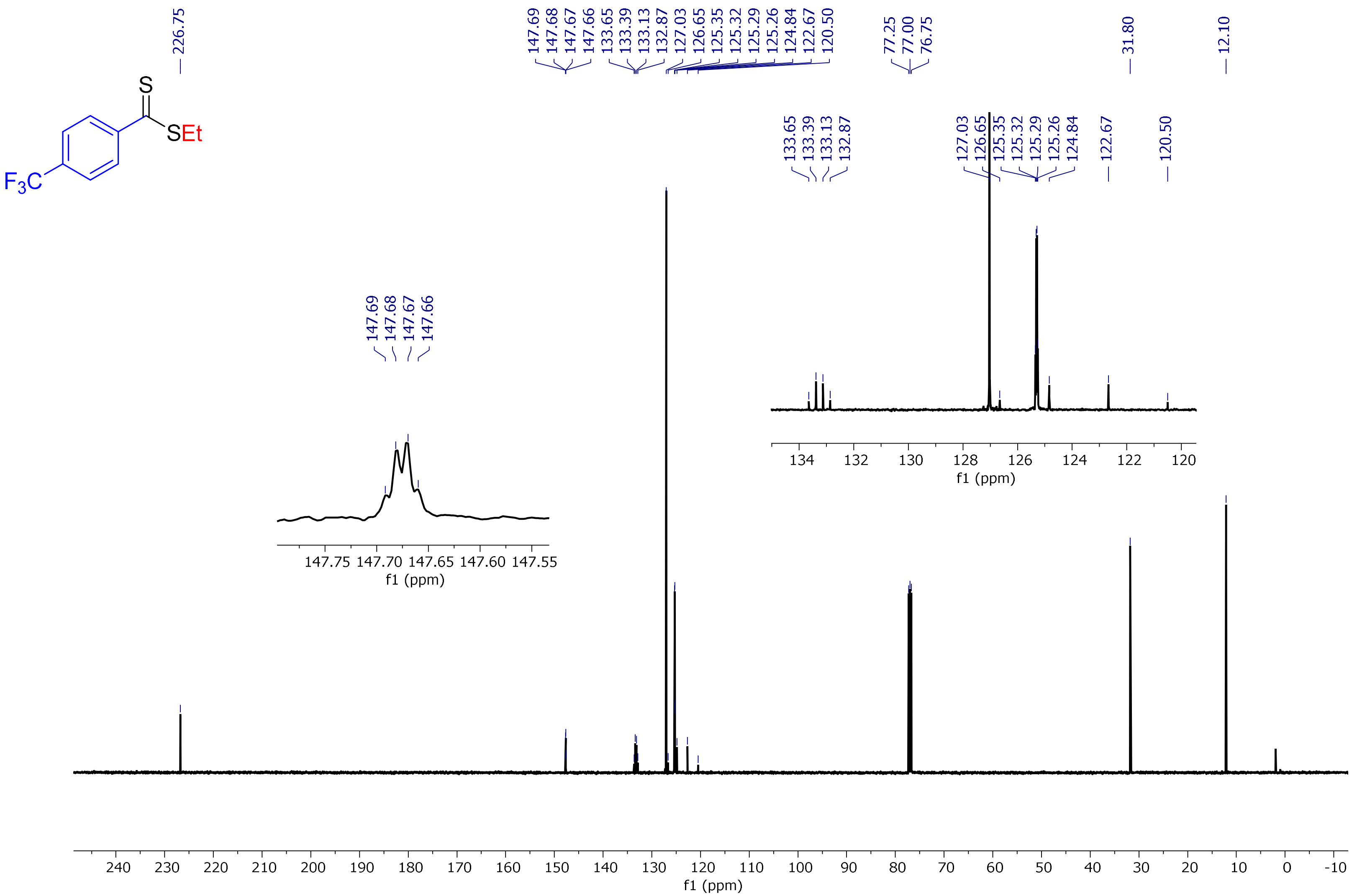

Figure S17. ^{1}H NMR (500 MHz, $CDCl_3$) spectrum for **2i**

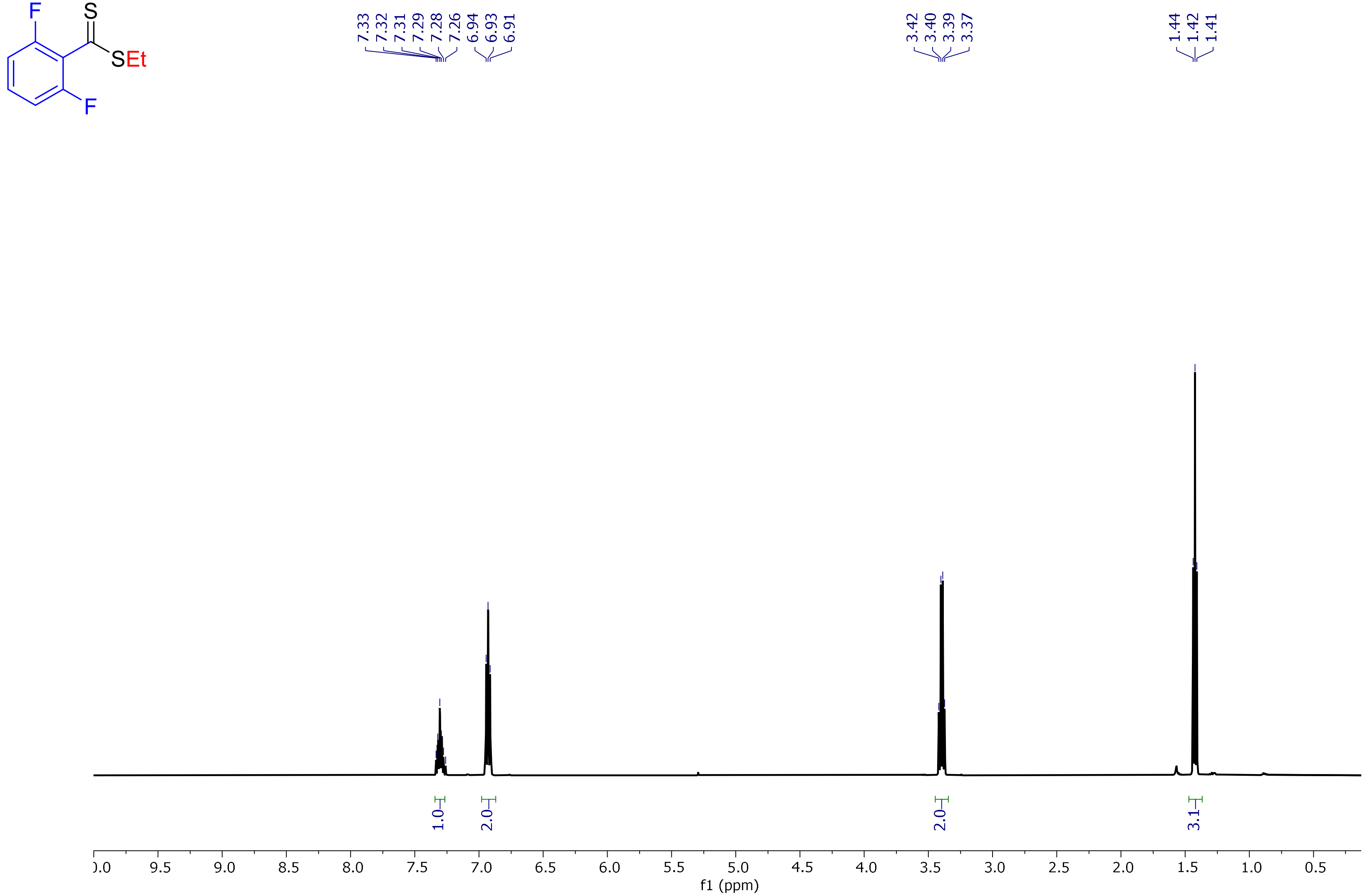

Figure S18. ^{13}C NMR (125 MHz, $CDCl_3$) spectrum for **2i**

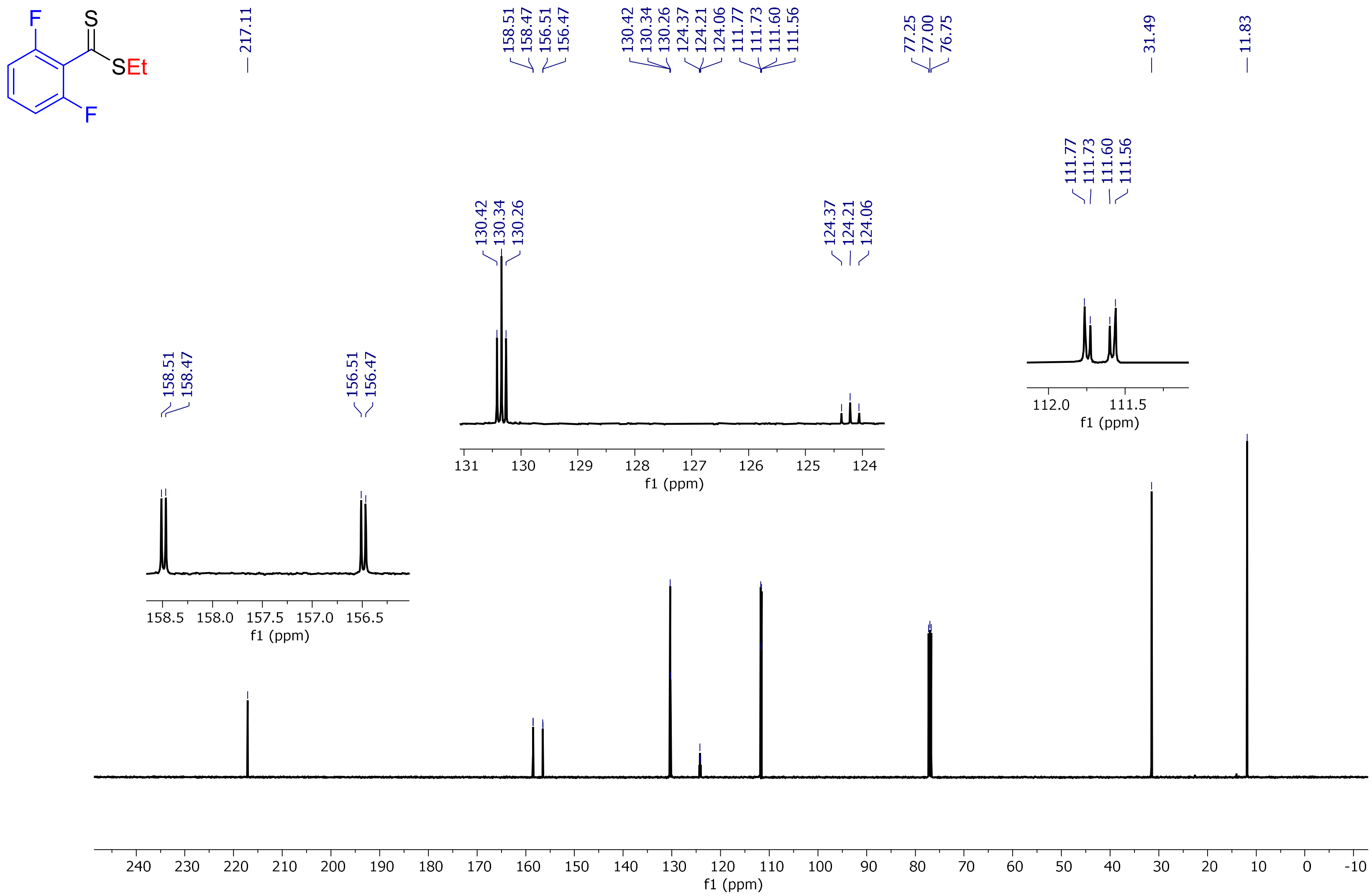

Figure S19. ^{1}H NMR (500 MHz, $CDCl_3$) spectrum for **2j**

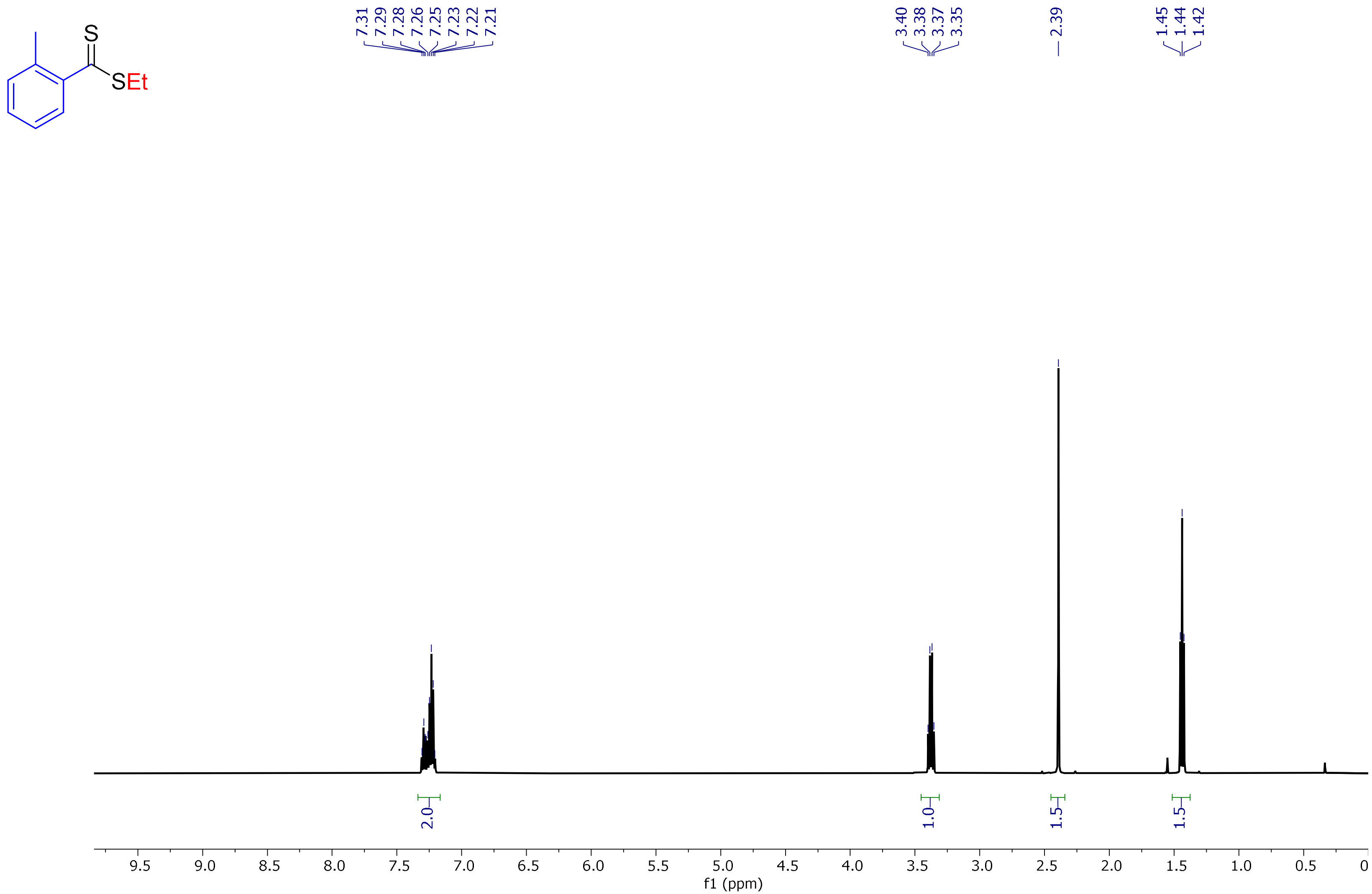

Figure S20. ^{13}C NMR (125 MHz, $CDCl_3$) spectrum for **2j**

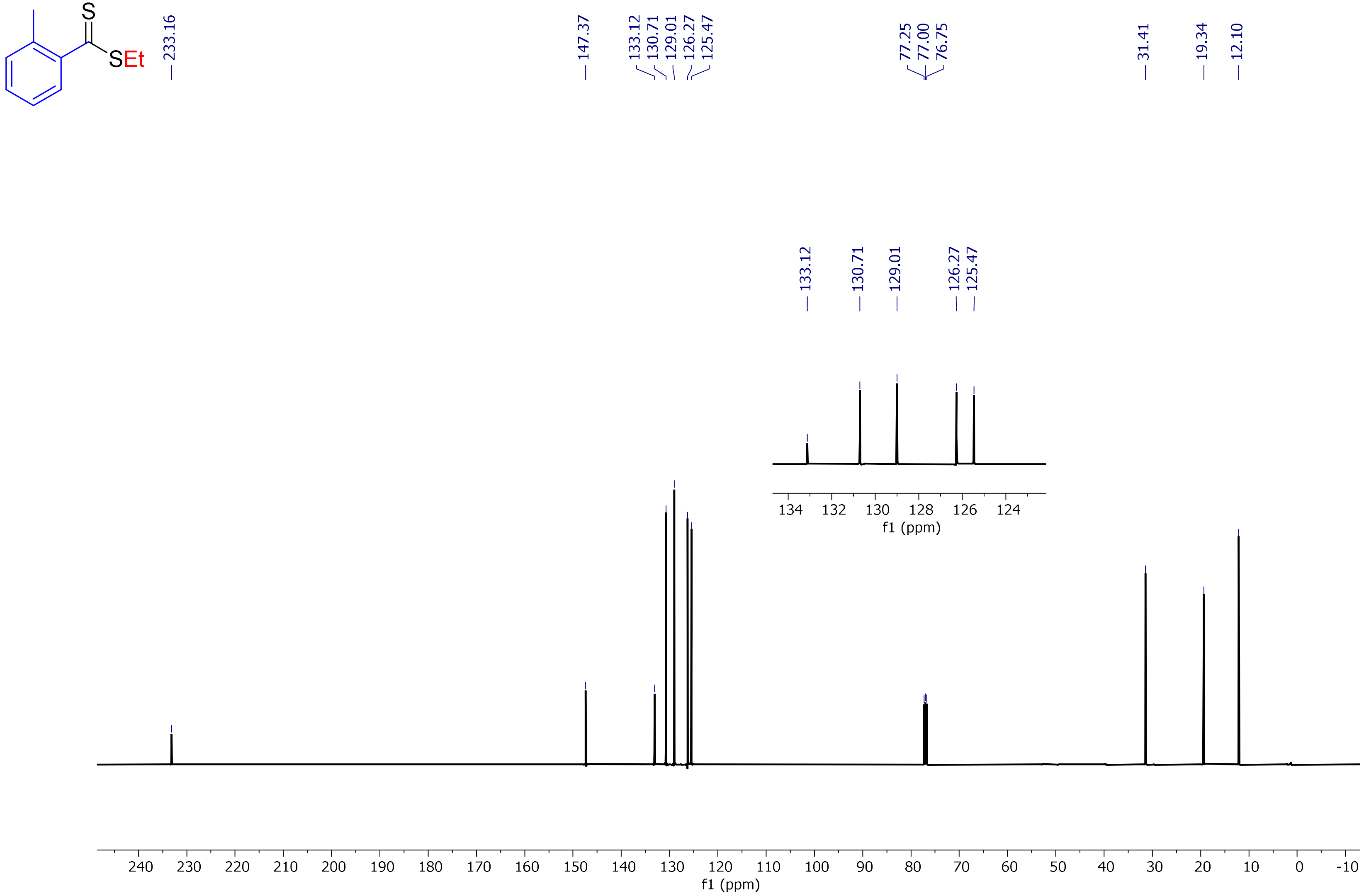

Figure S21. ^{1}H NMR (500 MHz, $CDCl_3$) spectrum for **2k**

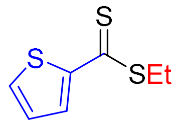

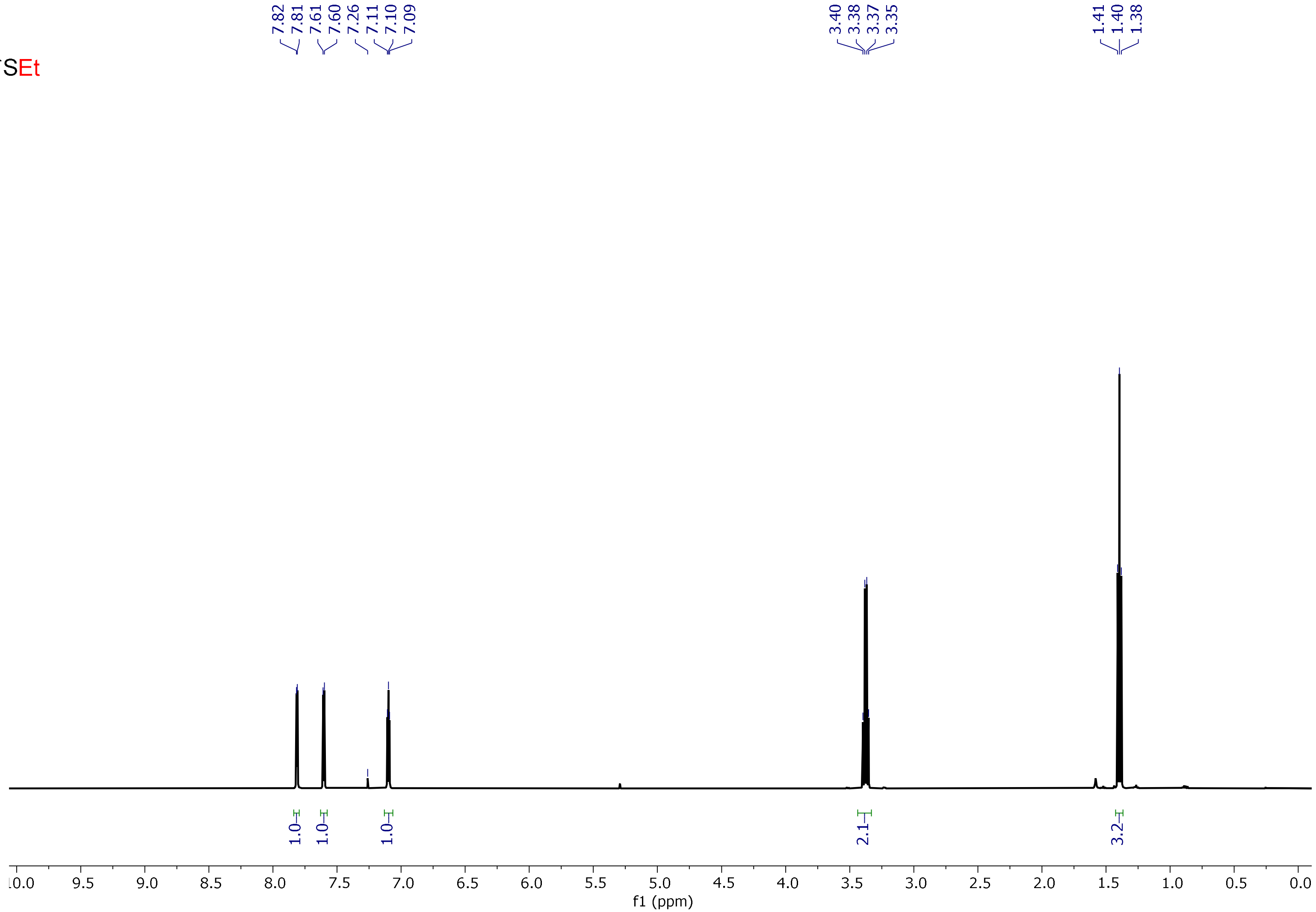

Figure S22. ^{13}C NMR (125 MHz, $CDCl_3$) spectrum for **2k**

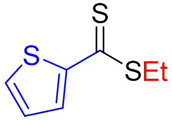

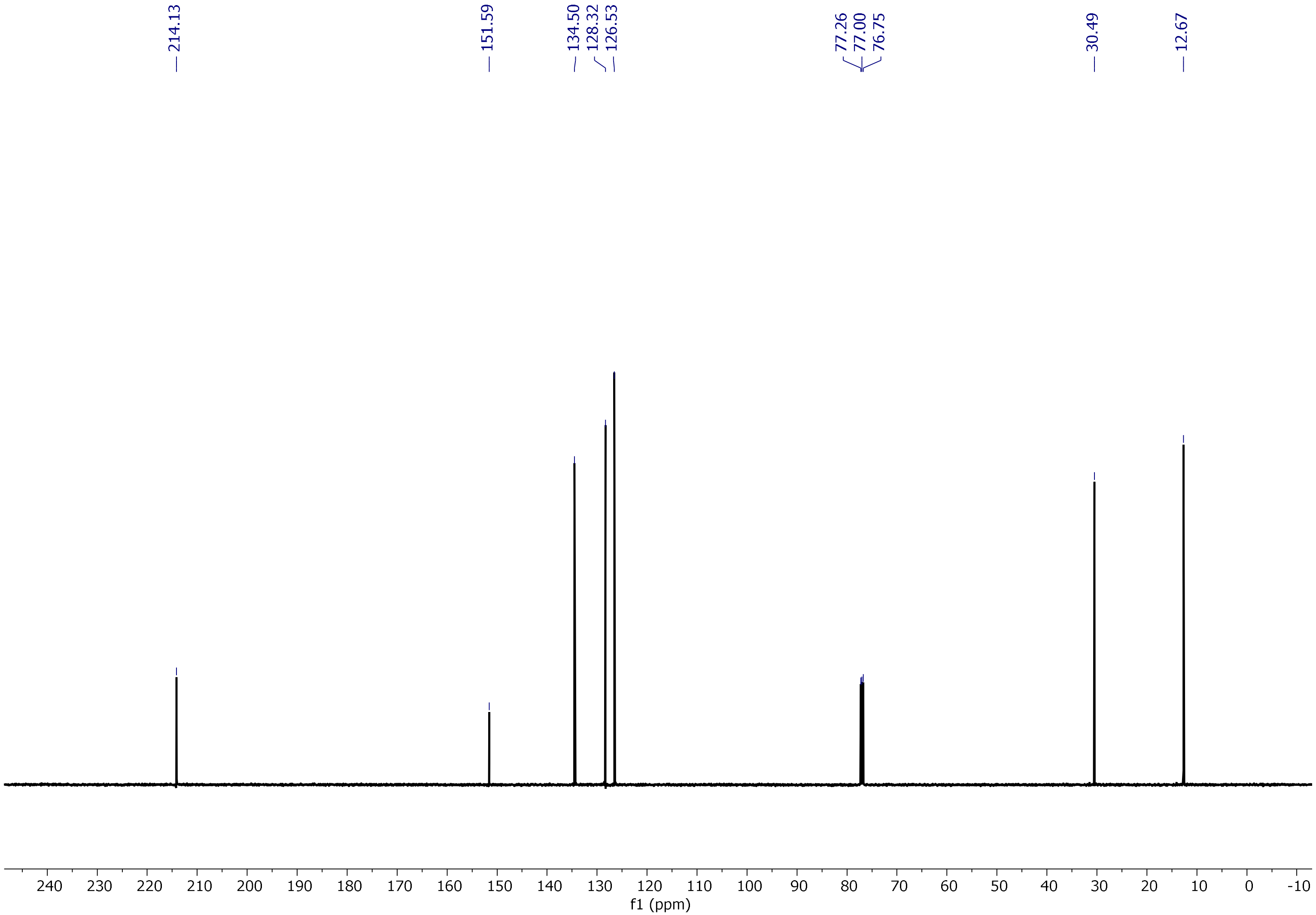

Figure S23. ^{1}H NMR (500 MHz, $CDCl_3$) spectrum for **2l**

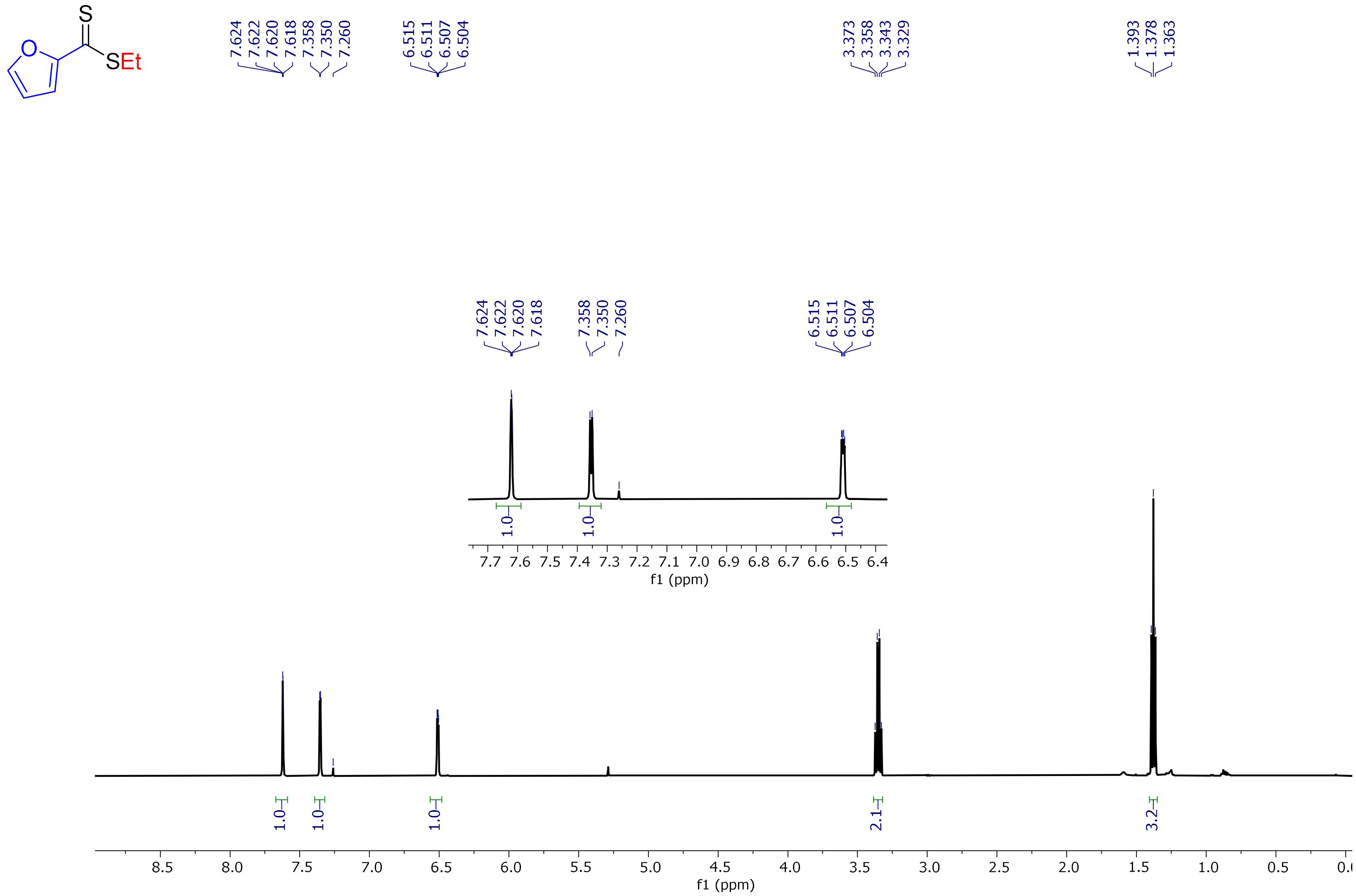

Figure S24. ^{13}C NMR (125 MHz, $CDCl_3$) spectrum for **2l**

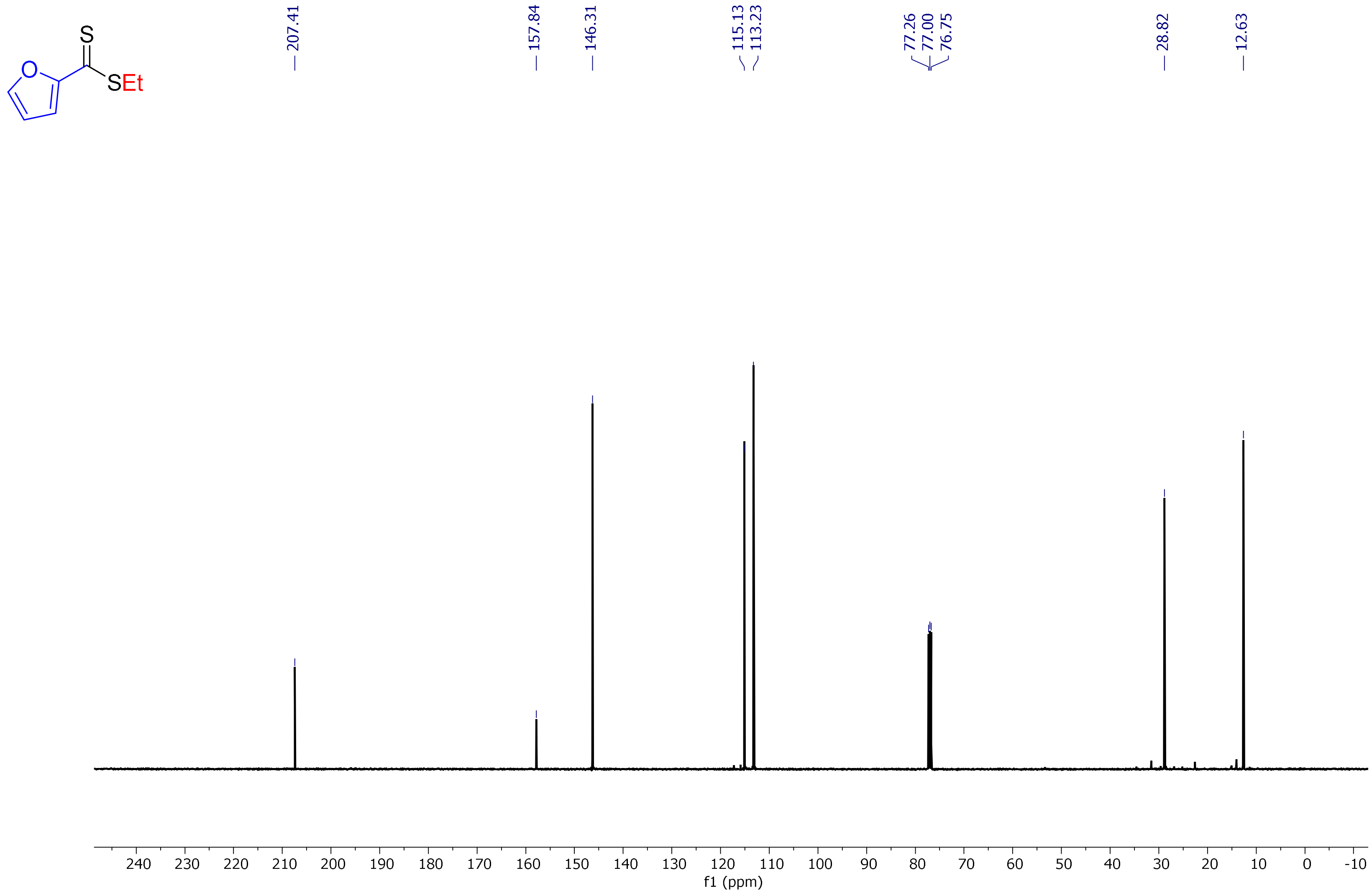

Figure S25. ^{1}H NMR (500 MHz, $CDCl_3$) spectrum for **2n**

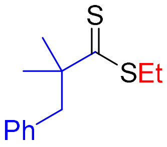

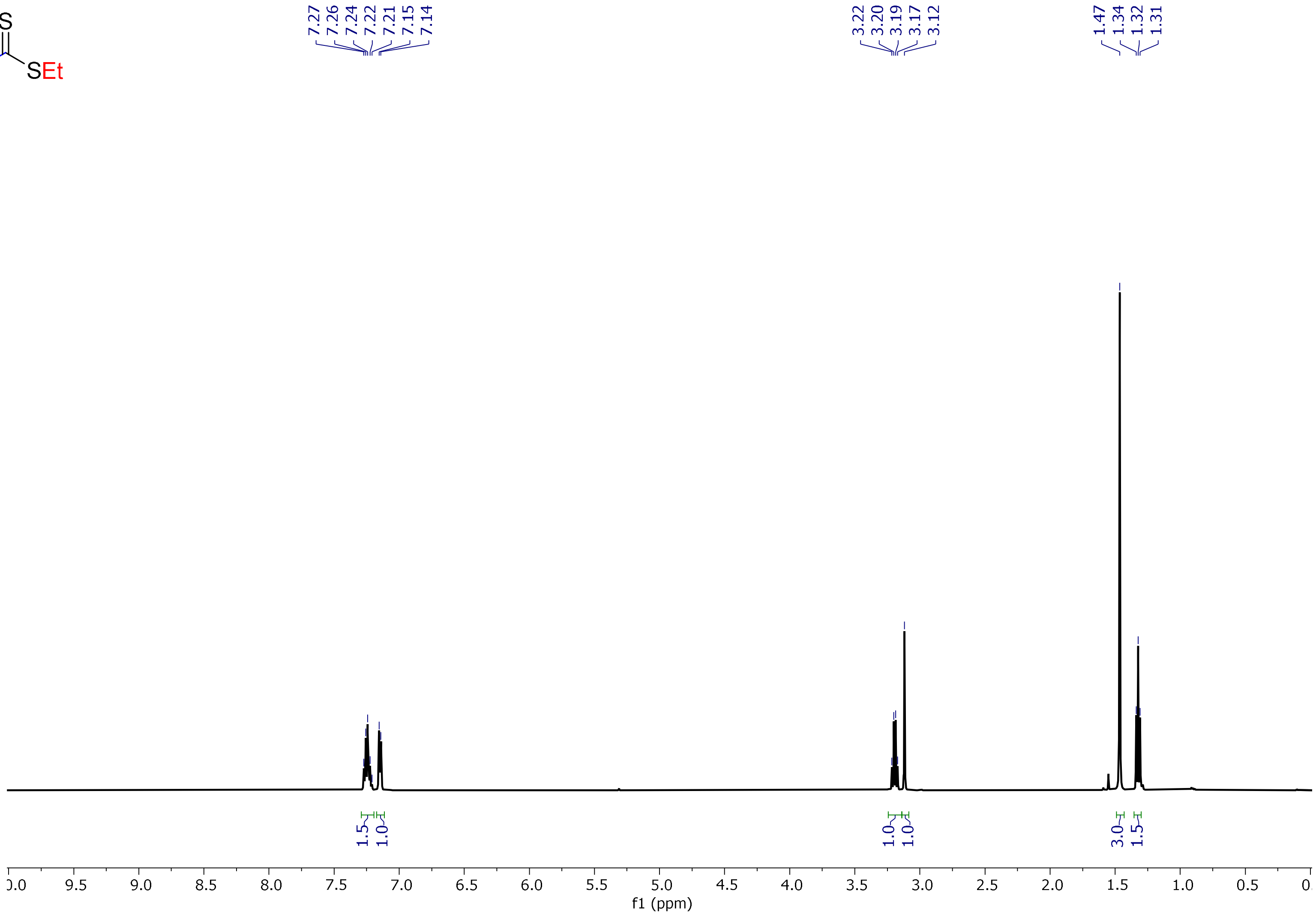

Figure S26. ^{13}C NMR (125 MHz, $CDCl_3$) spectrum for **2n**

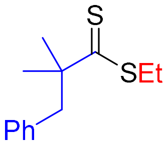

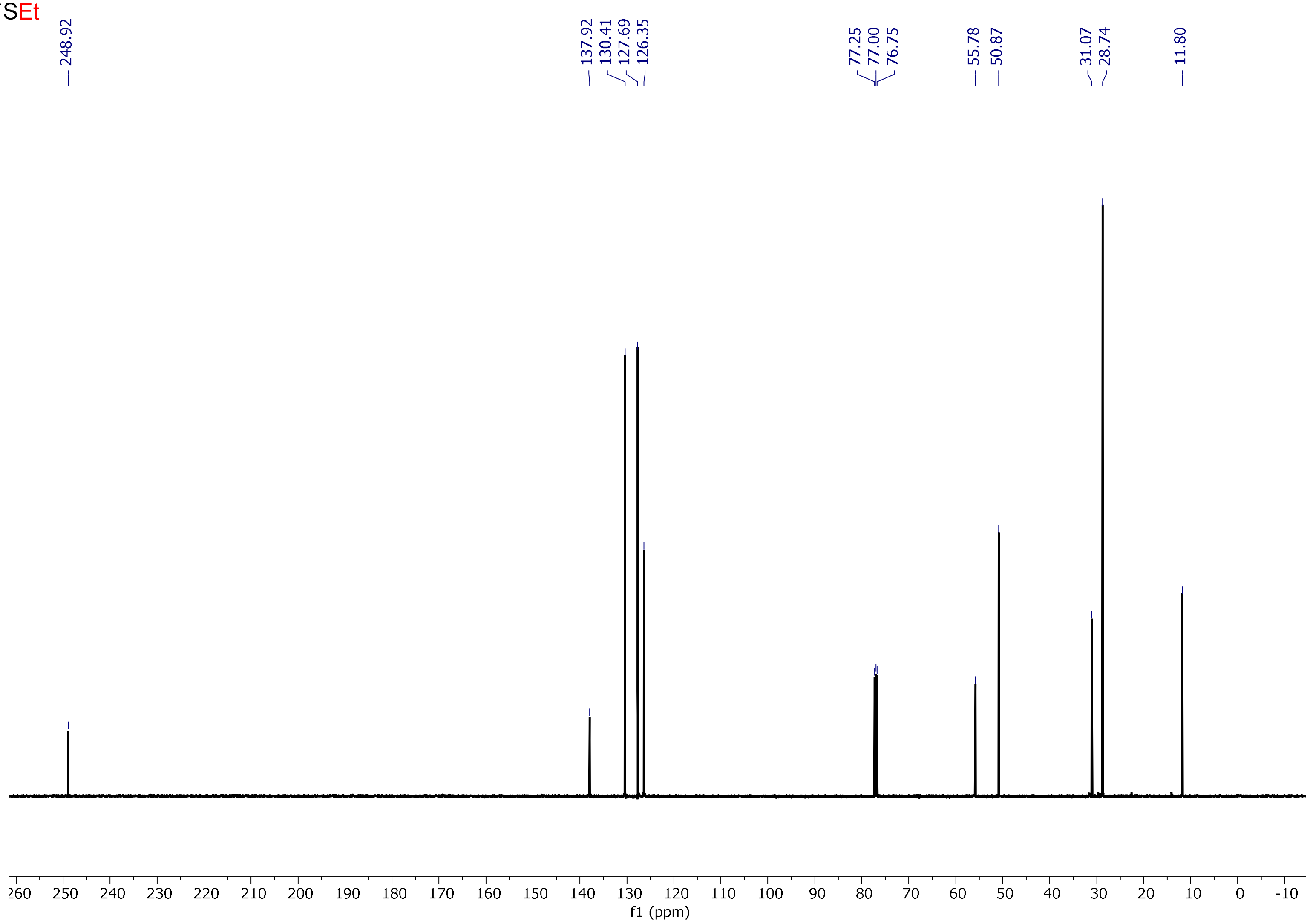

Figure S27. ^{1}H NMR (500 MHz, $CDCl_3$) spectrum for **3a**

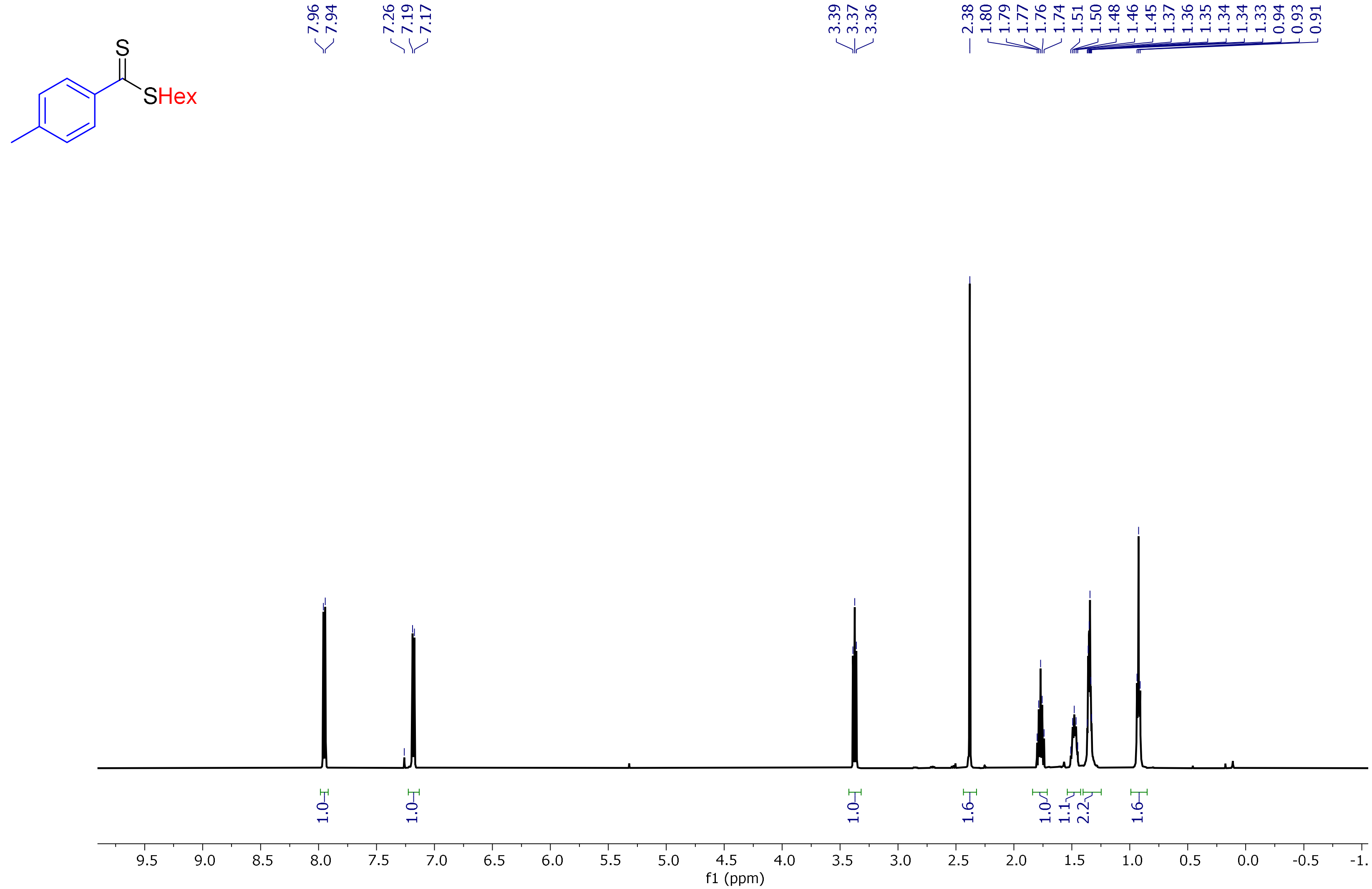

Figure S28. ^{13}C NMR (125 MHz, $CDCl_3$) spectrum for **3a**

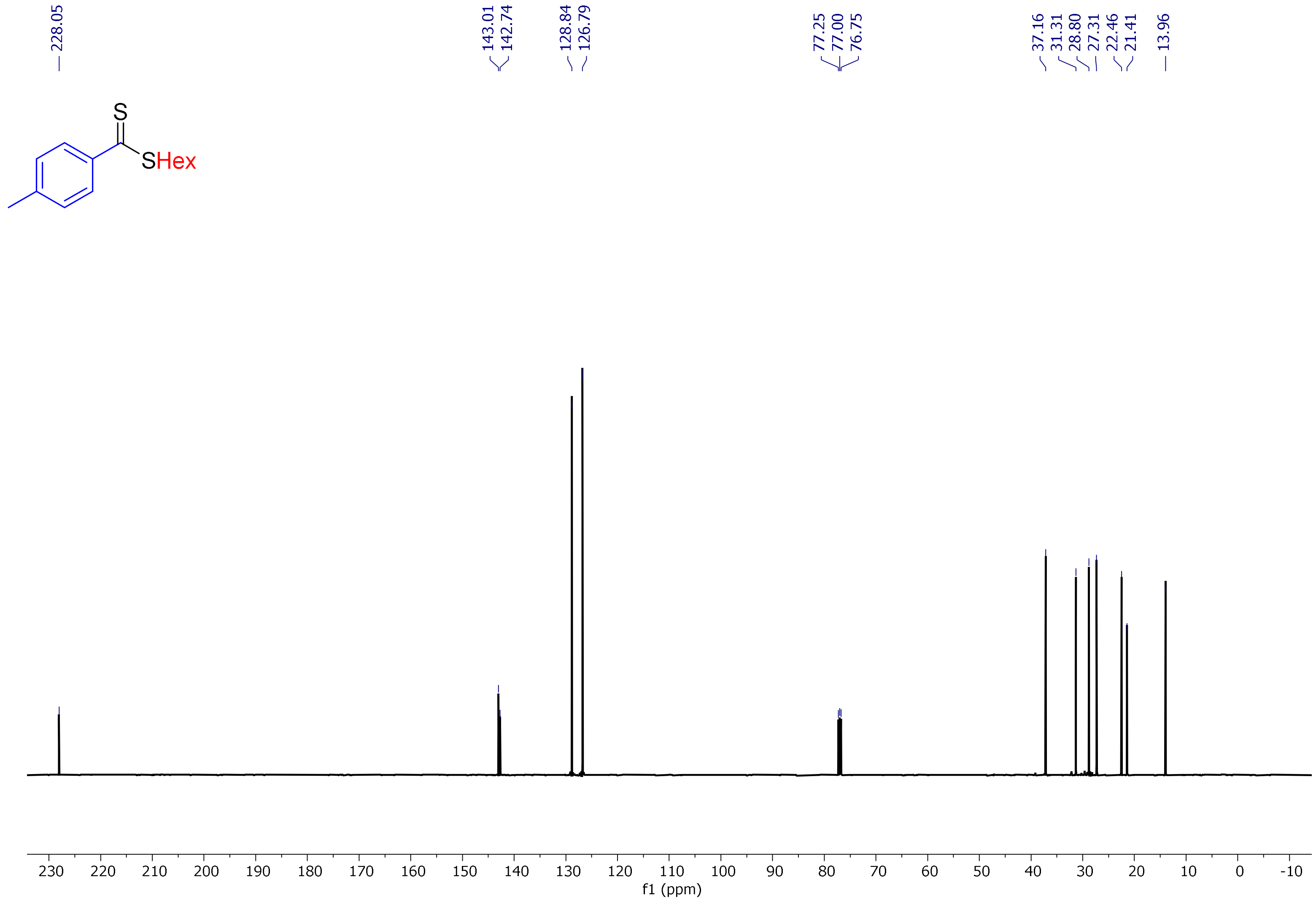

Figure S29. ^{1}H NMR (500 MHz, $CDCl_3$) spectrum for **4a**

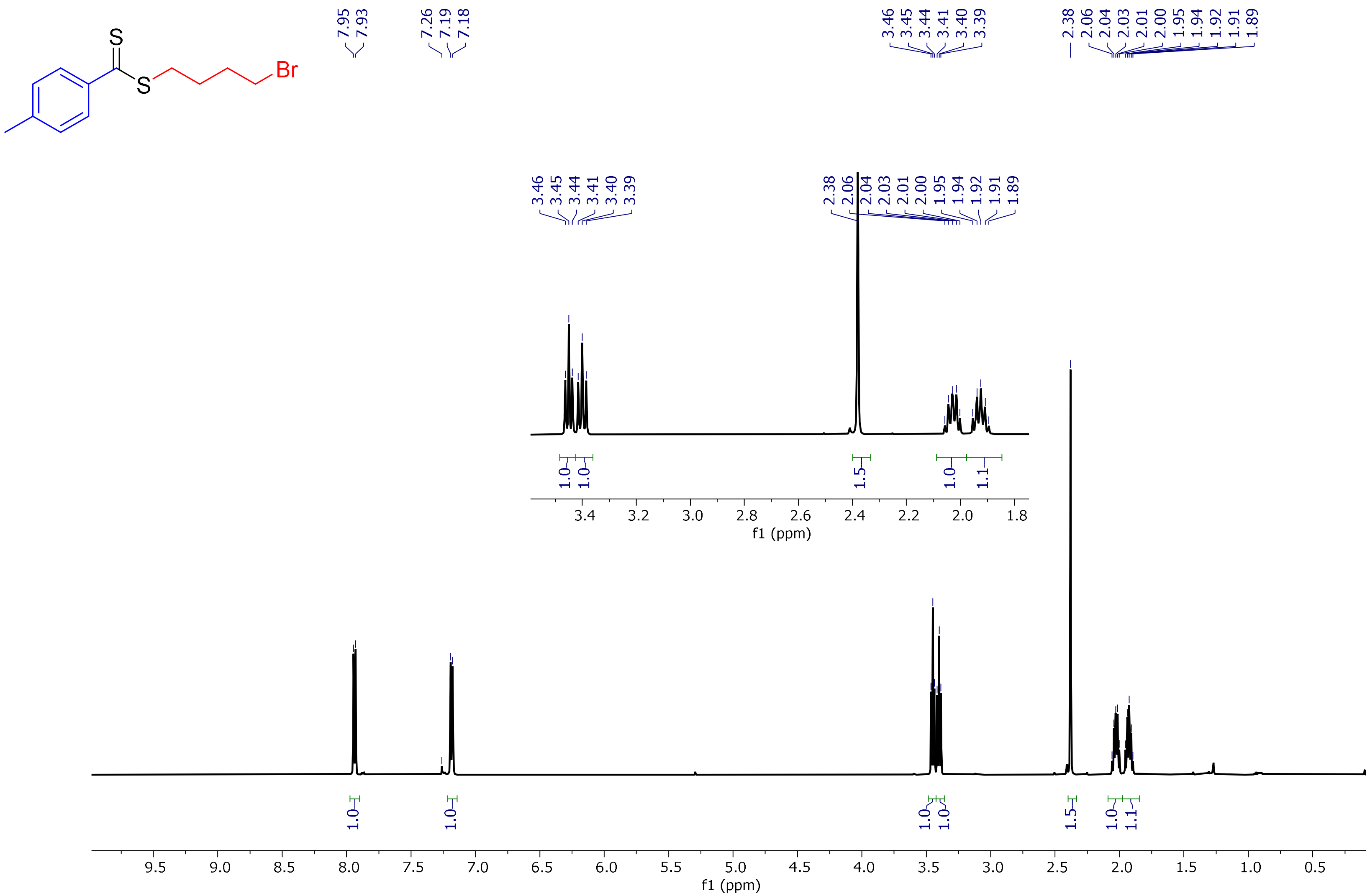

Figure S30. ^{13}C NMR (125 MHz, $CDCl_3$) spectrum for **4a**

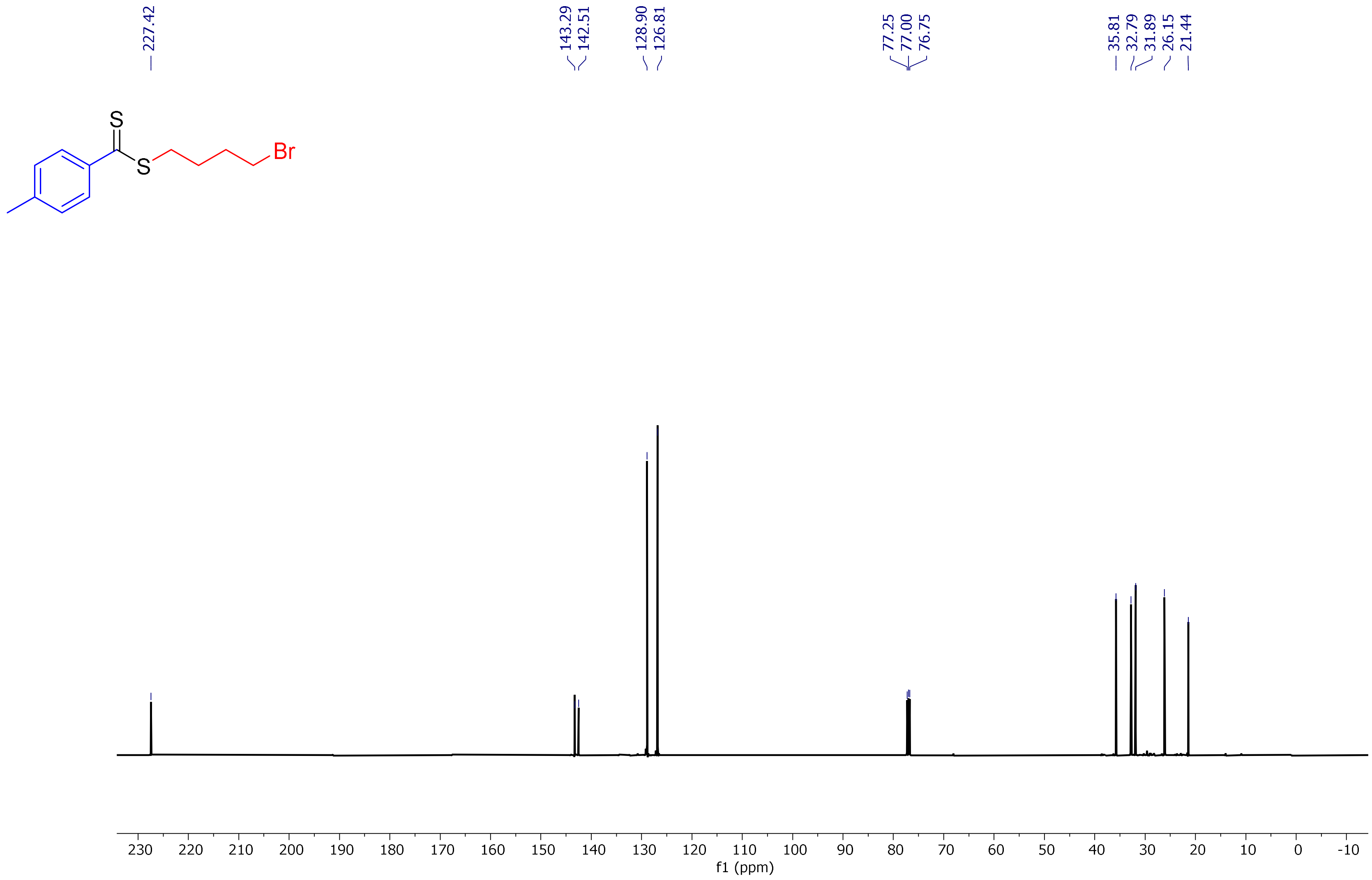

Figure S31. ^{1}H NMR (500 MHz, $CDCl_3$) spectrum for **5a**

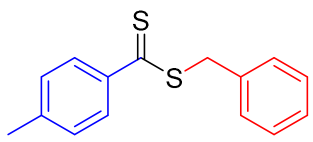

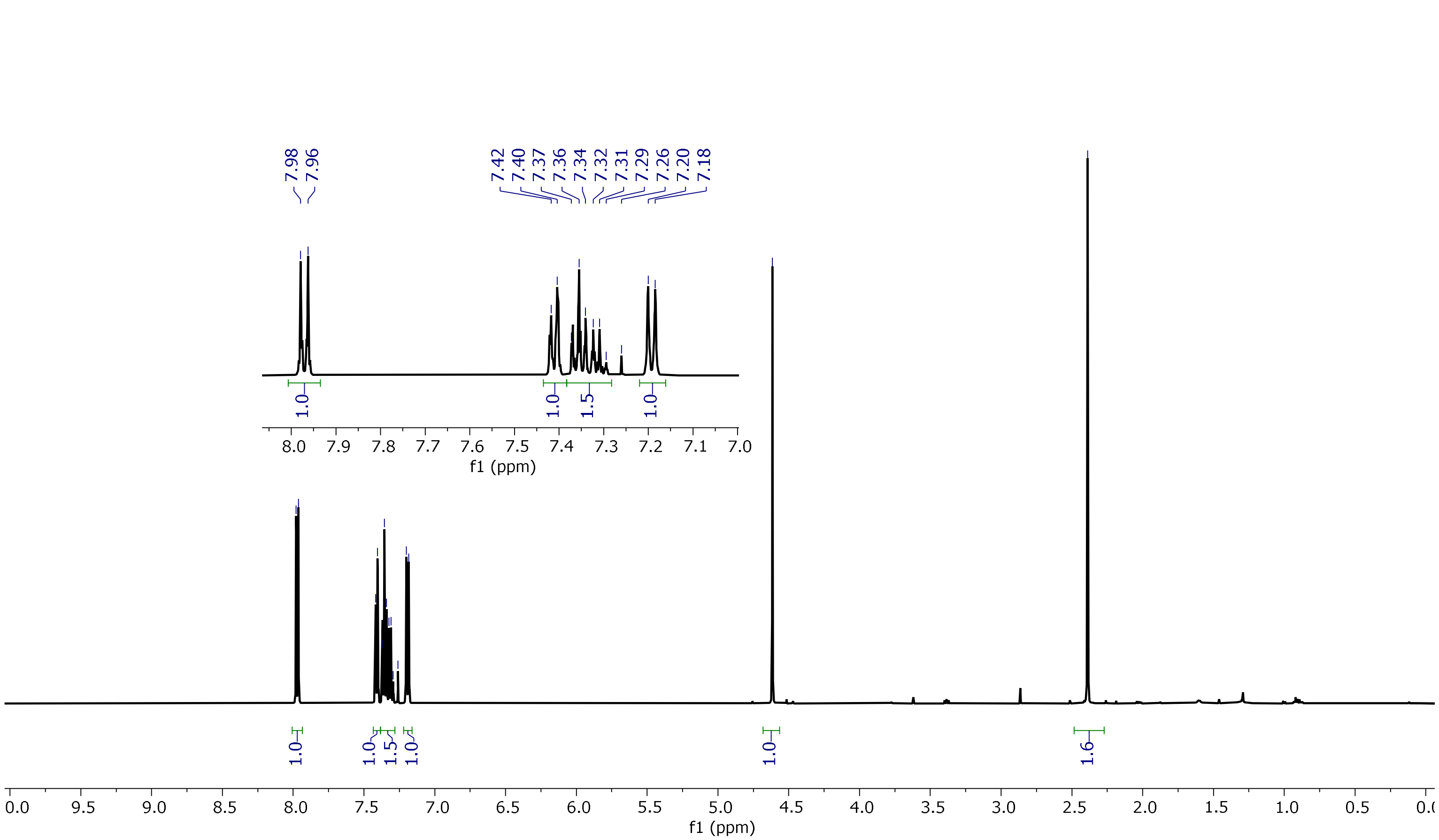

Figure S32. ^{13}C NMR (125 MHz, $CDCl_3$) spectrum for **5a**

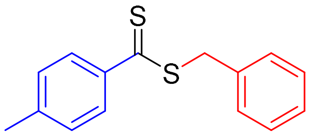

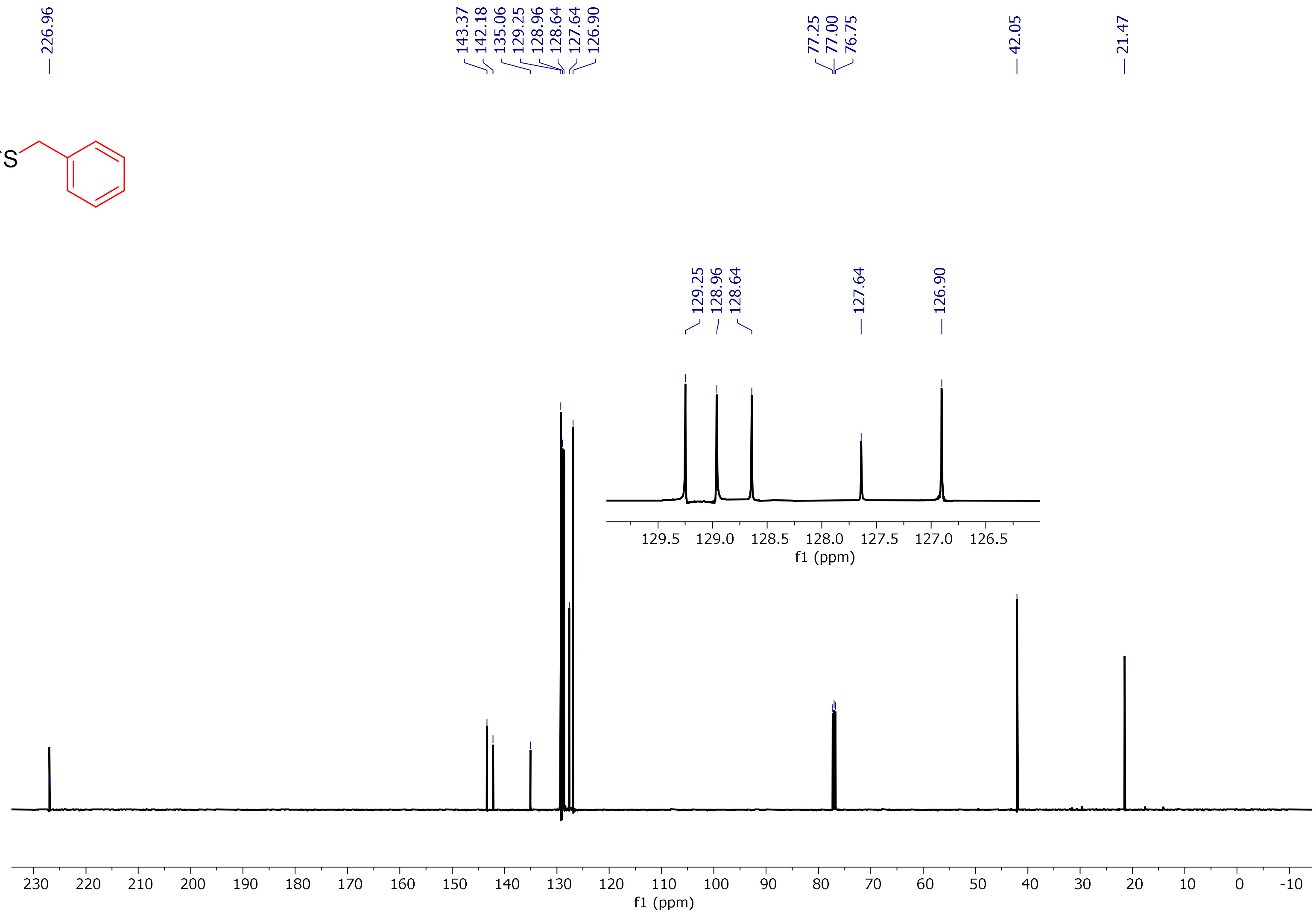

Figure S33. ^{1}H NMR (500 MHz, $CDCl_3$) spectrum for **6a**

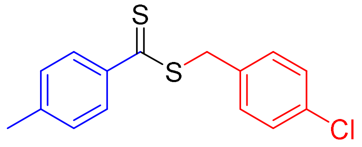

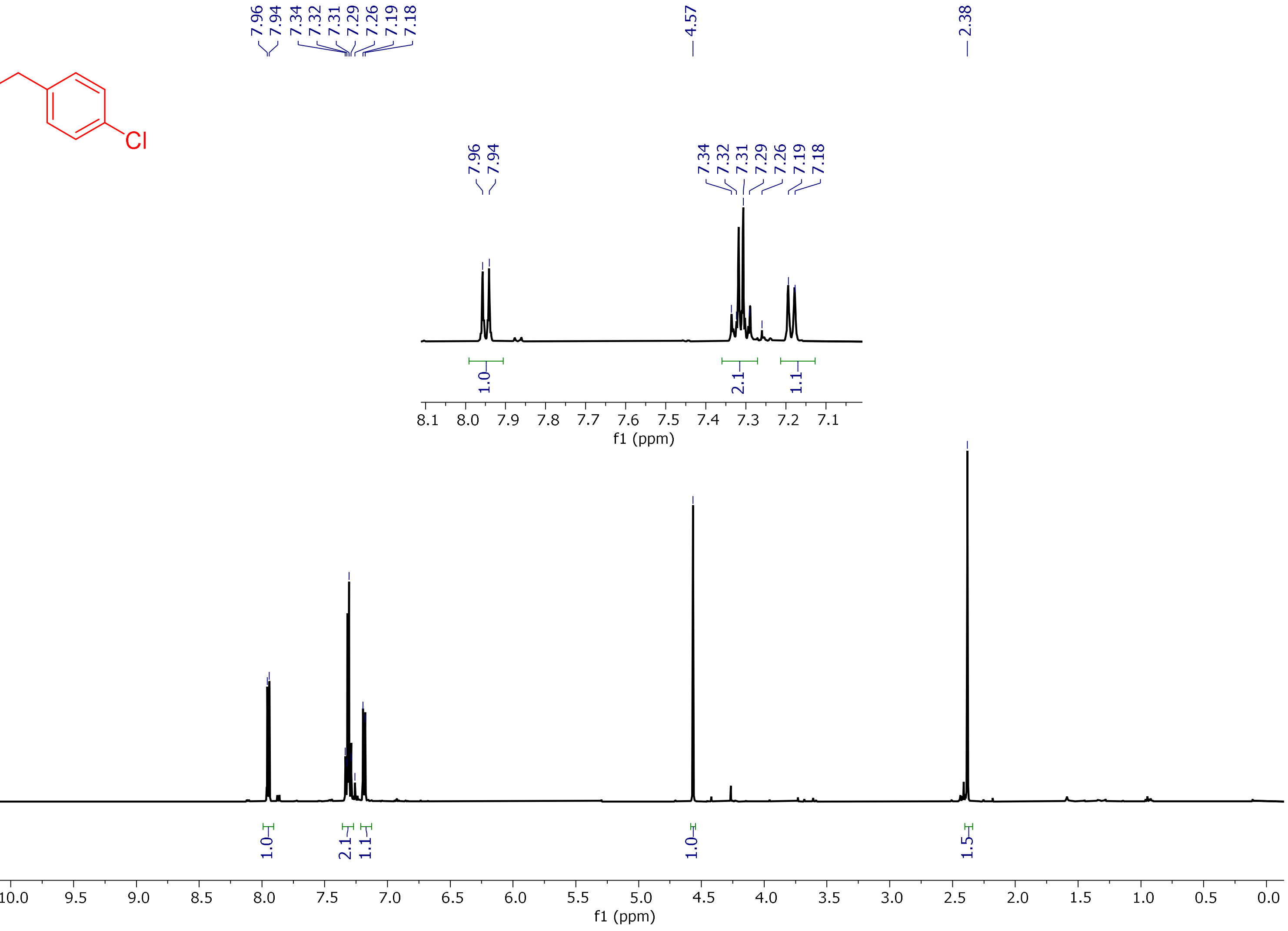

Figure S34. ^{13}C NMR (125 MHz, CDCl$_3$) spectrum for **6a**

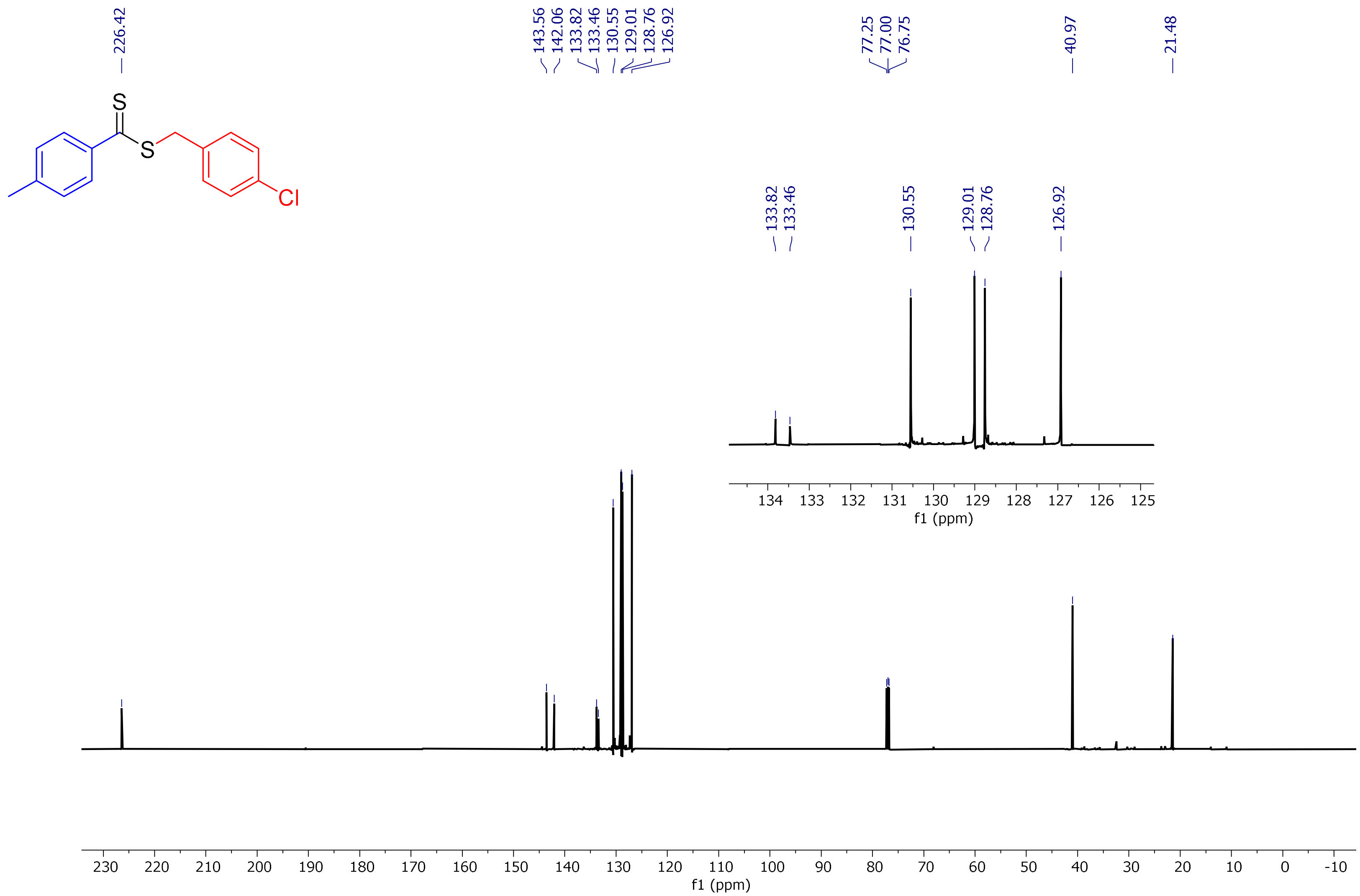

Figure S35. ^{1}H NMR (500 MHz, $CDCl_3$) spectrum for **7a**

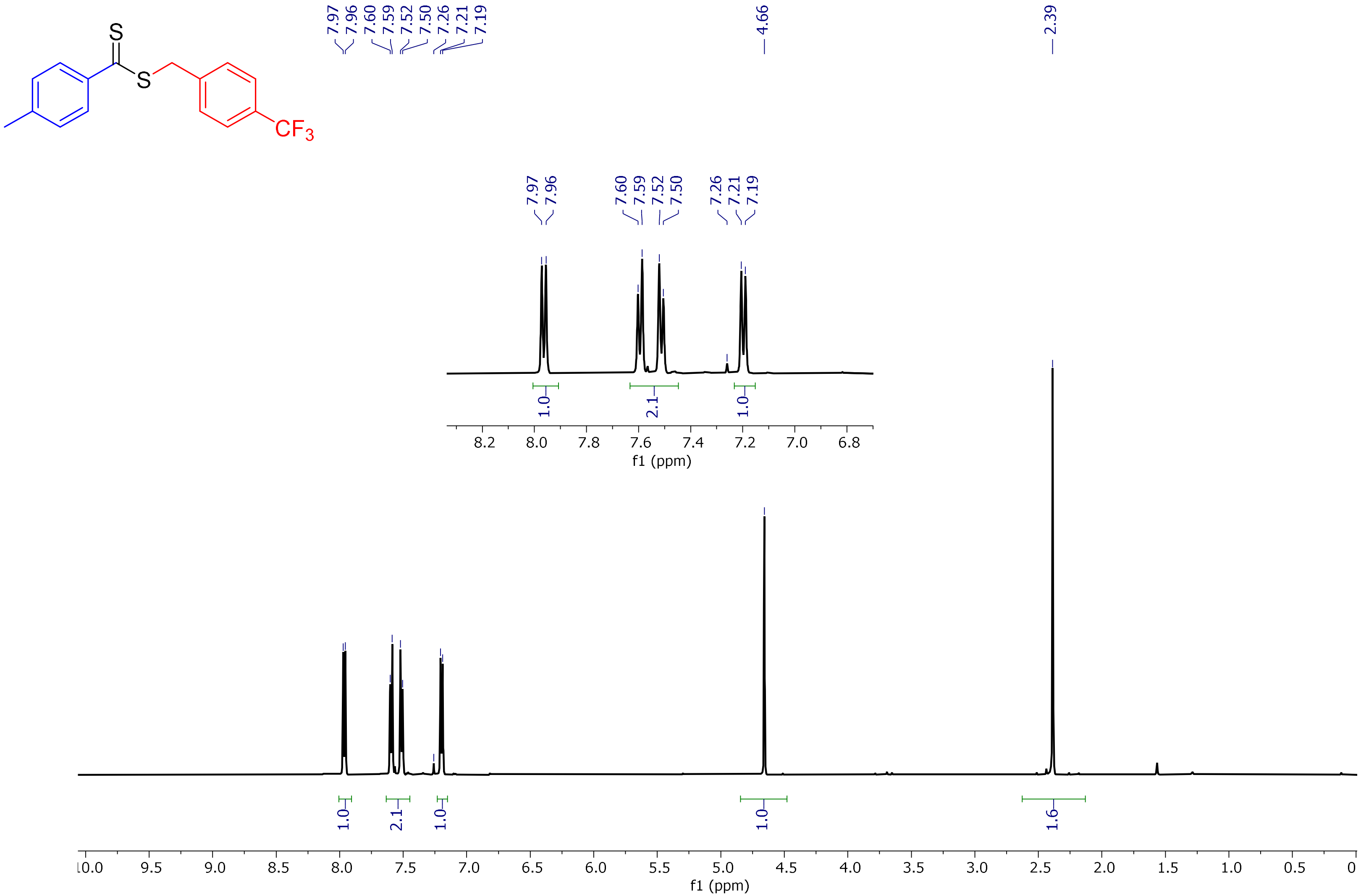

Figure S36. ^{13}C NMR (125 MHz, $CDCl_3$) spectrum for **7a**

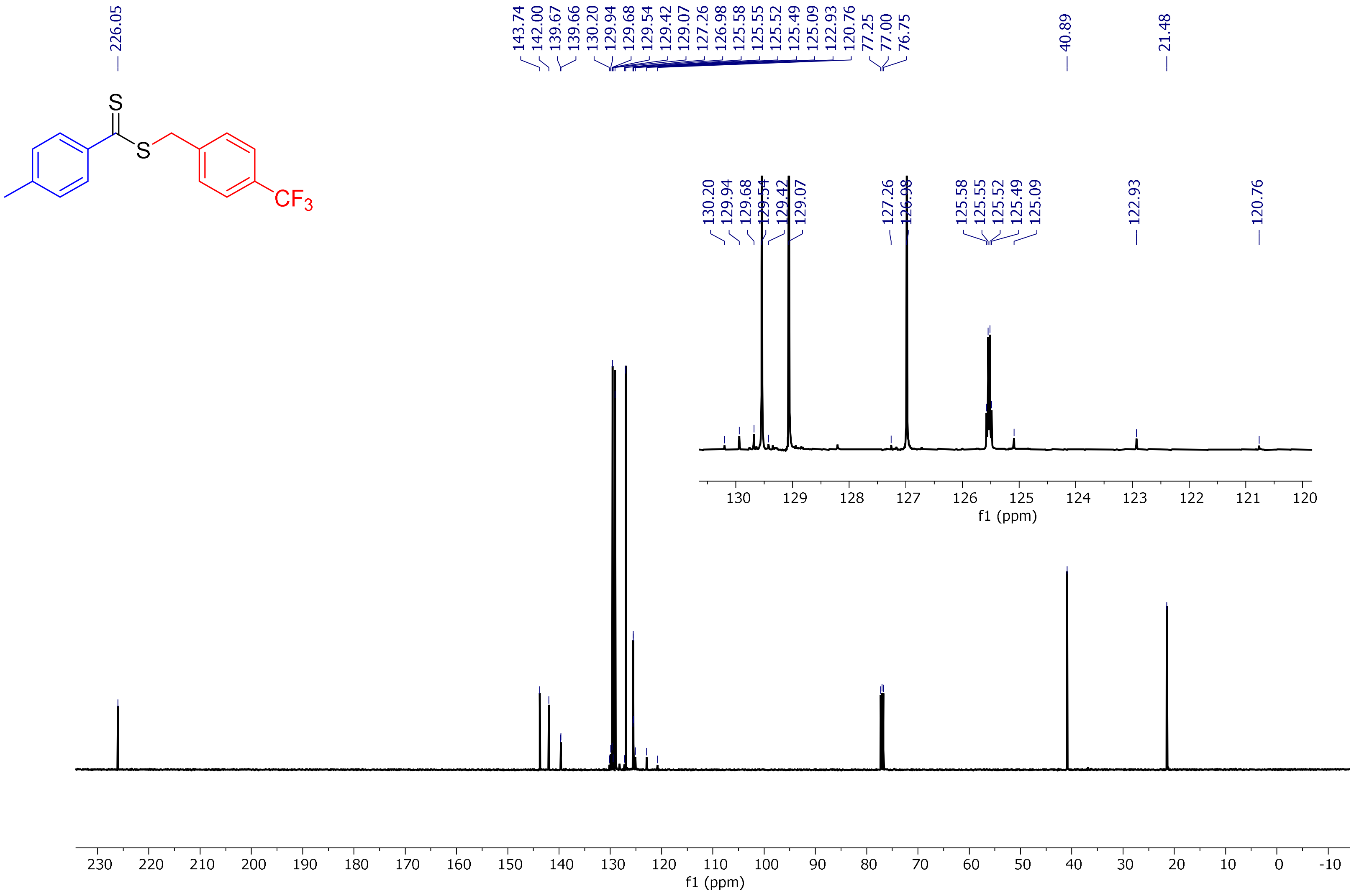

Figure S37. ^{1}H NMR (500 MHz, $CDCl_3$) spectrum for **8a**

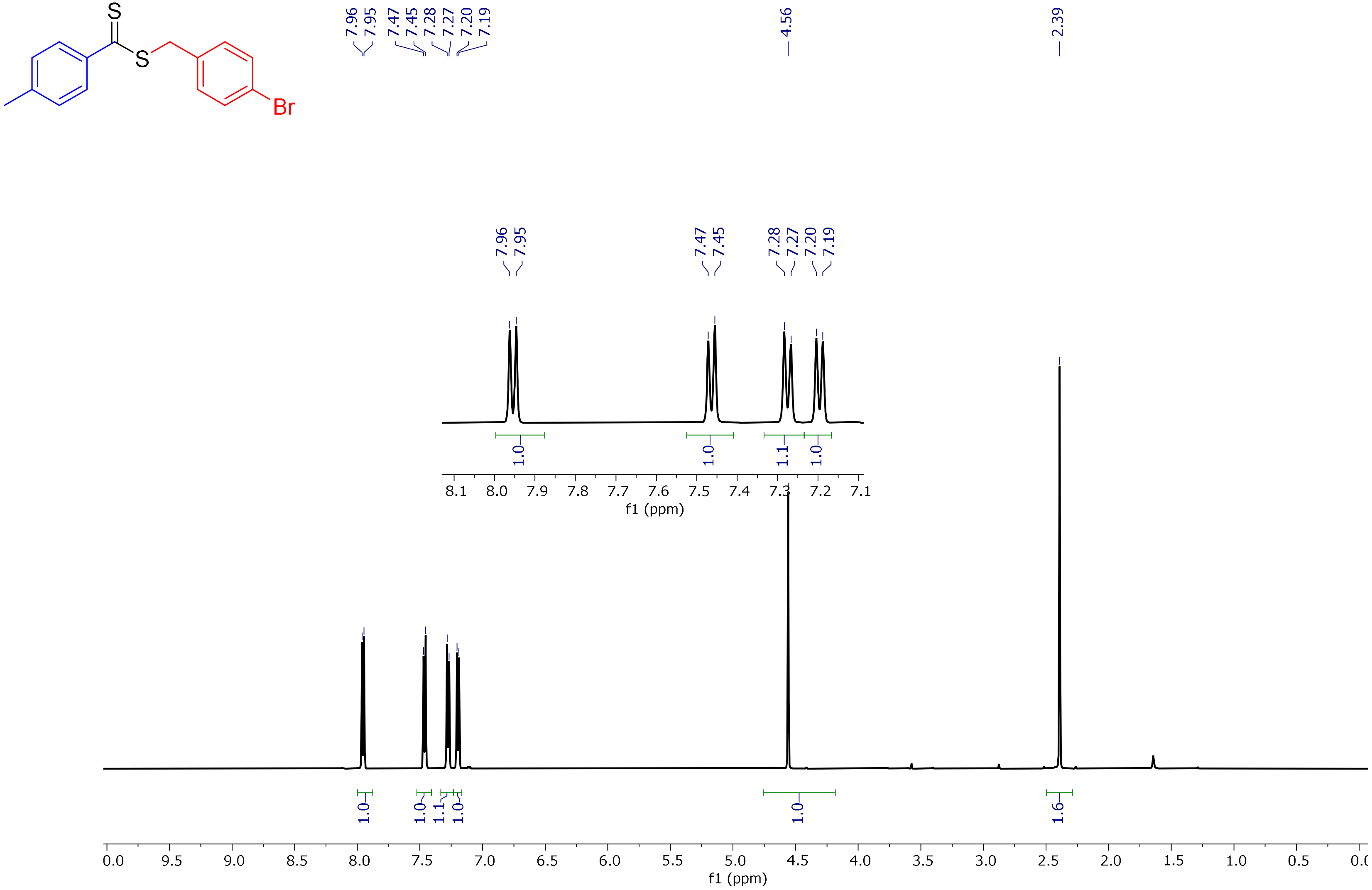

Figure S38. ^{13}C NMR (125 MHz, $CDCl_3$) spectrum for **8a**

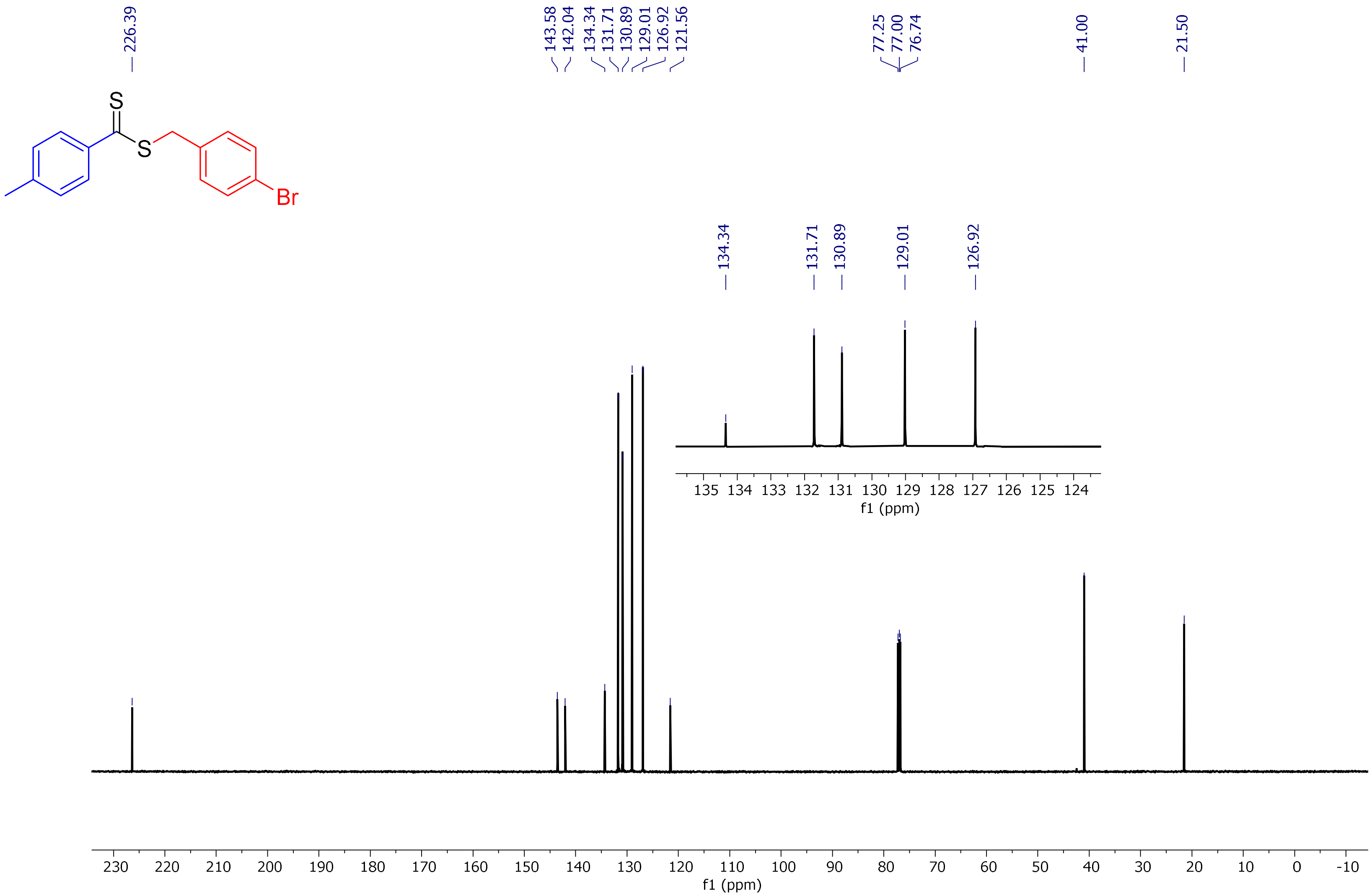

Figure S39. ^{1}H NMR (500 MHz, $CDCl_3$) spectrum for **9a**

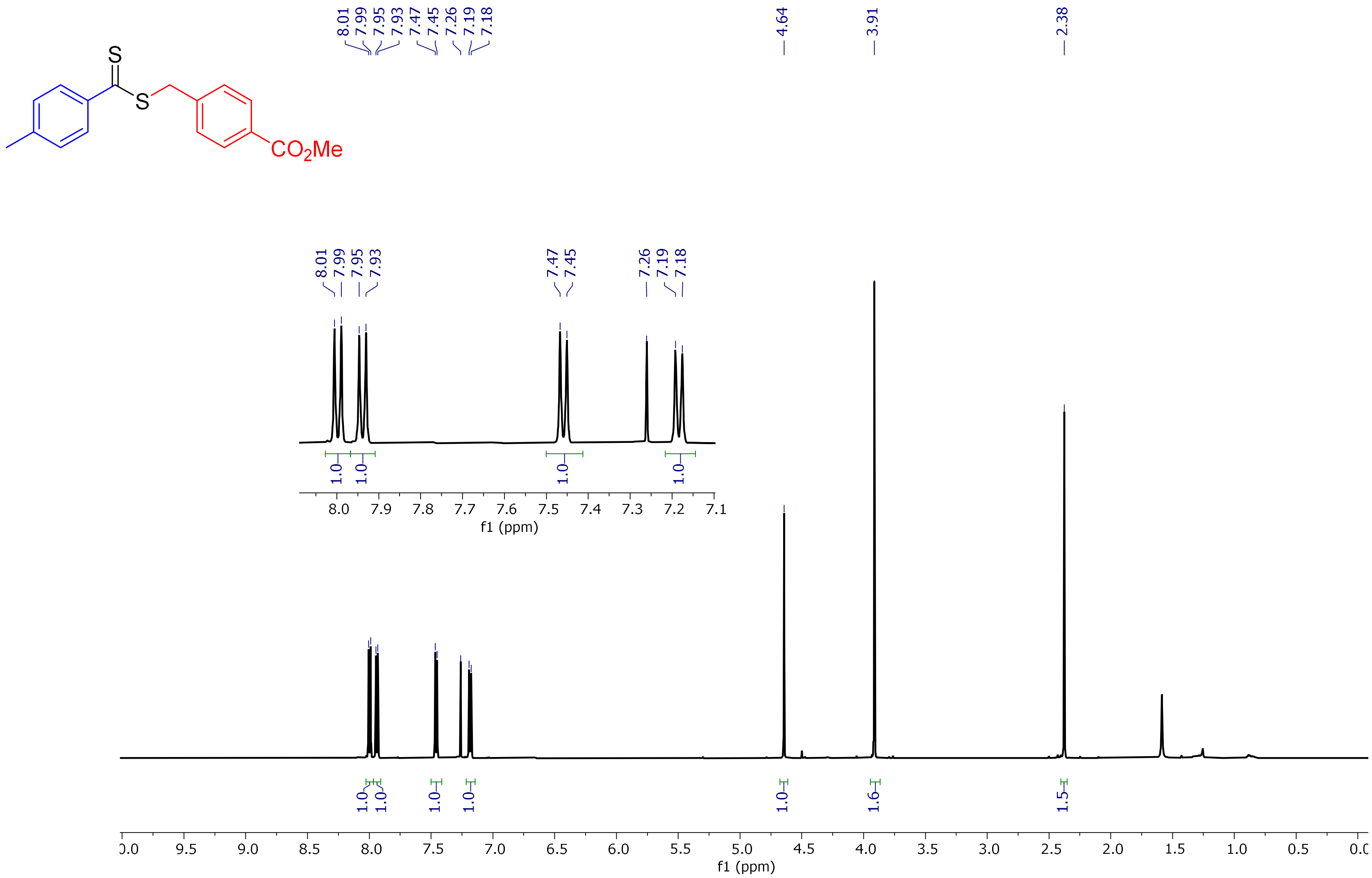

Figure S40. ^{13}C NMR (125 MHz, $CDCl_3$) spectrum for **9a**

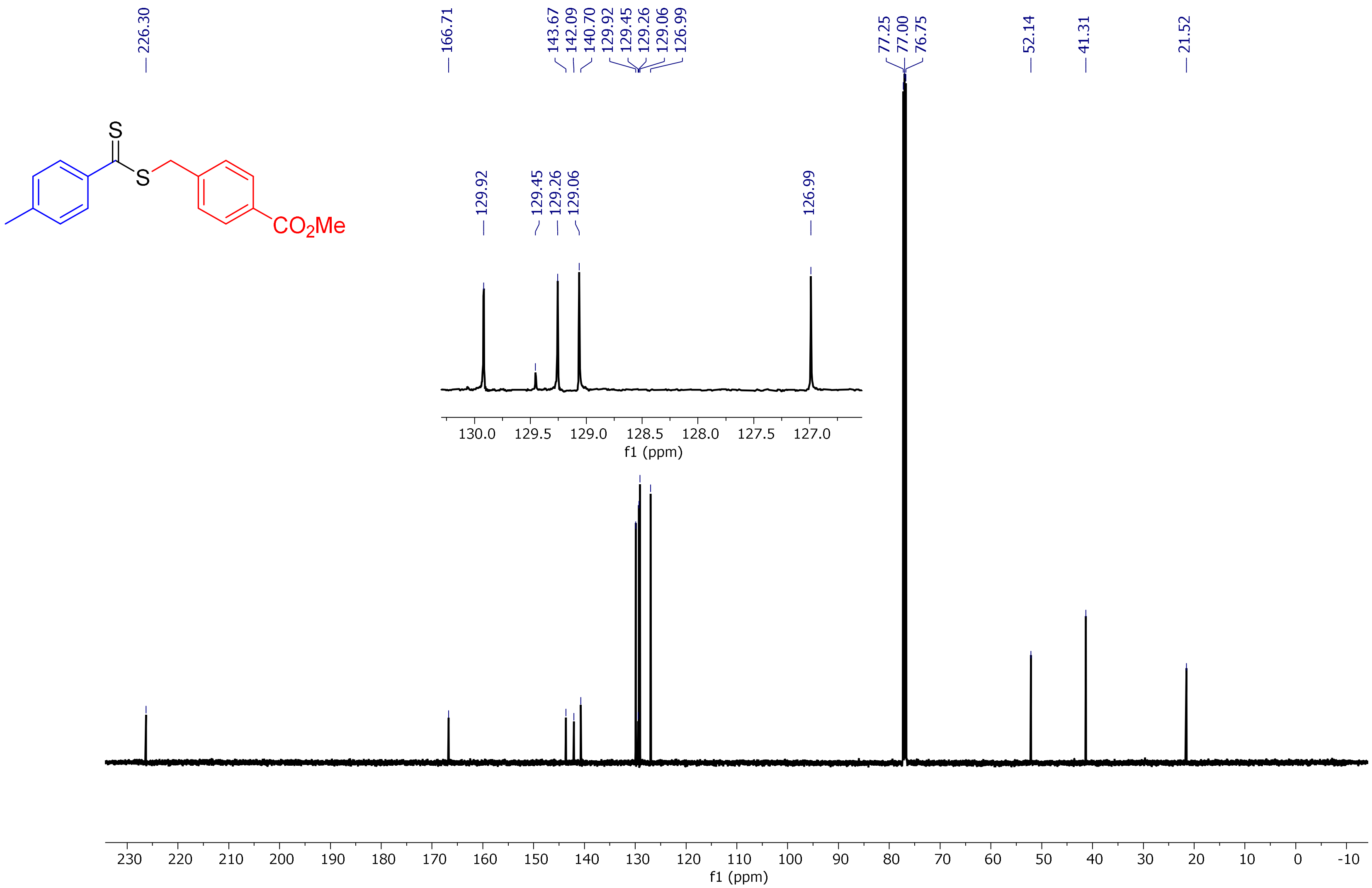

Figure S41. ^{1}H NMR (500 MHz, $CDCl_3$) spectrum for **10a**

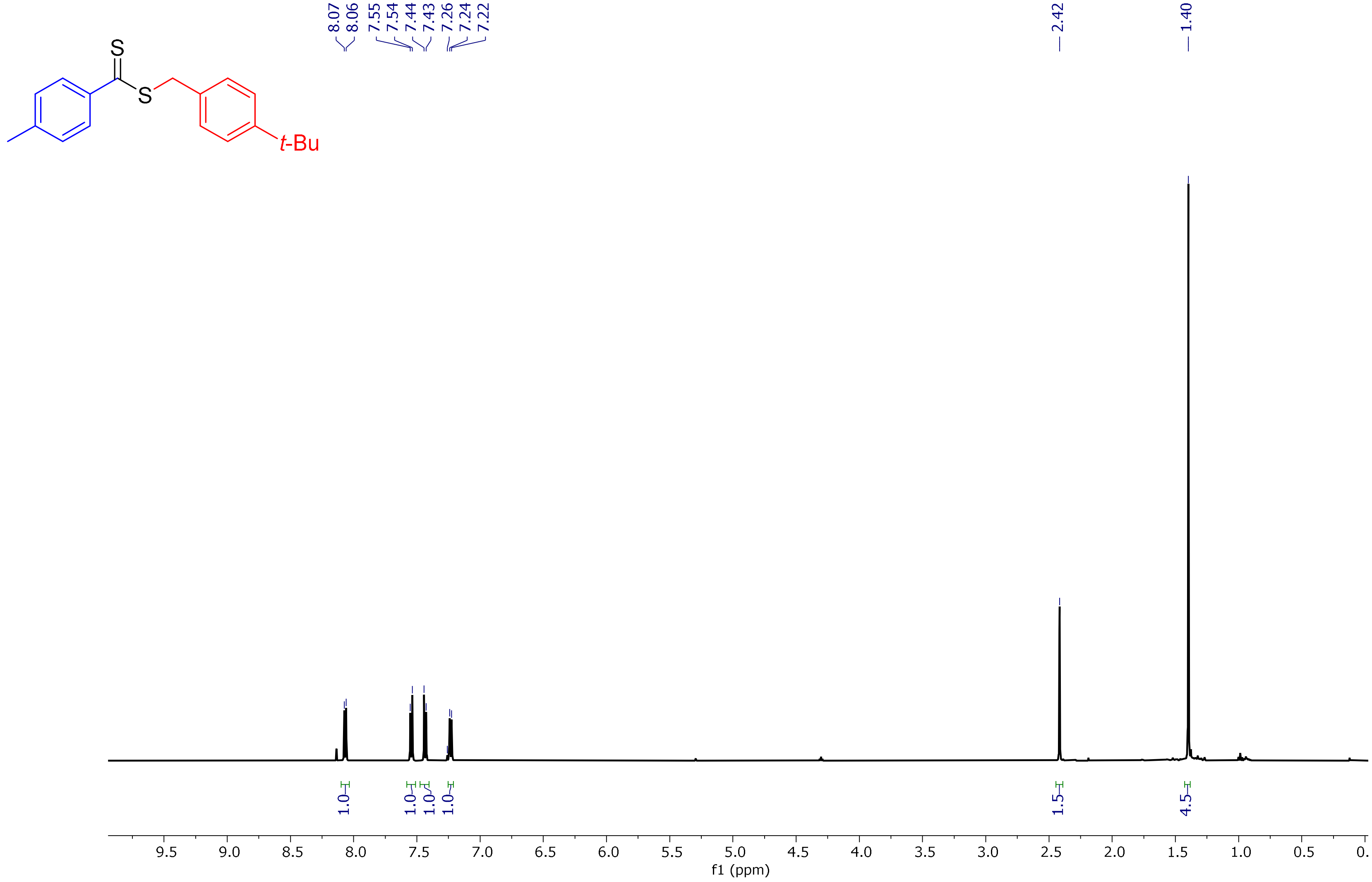

Figure S42. ^{13}C NMR (125 MHz, $CDCl_3$) spectrum for **10a**

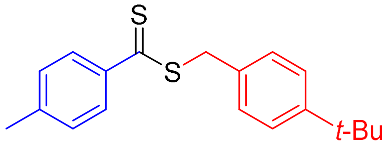

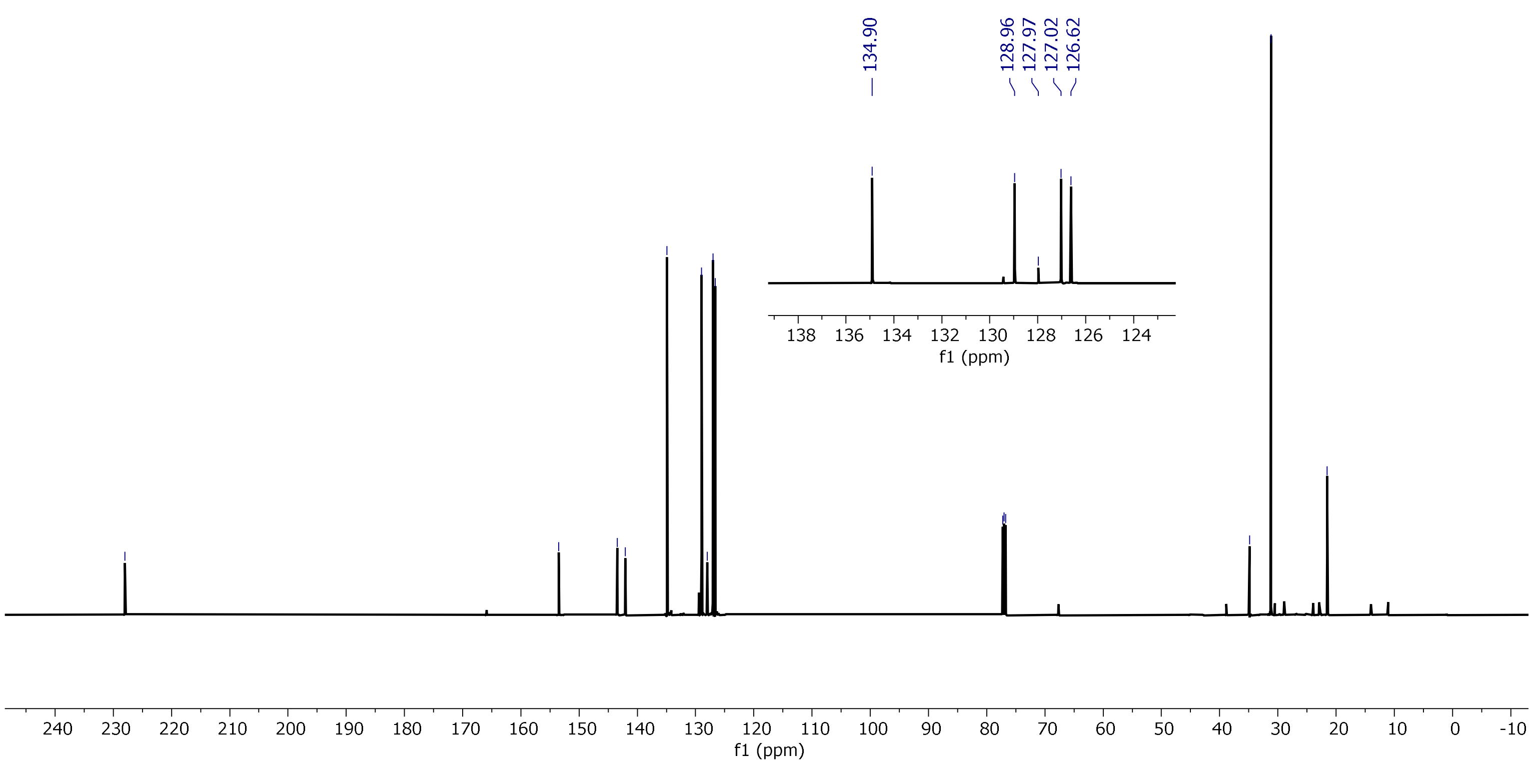

Figure S43. ^{1}H NMR (500 MHz, $CDCl_3$) spectrum for **11a**

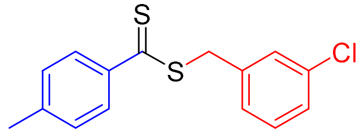

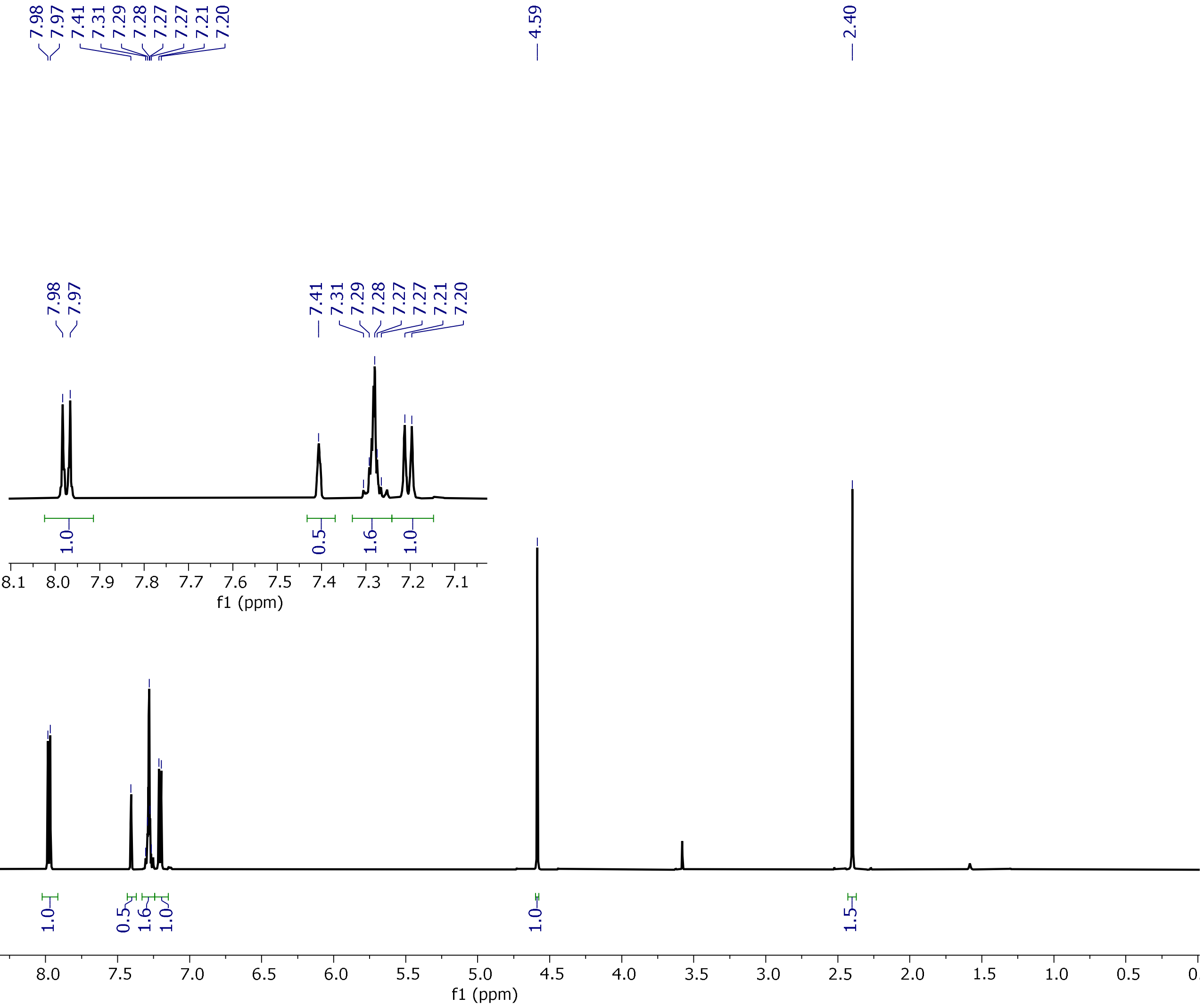

Figure S44. ^{13}C NMR (125 MHz, $CDCl_3$) spectrum for **11a**

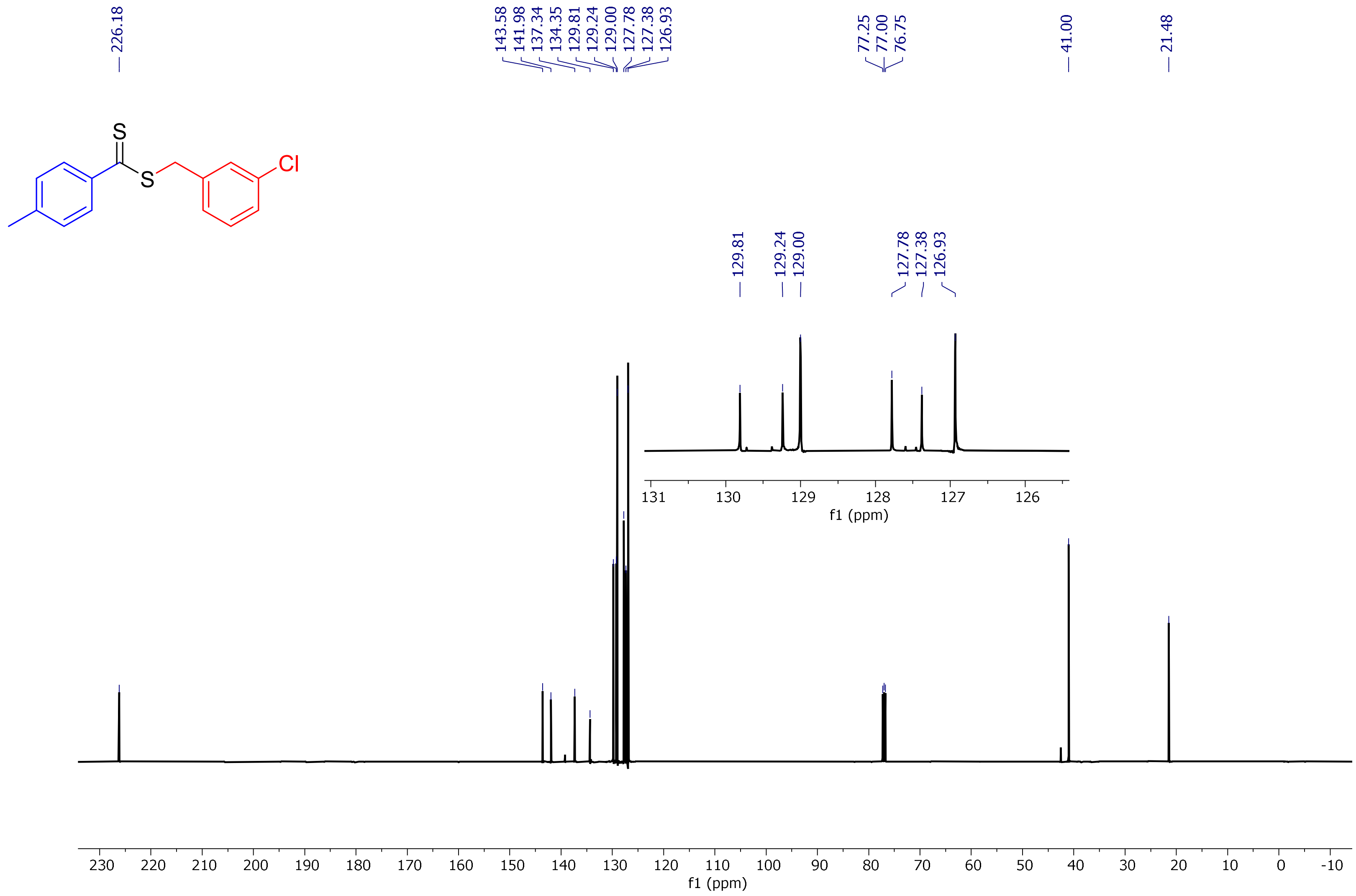

Figure S45. ^{1}H NMR (500 MHz, $CDCl_3$) spectrum for **12a**

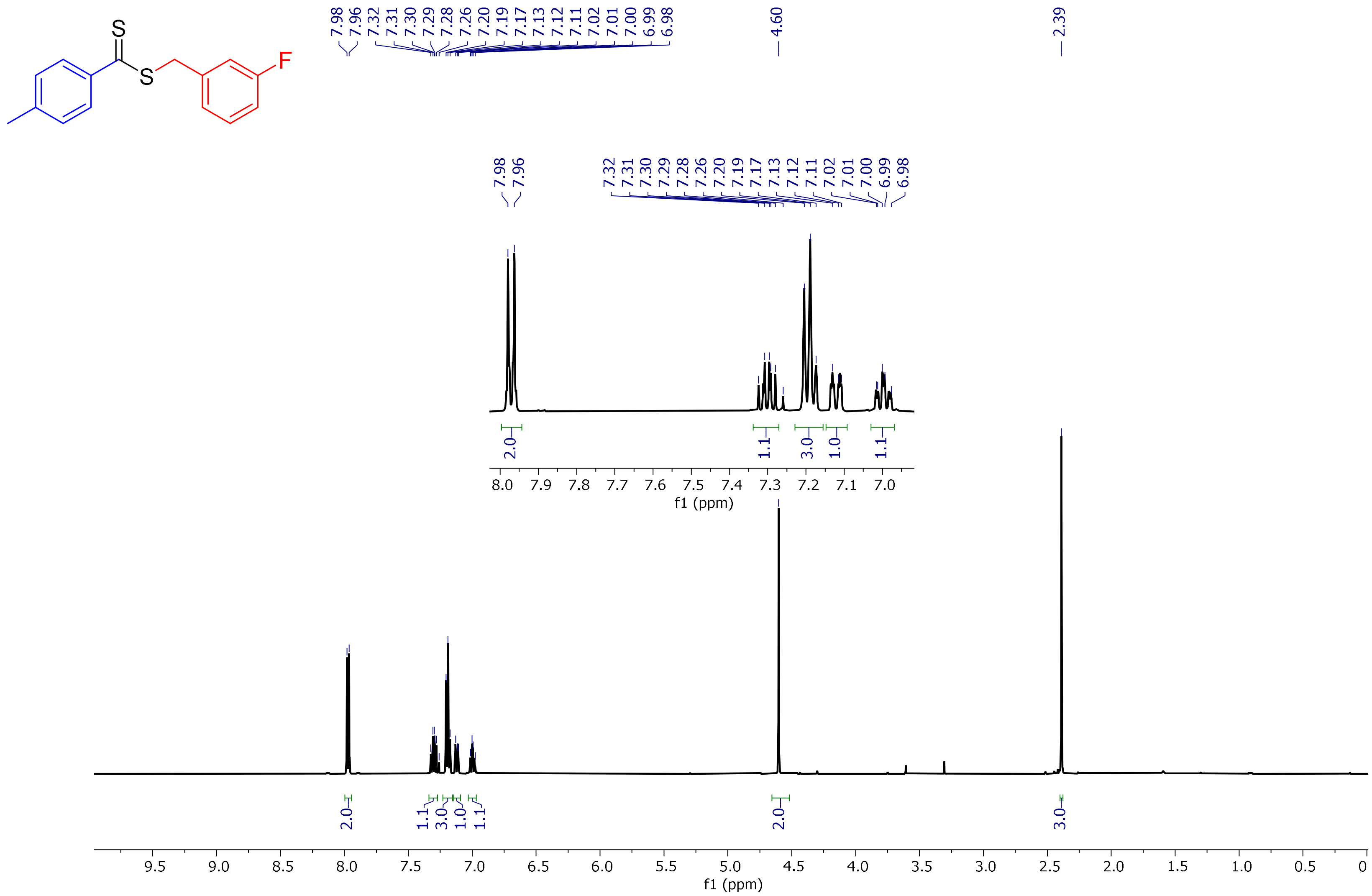

Figure S46. ^{13}C NMR (125 MHz, $CDCl_3$) spectrum for **12a**

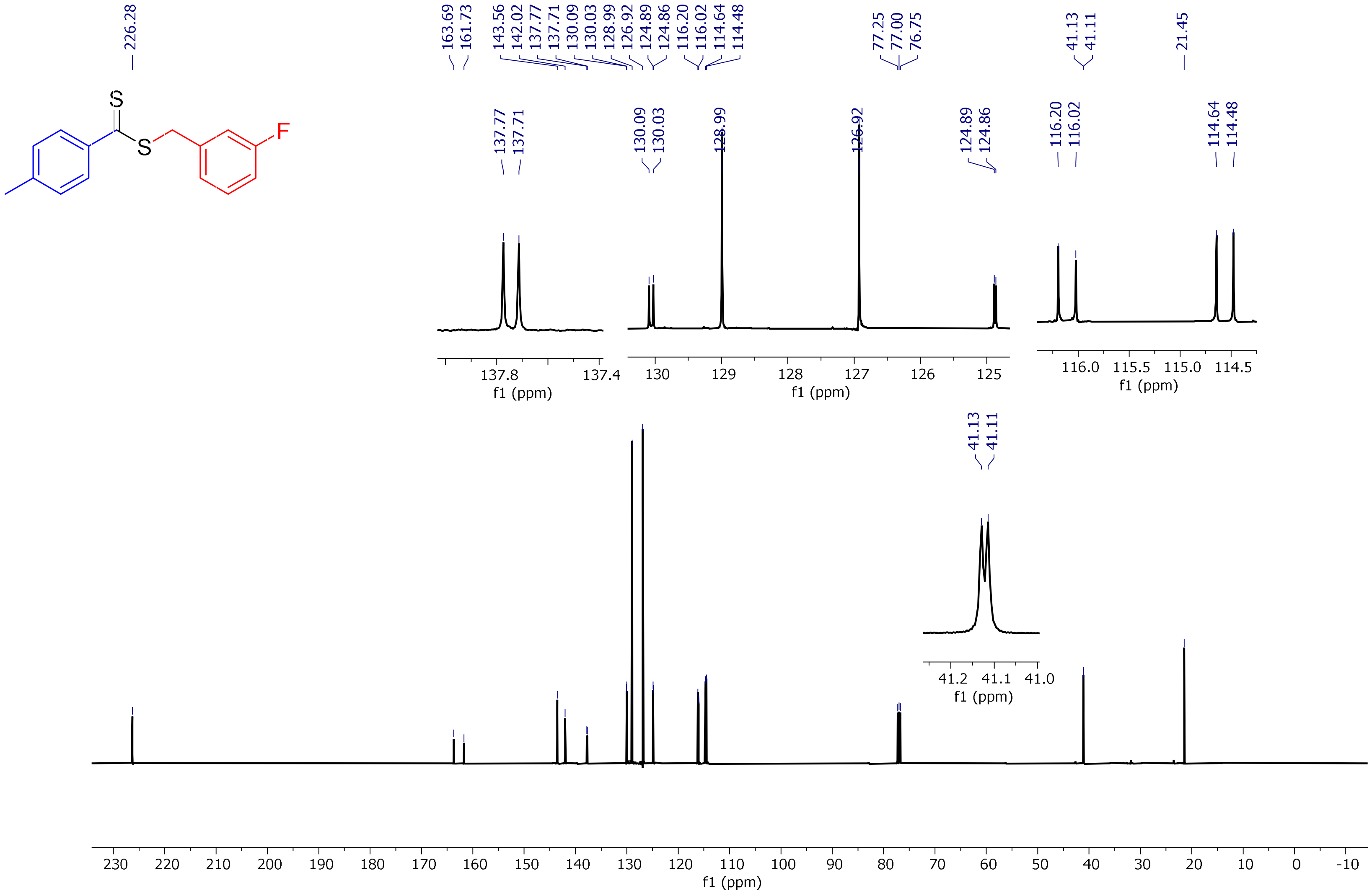

Figure S47. ^{1}H NMR (500 MHz, $CDCl_3$) spectrum for **13a**

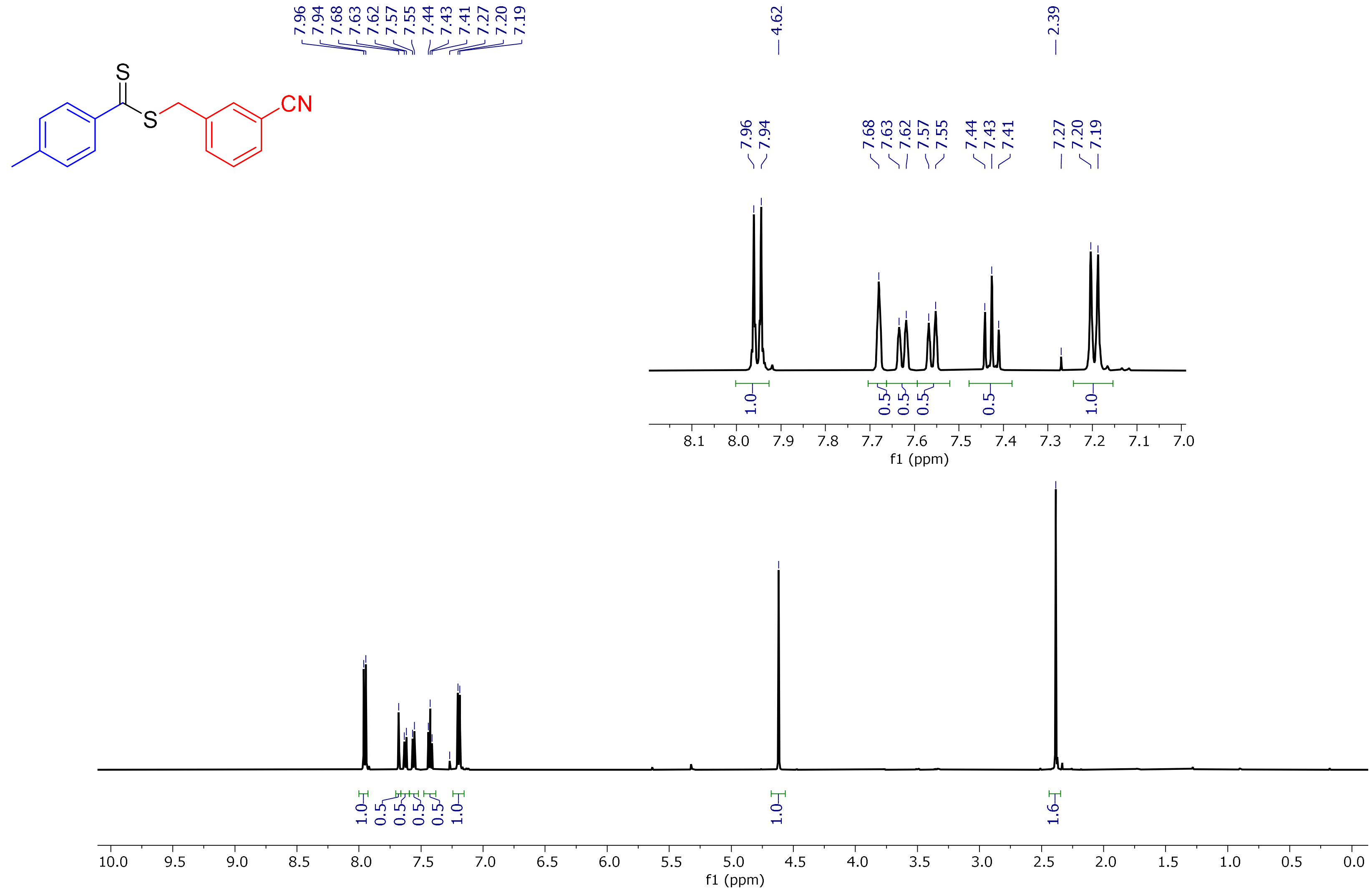

Figure S48. ^{13}C NMR (125 MHz, $CDCl_3$) spectrum for **13a**

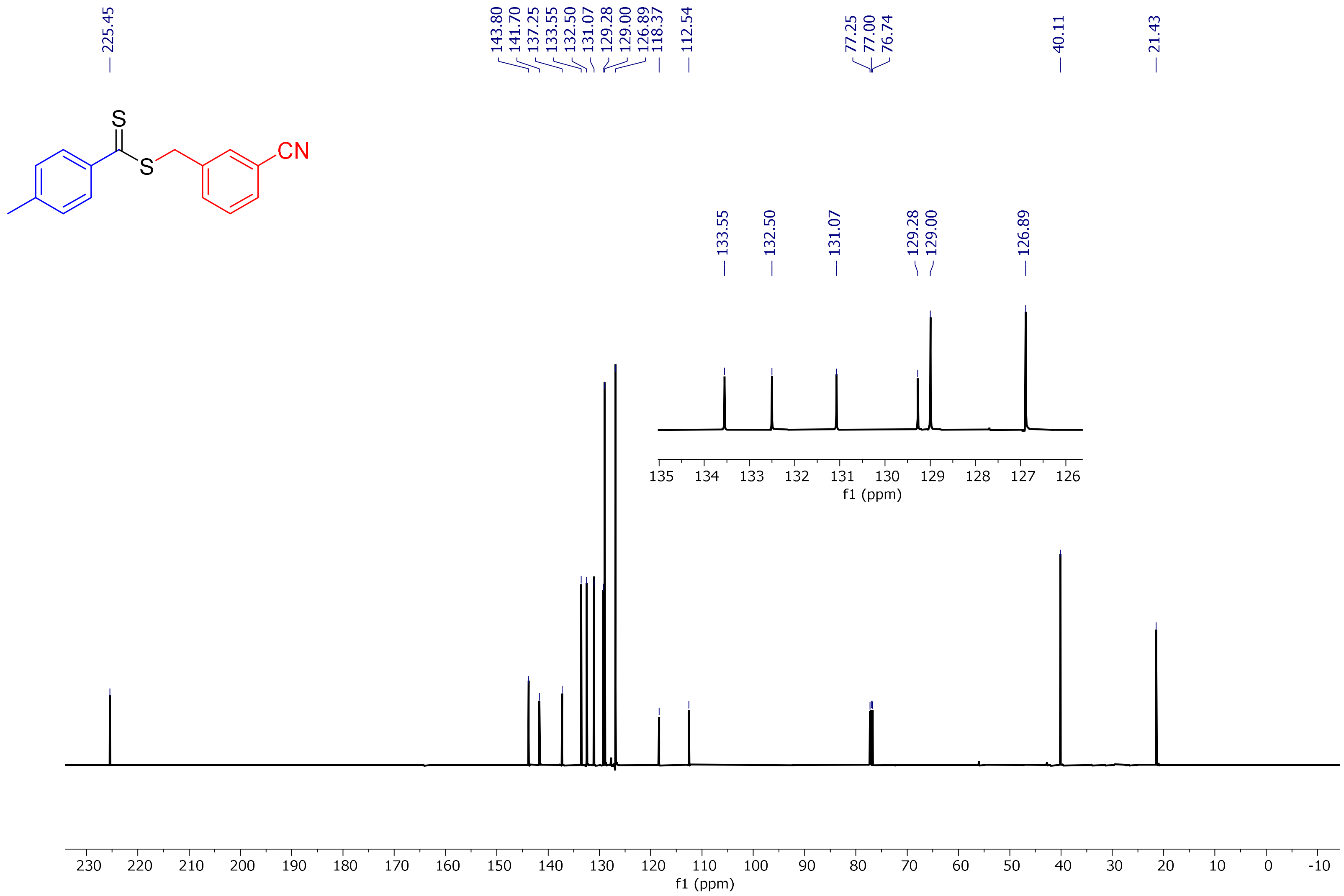

Figure S49. ^{1}H NMR (500 MHz, $CDCl_3$) spectrum for **14a**

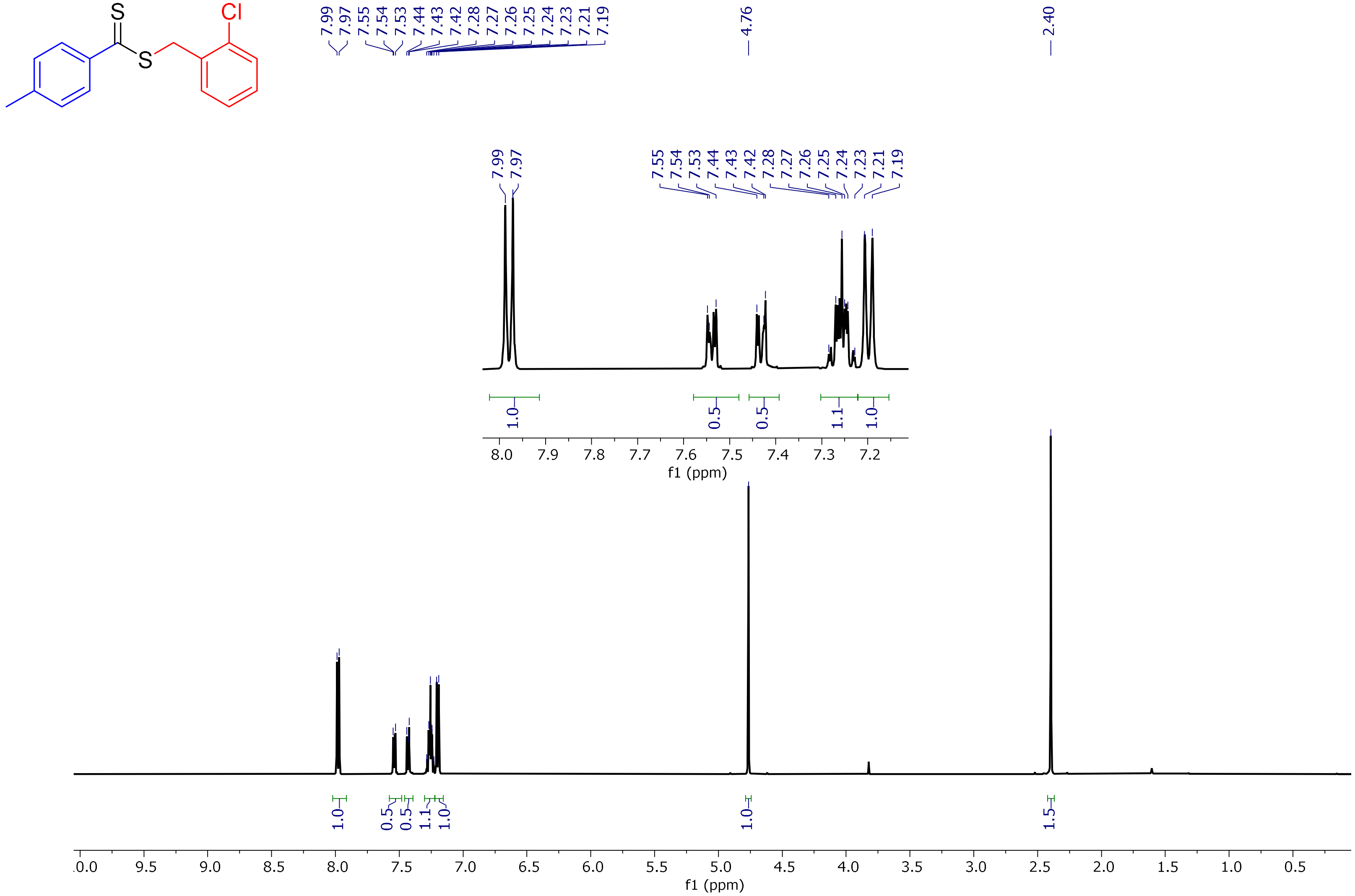

Figure S50. ^{13}C NMR (125 MHz, $CDCl_3$) spectrum for **14a**

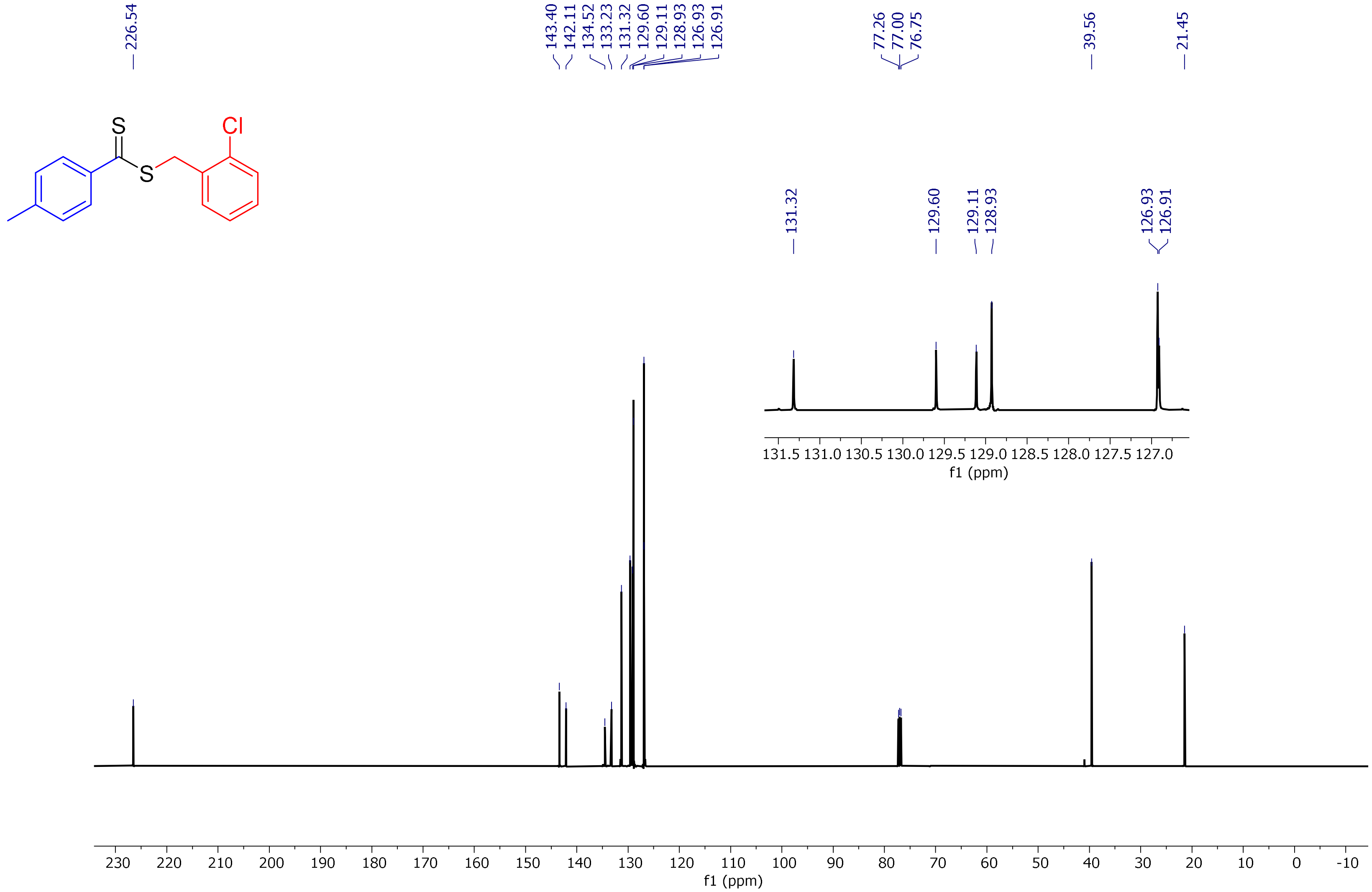

Figure S51. ^{1}H NMR (500 MHz, $CDCl_3$) spectrum for **15a**

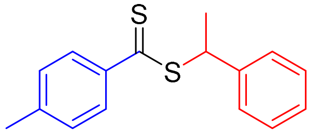

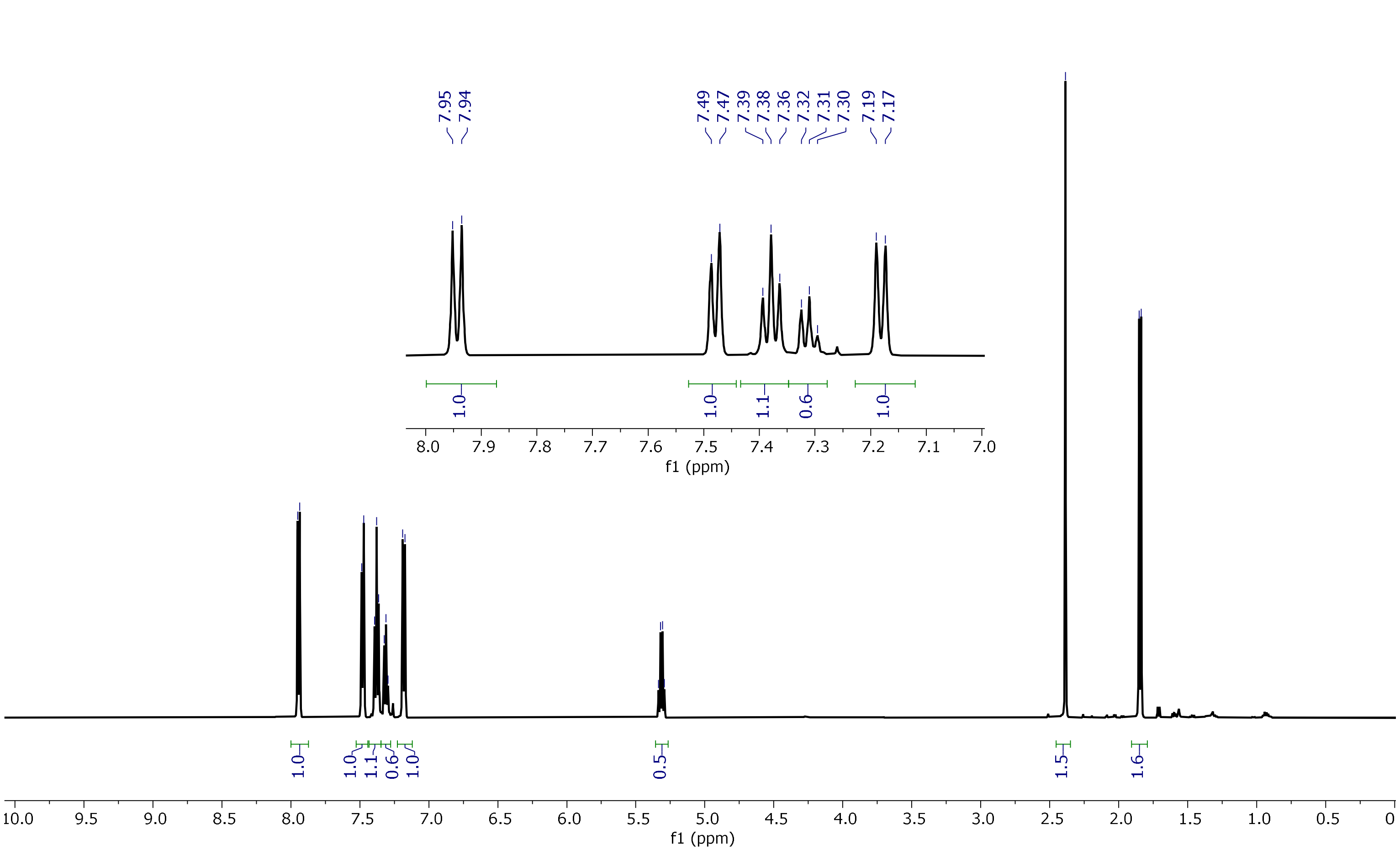

Figure S52. ^{13}C NMR (125 MHz, $CDCl_3$) spectrum for **15a**

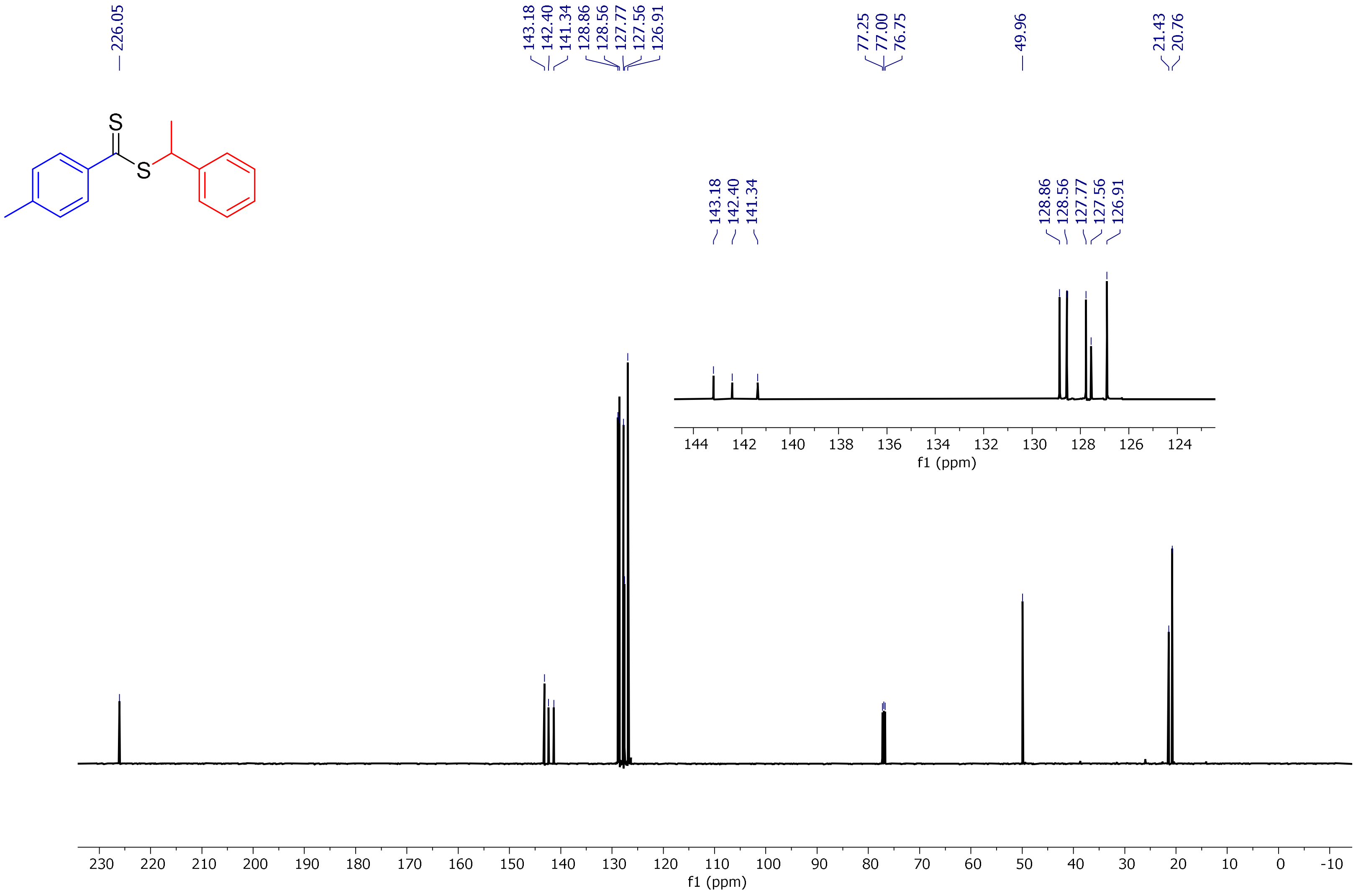

Figure S53. ^{1}H NMR (500 MHz, $CDCl_3$) spectrum for **16a**

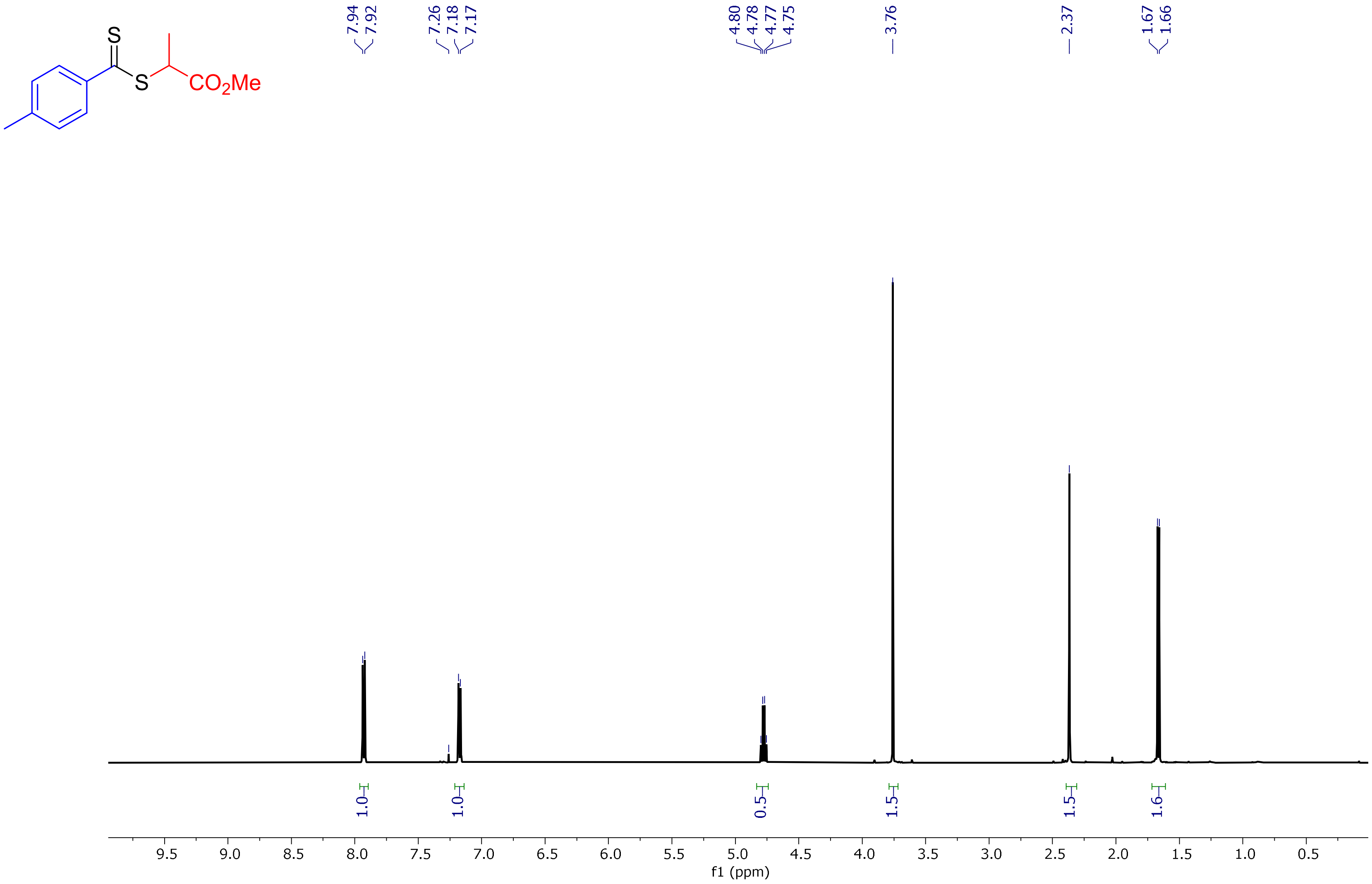

Figure S54. ^{13}C NMR (125 MHz, $CDCl_3$) spectrum for **16a**

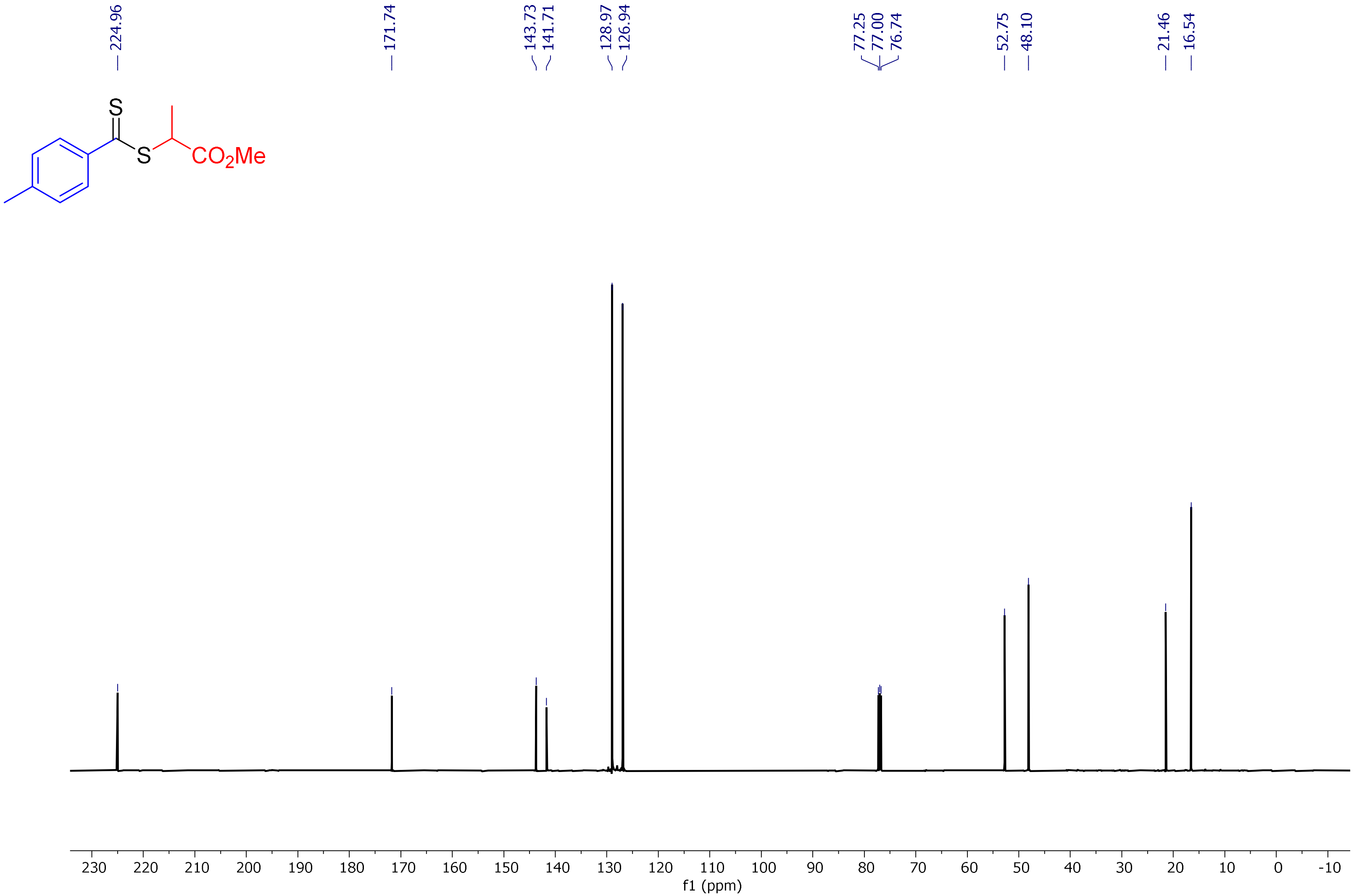

Figure S55. ^{1}H NMR (500 MHz, $CDCl_3$) spectrum for **17a**

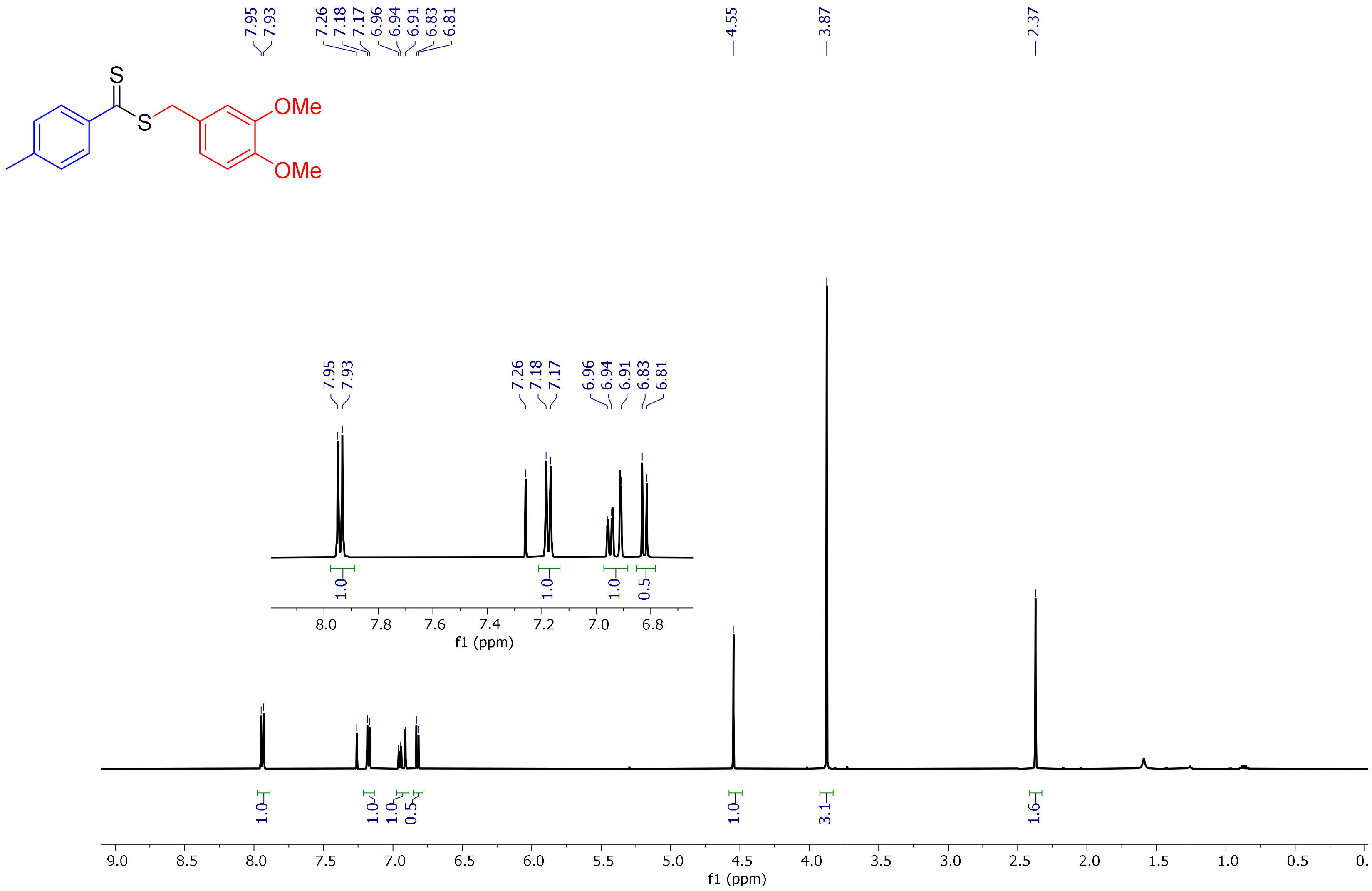

Figure S56. ^{13}C NMR (125 MHz, $CDCl_3$) spectrum for **17a**

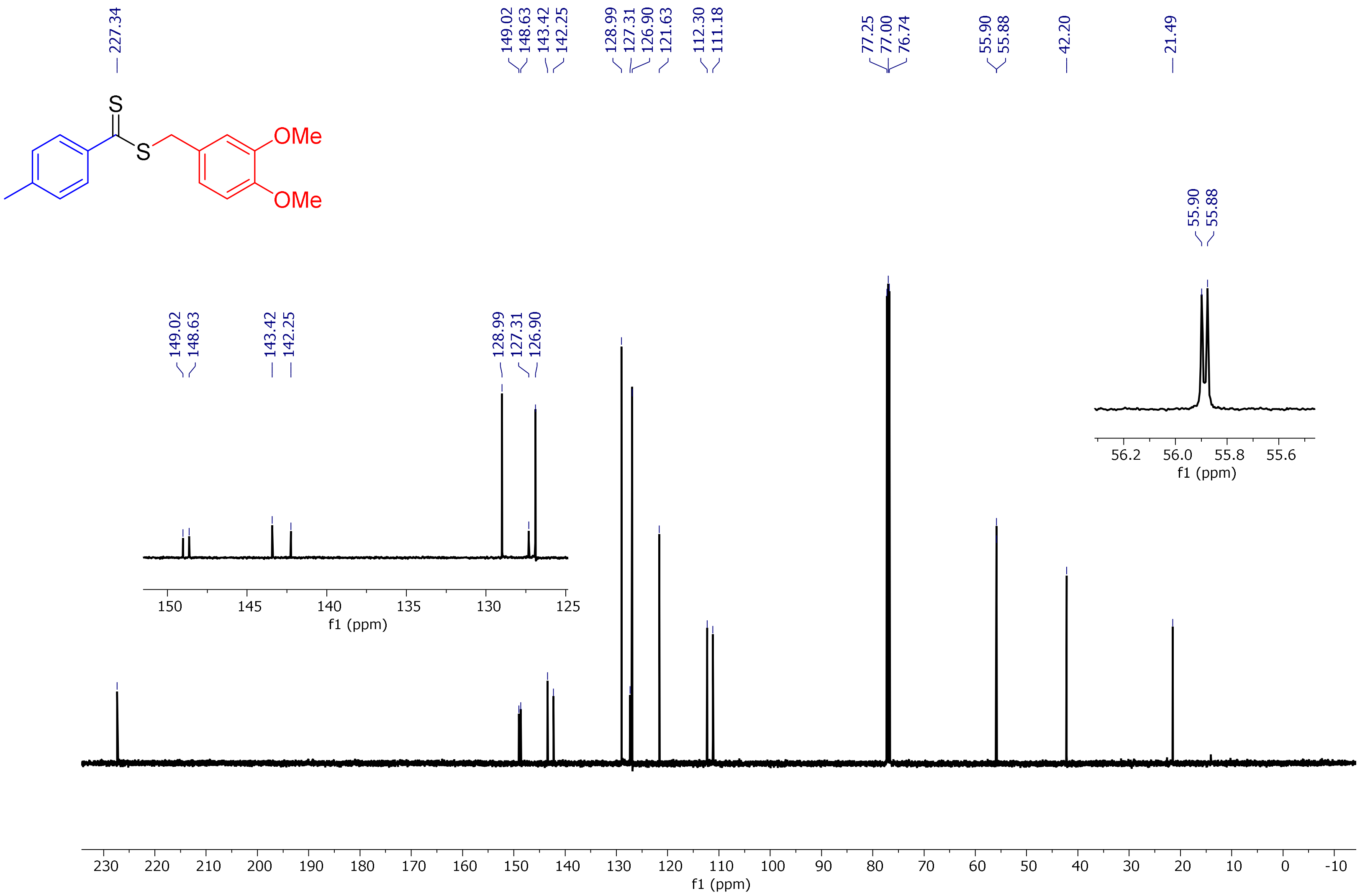

Figure S57. ^{1}H NMR (500 MHz, $CDCl_3$) spectrum for **18a**

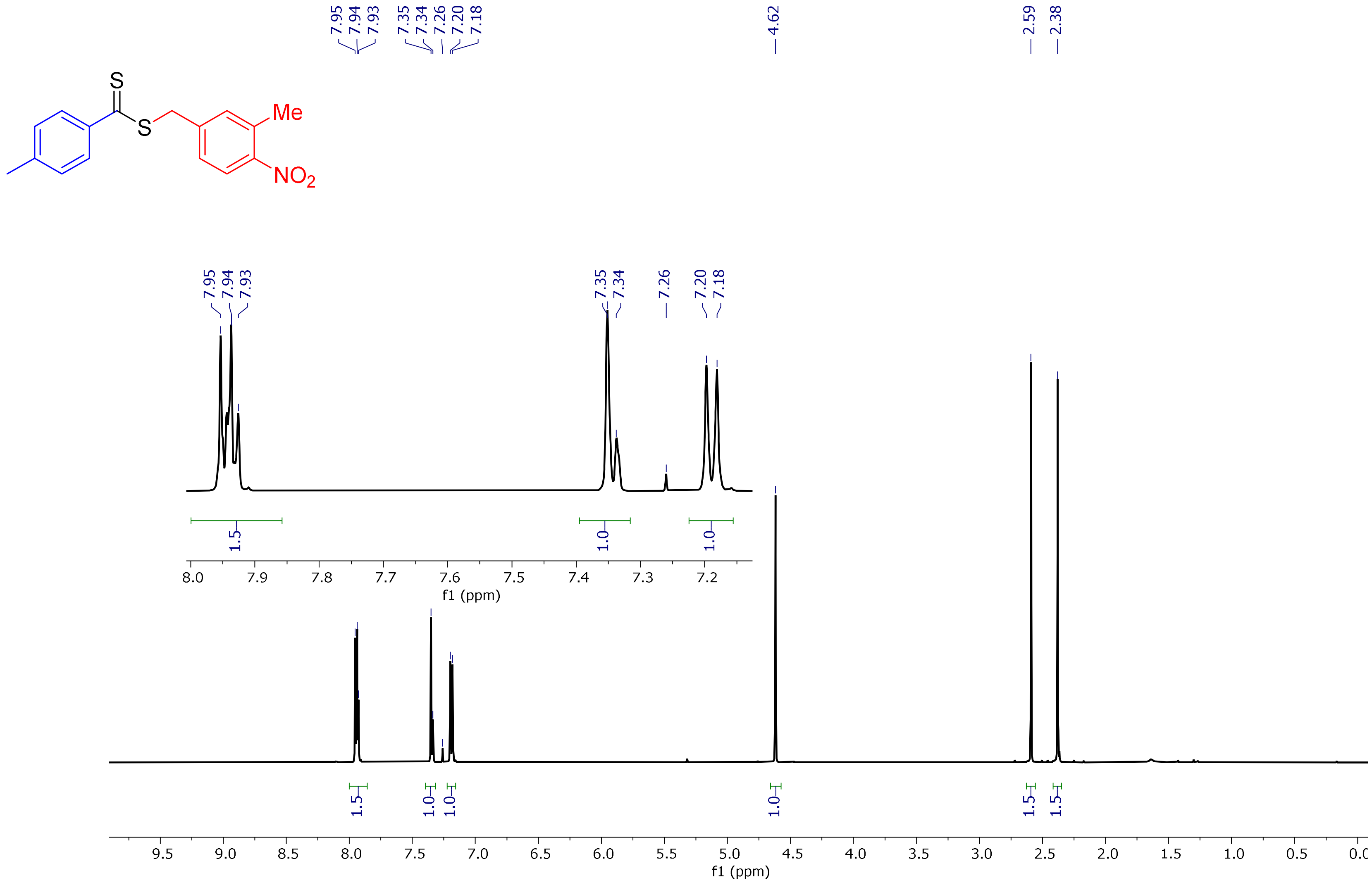

Figure S58. ^{13}C NMR (125 MHz, $CDCl_3$) spectrum for **18a**

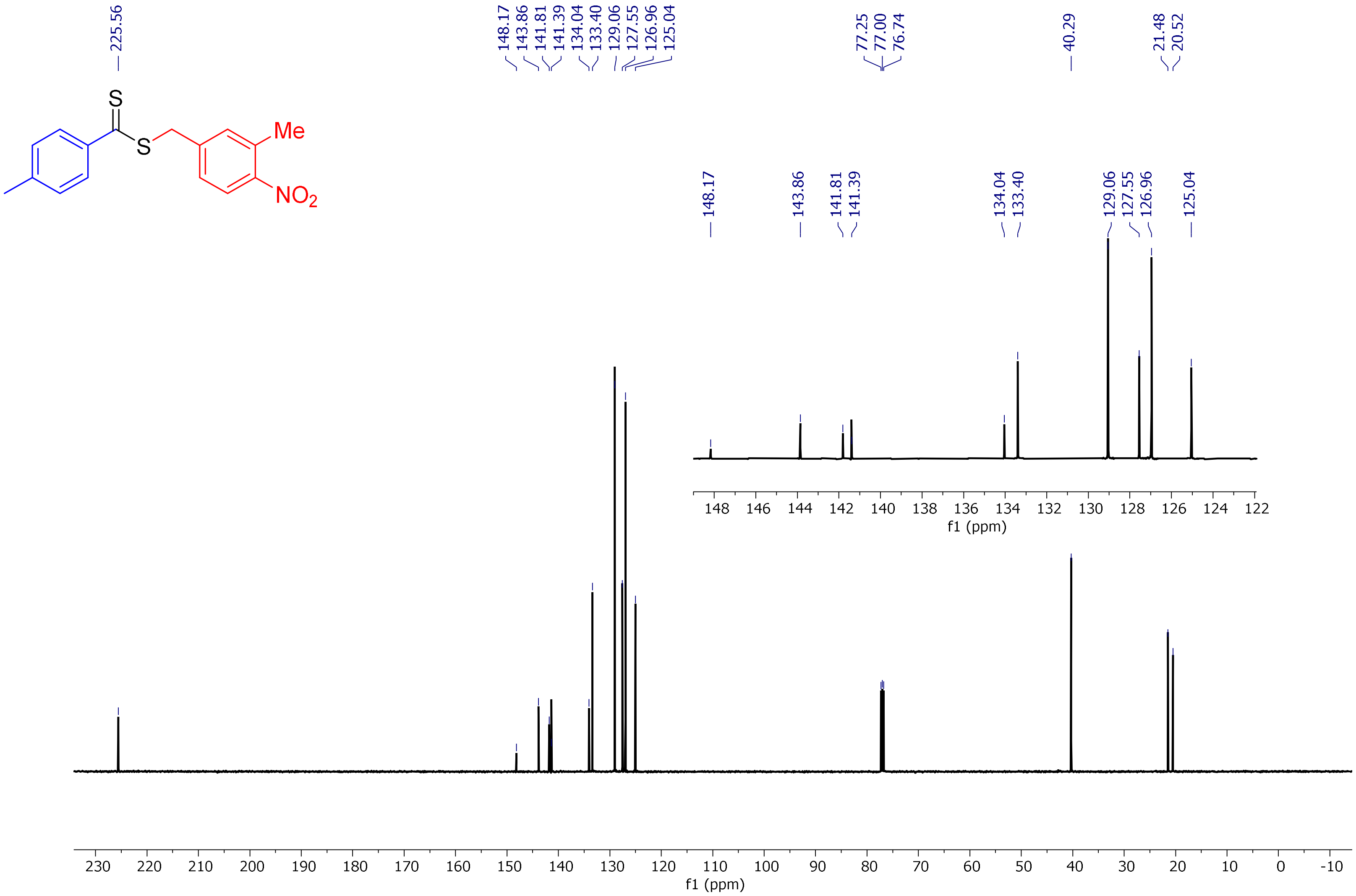

Figure S59. ^{1}H NMR (500 MHz, $CDCl_3$) spectrum for **19a**

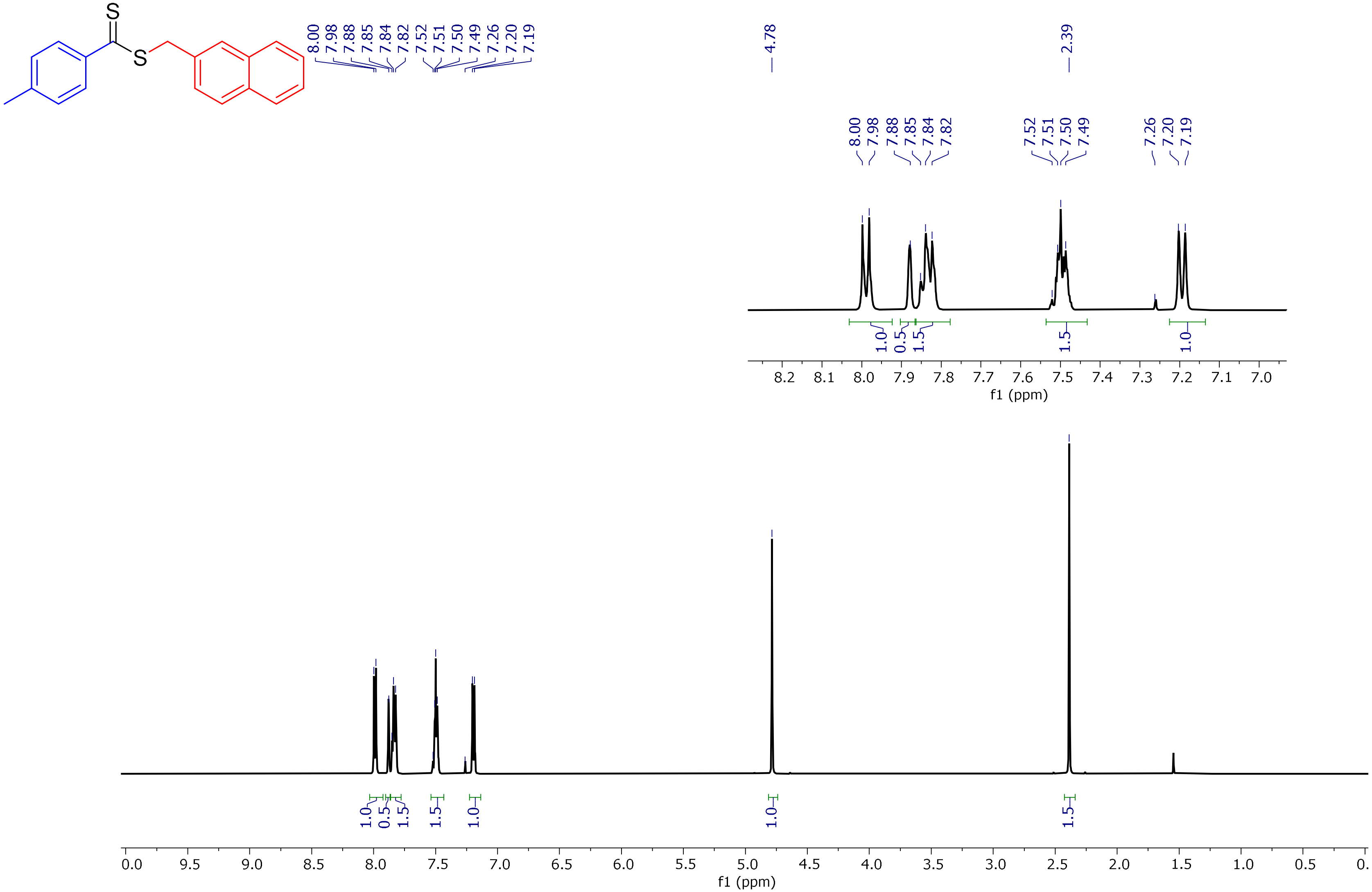

Figure S60. ^{13}C NMR (125 MHz, $CDCl_3$) spectrum for **19a**

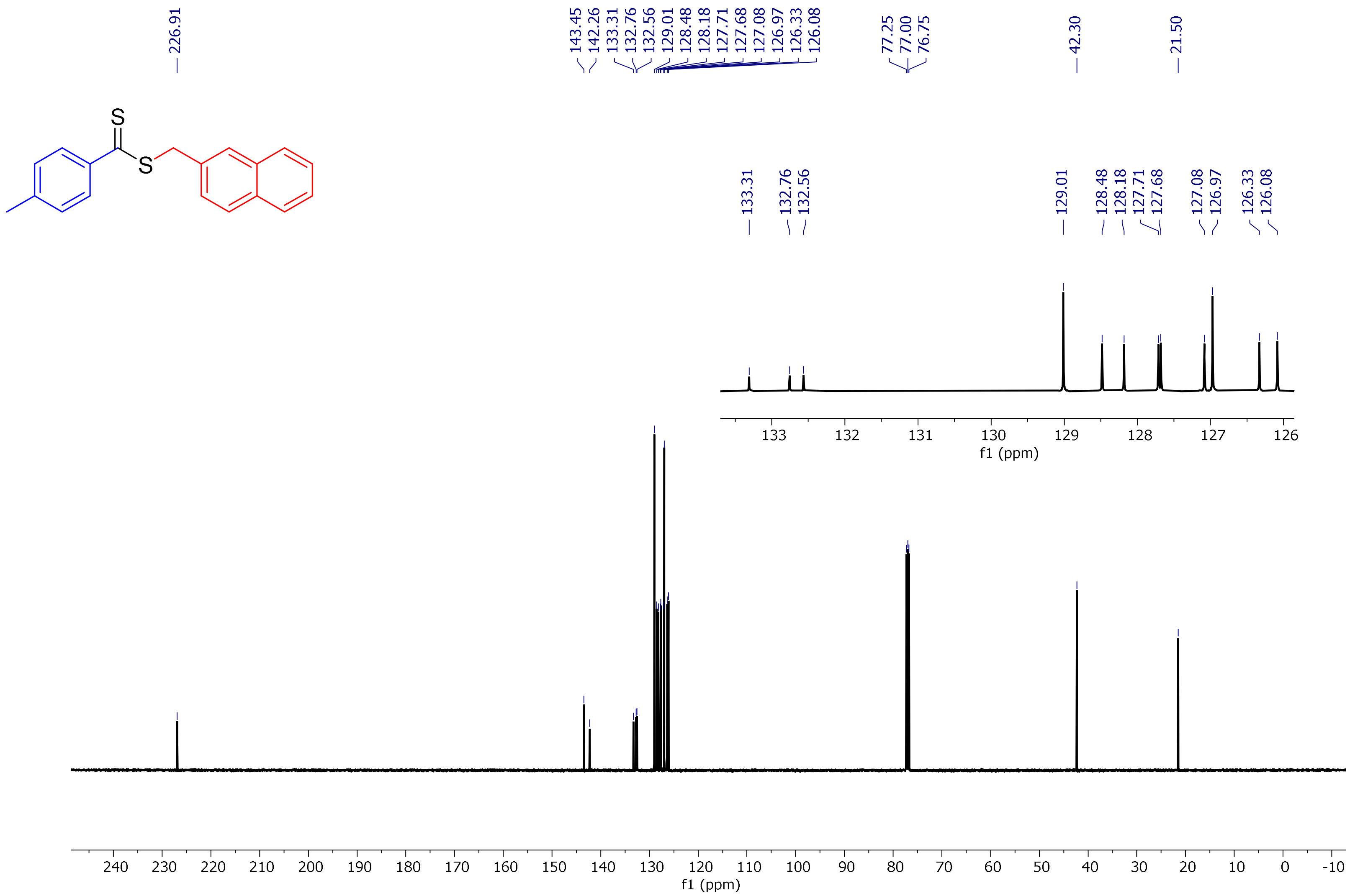

Figure S61. ^{1}H NMR (500 MHz, $CDCl_3$) spectrum for **20a**

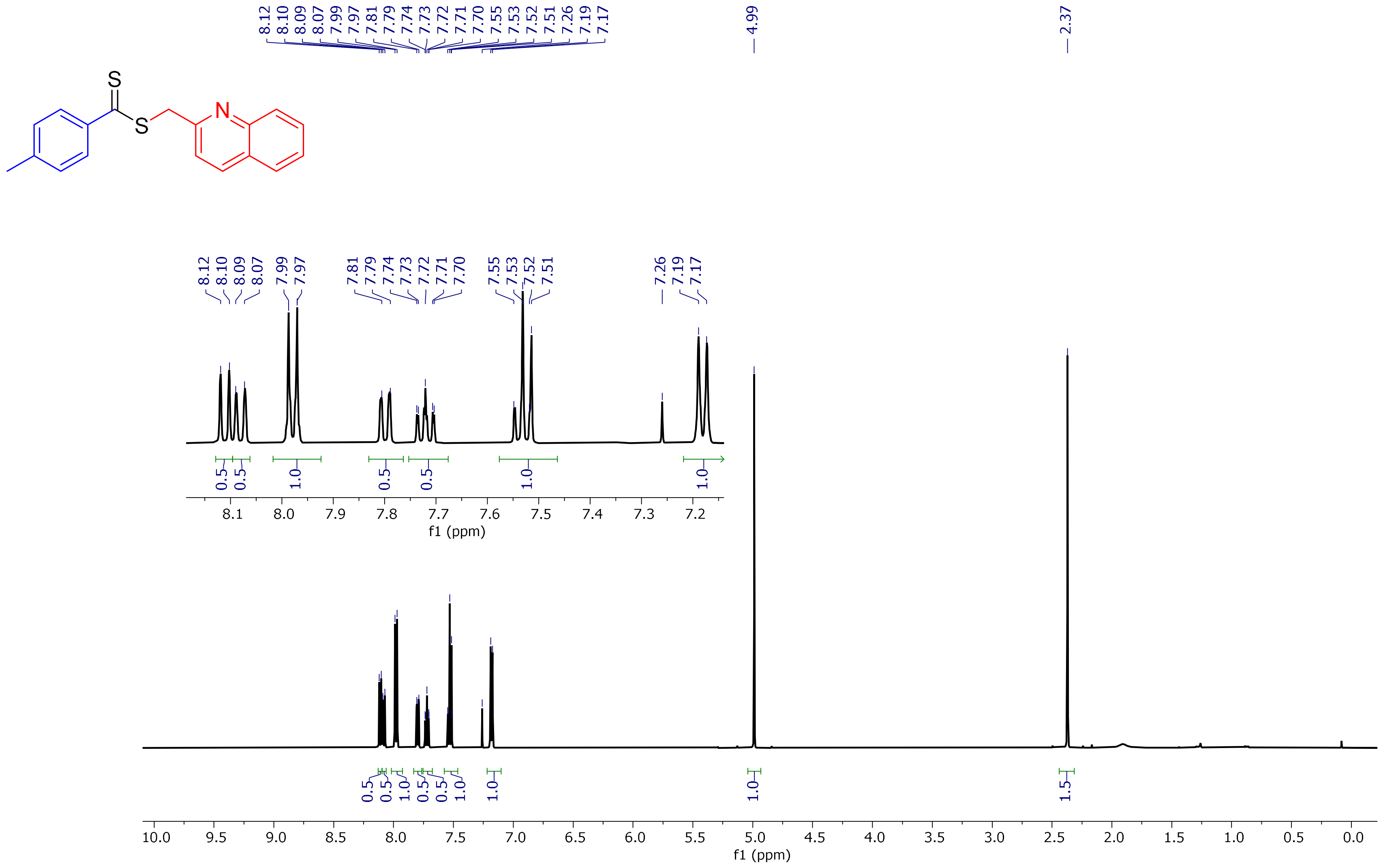

Figure S62. ^{13}C NMR (125 MHz, $CDCl_3$) spectrum for **20a**

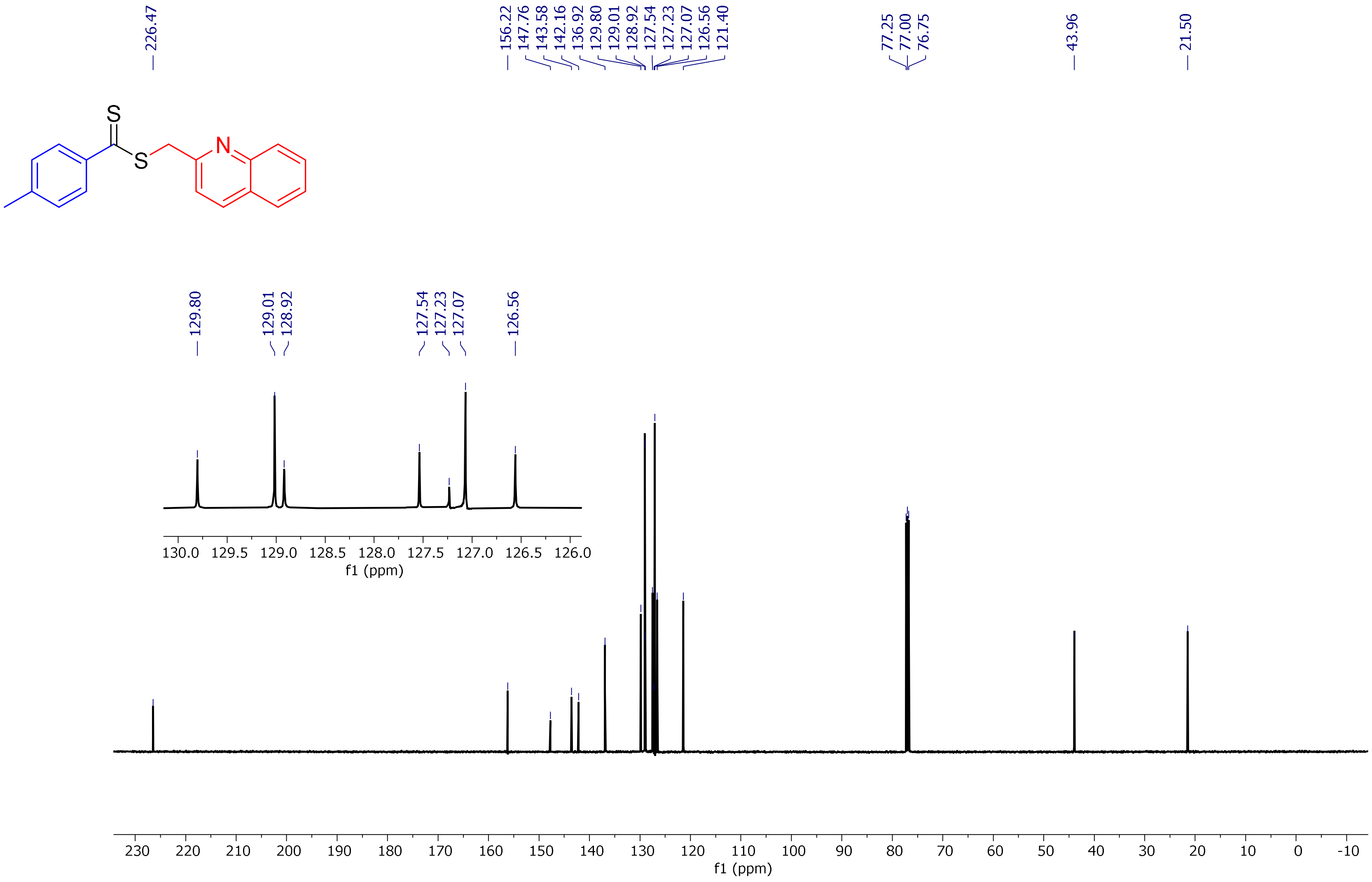

Figure S63. ^{1}H NMR (500 MHz, $CDCl_3$) spectrum for **21a**

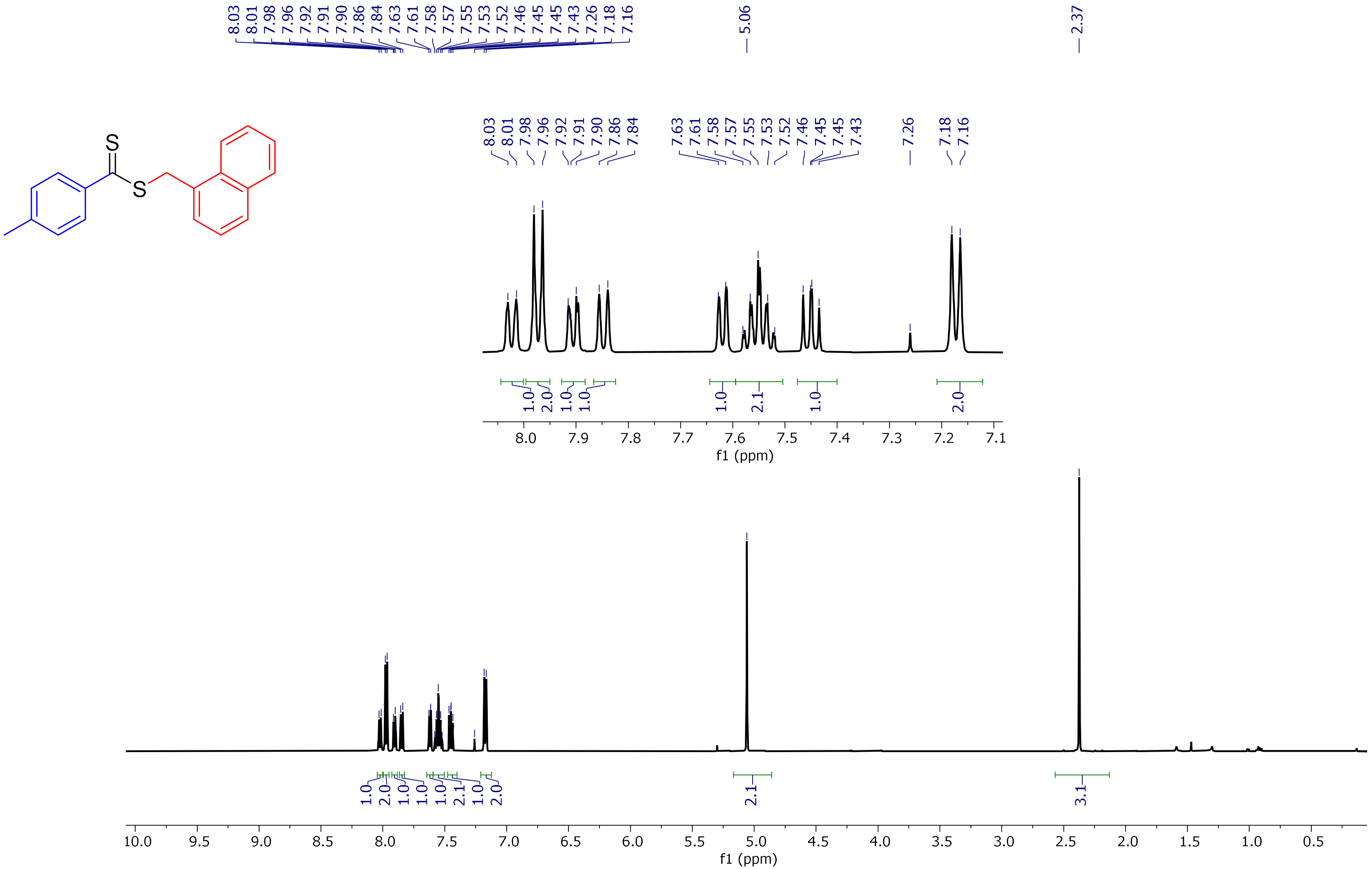

Figure S64. ^{13}C NMR (125 MHz, $CDCl_3$) spectrum for **21a**

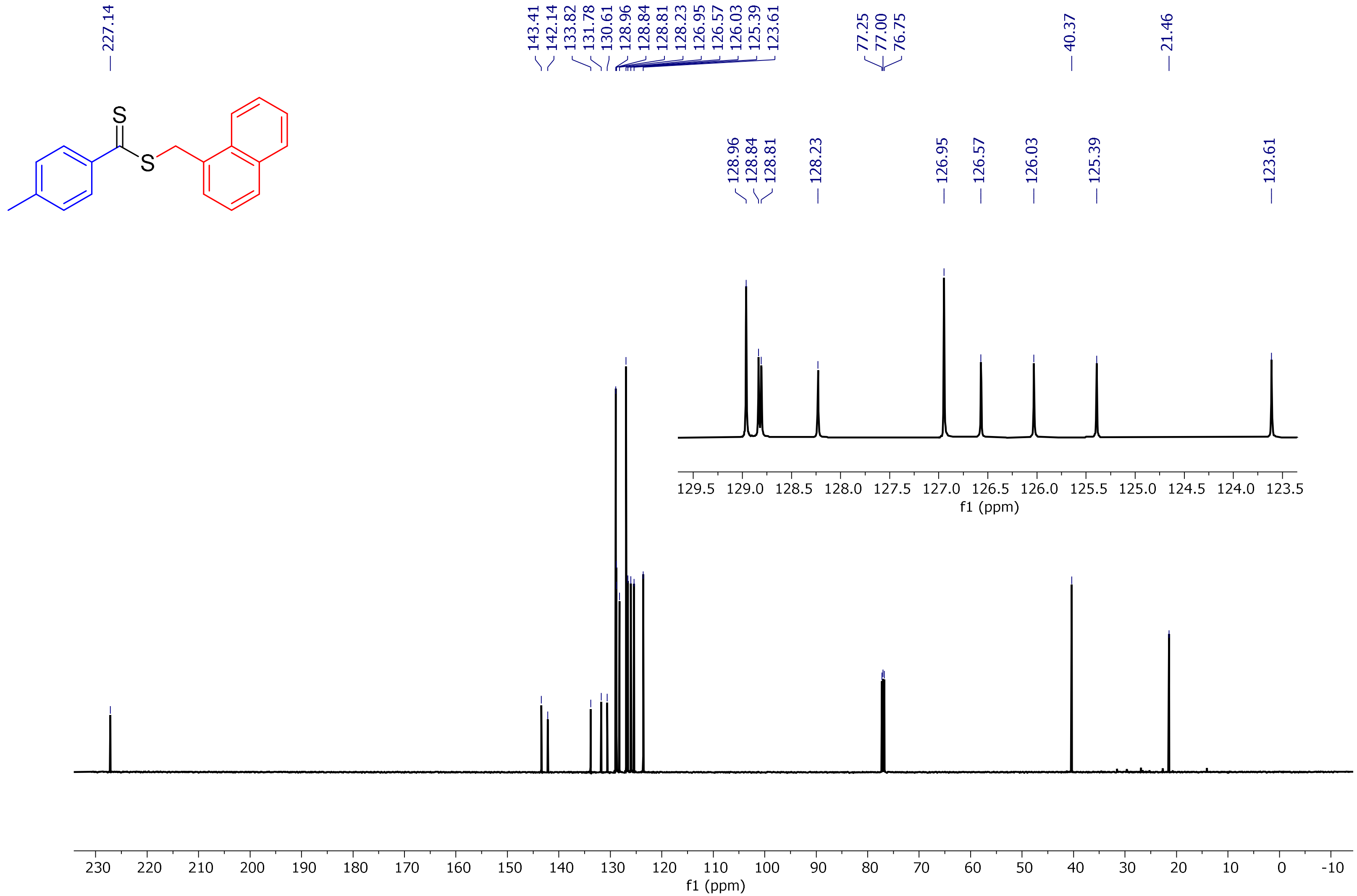

Figure S65. ^{1}H NMR (500 MHz, $CDCl_3$) spectrum for **22a**

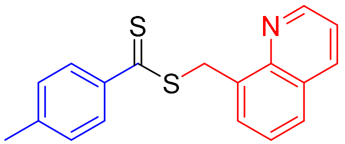

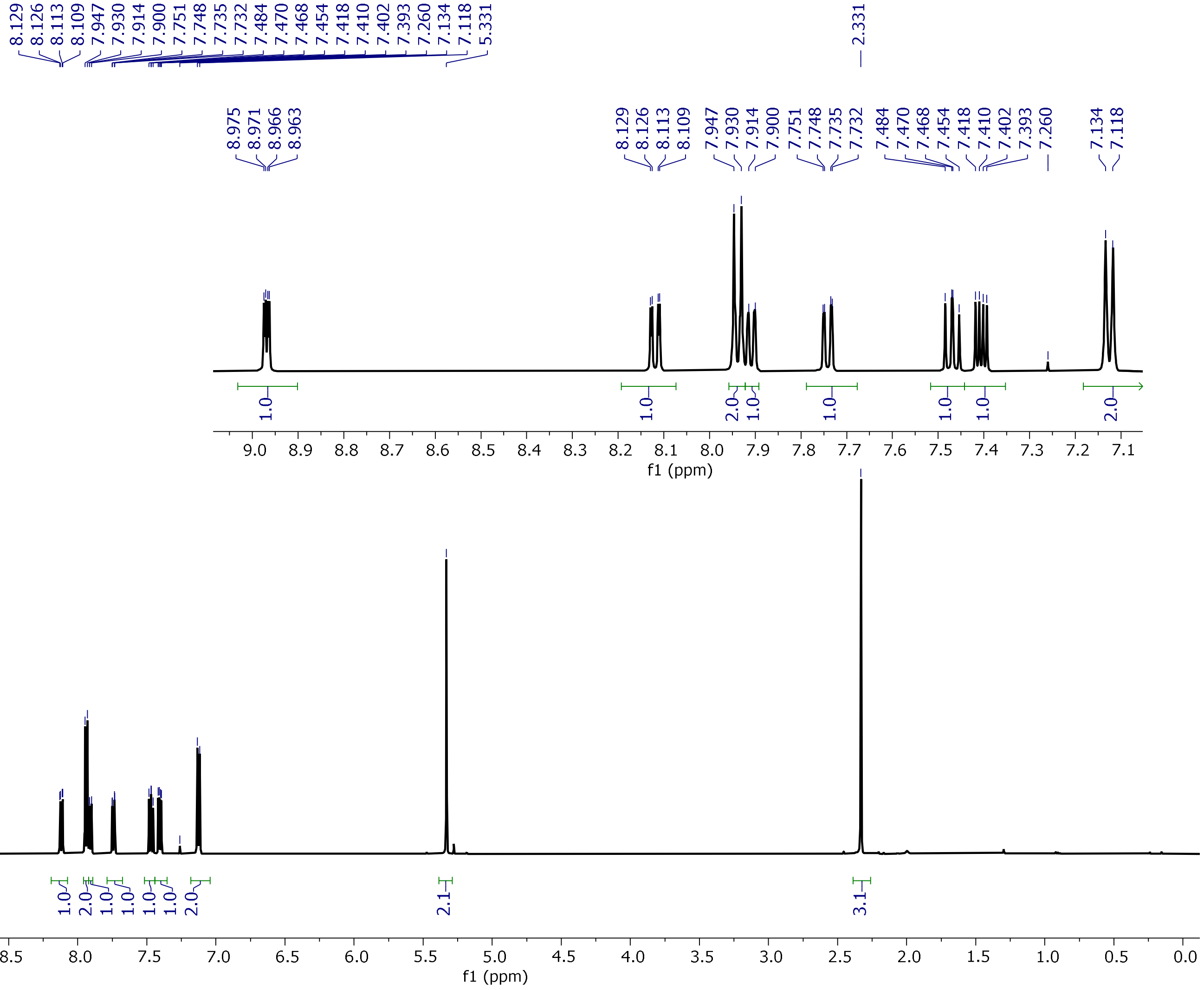

Figure S66. ^{13}C NMR (125 MHz, $CDCl_3$) spectrum for **22a**

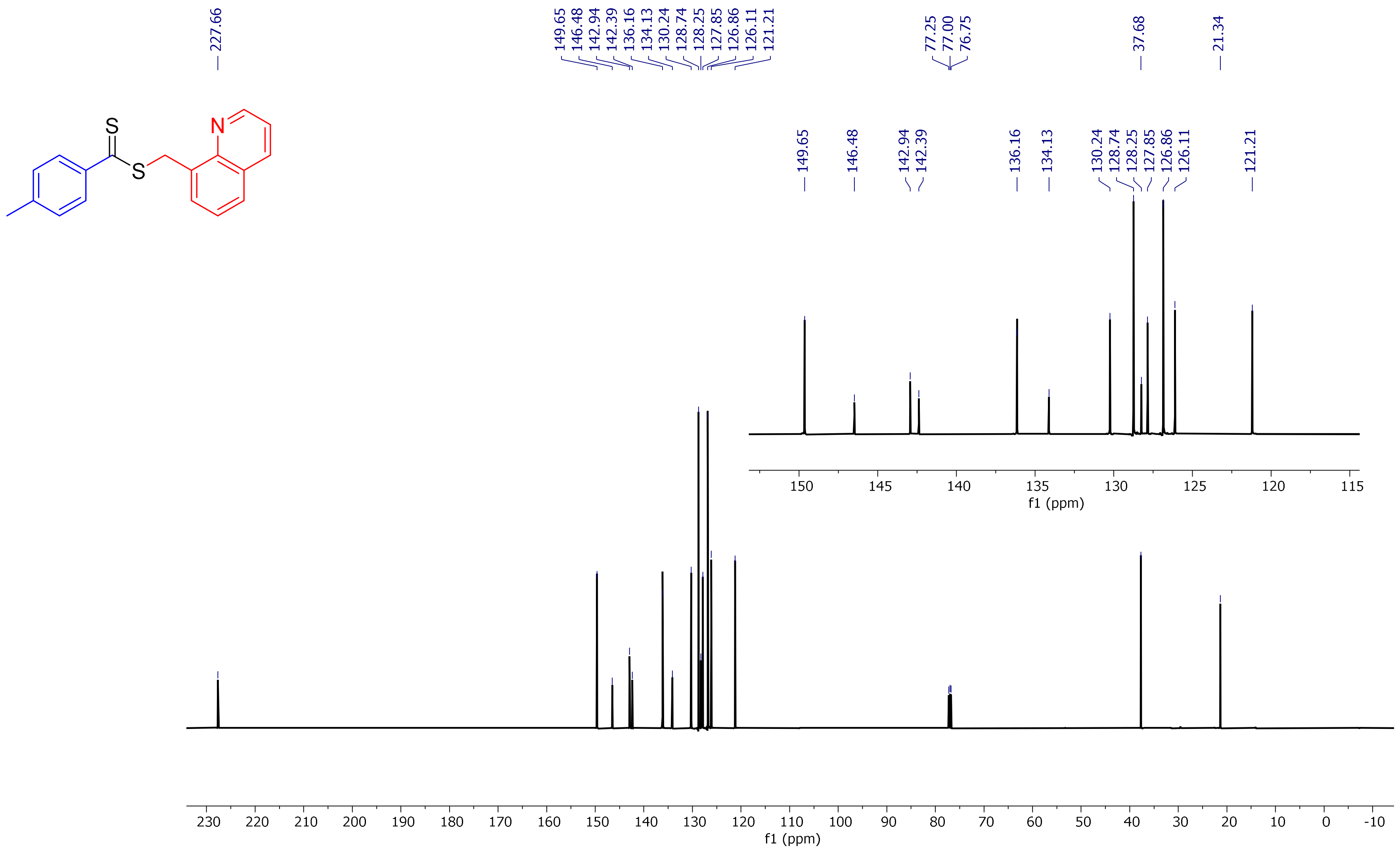

Figure S67. ^{1}H NMR (500 MHz, $CDCl_3$) spectrum for **23a**

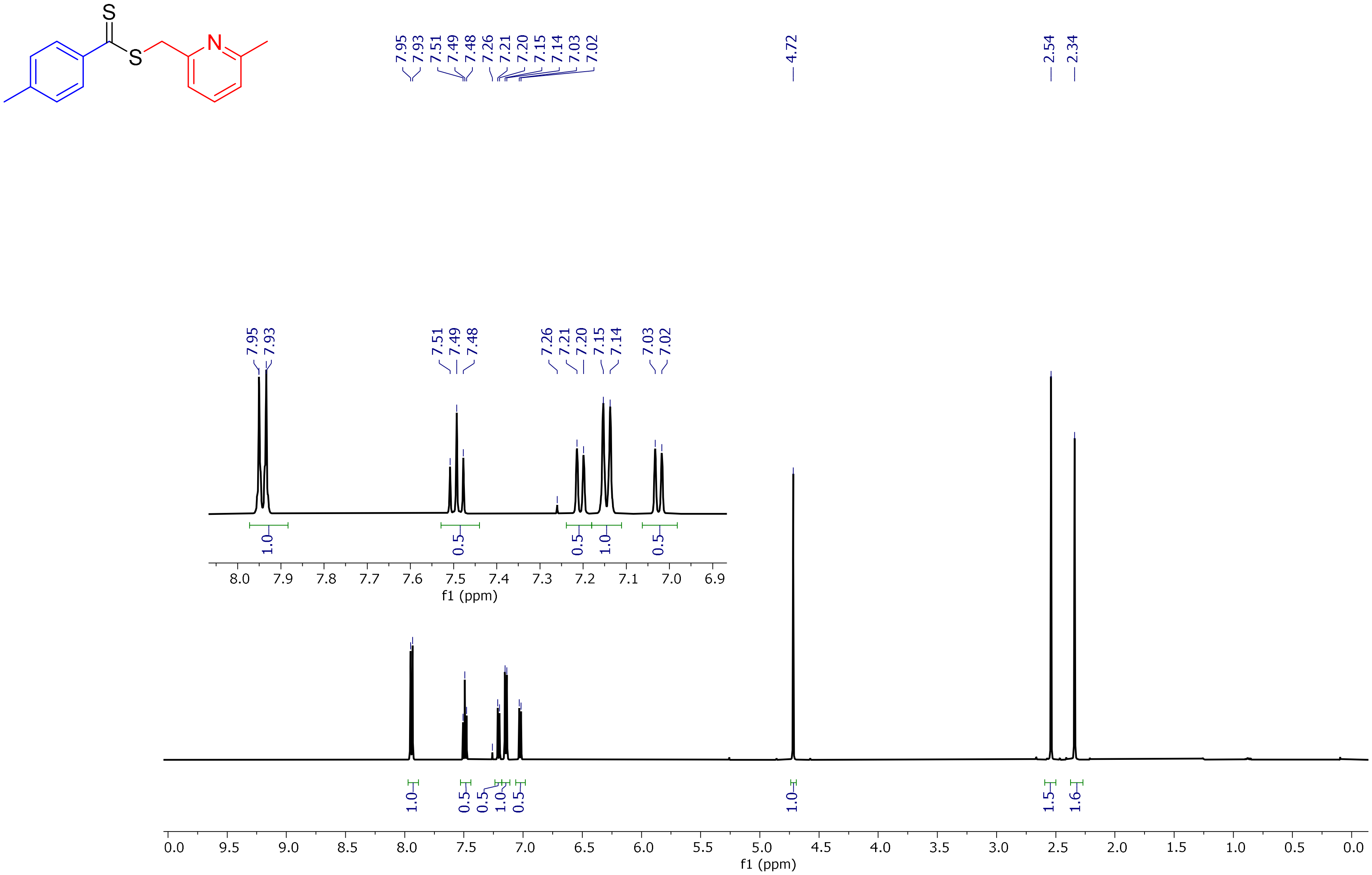

Figure S68. ^{13}C NMR (125 MHz, $CDCl_3$) spectrum for **23a**

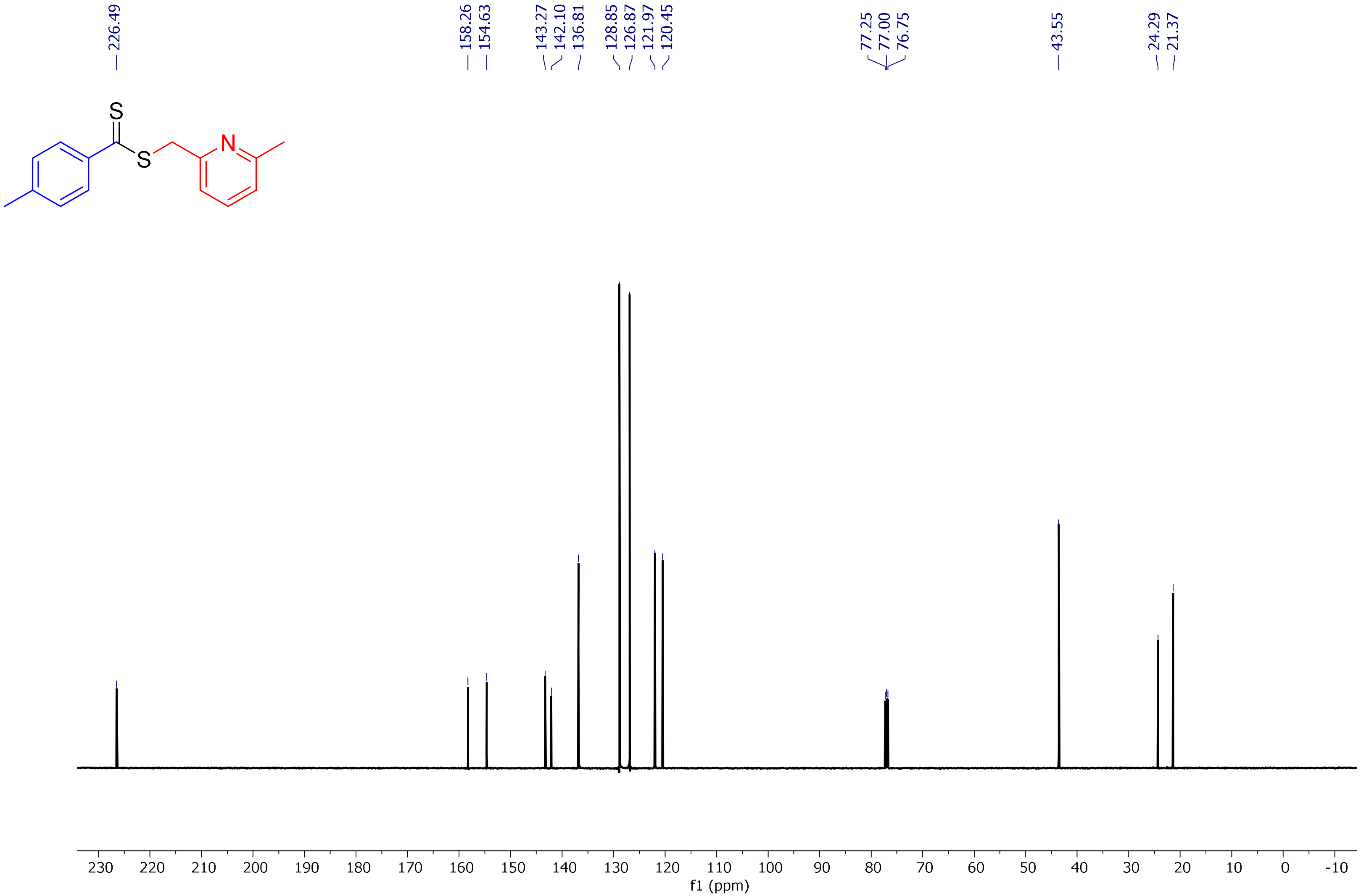

Figure S69. ^{1}H NMR (500 MHz, $CDCl_3$) spectrum for **24a**

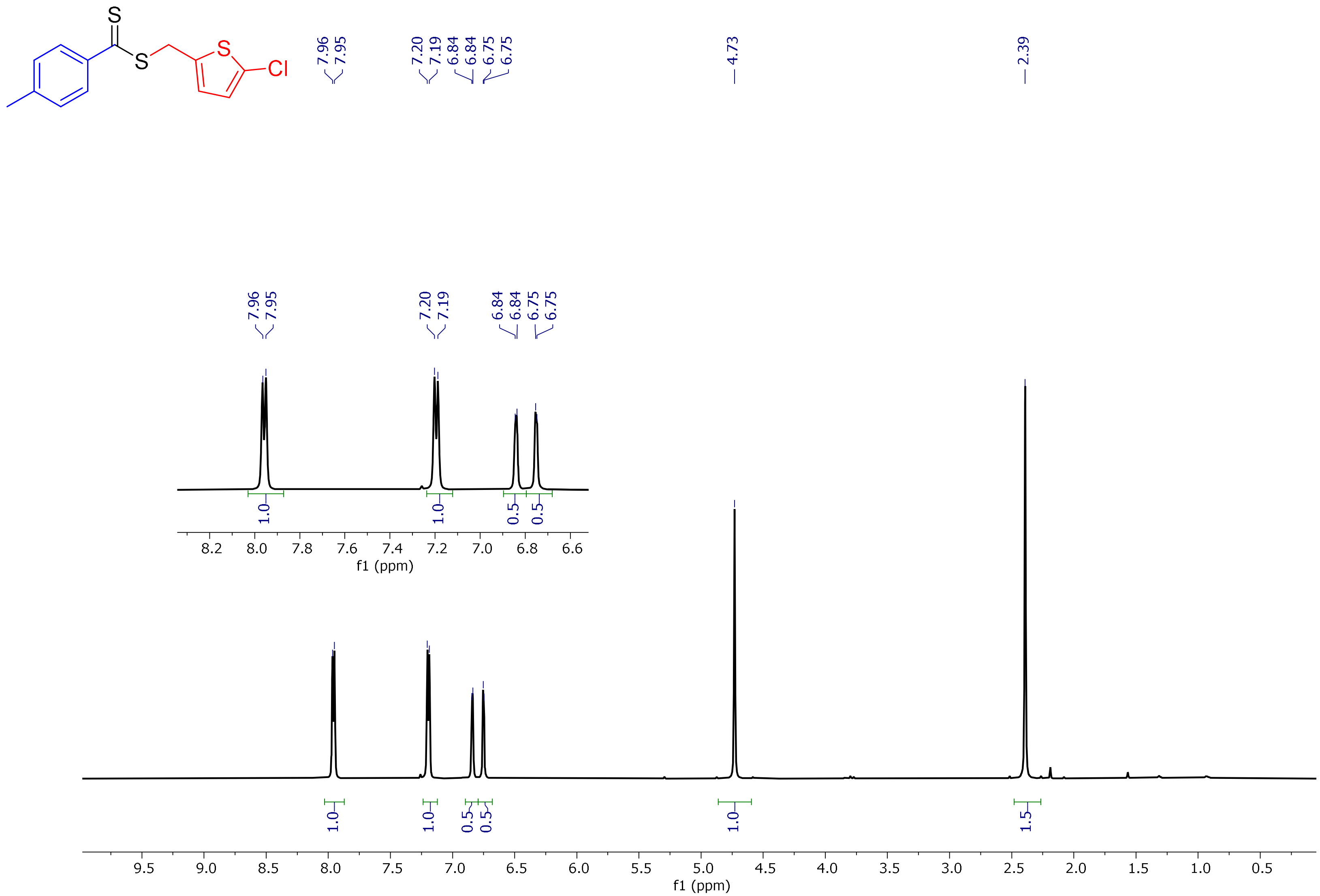

Figure S70. ^{13}C NMR (125 MHz, $CDCl_3$) spectrum for **24a**

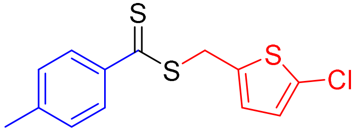

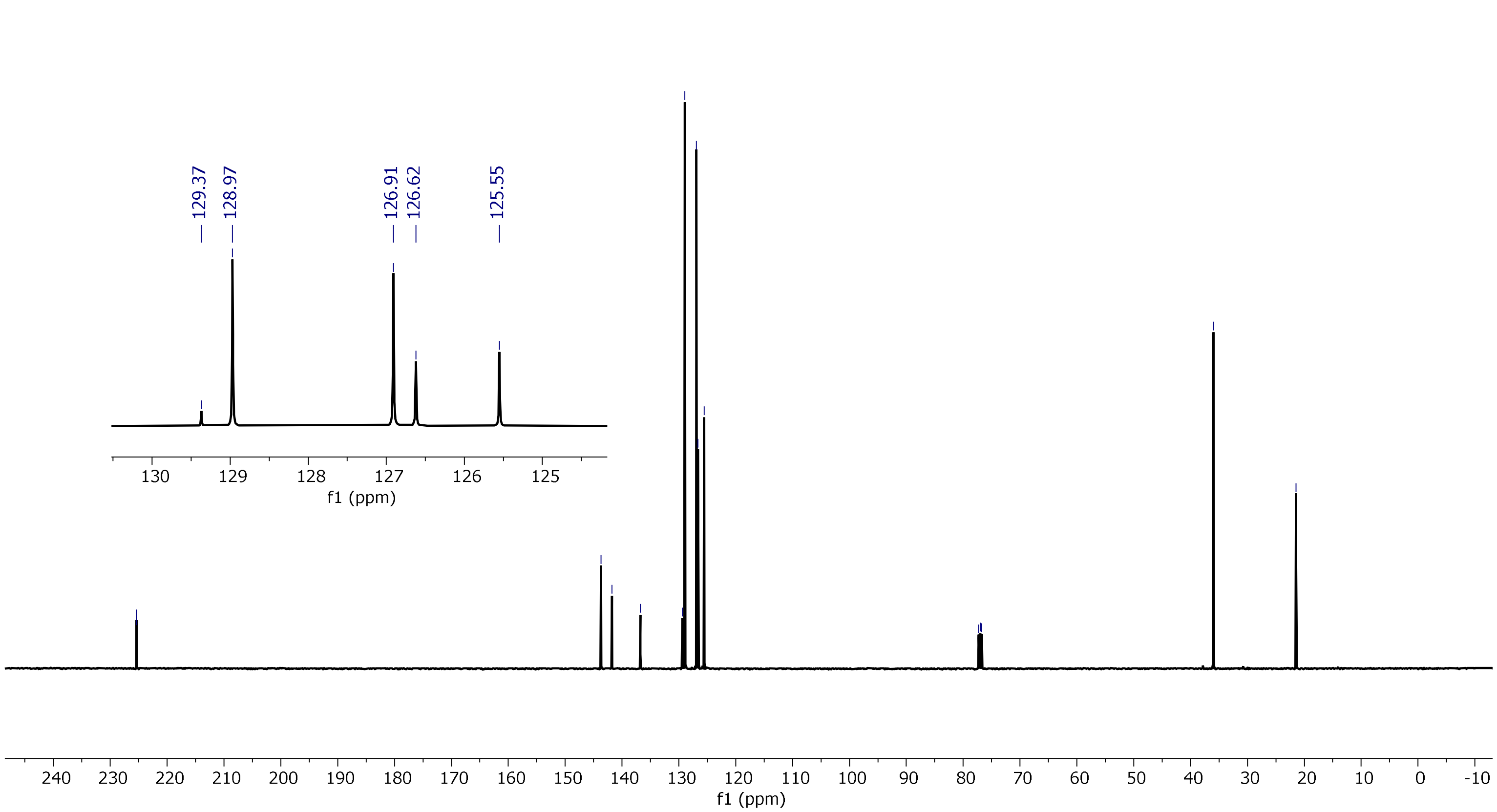

Figure S71. ^{1}H NMR (500 MHz, $CDCl_3$) spectrum for **25a**

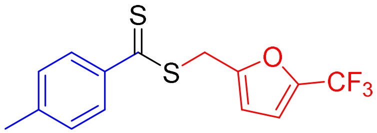

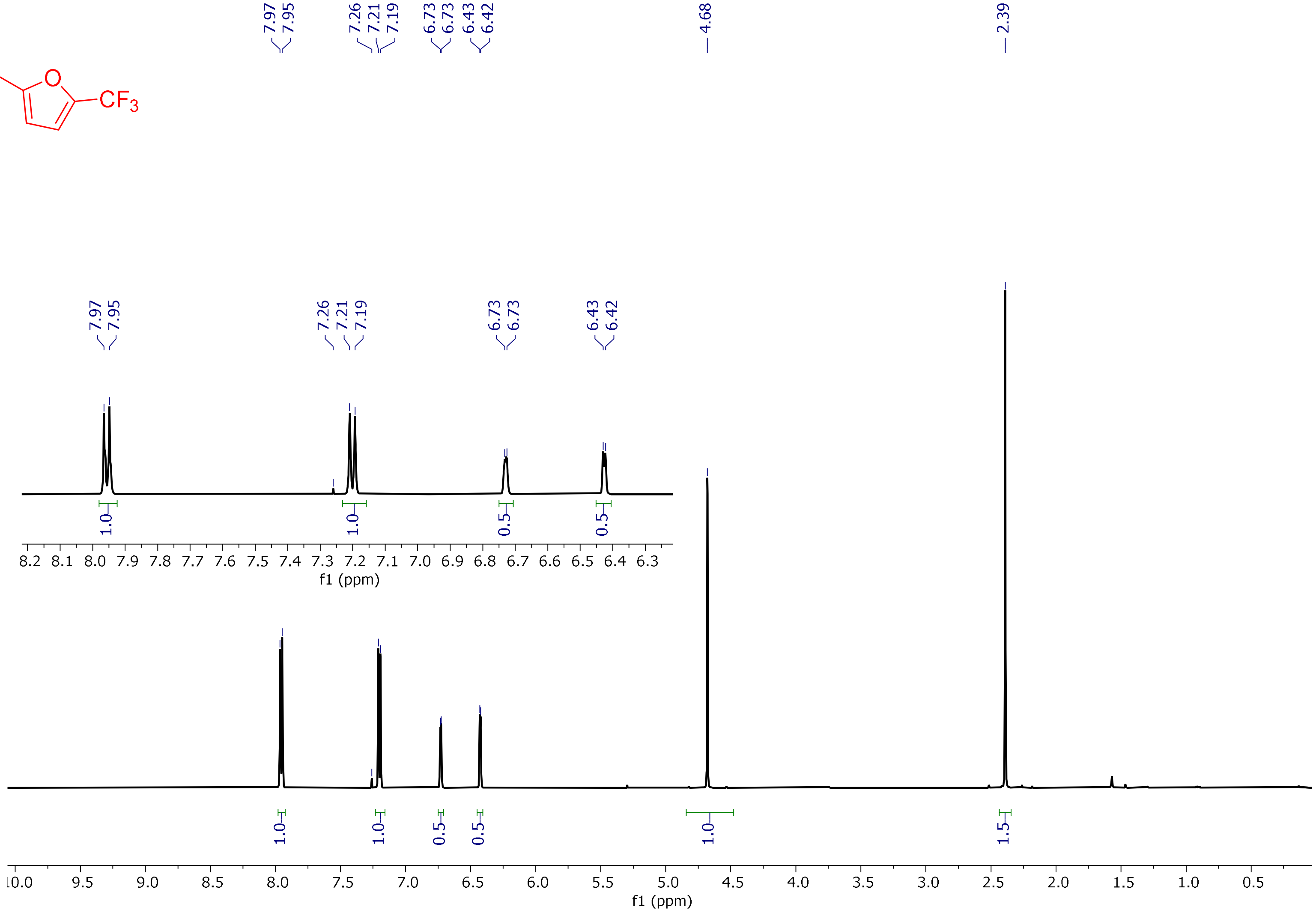

Figure S72. ^{13}C NMR (125 MHz, $CDCl_3$) spectrum for **25a**

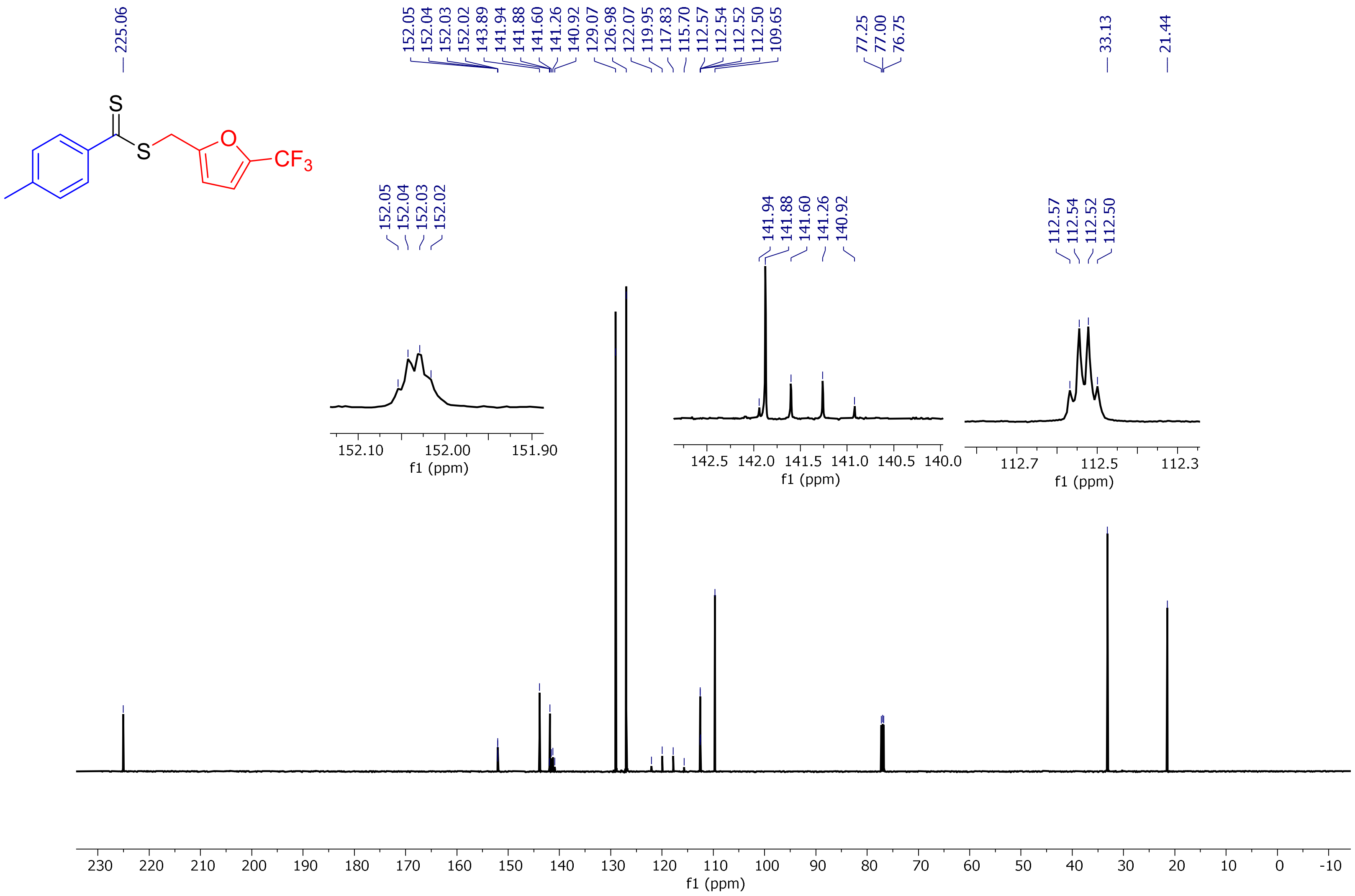

Figure S73. ^{1}H NMR (500 MHz, $CDCl_3$) spectrum for **26a**

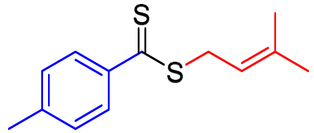

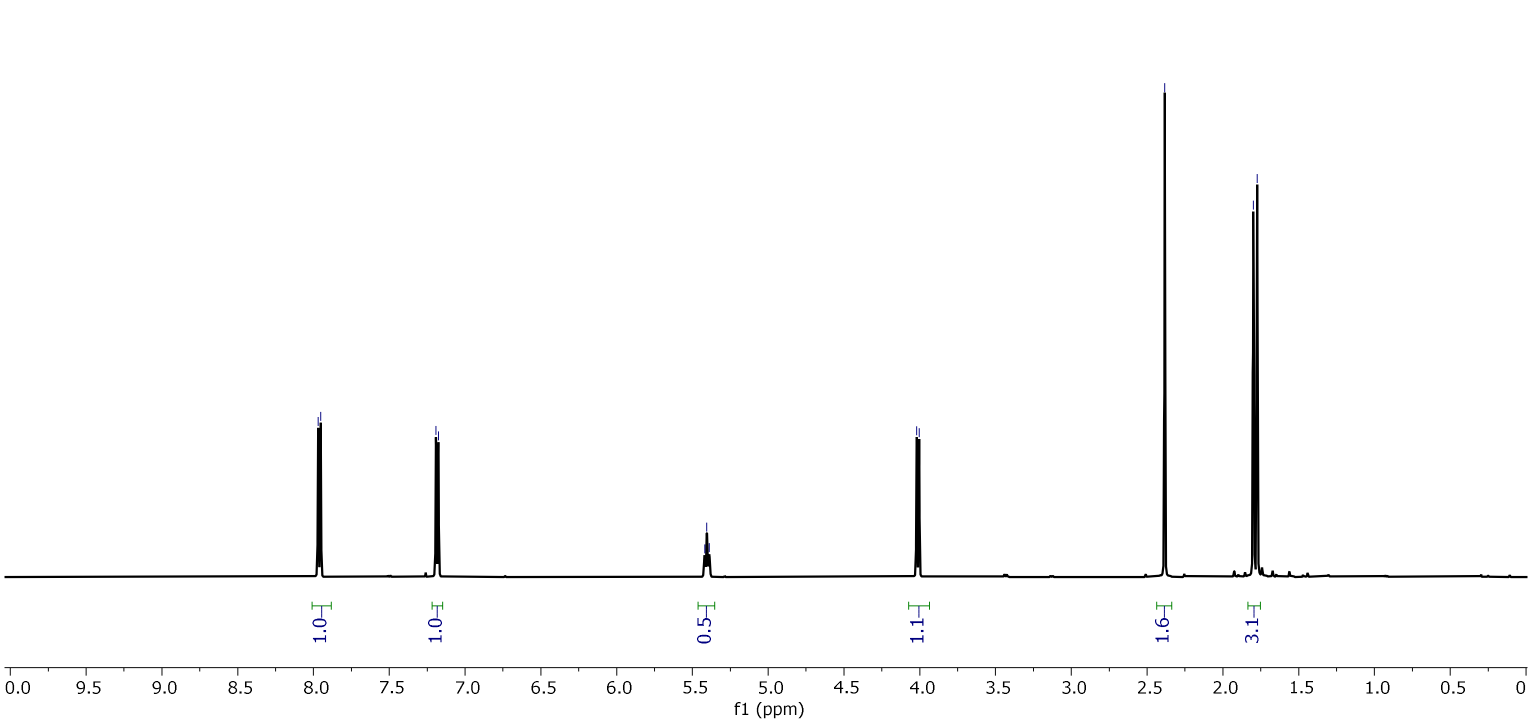

Figure S74. ^{13}C NMR (125 MHz, $CDCl_3$) spectrum for **26a**

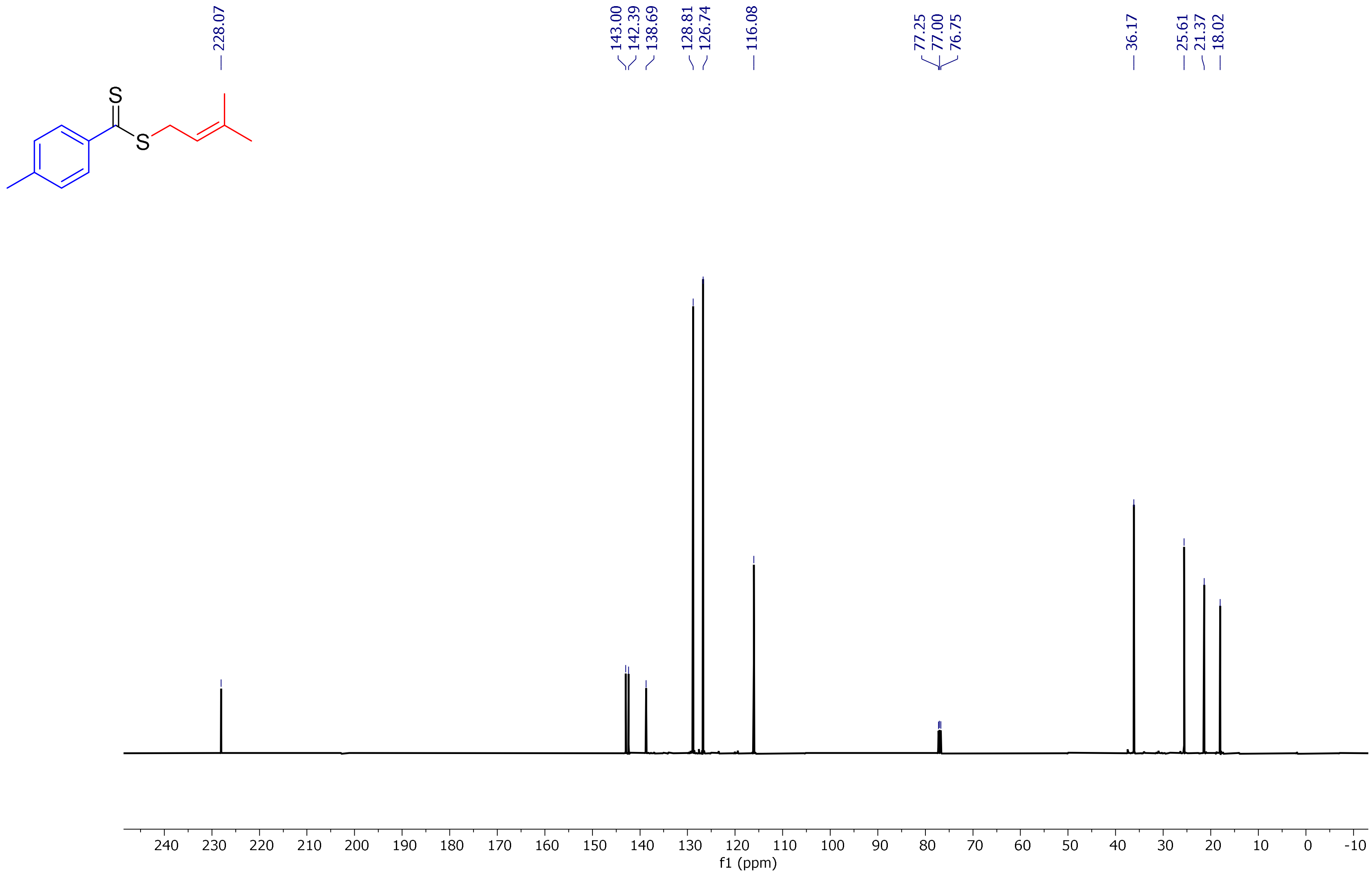

Figure S75. ^{1}H NMR (500 MHz, $CDCl_3$) spectrum for **27a**

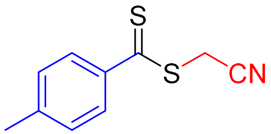

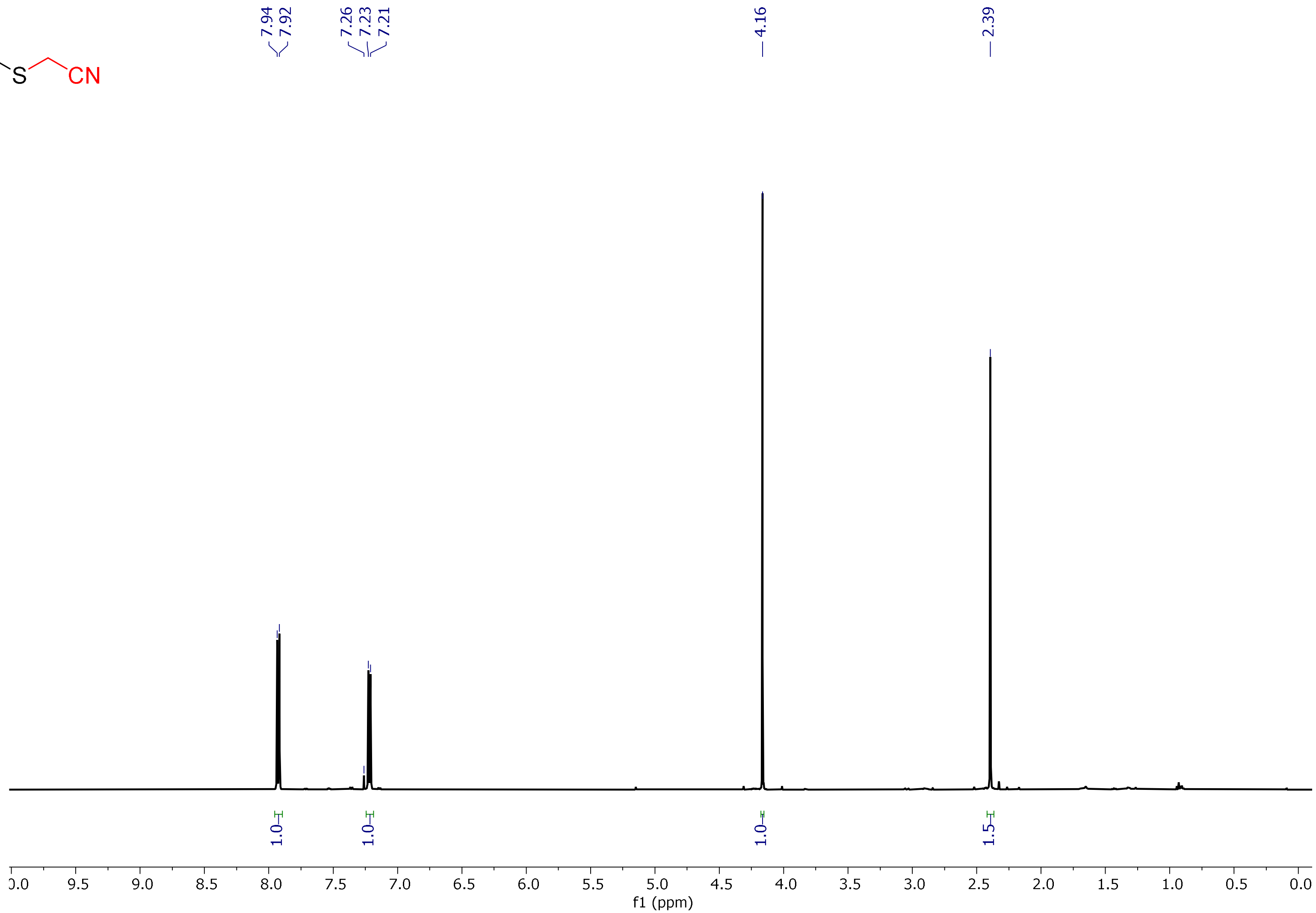

Figure S76. ^{13}C NMR (125 MHz, $CDCl_3$) spectrum for **27a**

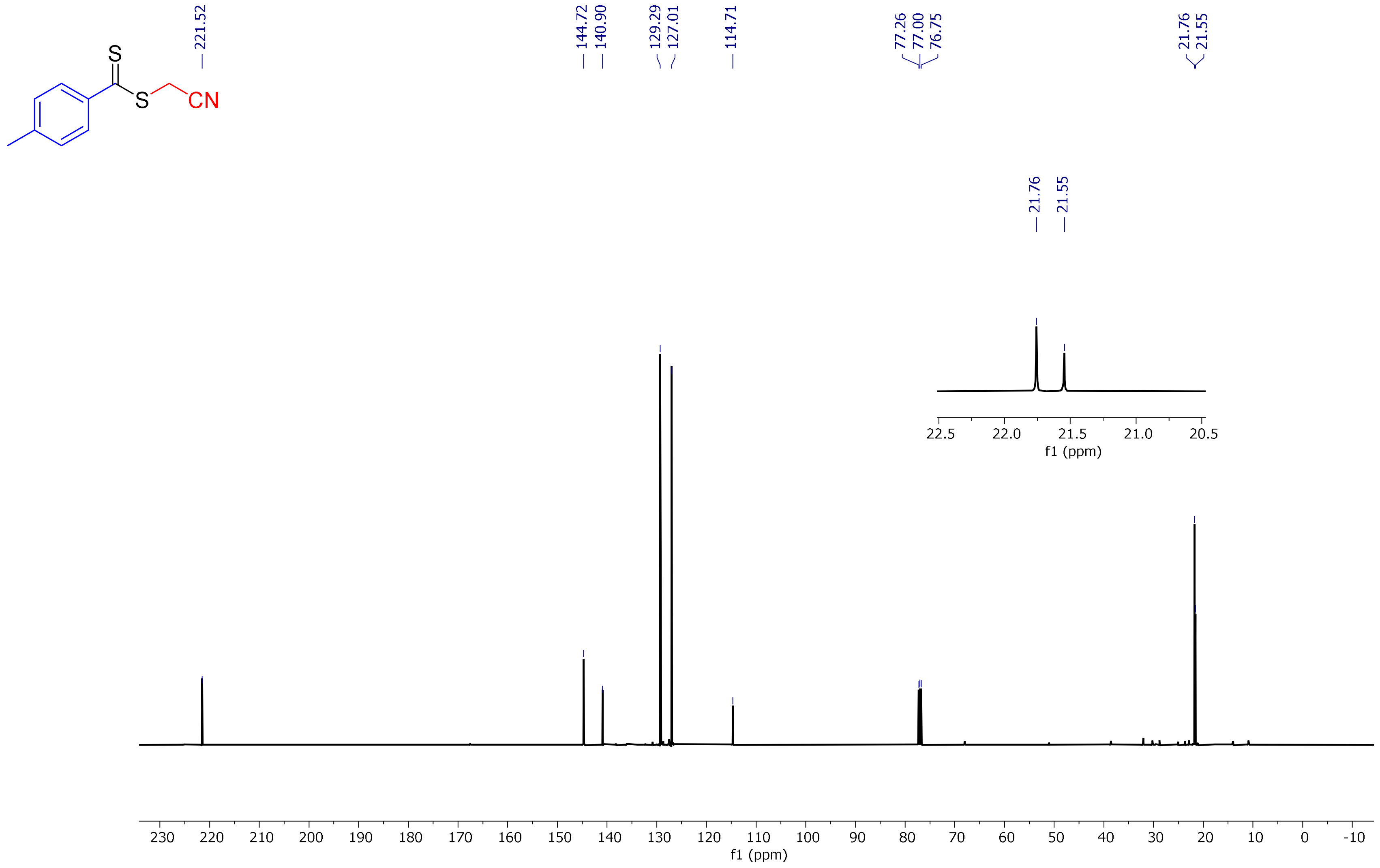

Figure S77. ^{1}H NMR (500 MHz, $CDCl_3$) spectrum for **28a**

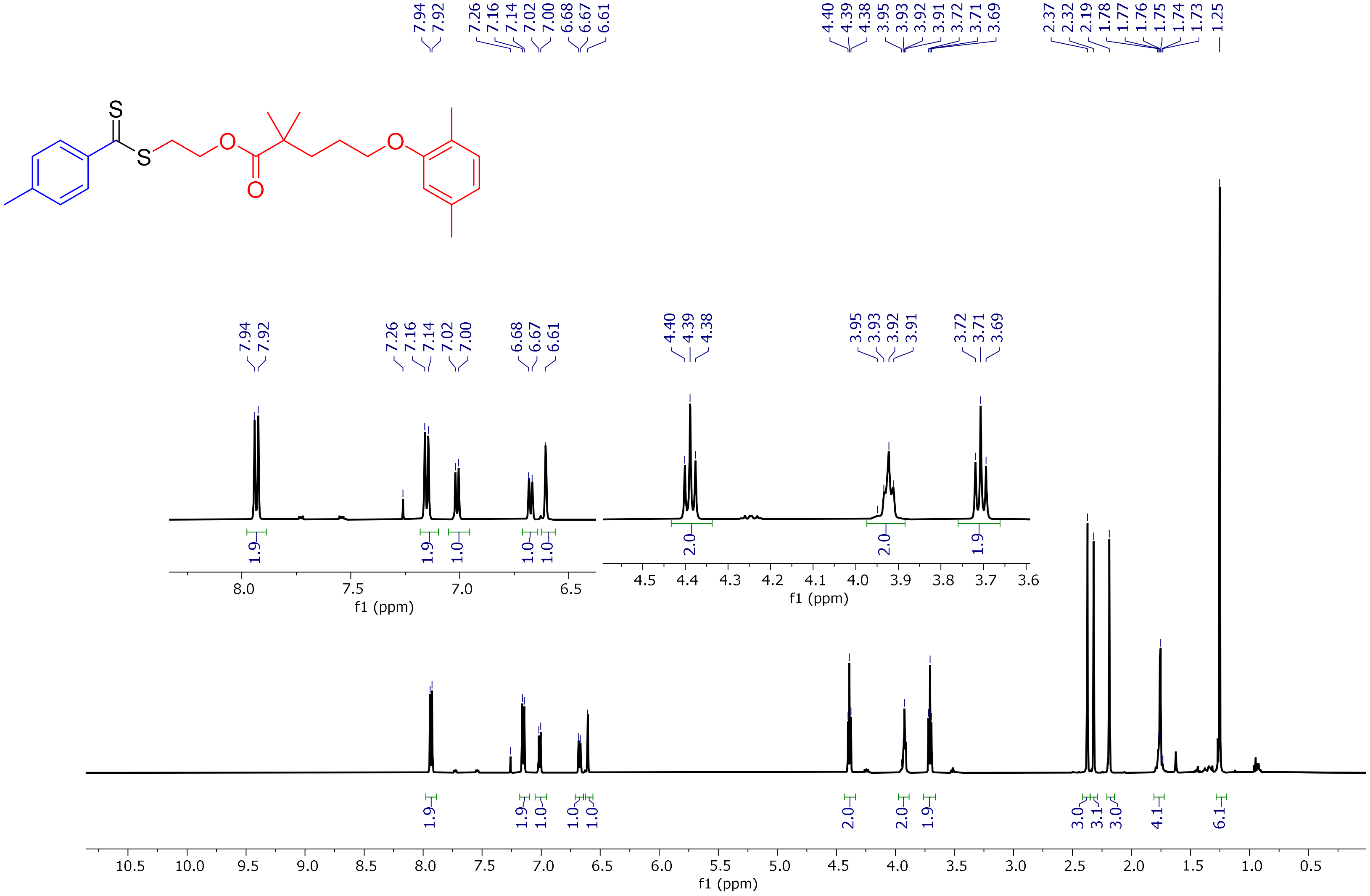

Figure S78. ^{13}C NMR (125 MHz, $CDCl_3$) spectrum for **28a**

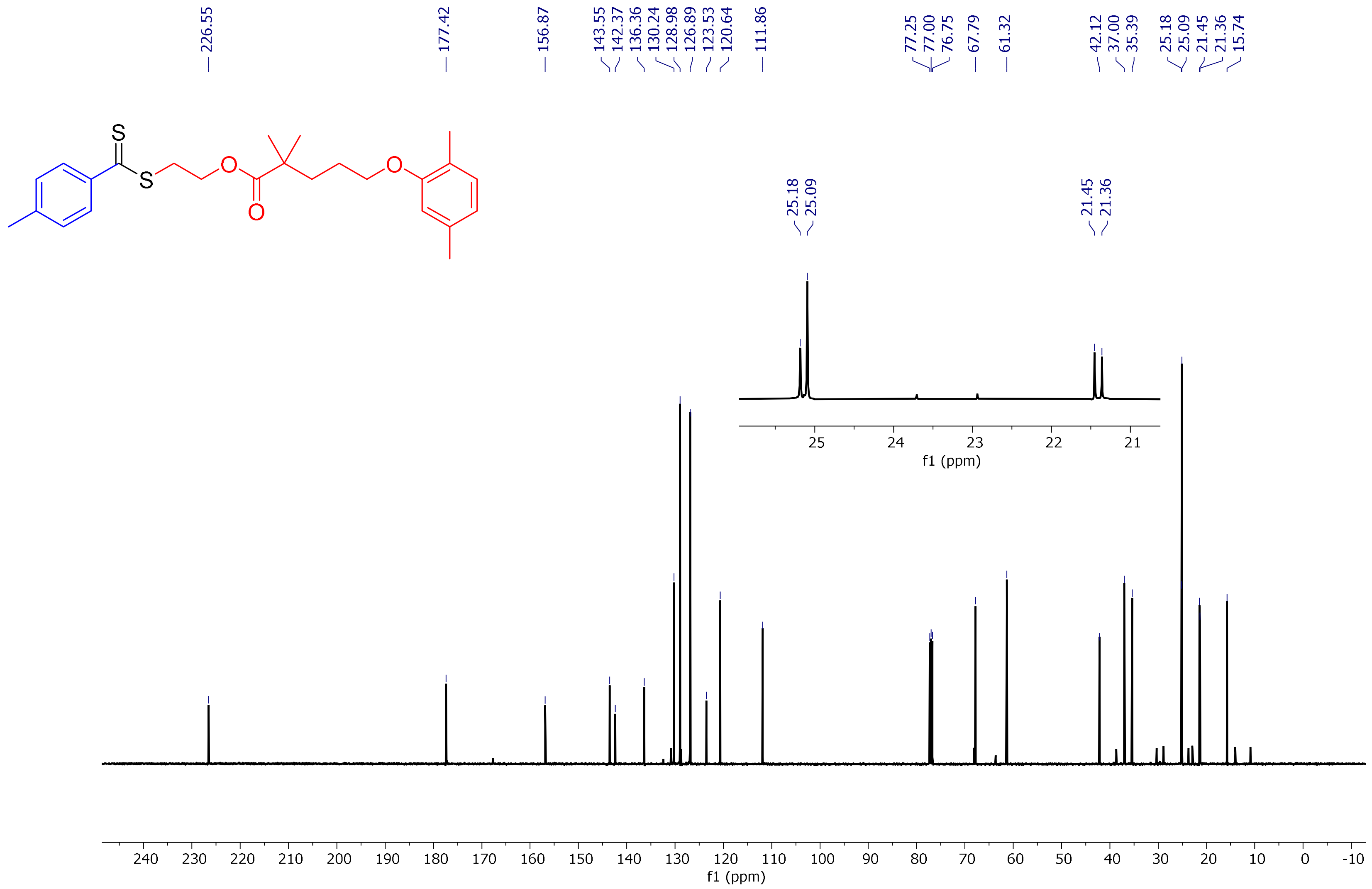

Figure S79. ^{13}C-APT NMR (125 MHz, $CDCl_3$) spectrum for **28a**

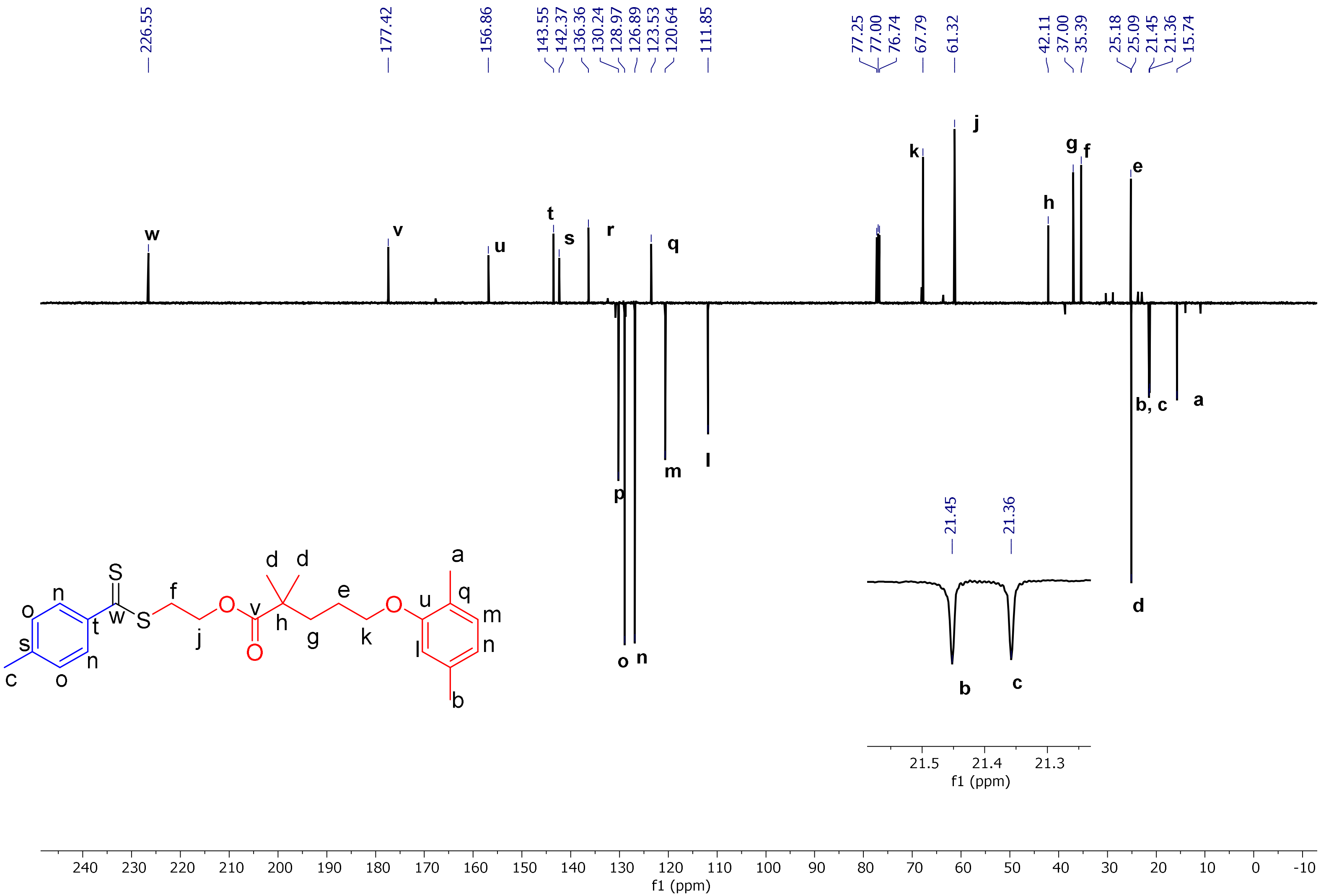

Figure S80. ^{1}H NMR (500 MHz, $CDCl_3$) spectrum for **29a**

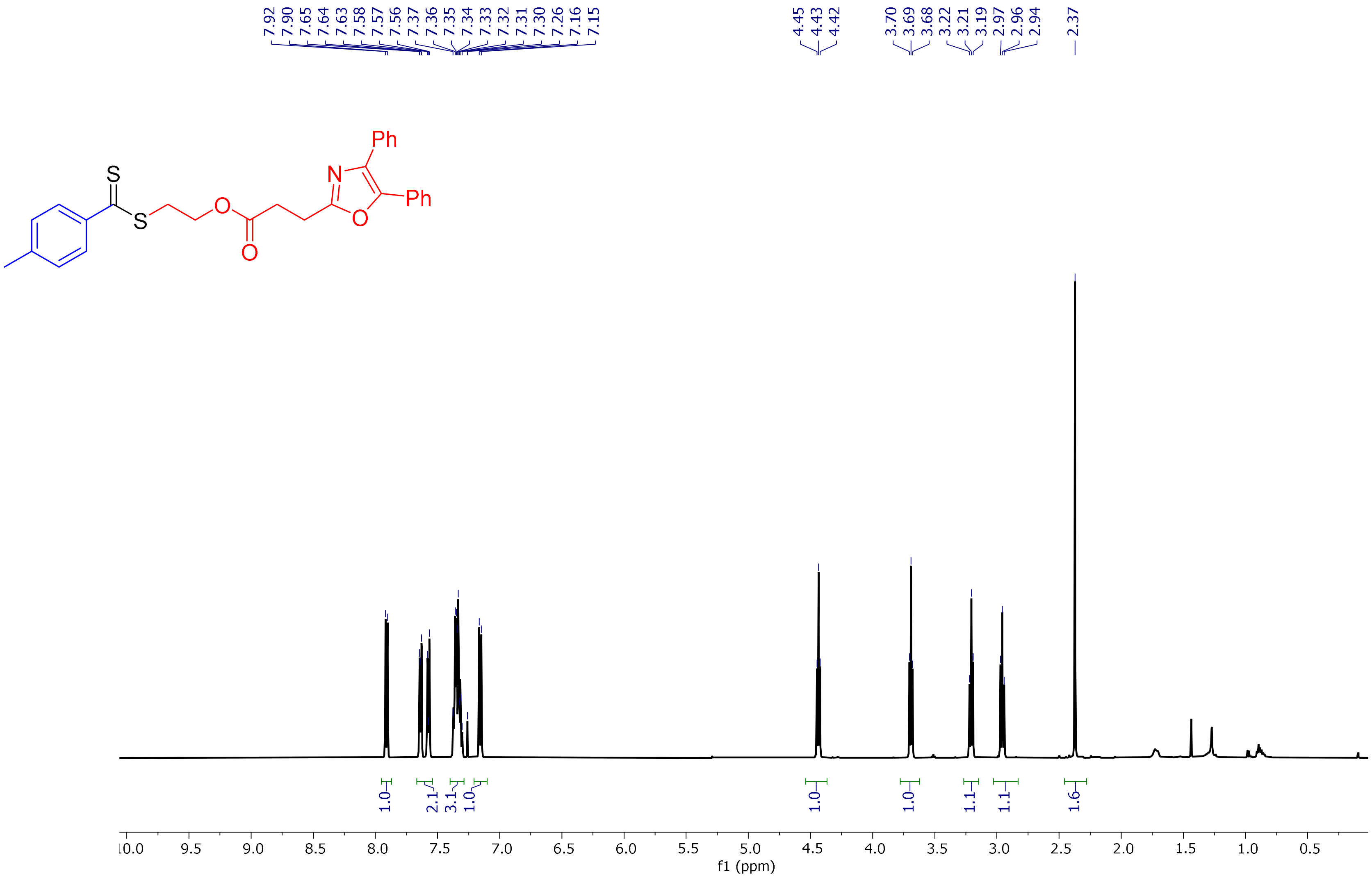

Figure S81. ^{13}C NMR (125 MHz, $CDCl_3$) spectrum for **29a**

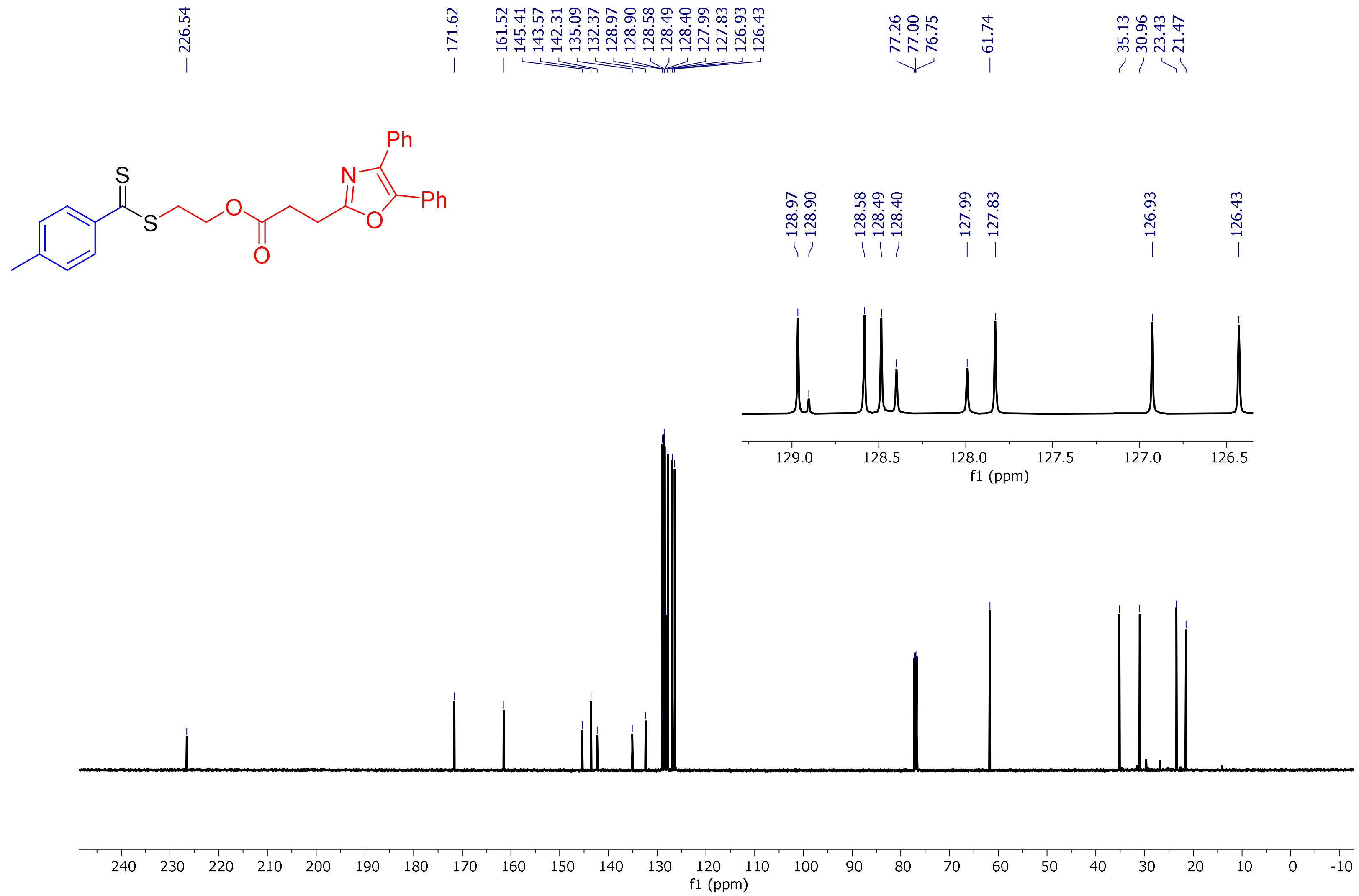

Figure S82. ^{1}H NMR (500 MHz, $CDCl_3$) spectrum for **30a**

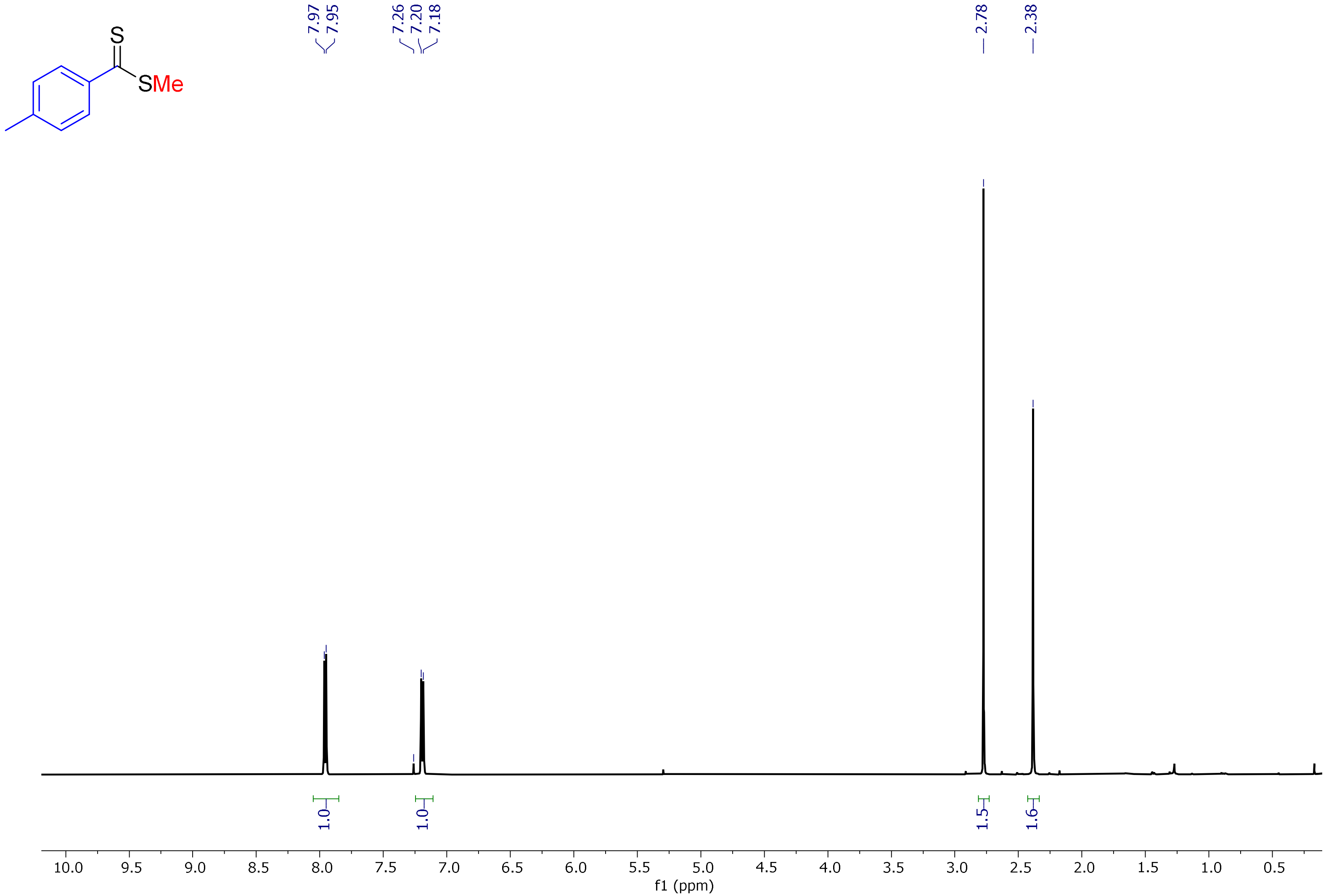

Figure S83. ^{13}C NMR (125 MHz, $CDCl_3$) spectrum for **30a**

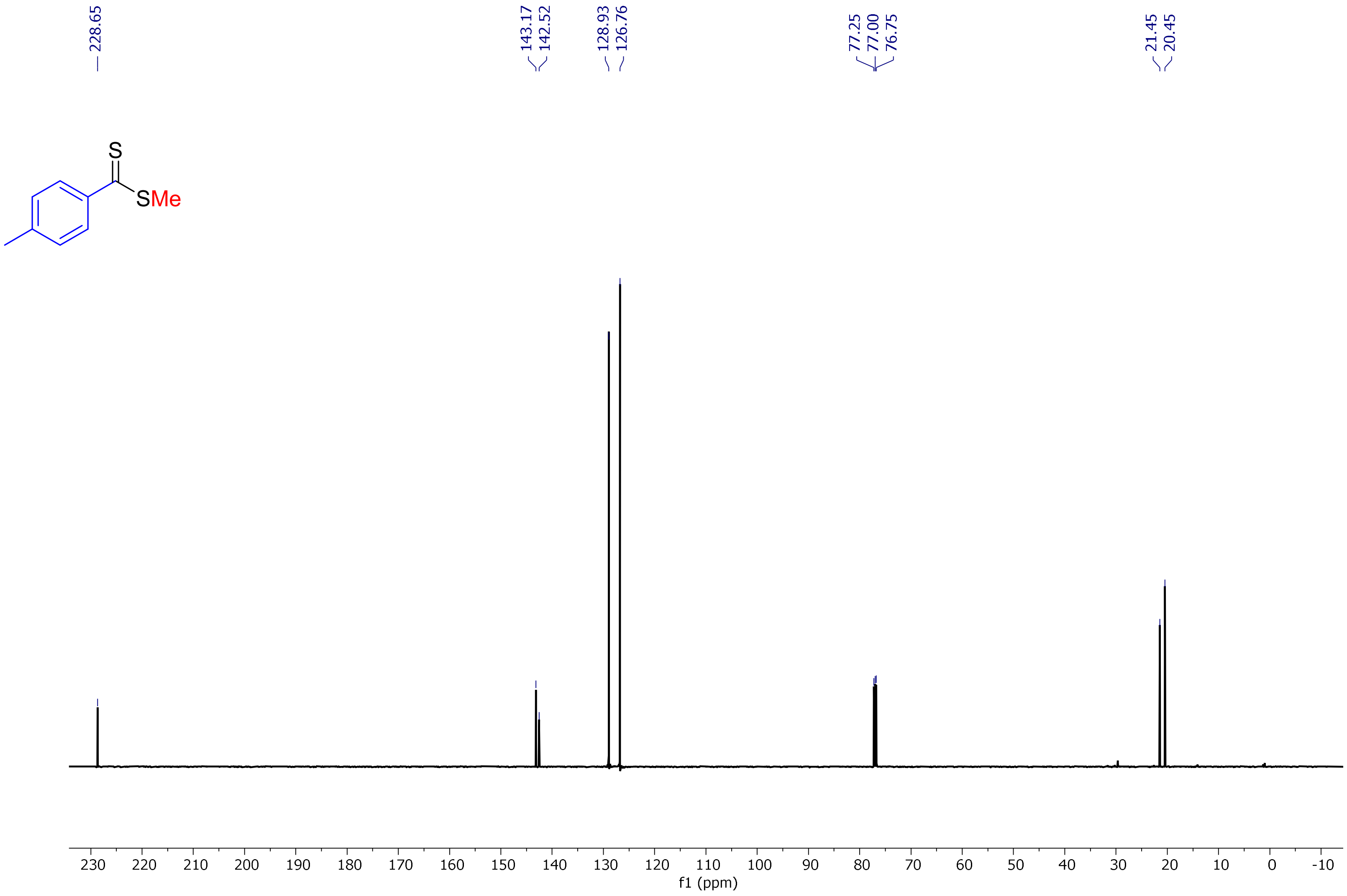

Figure S84. ^{1}H NMR (500 MHz, $CDCl_3$) spectrum for **30b**

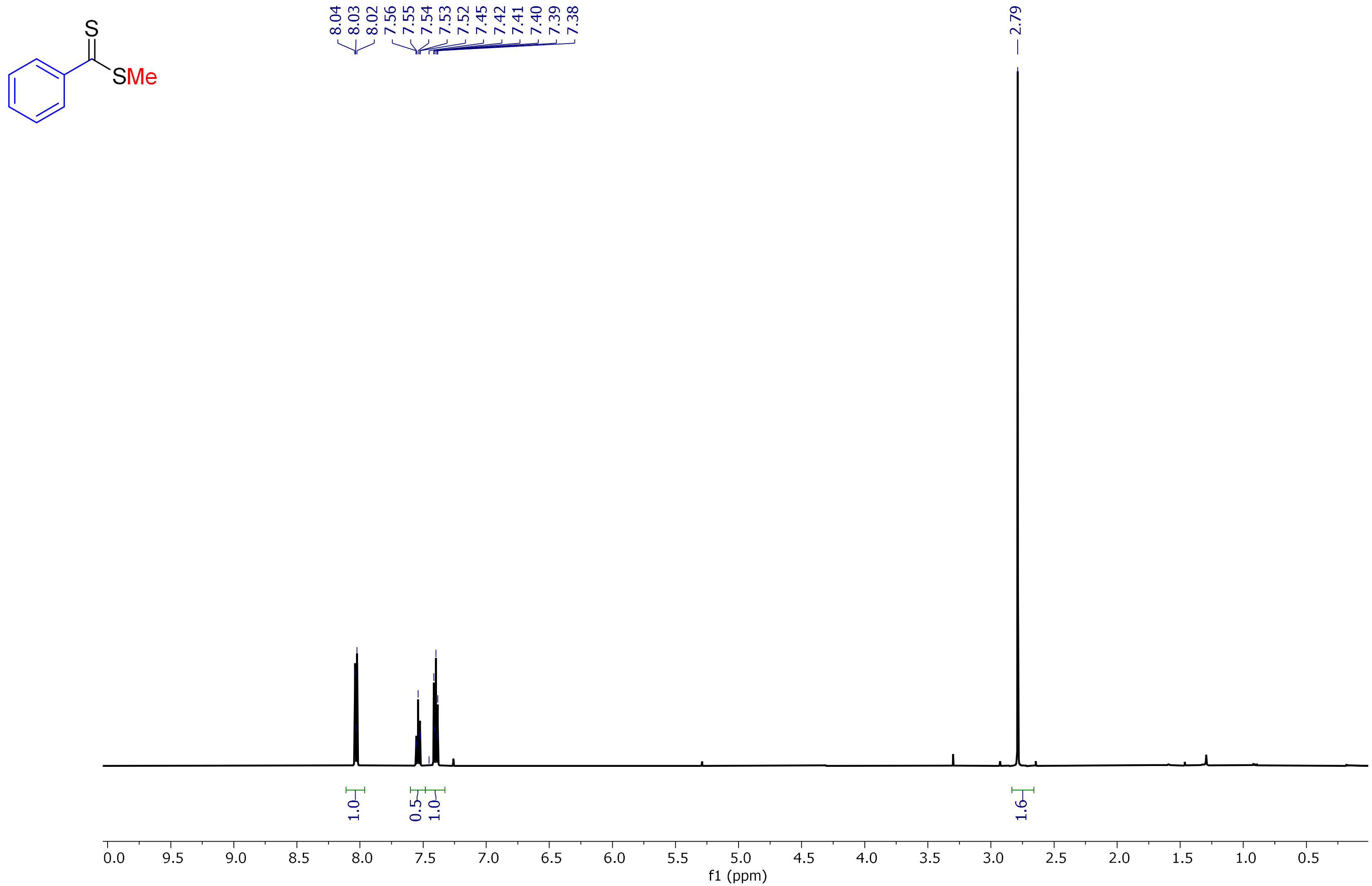

Figure S85. ^{13}C NMR (125 MHz, $CDCl_3$) spectrum for **30b**

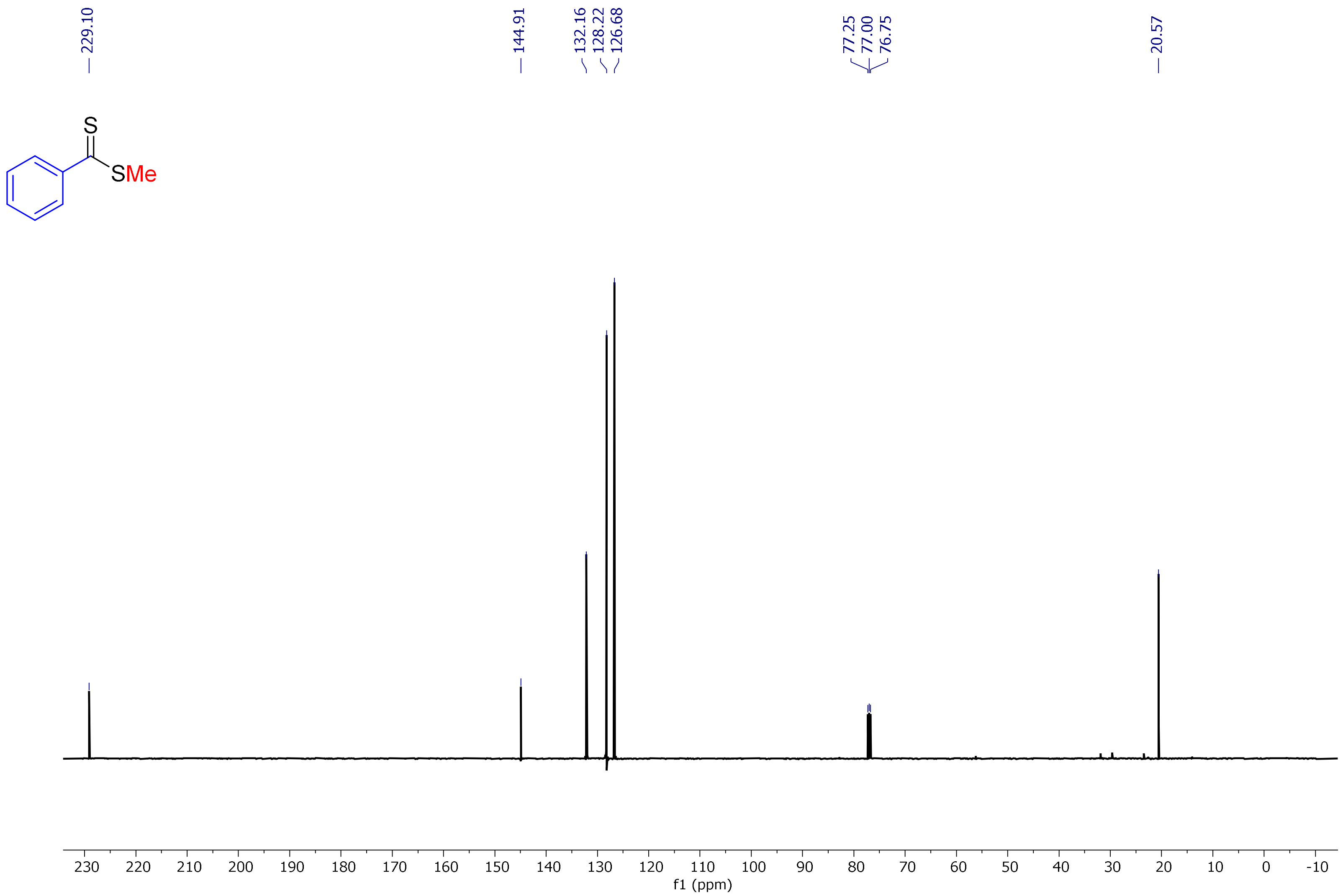

Figure S86. ^{1}H NMR (500 MHz, $CDCl_3$) spectrum for **30c**

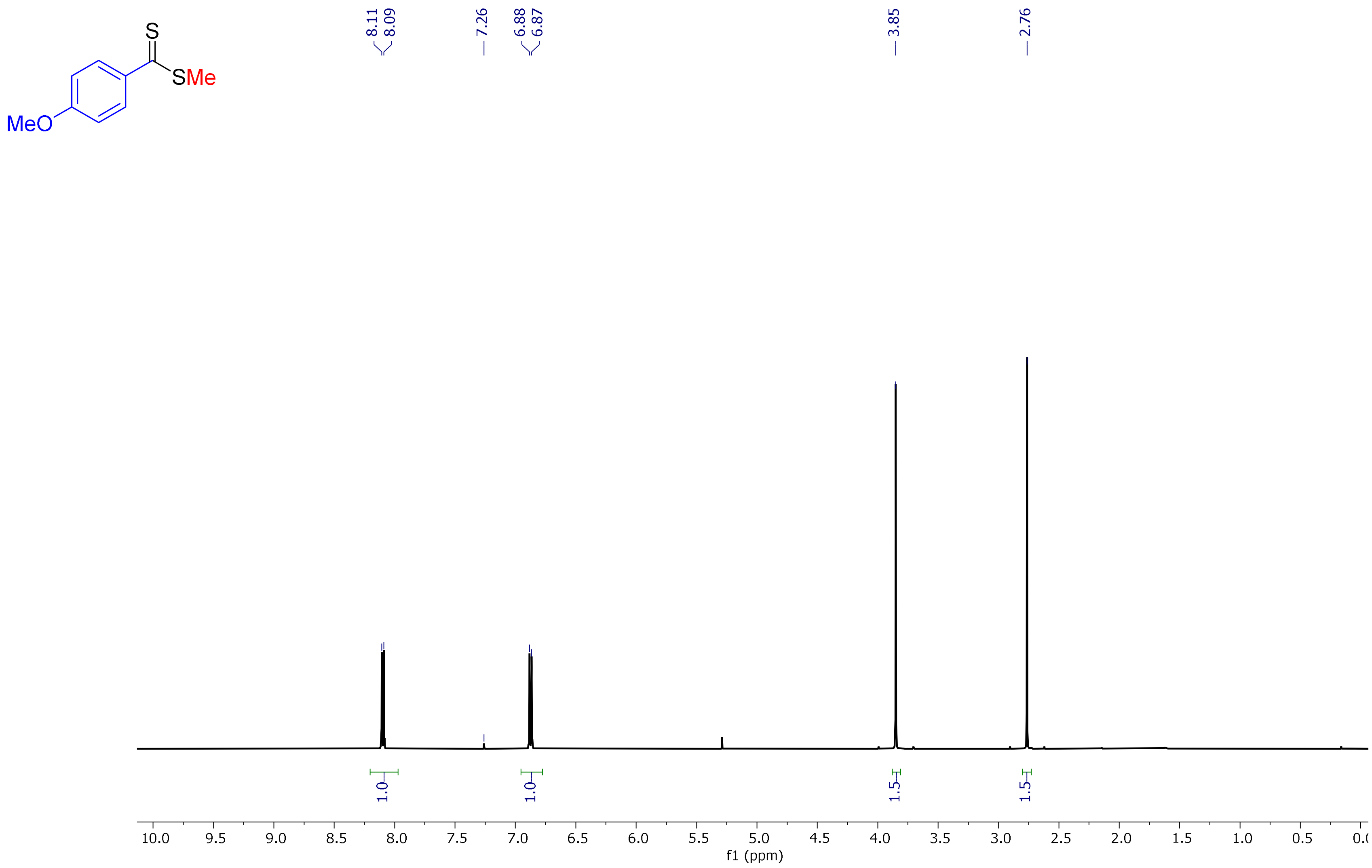

Figure S87. ^{13}C NMR (125 MHz, $CDCl_3$) spectrum for **30c**

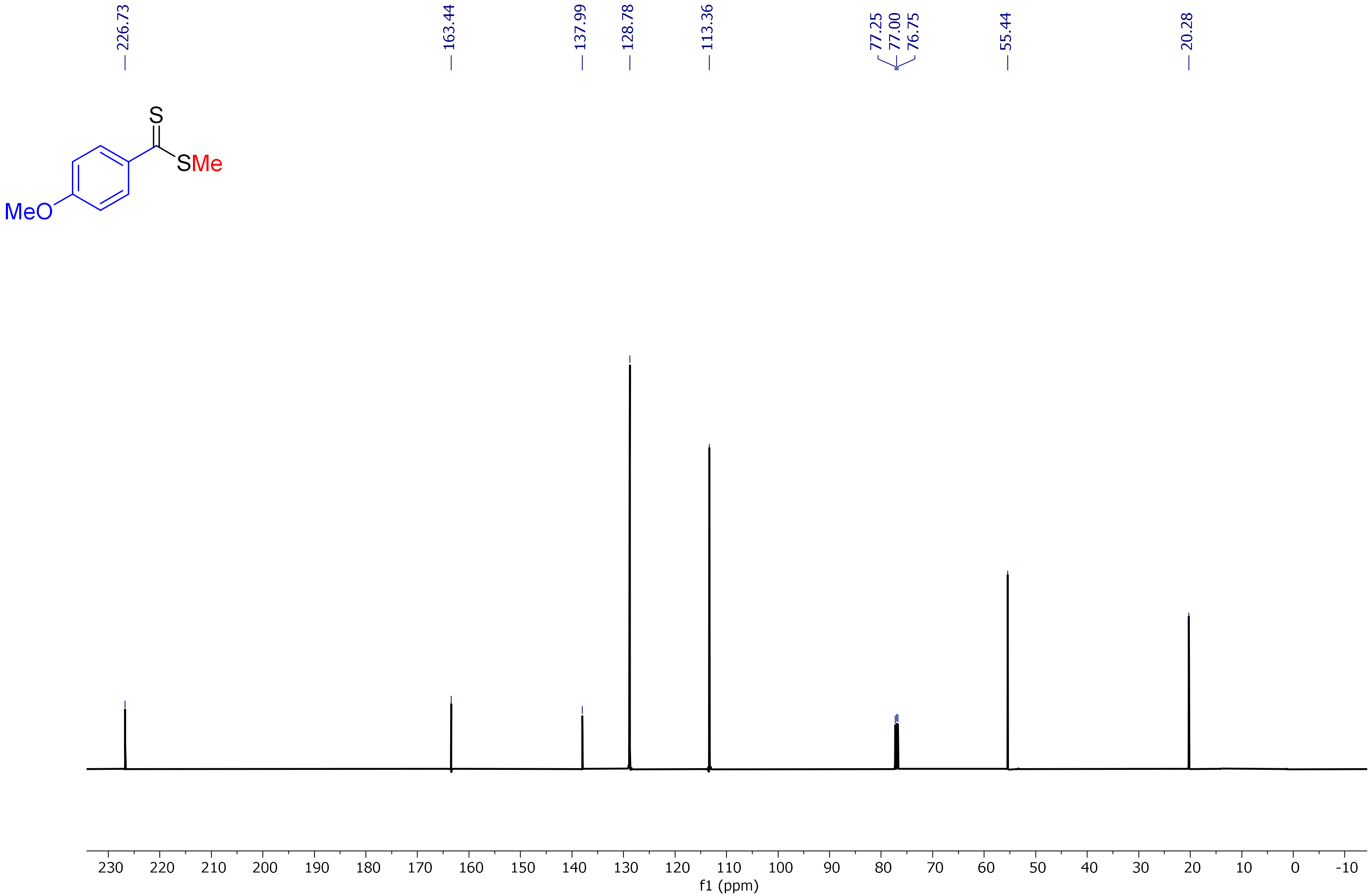

Figure S88. ^{1}H NMR (500 MHz, $CDCl_3$) spectrum for **30d**

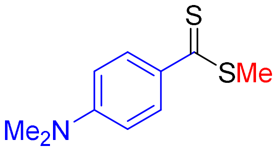

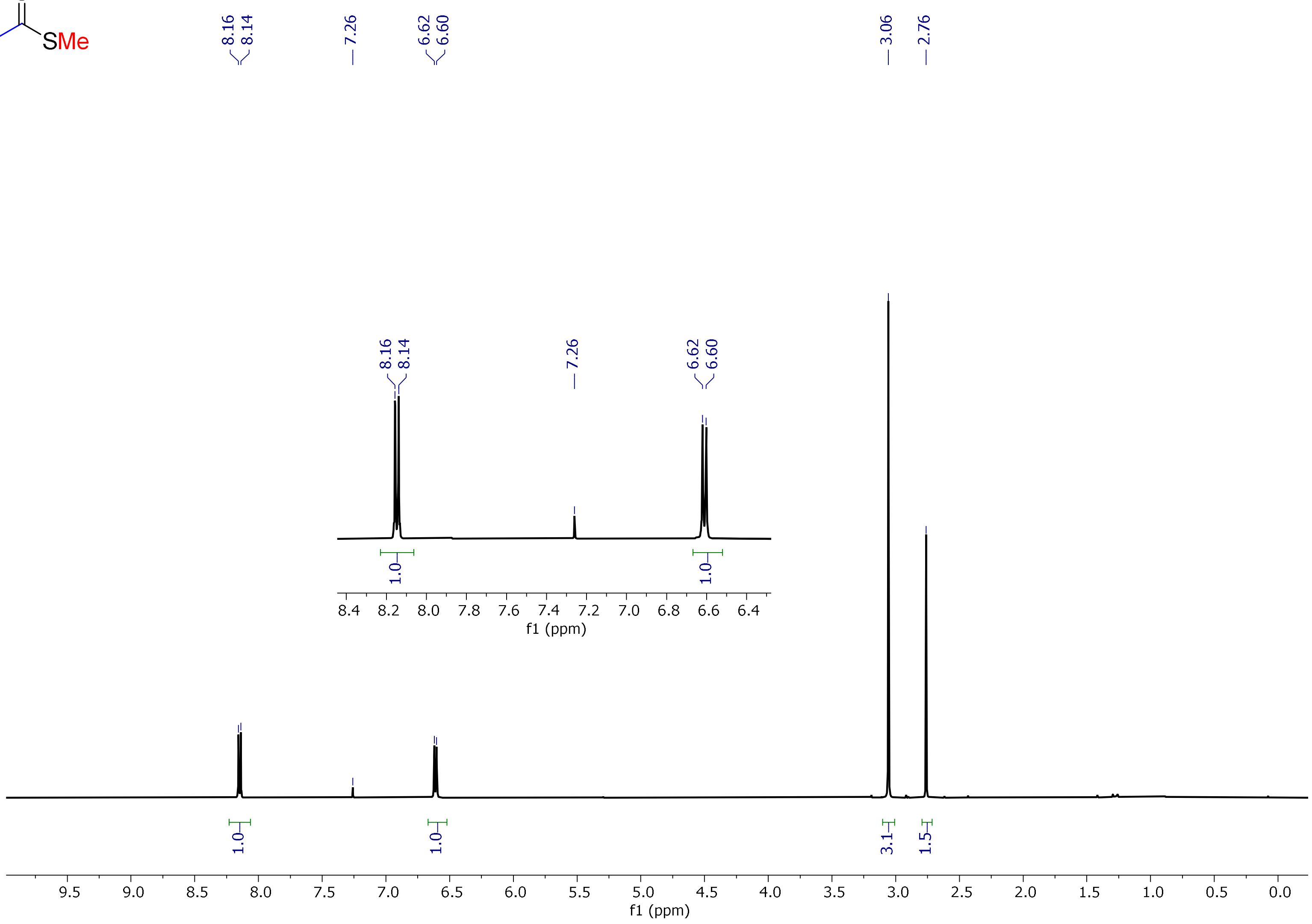

Figure S89. ^{13}C NMR (125 MHz, $CDCl_3$) spectrum for **30d**

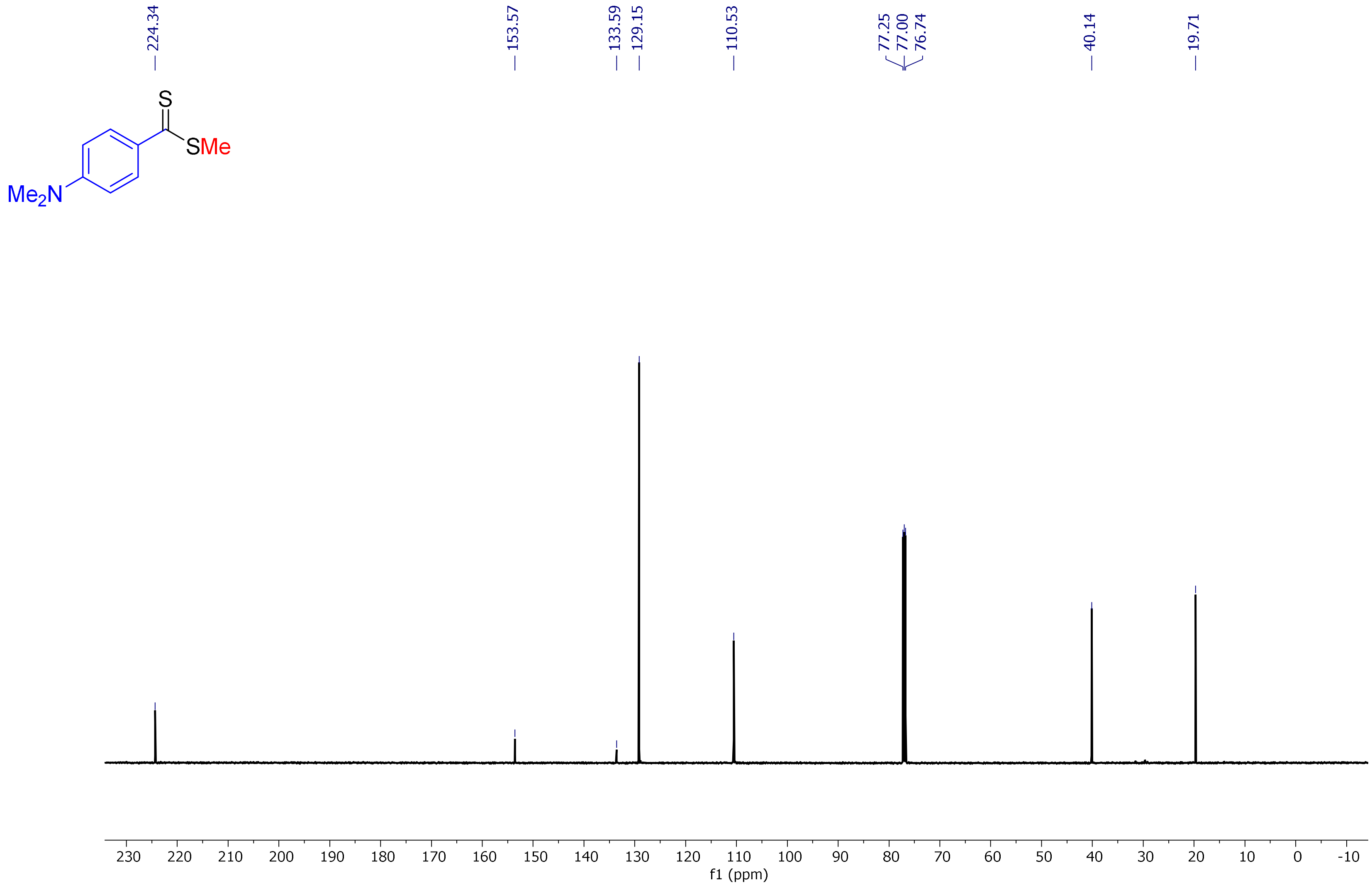

Figure S90. ^{1}H NMR (500 MHz, $CDCl_3$) spectrum for **30e**

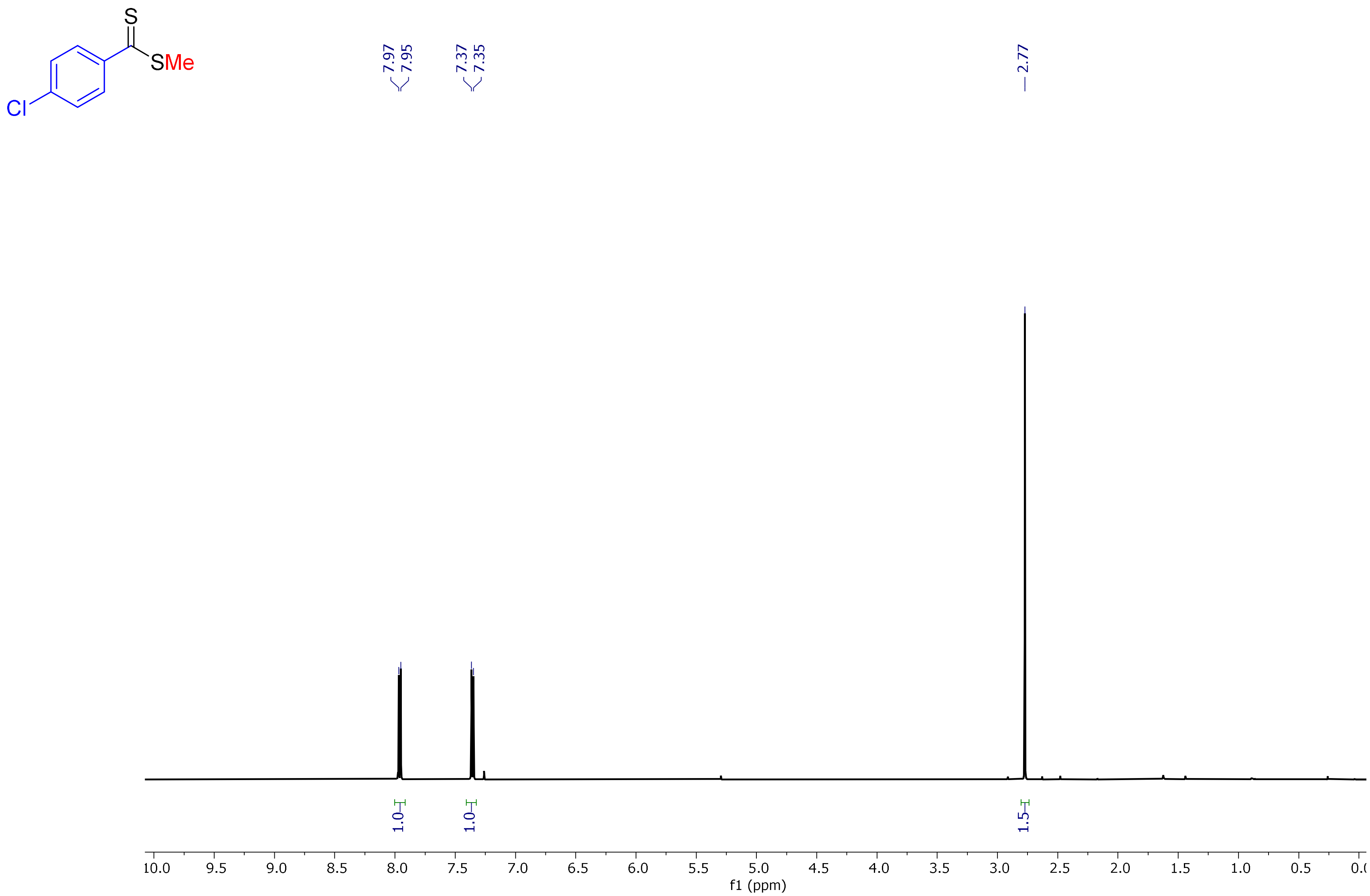

Figure S91. ^{13}C NMR (125 MHz, $CDCl_3$) spectrum for **30e**

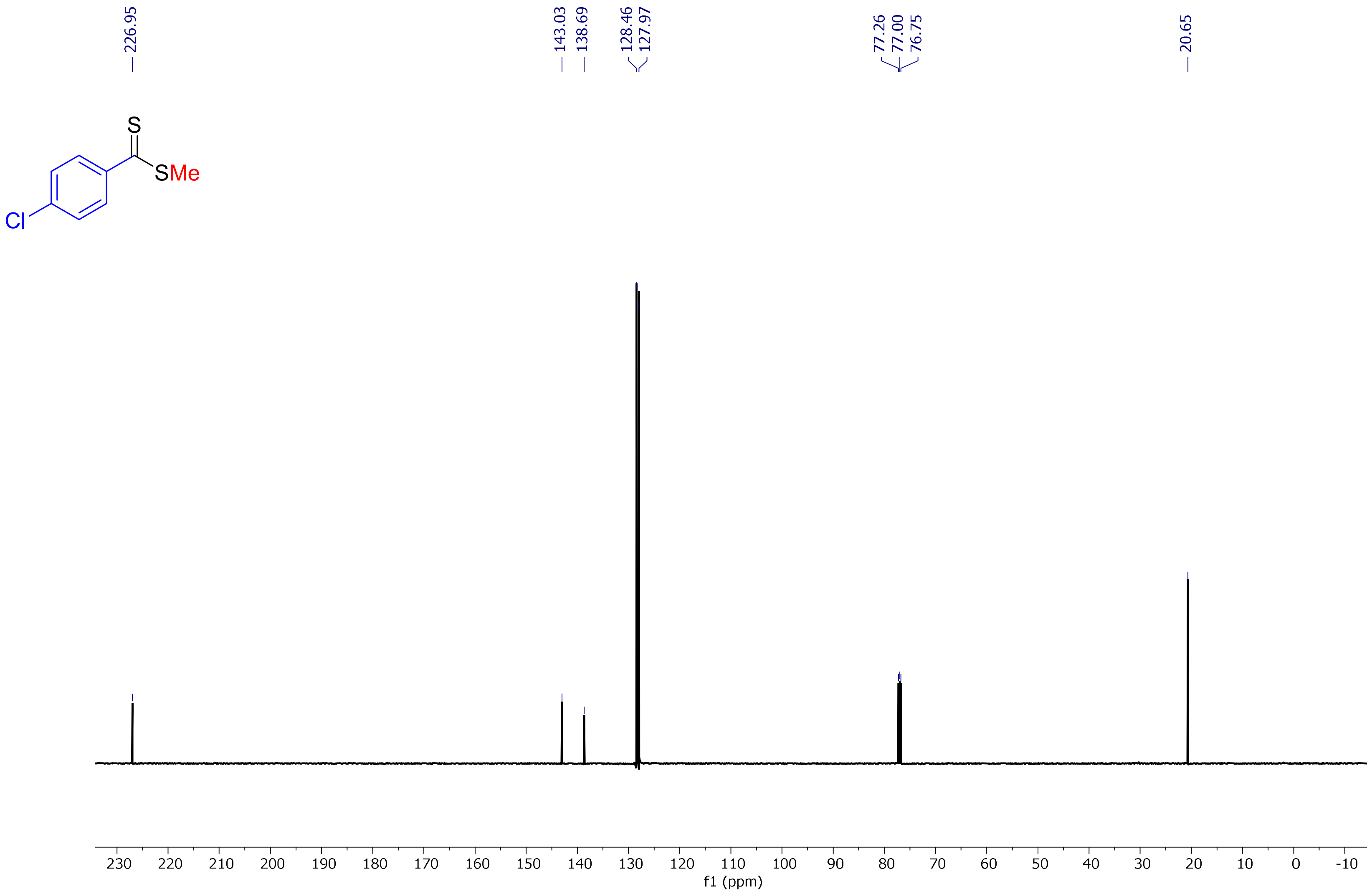

Figure S92. ^{1}H NMR (500 MHz, $CDCl_3$) spectrum for **30f**

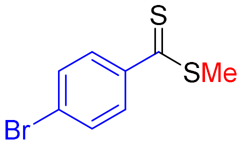

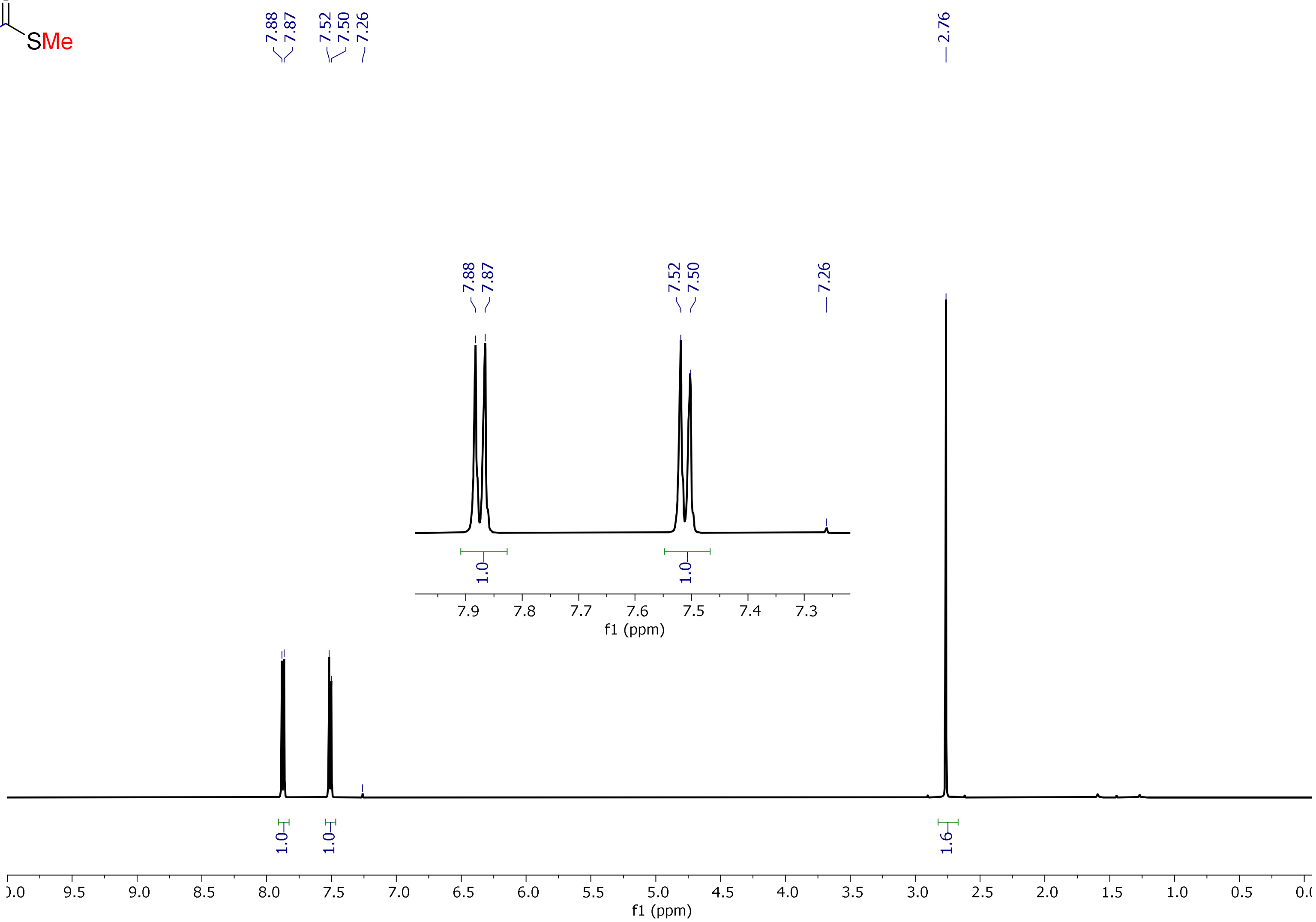

Figure S93. ^{13}C NMR (125 MHz, $CDCl_3$) spectrum for **30f**

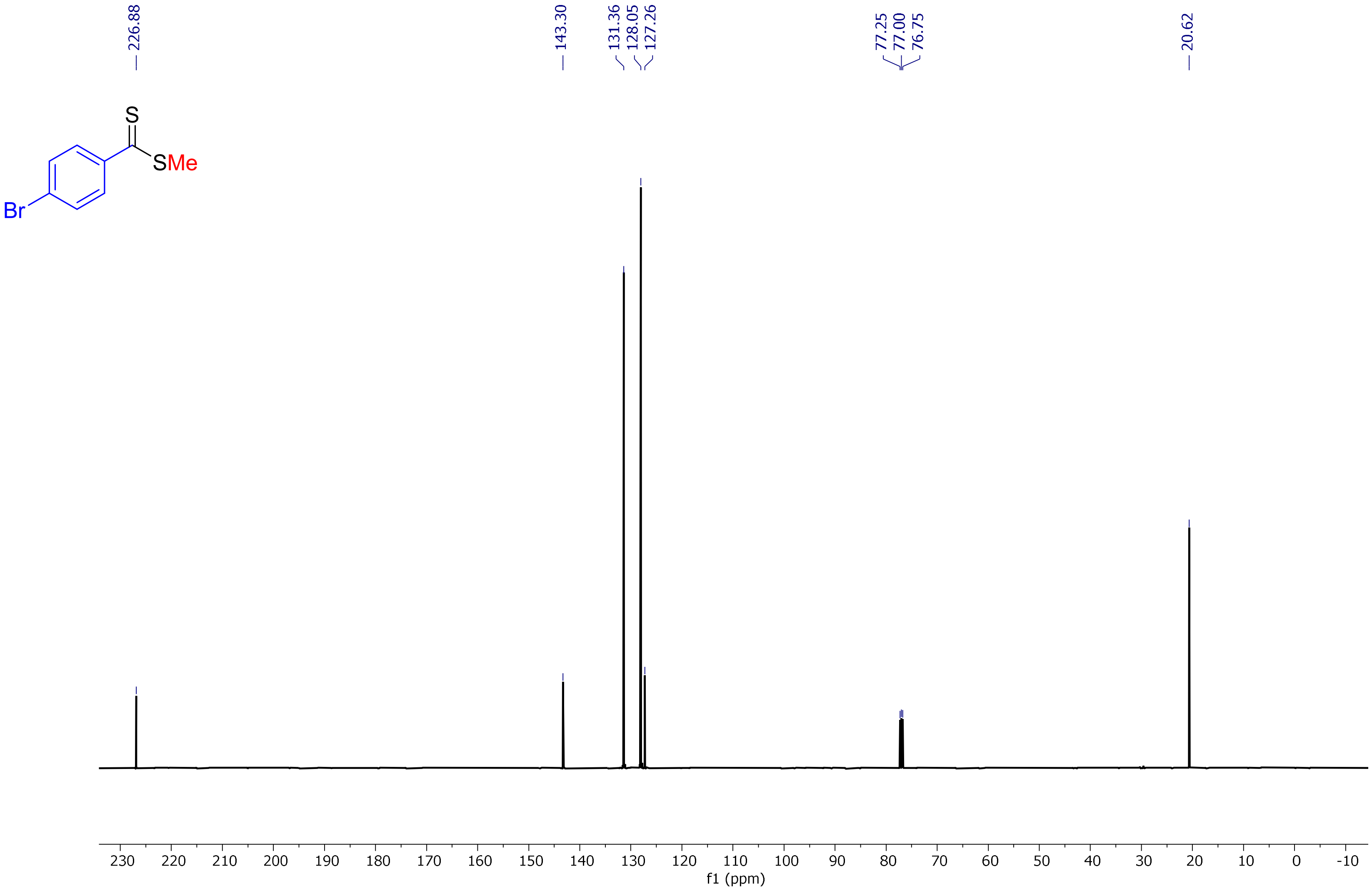

Figure S94.^{1}H NMR (500 MHz, $CDCl_3$) spectrum for **30g**

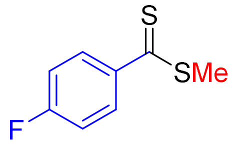

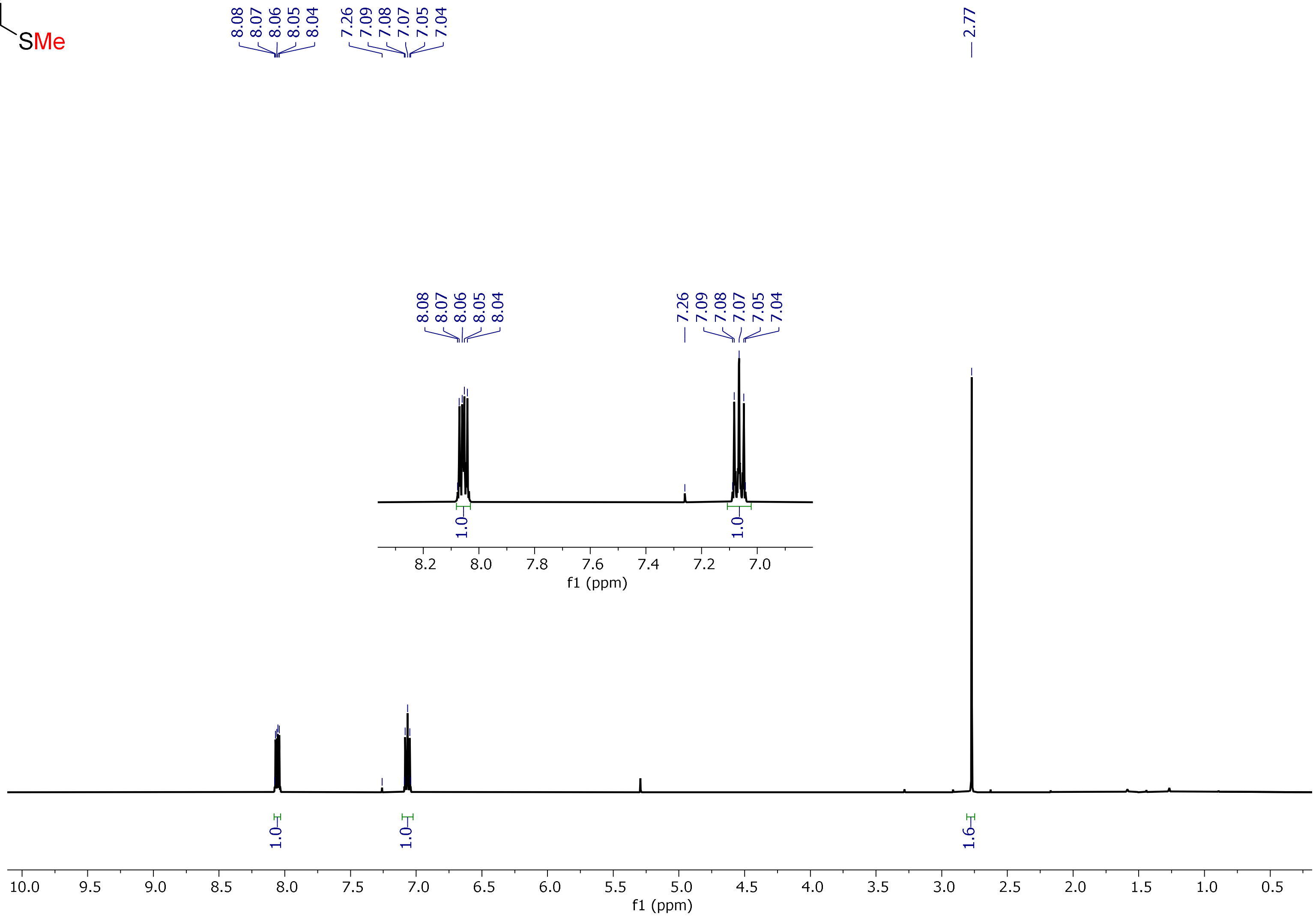

Figure S95. ^{13}C NMR (125 MHz, $CDCl_3$) spectrum for **30g**

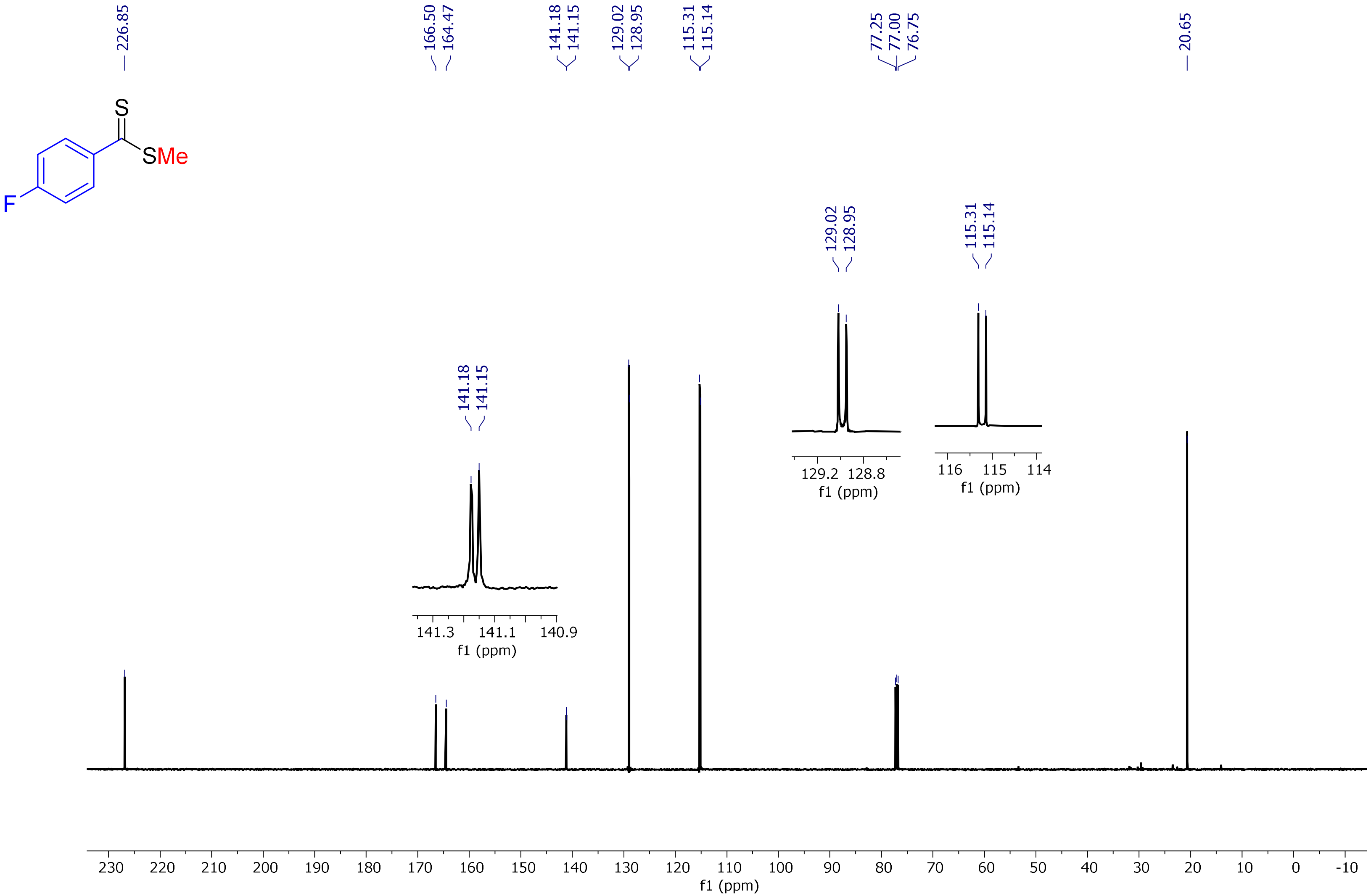

Figure S96. ^{1}H NMR (500 MHz, $CDCl_3$) spectrum for **30i**

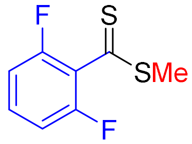

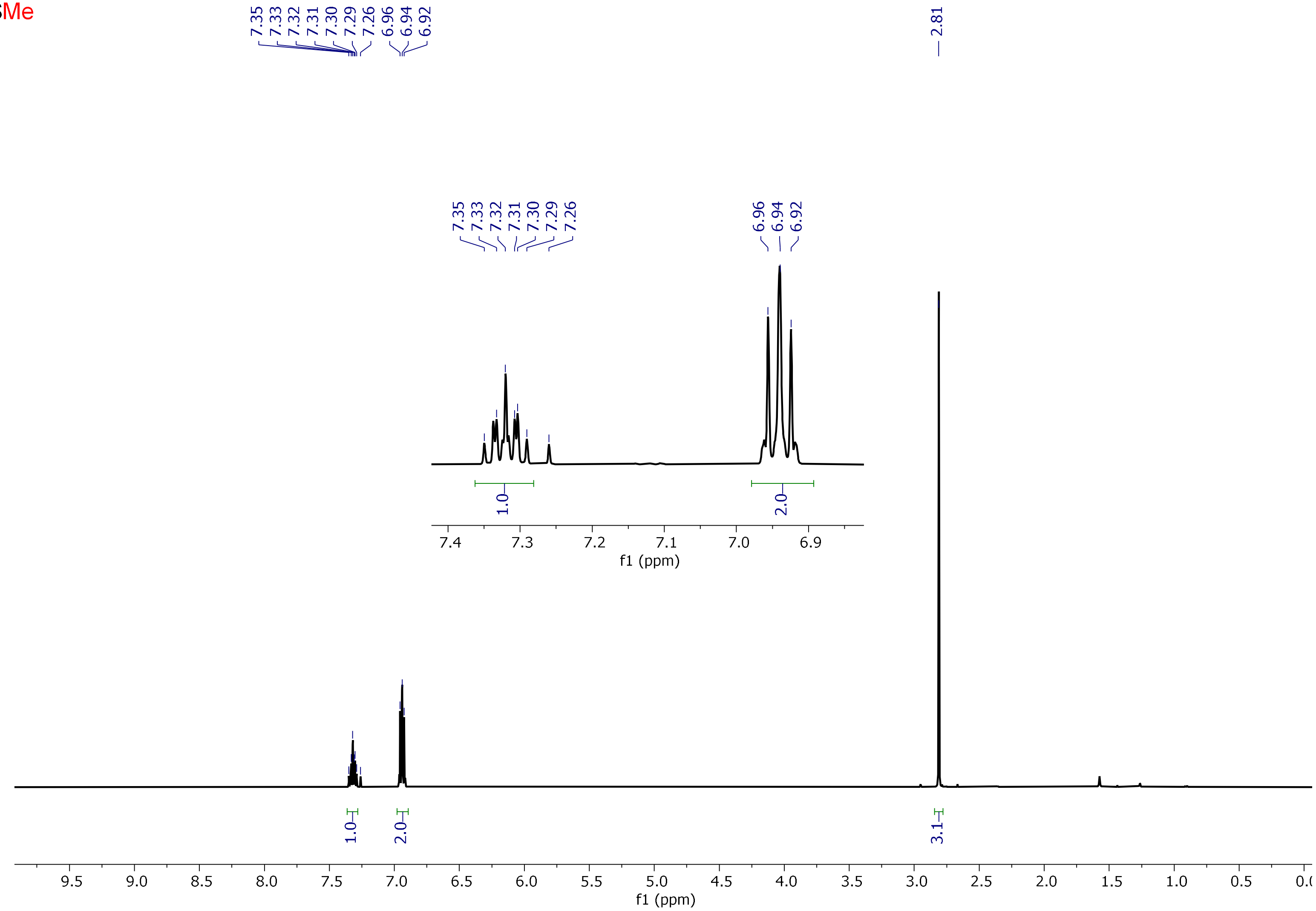

Figure S97. ^{13}C NMR (125 MHz, $CDCl_3$) spectrum for **30i**

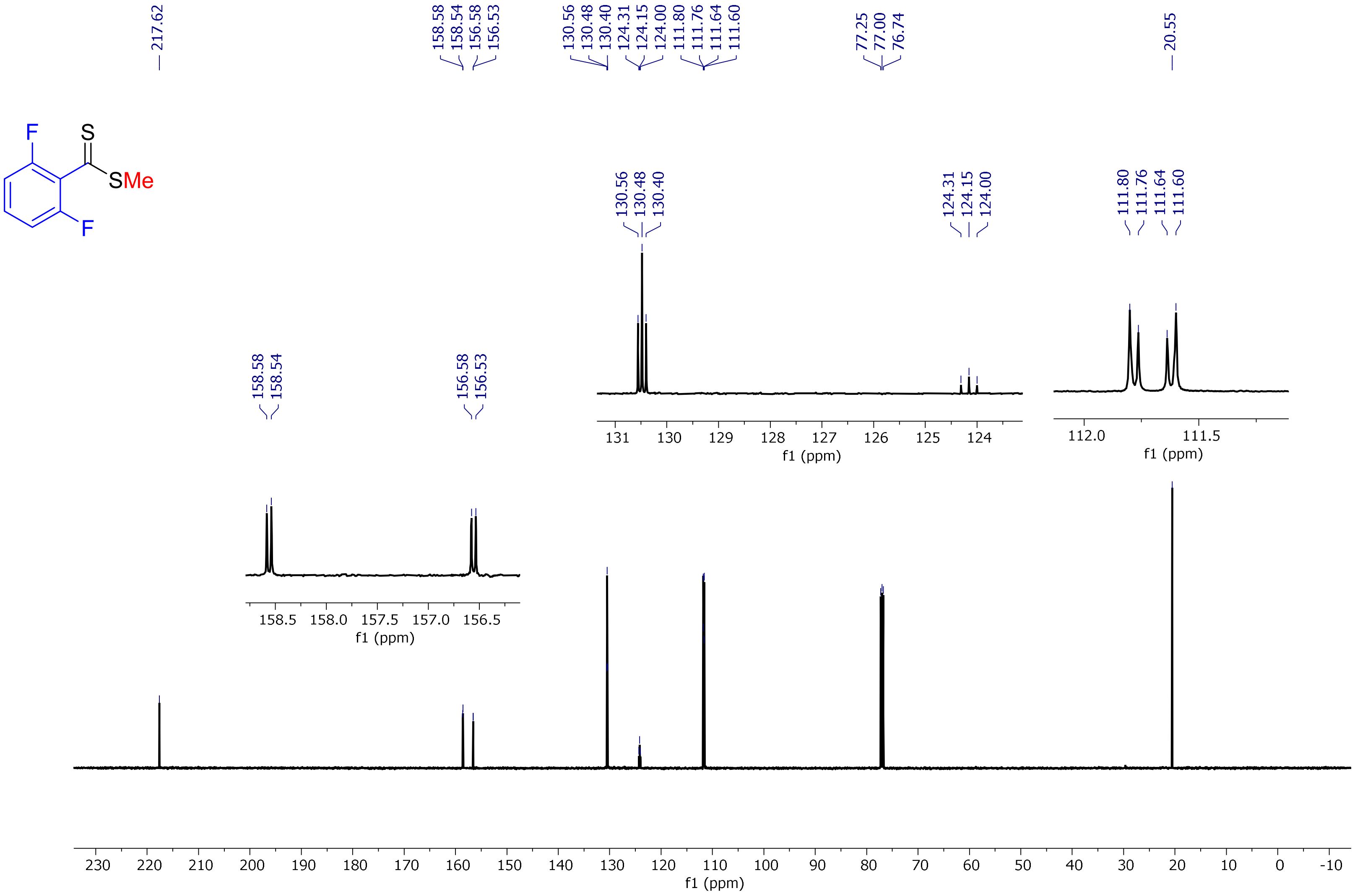

Figure S98. ^{1}H NMR (500 MHz, $CDCl_3$) spectrum for **30j**

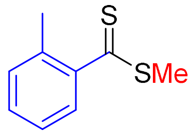

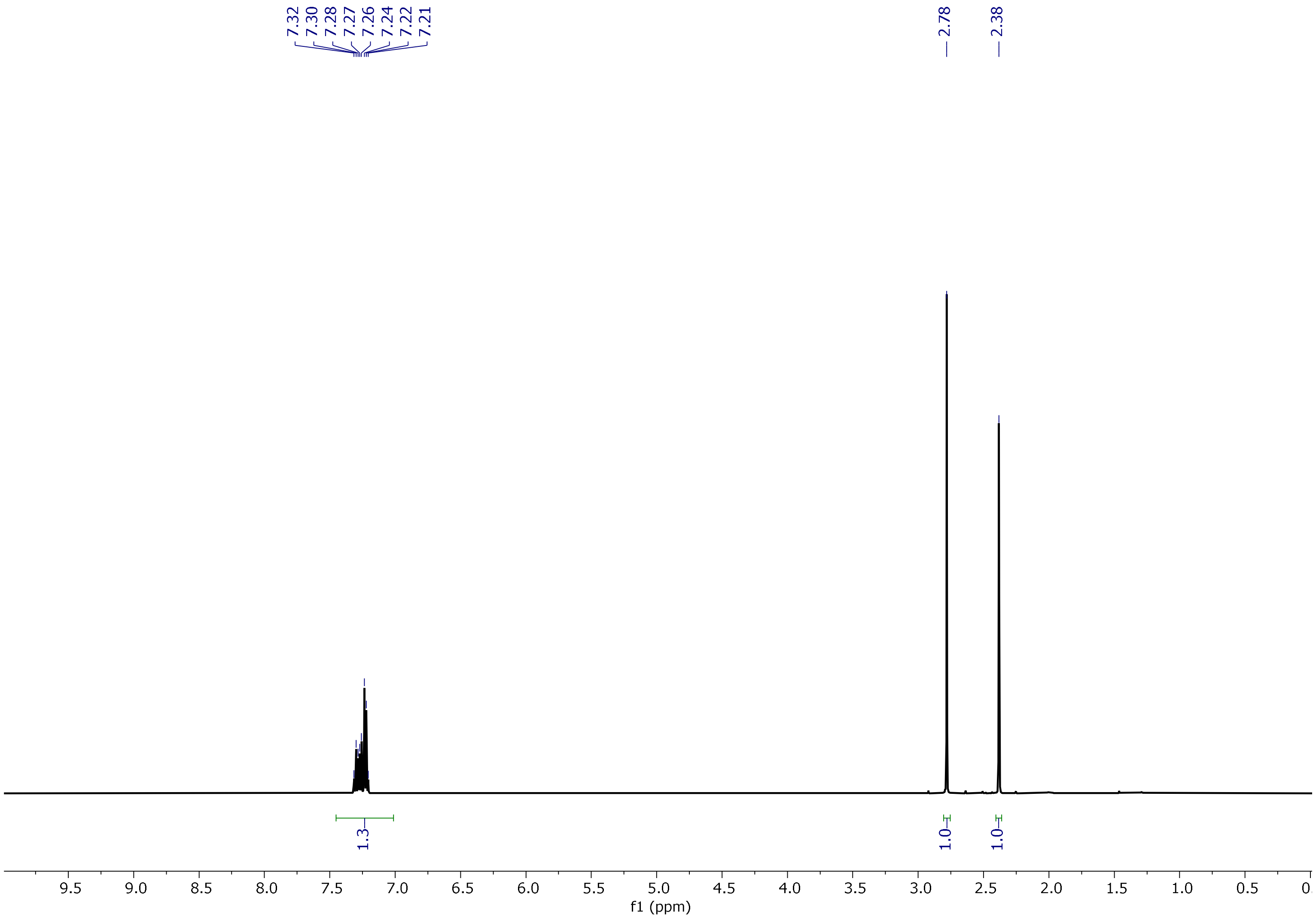

Figure S99. ^{13}C NMR (125 MHz, $CDCl_3$) spectrum for **30j**

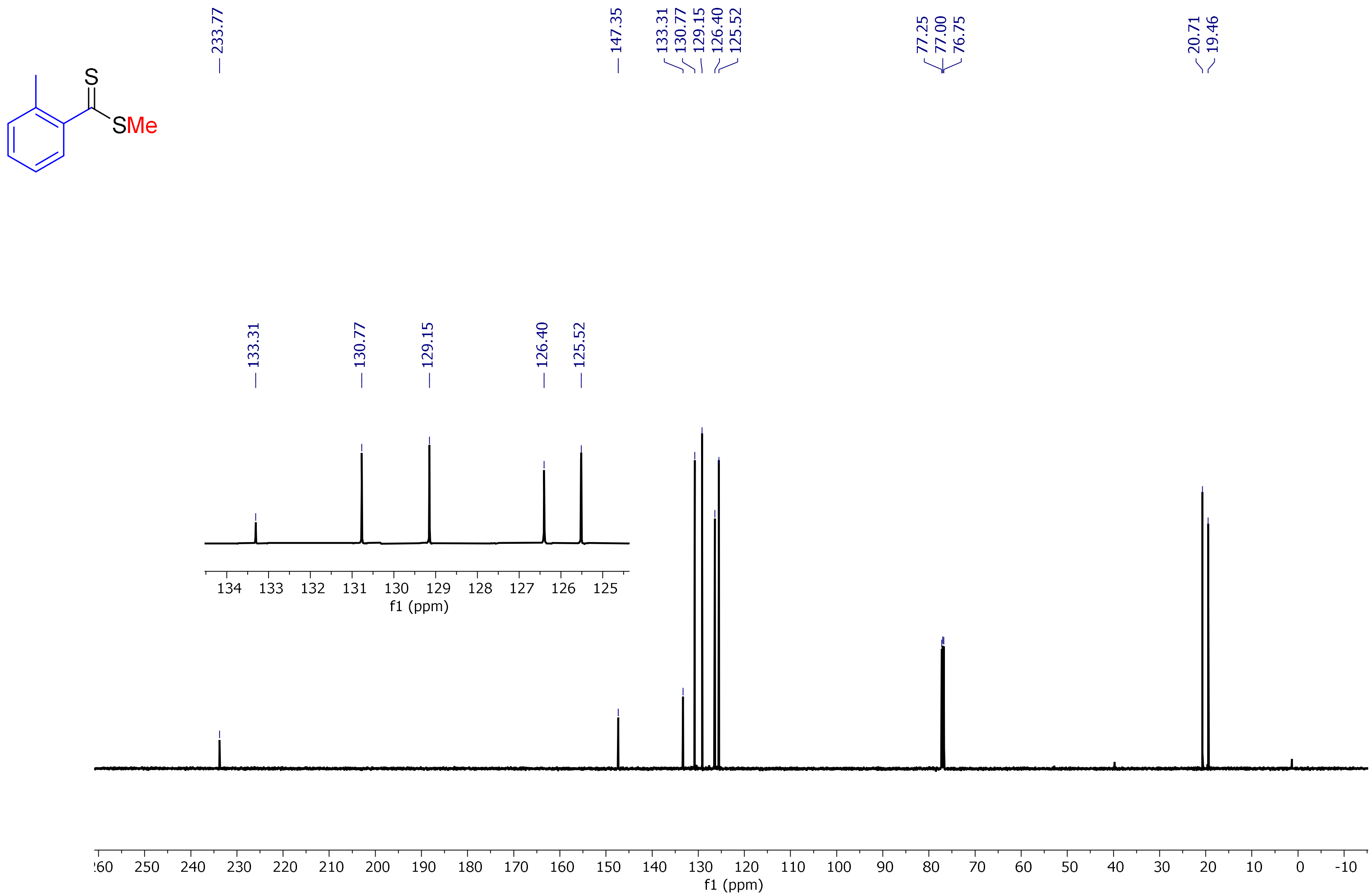

Figure S100. ^{1}H NMR (500 MHz, $CDCl_3$) spectrum for **30k**

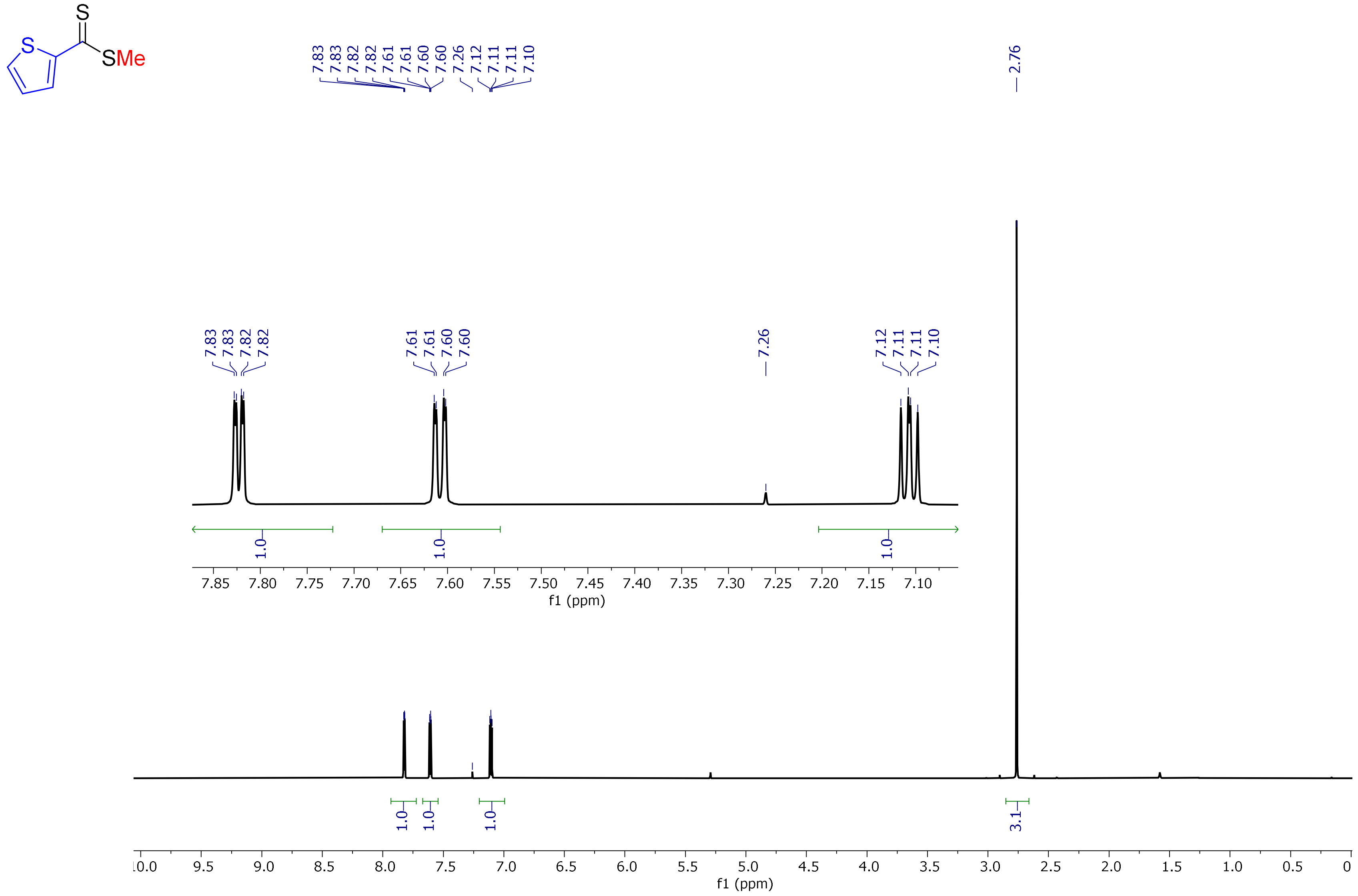

Figure S101. ^{13}C NMR (125 MHz, $CDCl_3$) spectrum for **30k**

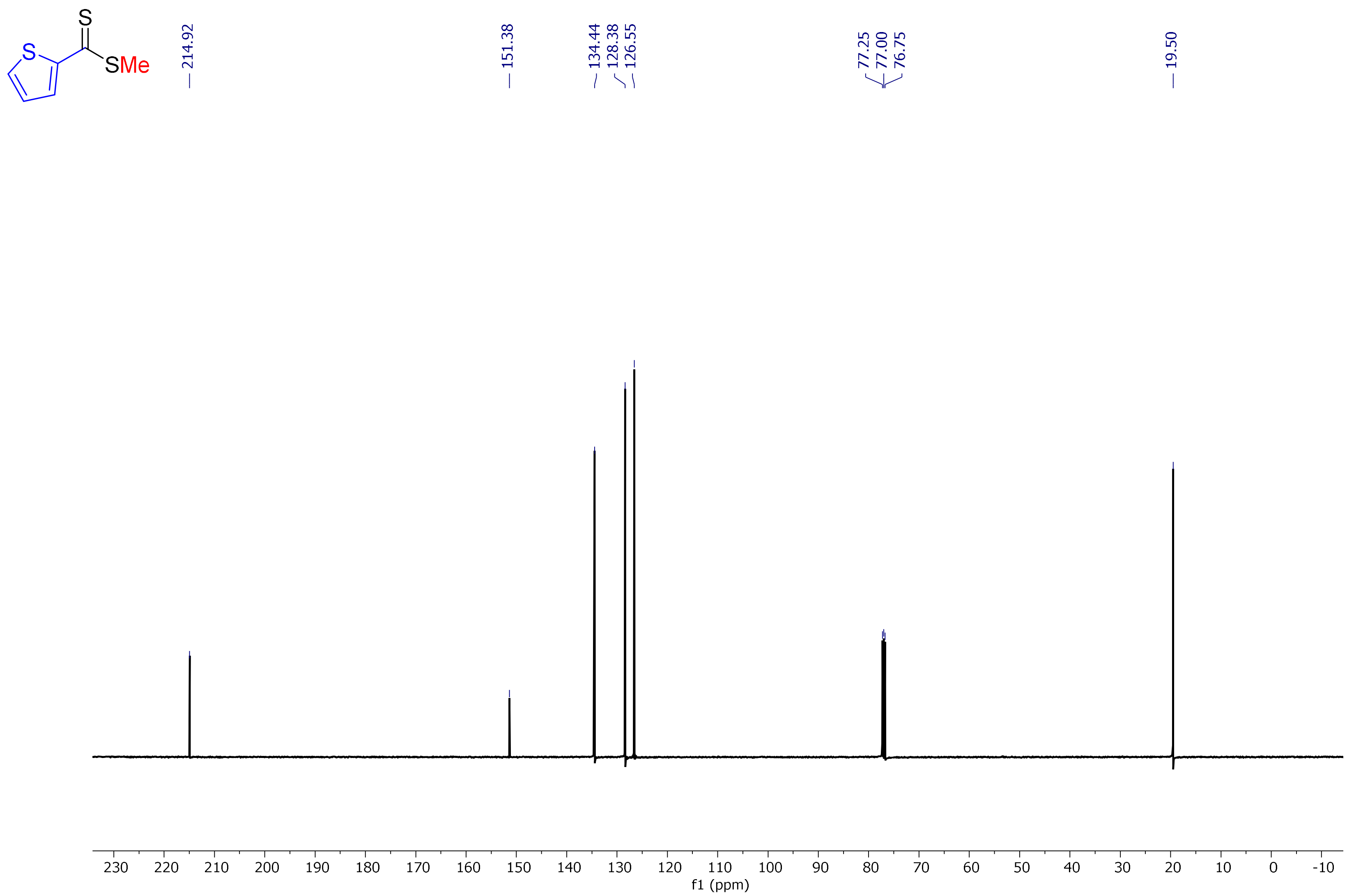

Figure S102. ^{1}H NMR (500 MHz, $CDCl_3$) spectrum for **30l**

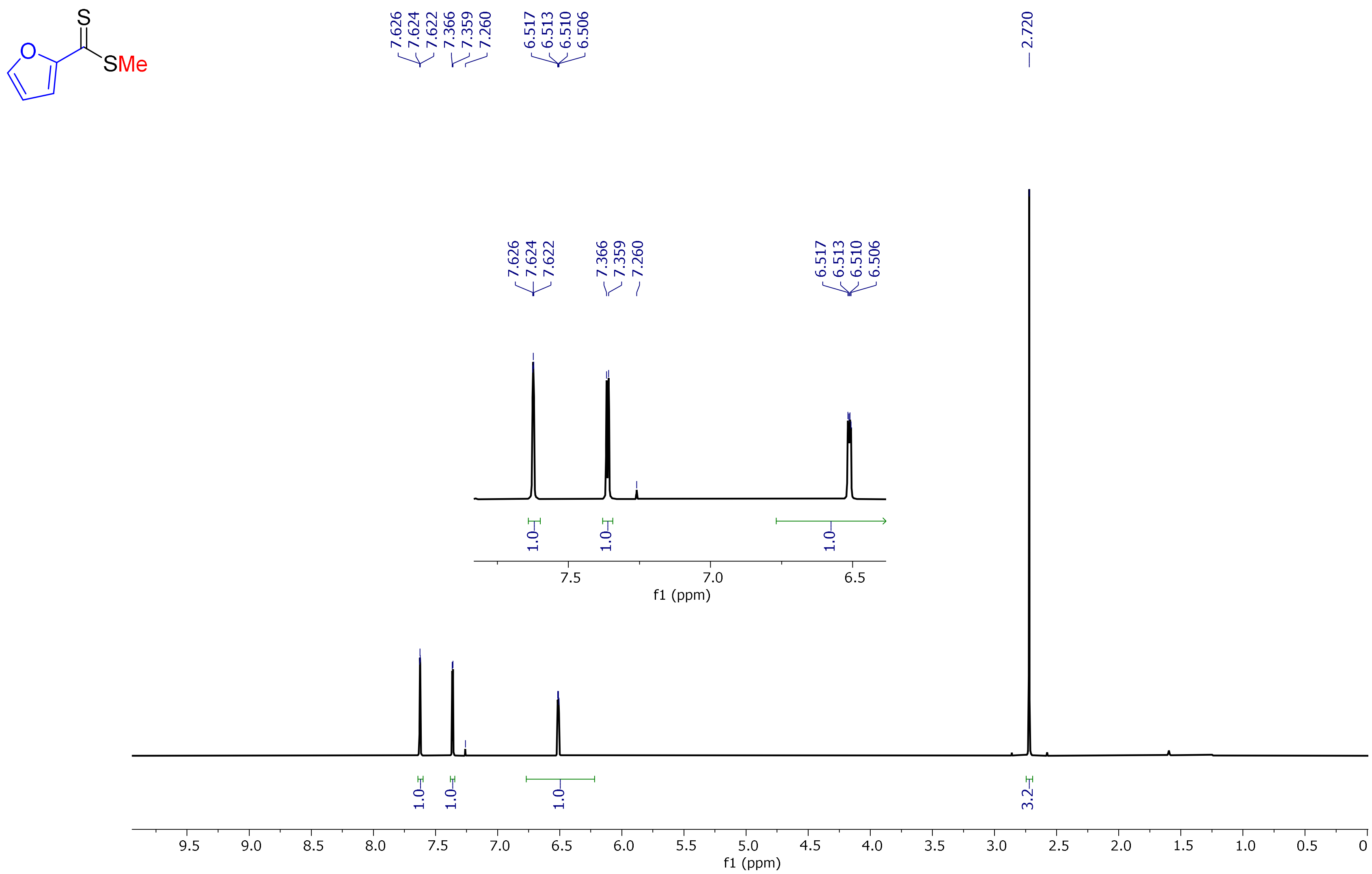

Figure S103. ^{13}C NMR (125 MHz, $CDCl_3$) spectrum for **30l**

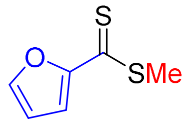

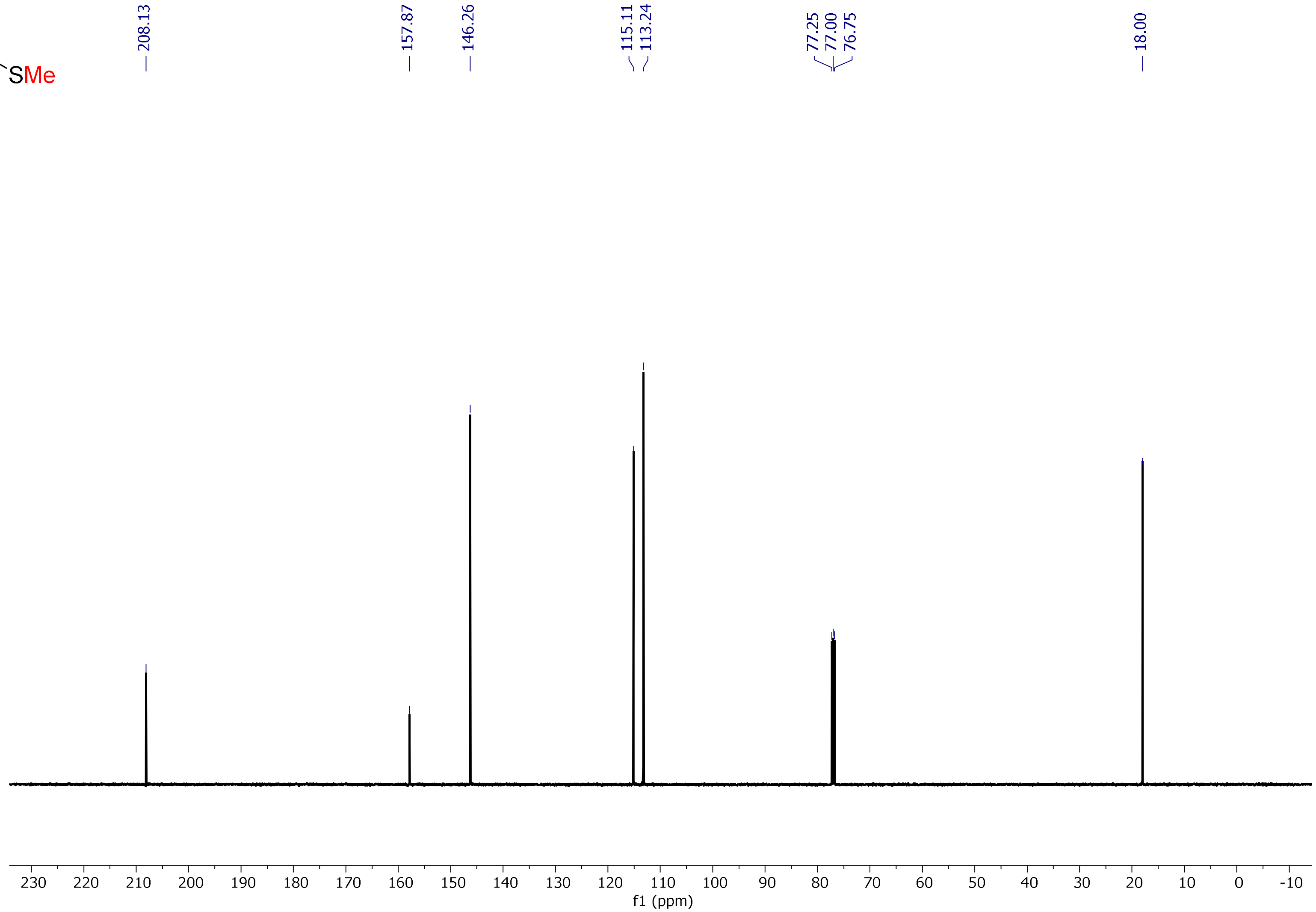

Figure S104. ^{1}H NMR (500 MHz, $CDCl_3$) spectrum for **31a**

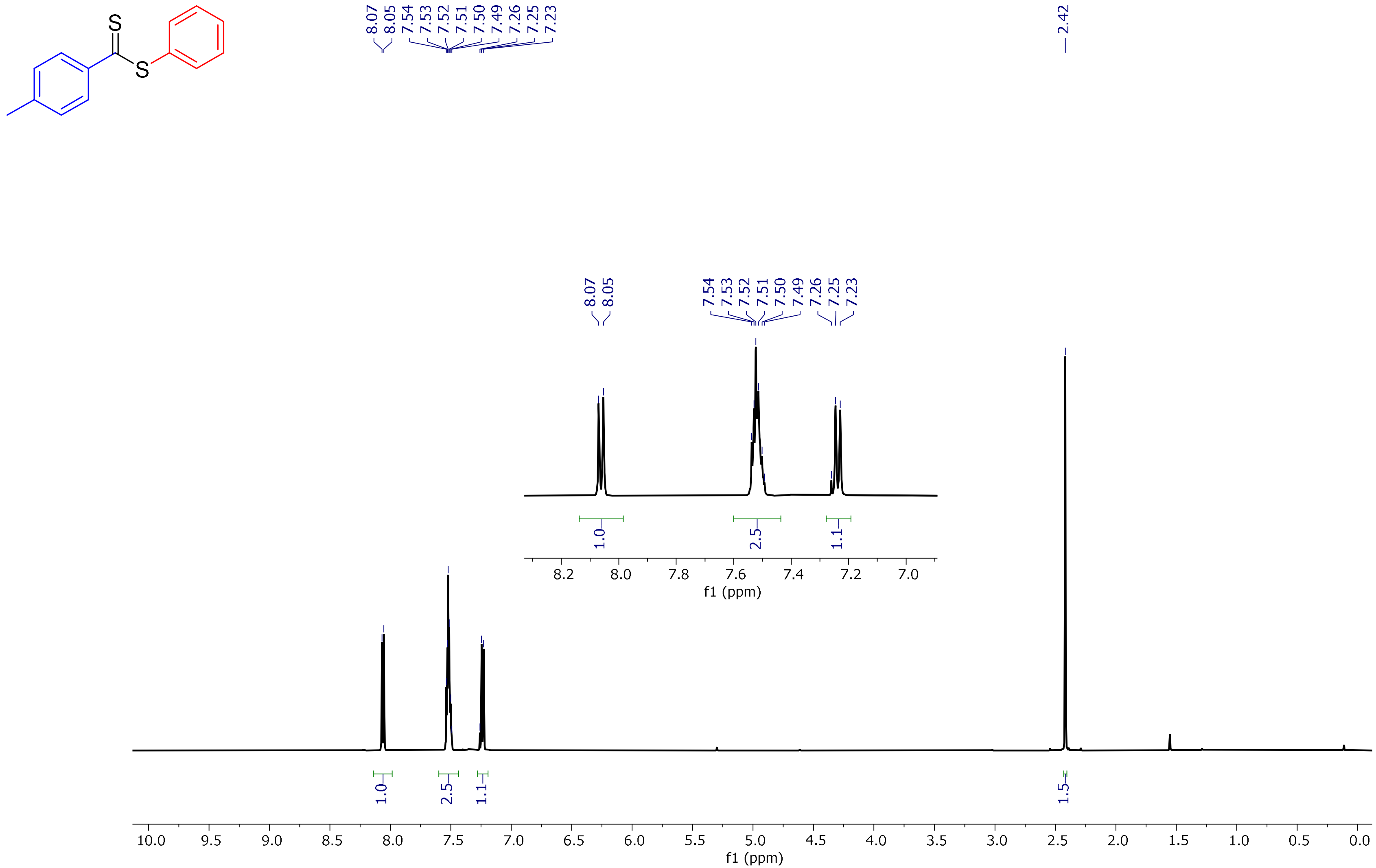

Figure S105. ^{13}C NMR (125 MHz, $CDCl_3$) spectrum for **31a**

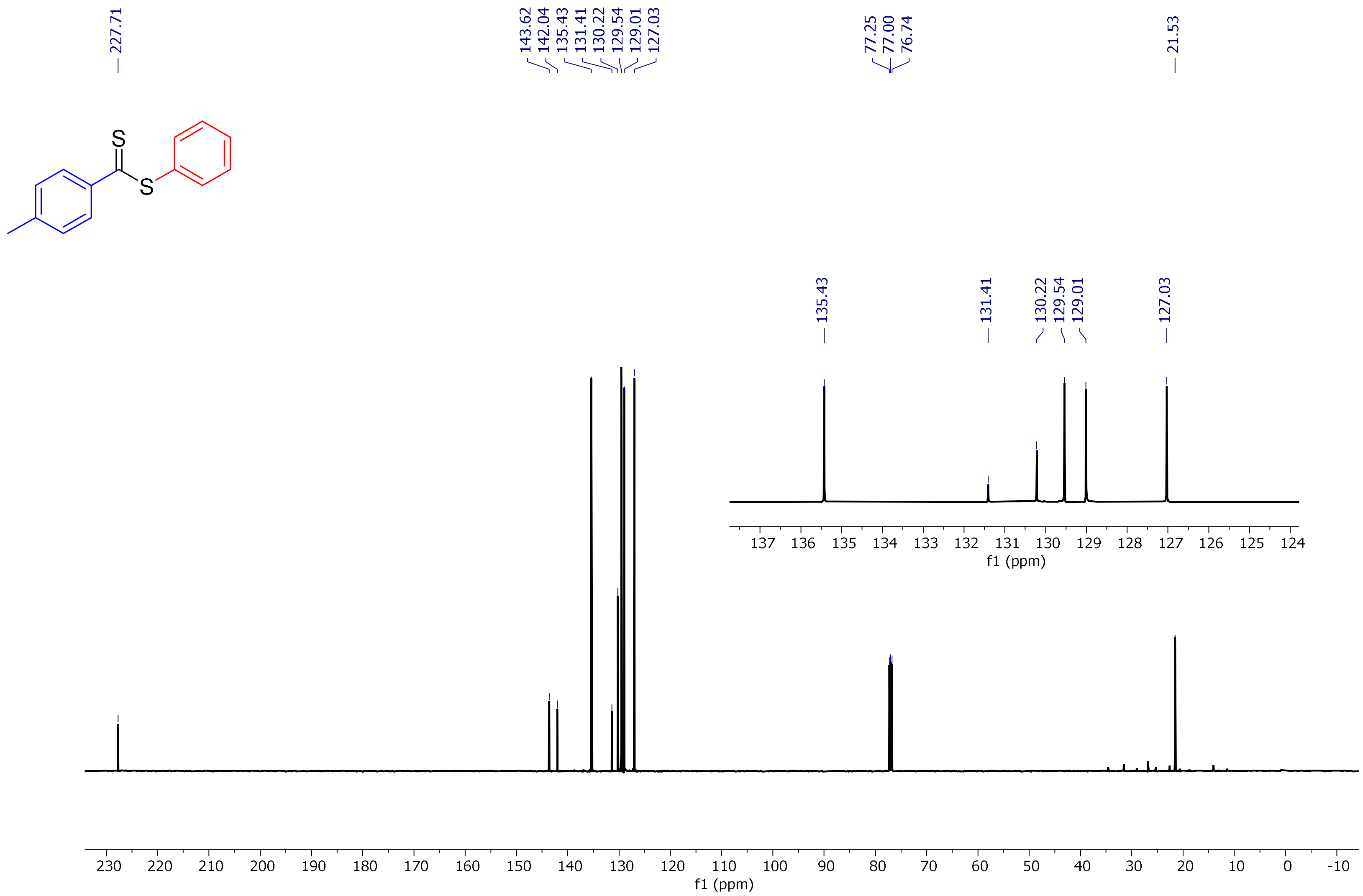

Figure S106. ^{1}H NMR (500 MHz, $CDCl_3$) spectrum for **31c**

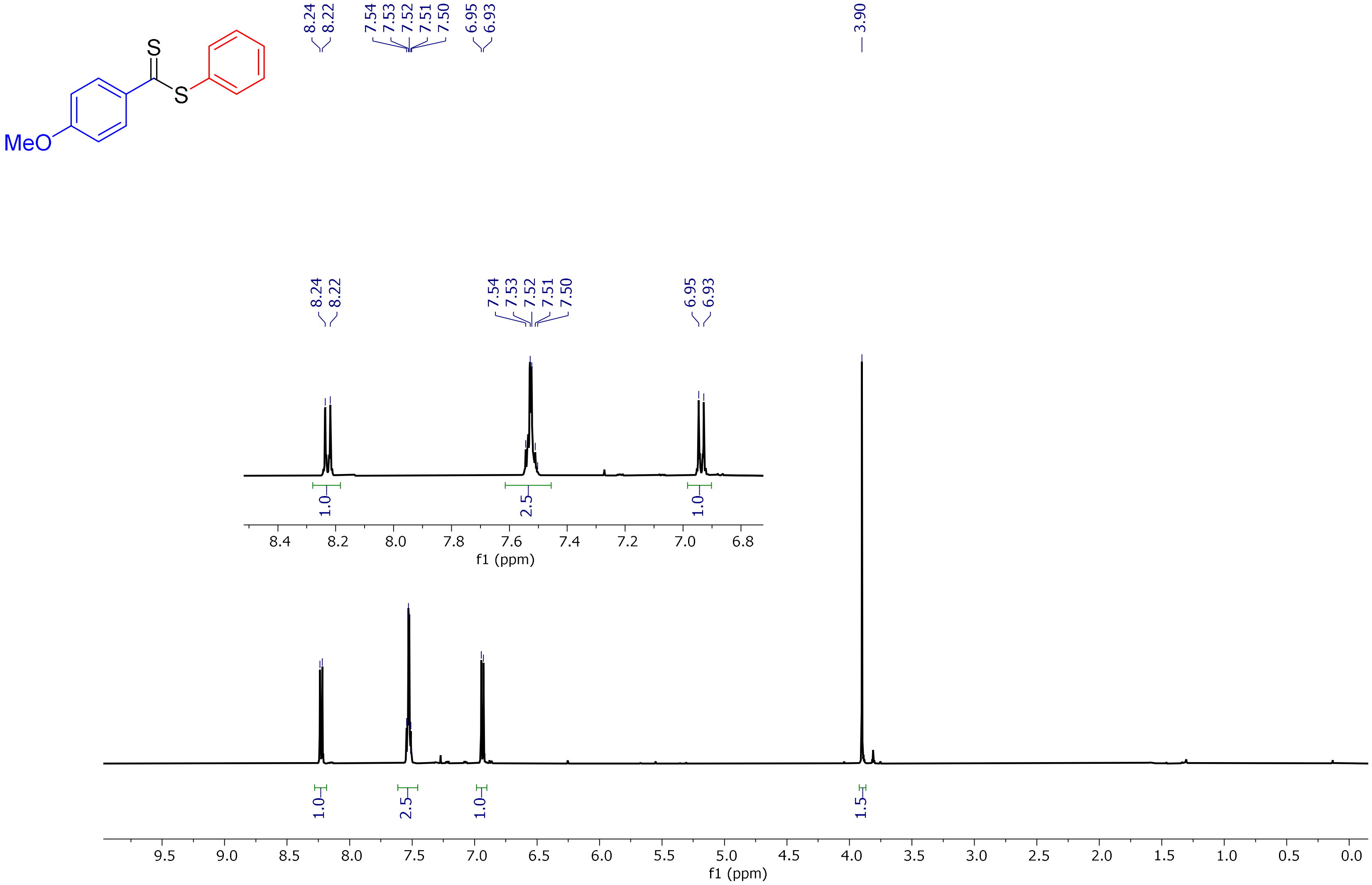

Figure S107. ^{13}C NMR (125 MHz, CDCl$_3$) spectrum for **31c**

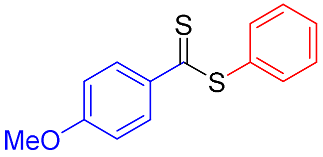

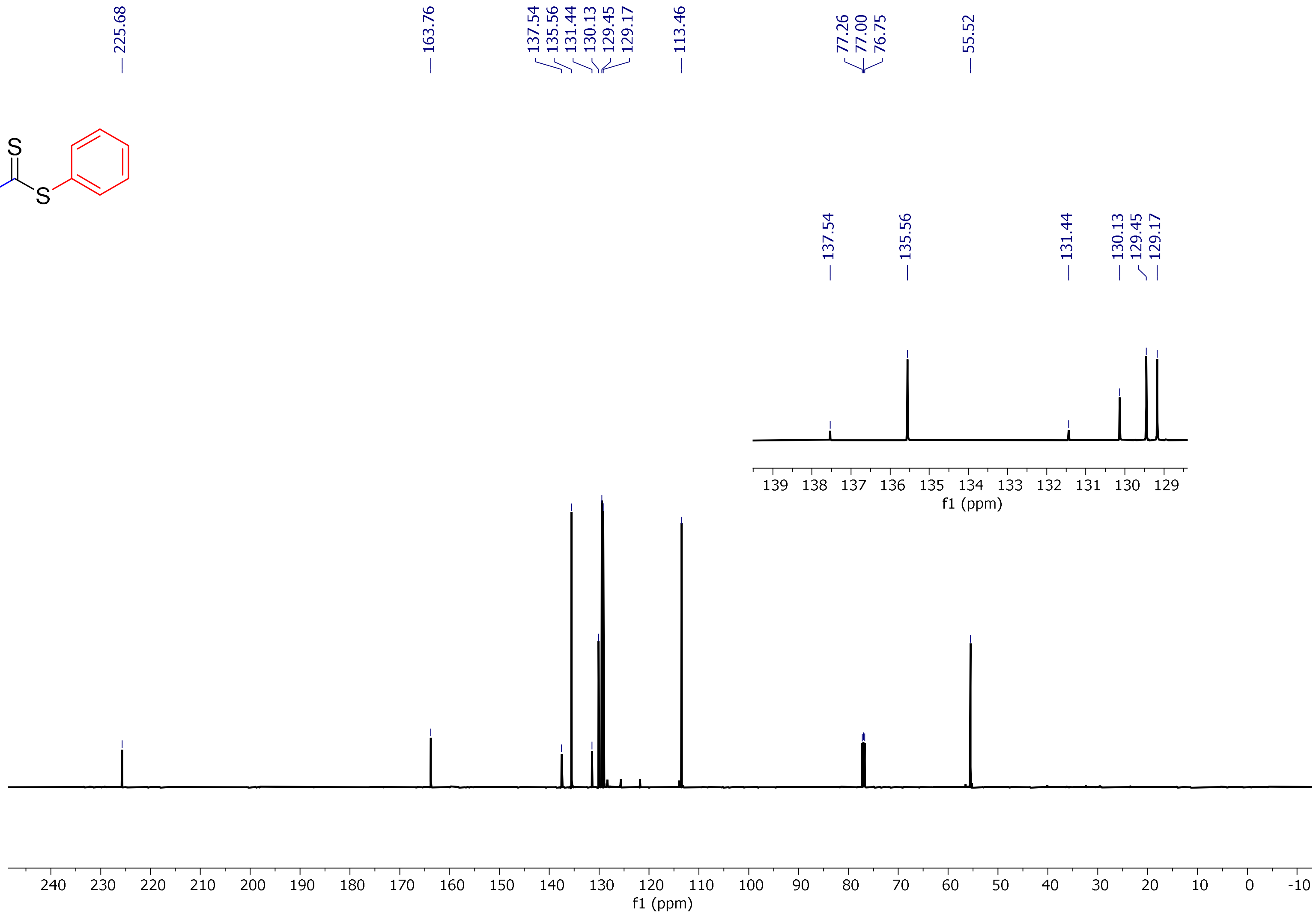

Figure S108. ^{1}H NMR (500 MHz, $CDCl_3$) spectrum for **31d**

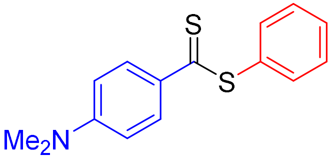

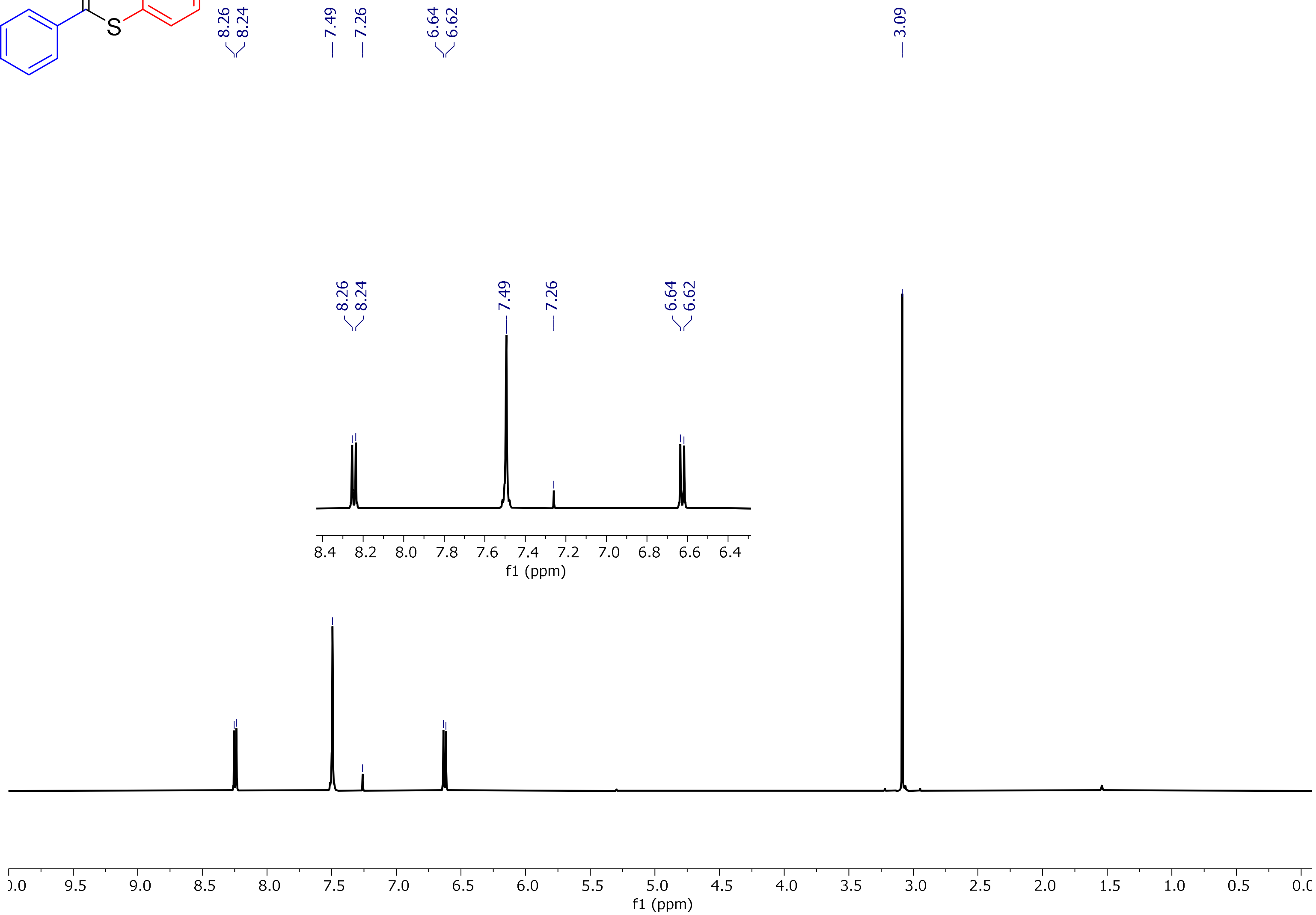

Figure S109. ^{13}C NMR (125 MHz, $CDCl_3$) spectrum for **31d**

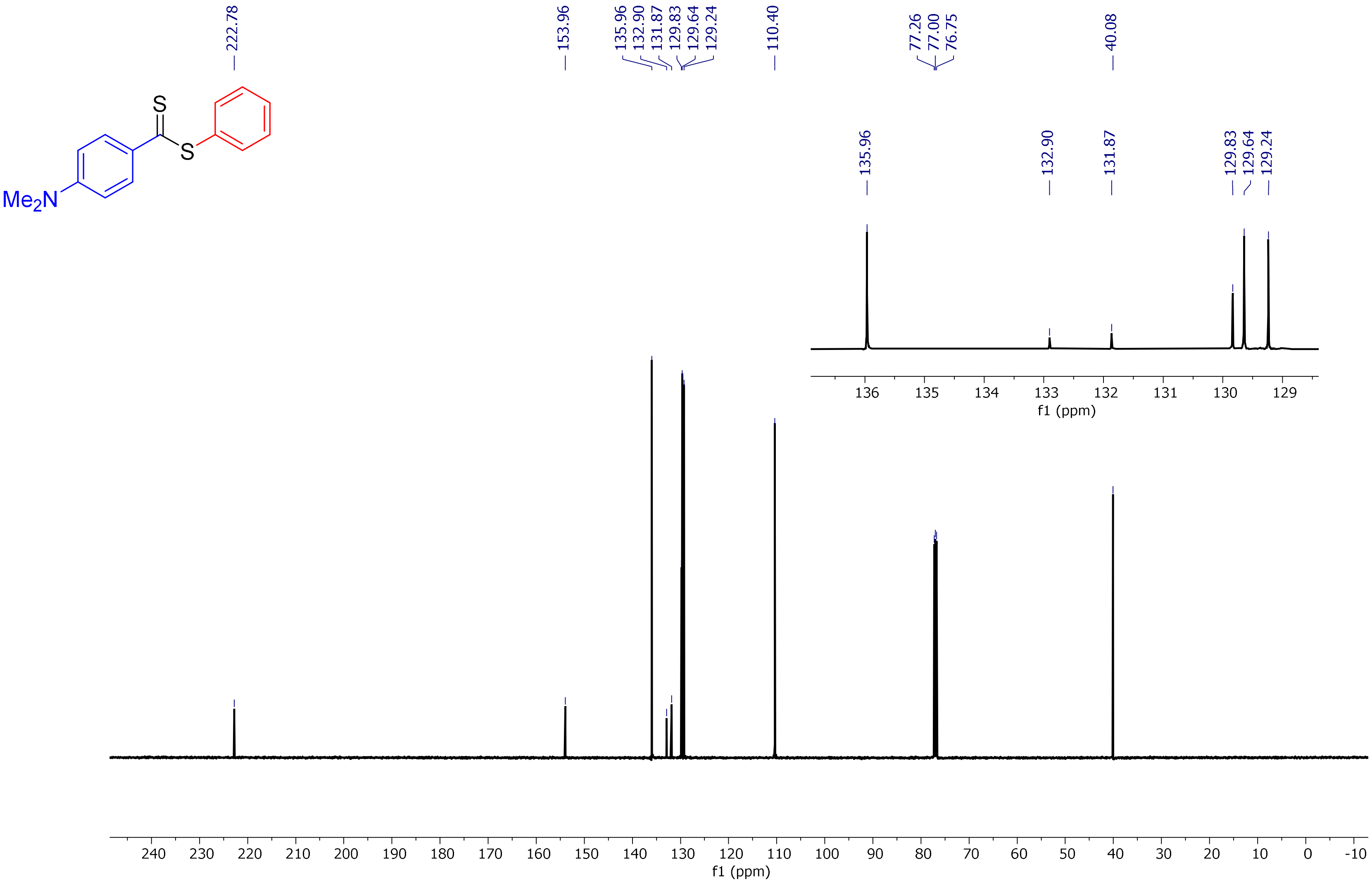

Figure S110. ^{1}H NMR (500 MHz, $CDCl_3$) spectrum for **31g**

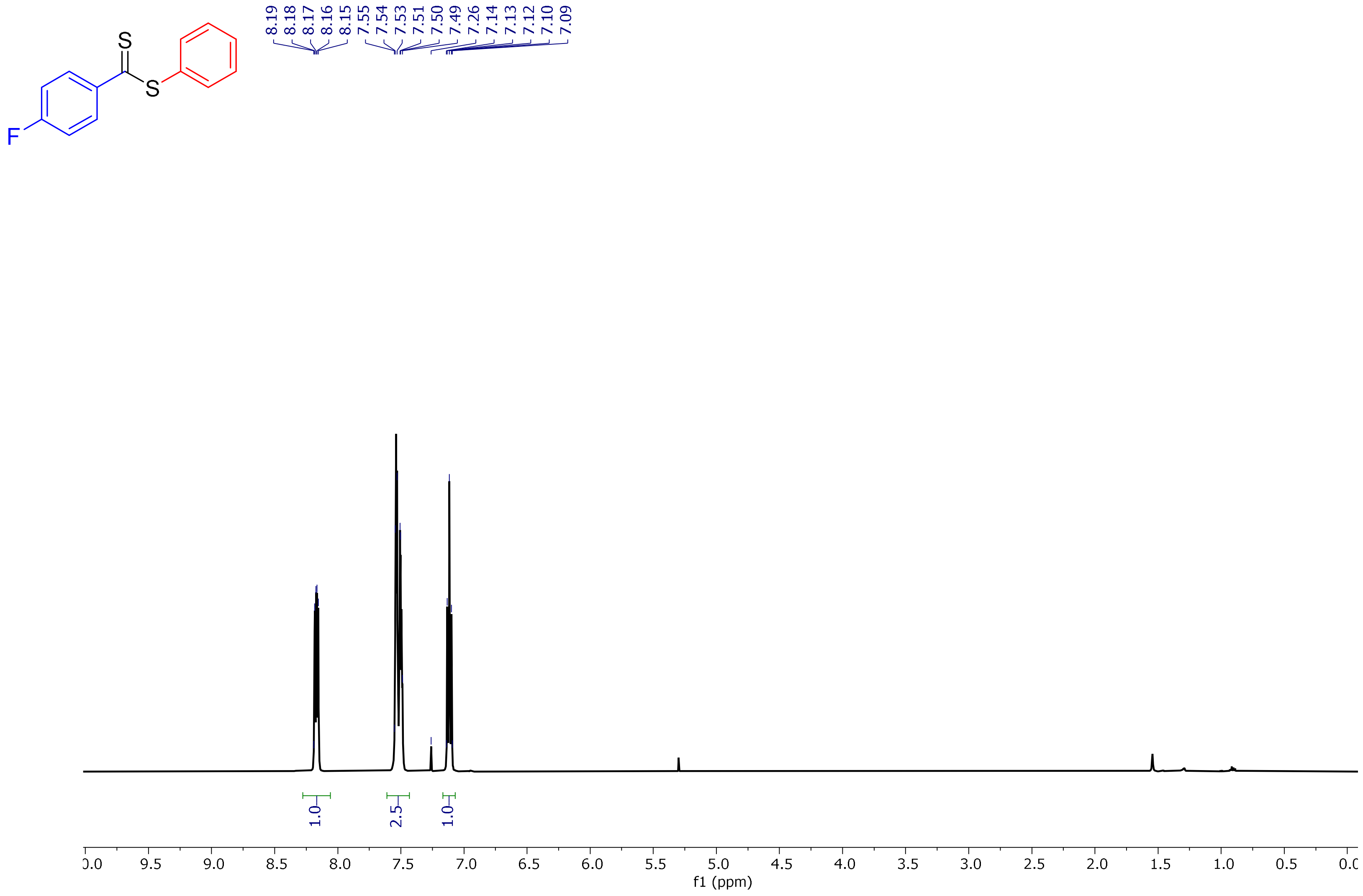

Figure S111. ^{13}C NMR (125 MHz, $CDCl_3$) spectrum for **31g**

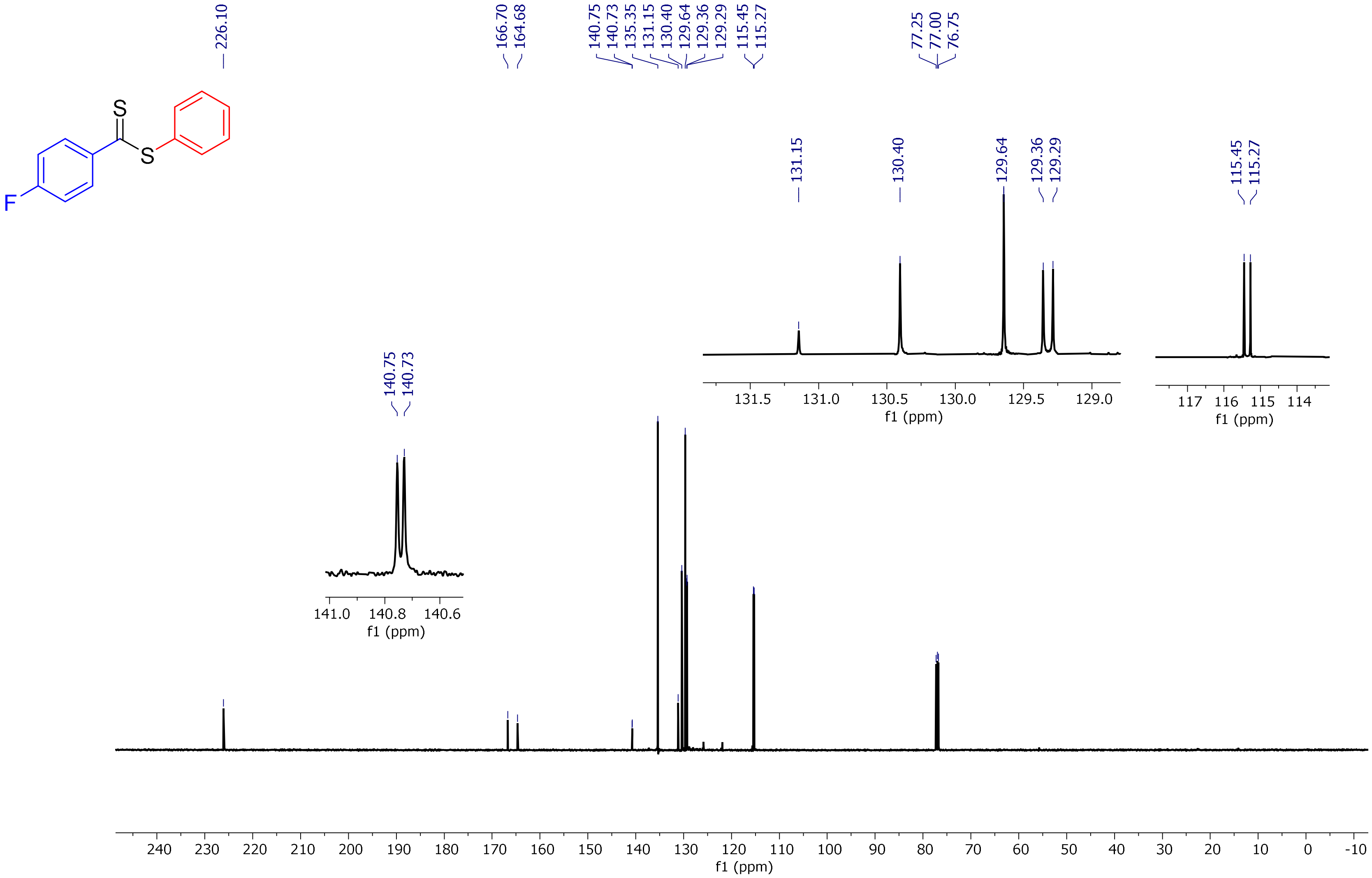

Figure S112. ^{1}H NMR (500 MHz, $CDCl_3$) spectrum for **31i**

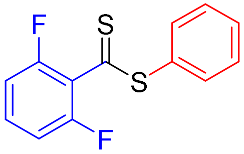

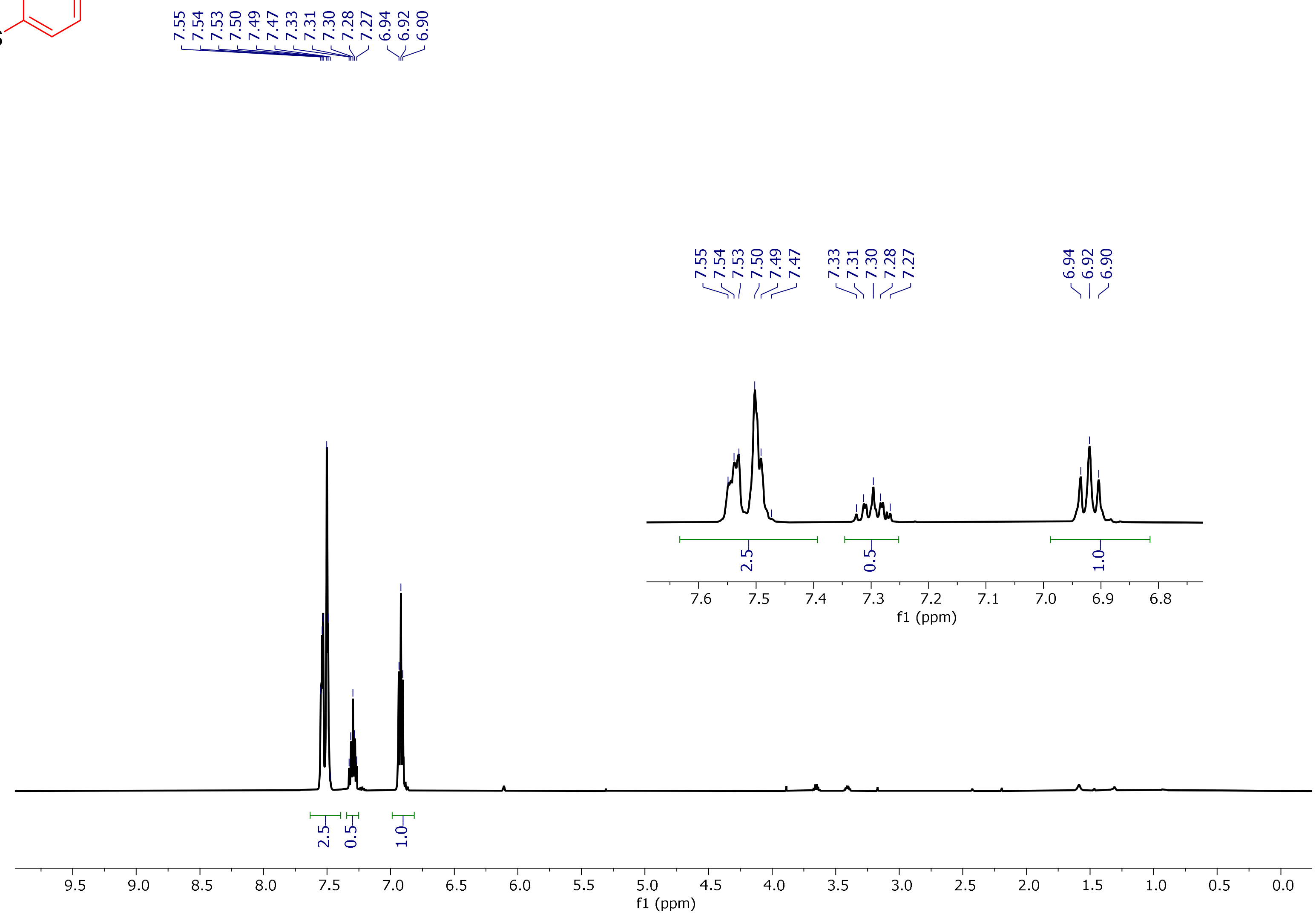

Figure S113. ^{13}C NMR (125 MHz, $CDCl_3$) spectrum for **31i**

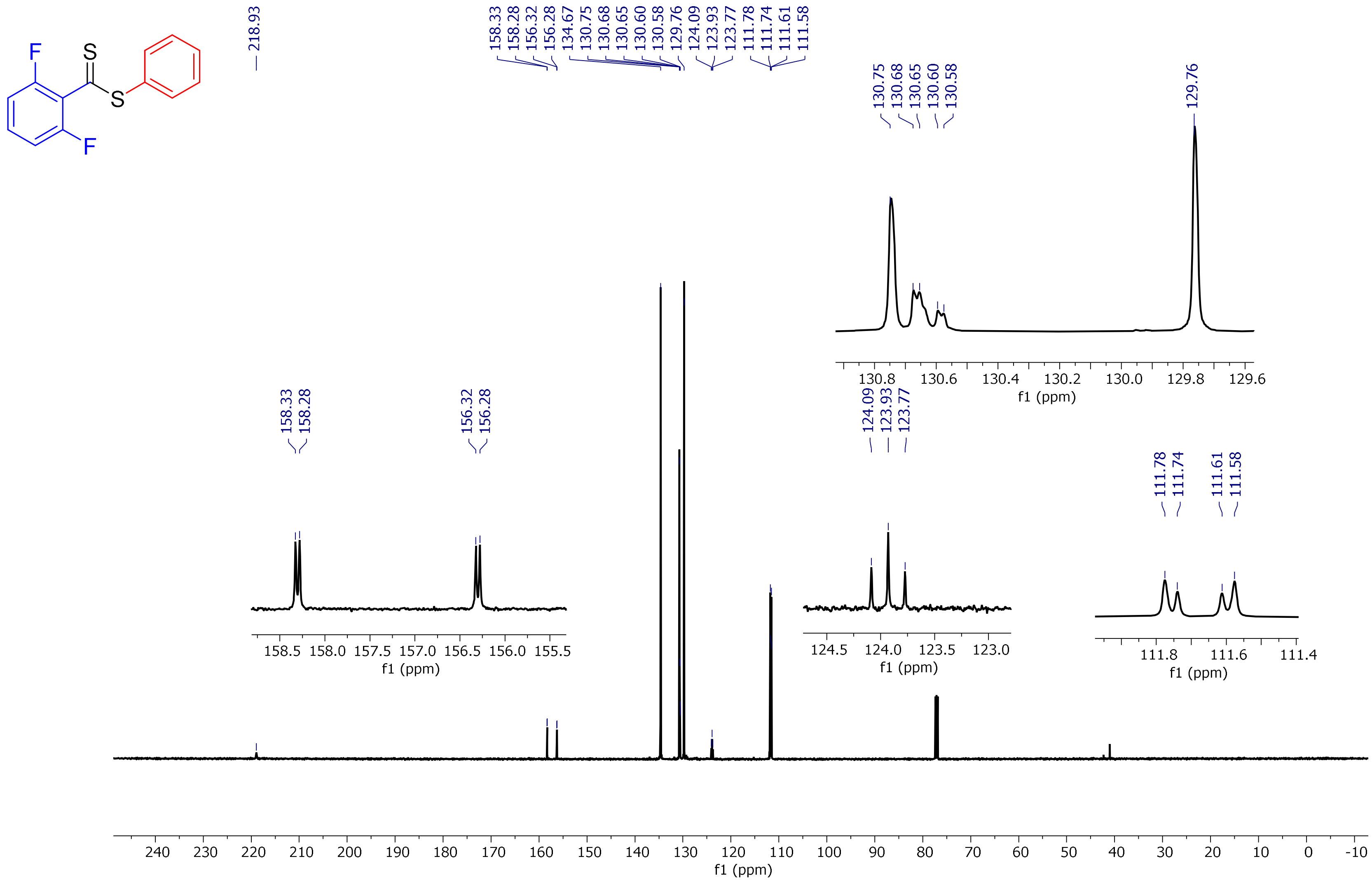

Figure S114. ^{1}H NMR (500 MHz, $CDCl_3$) spectrum for **31j**

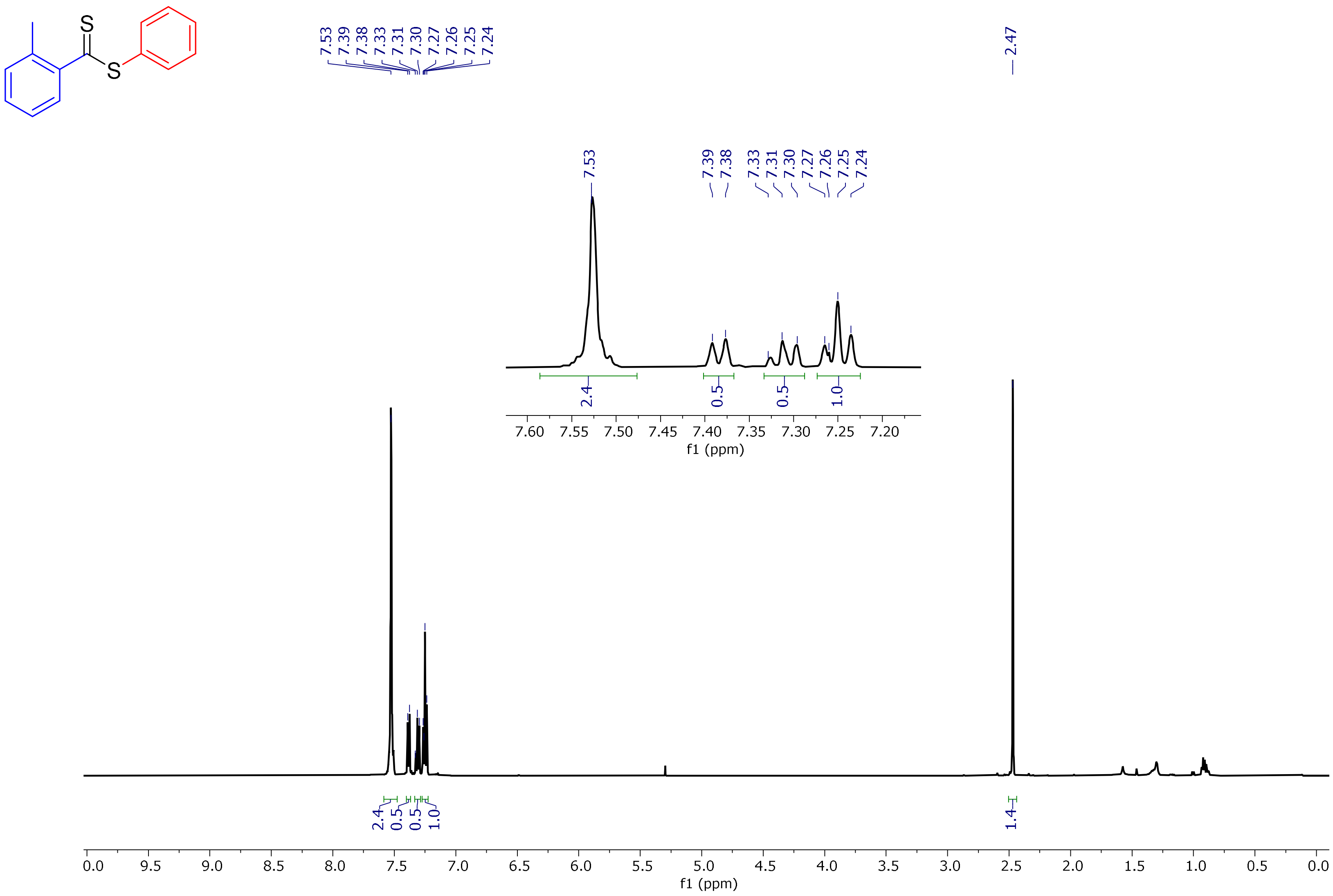

Figure S115. ^{13}C NMR (125 MHz, $CDCl_3$) spectrum for **31j**

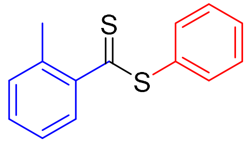

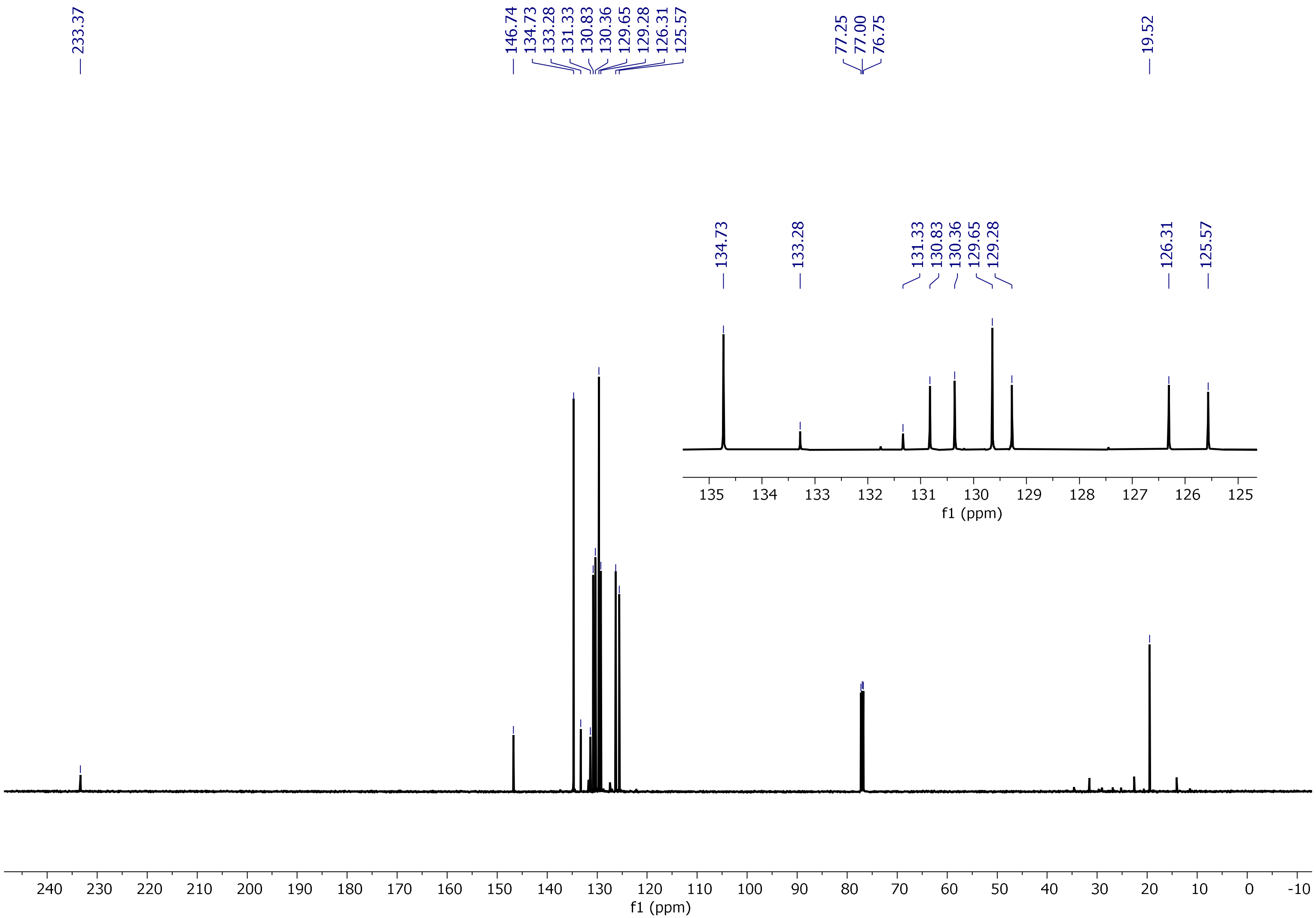

Figure S116. ^{1}H NMR (500 MHz, $CDCl_3$) spectrum for **31k**

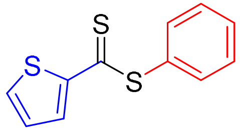

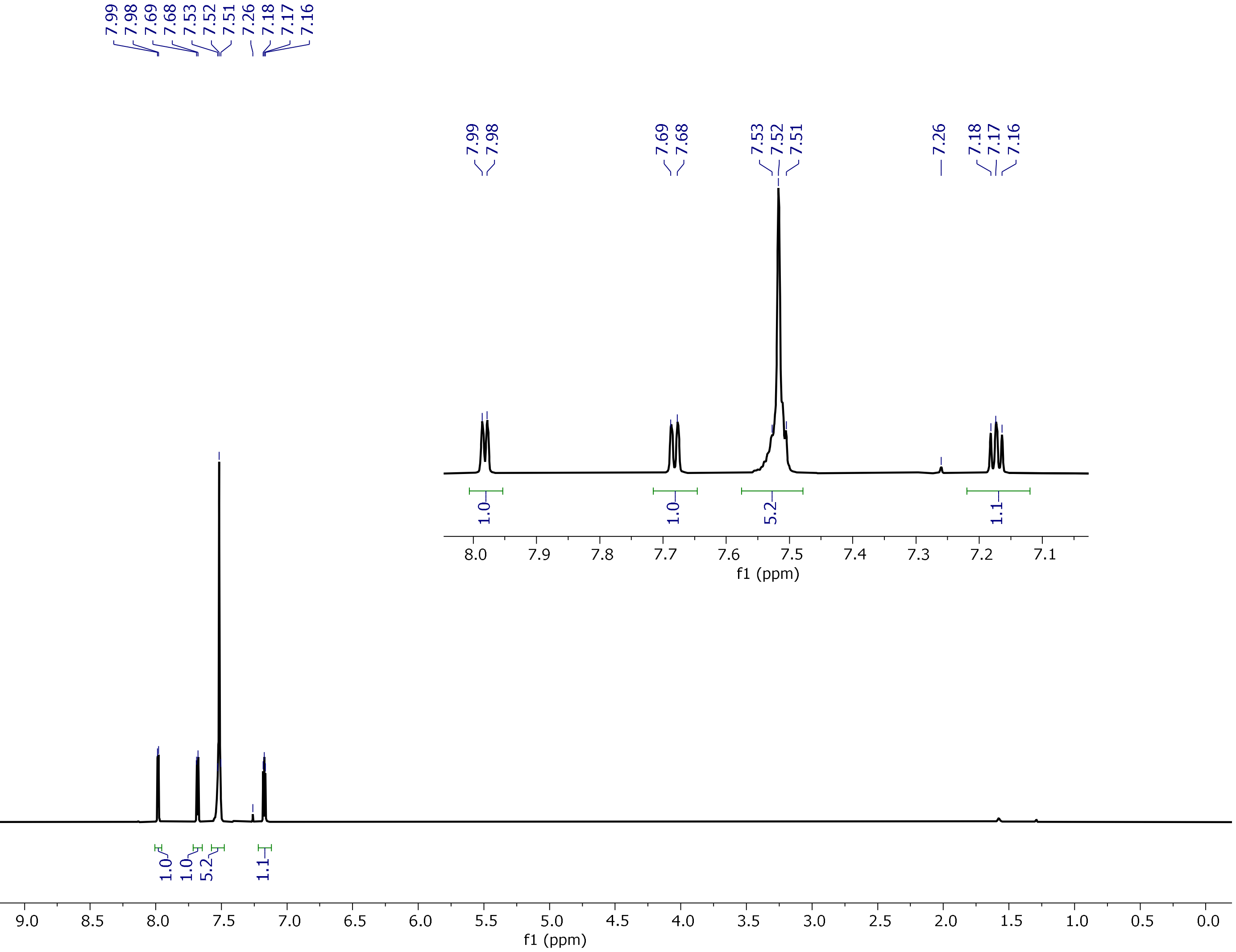

Figure S117. ^{13}C NMR (125 MHz, $CDCl_3$) spectrum for **31k**

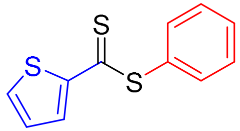

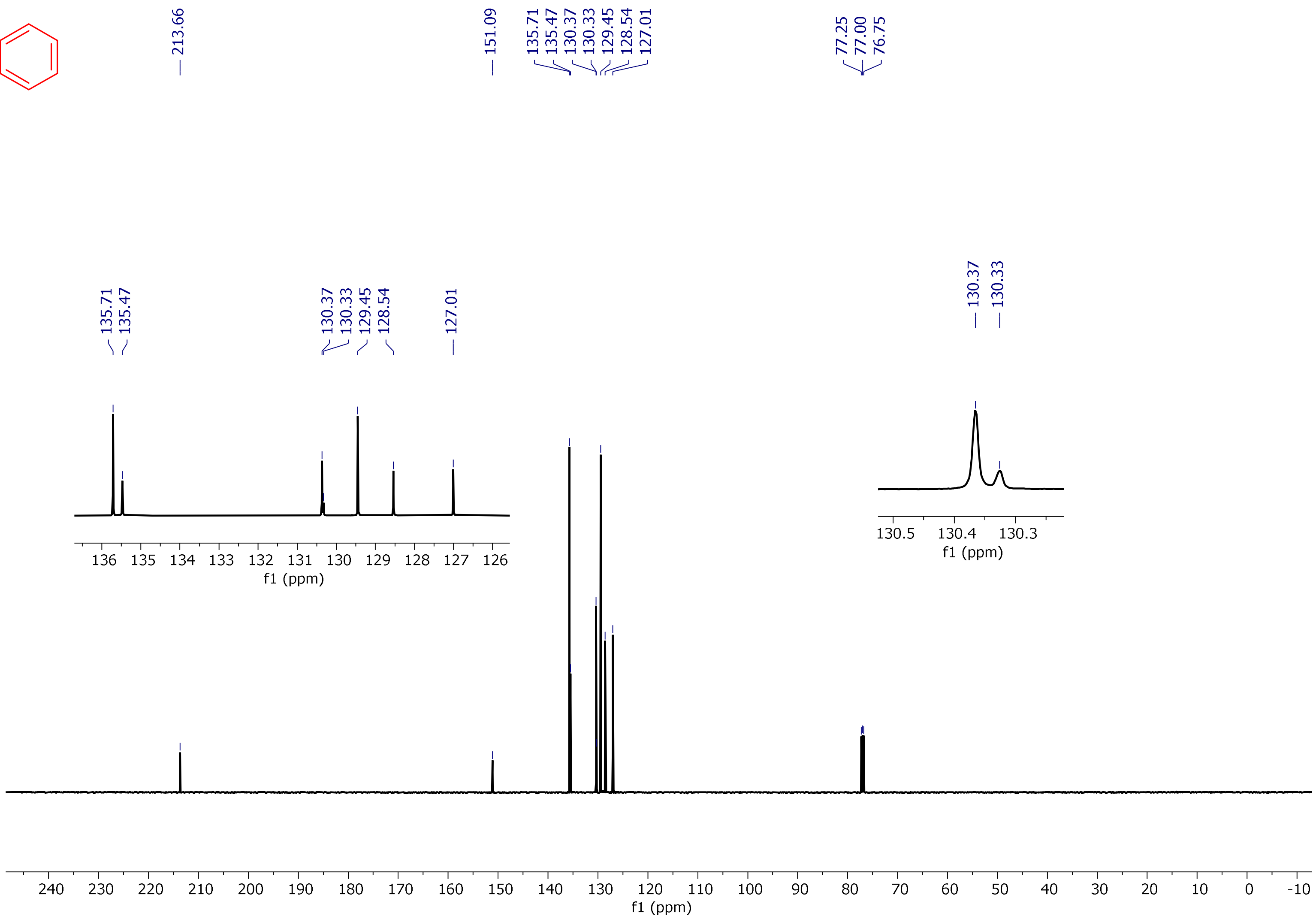

Figure S118. ^{1}H NMR (500 MHz, $CDCl_3$) spectrum for **32a**

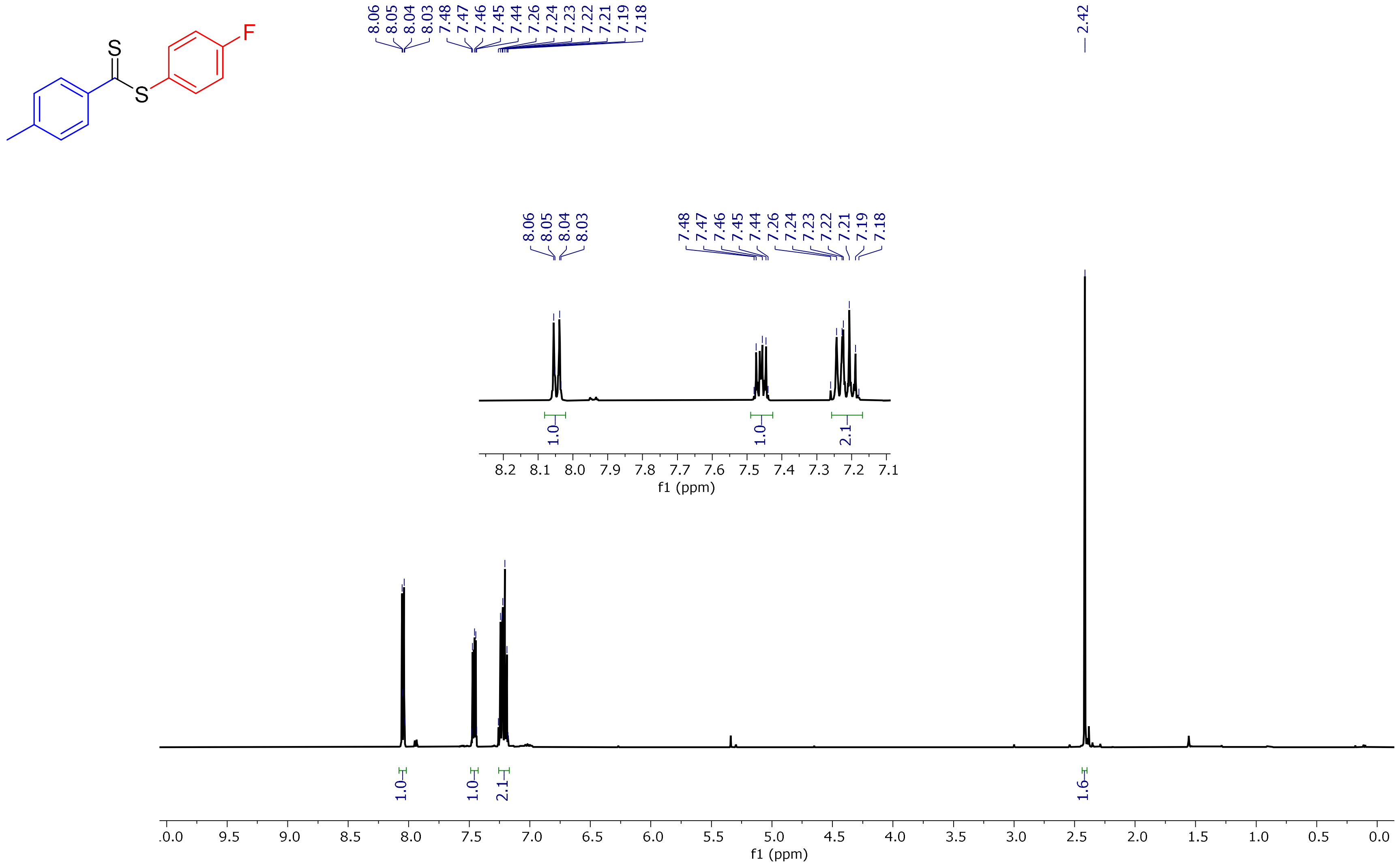

Figure S119. ^{13}C NMR (125 MHz, $CDCl_3$) spectrum for **32a**

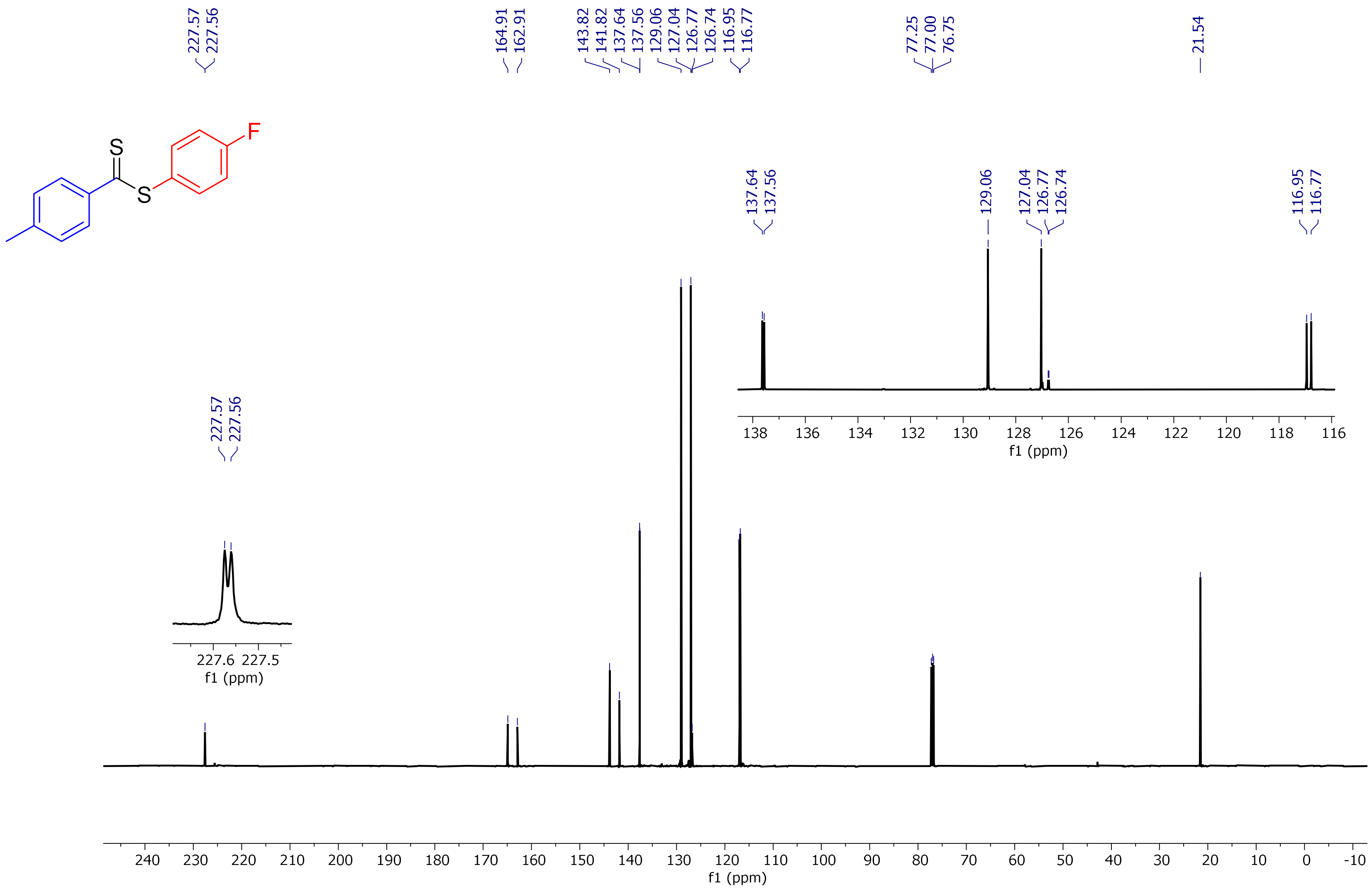

Figure S120. ^{1}H NMR (500 MHz, $CDCl_3$) spectrum for **33a**

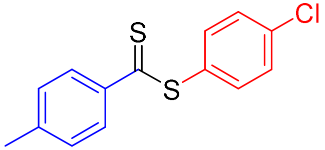

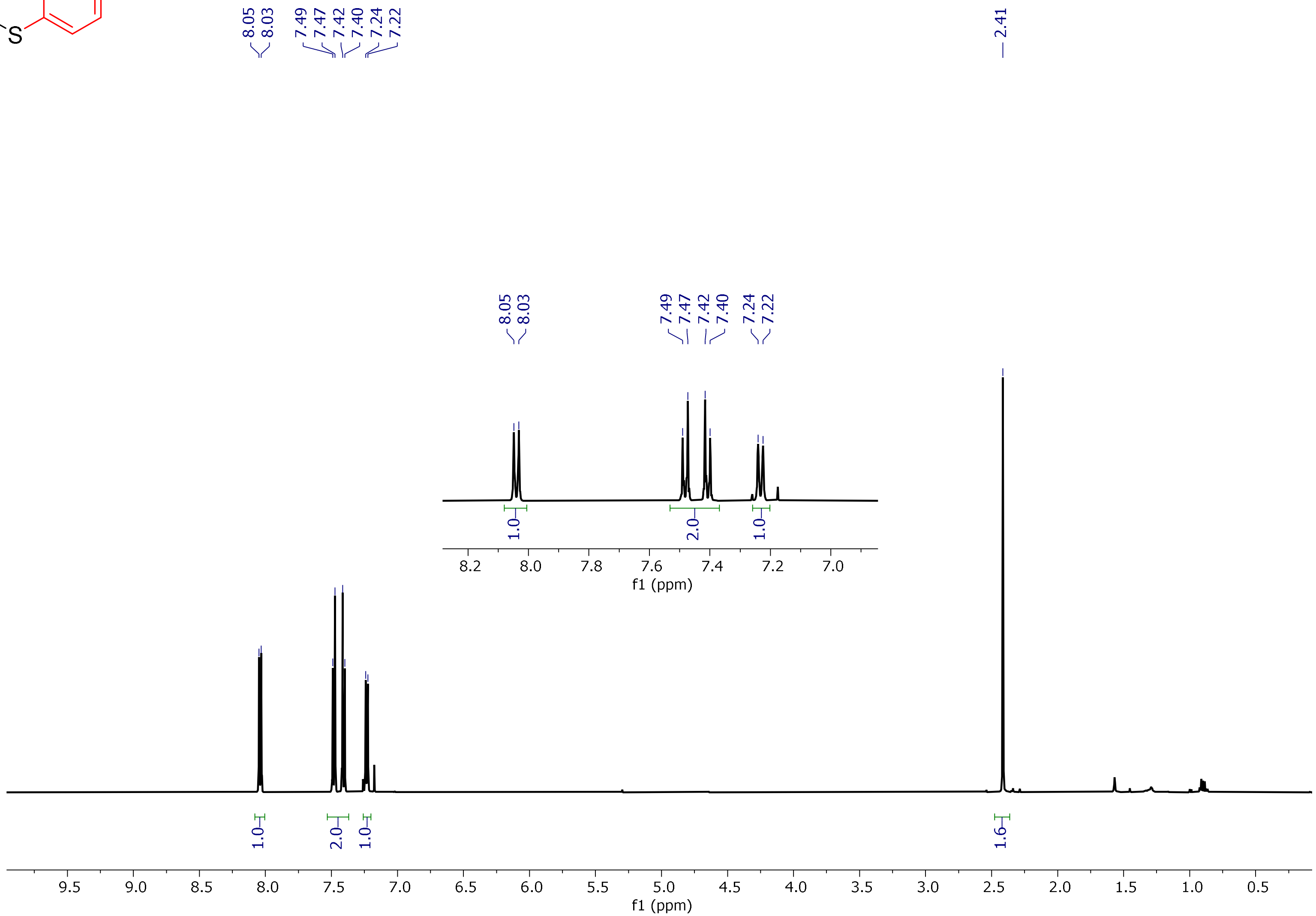

Figure S121. ^{13}C NMR (125 MHz, $CDCl_3$) spectrum for **33a**

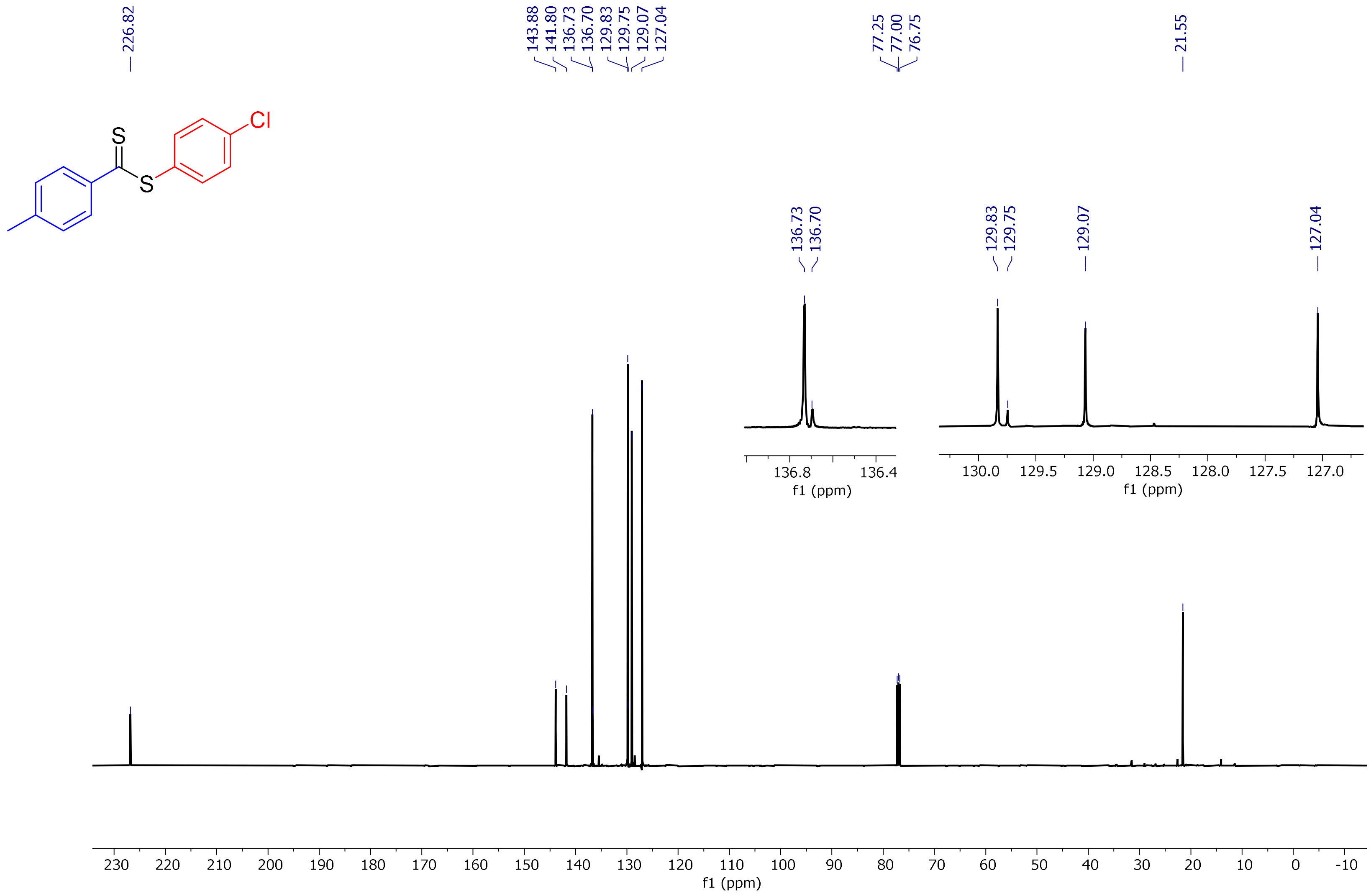

Figure S122. ^{1}H NMR (500 MHz, $CDCl_3$) spectrum for **34a**

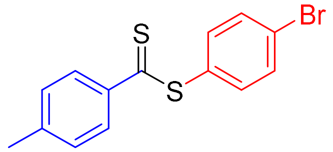

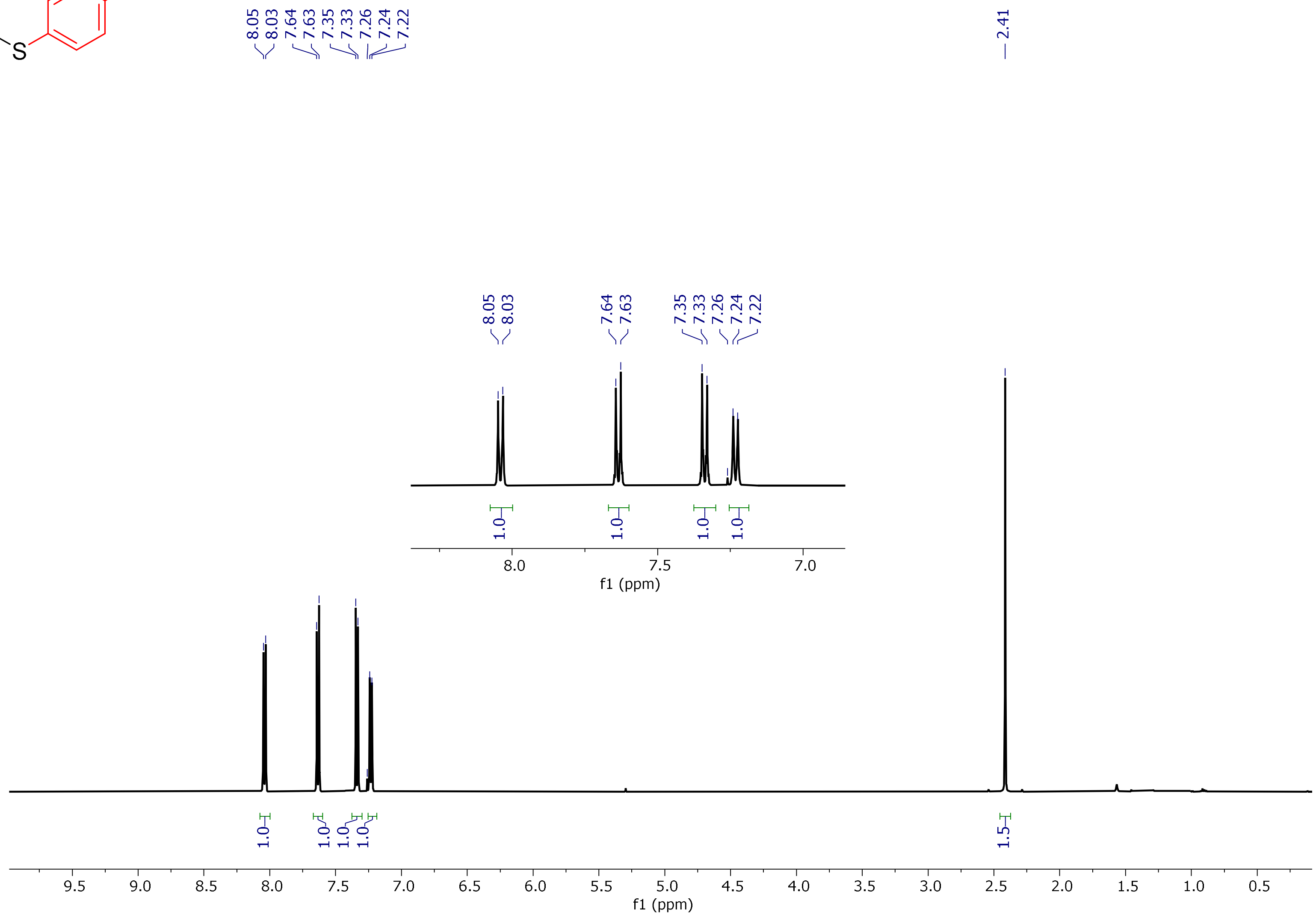

Figure S123. ^{13}C NMR (125 MHz, $CDCl_3$) spectrum for **34a**

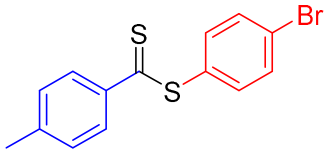

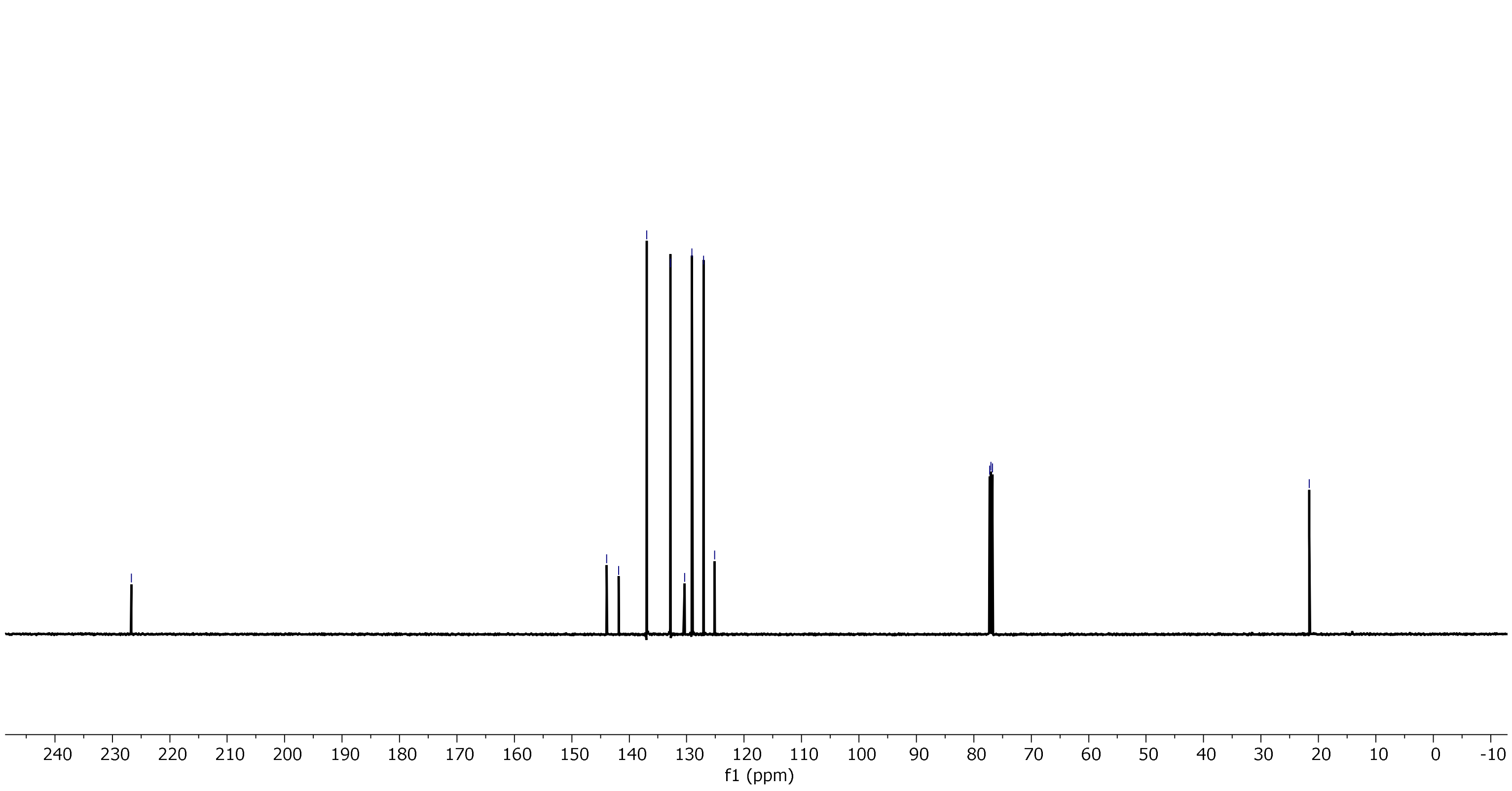

Figure S124. ^{1}H NMR (500 MHz, $CDCl_3$) spectrum for **35a**

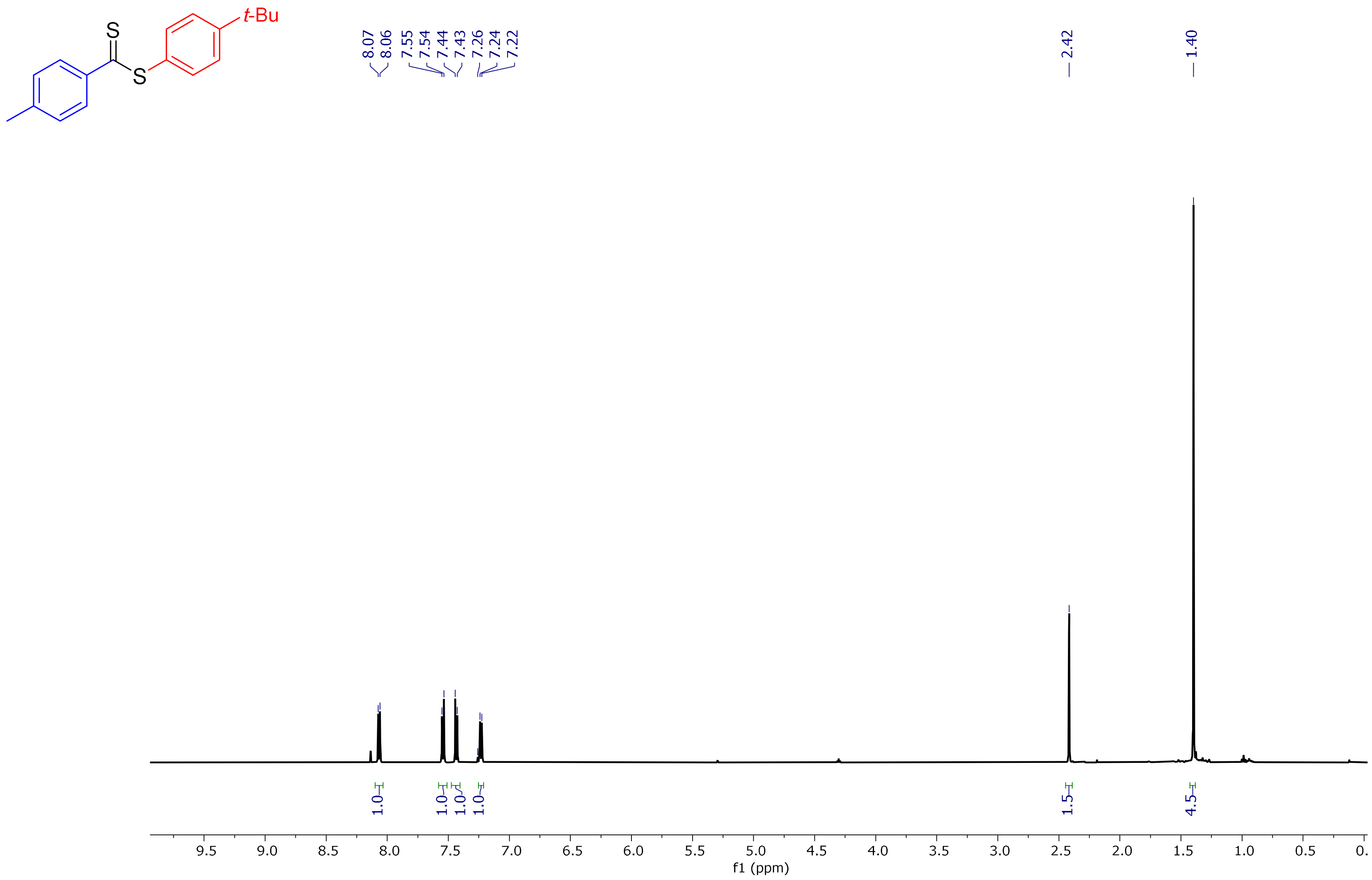

Figure S125. ^{13}C NMR (125 MHz, $CDCl_3$) spectrum for **35a**

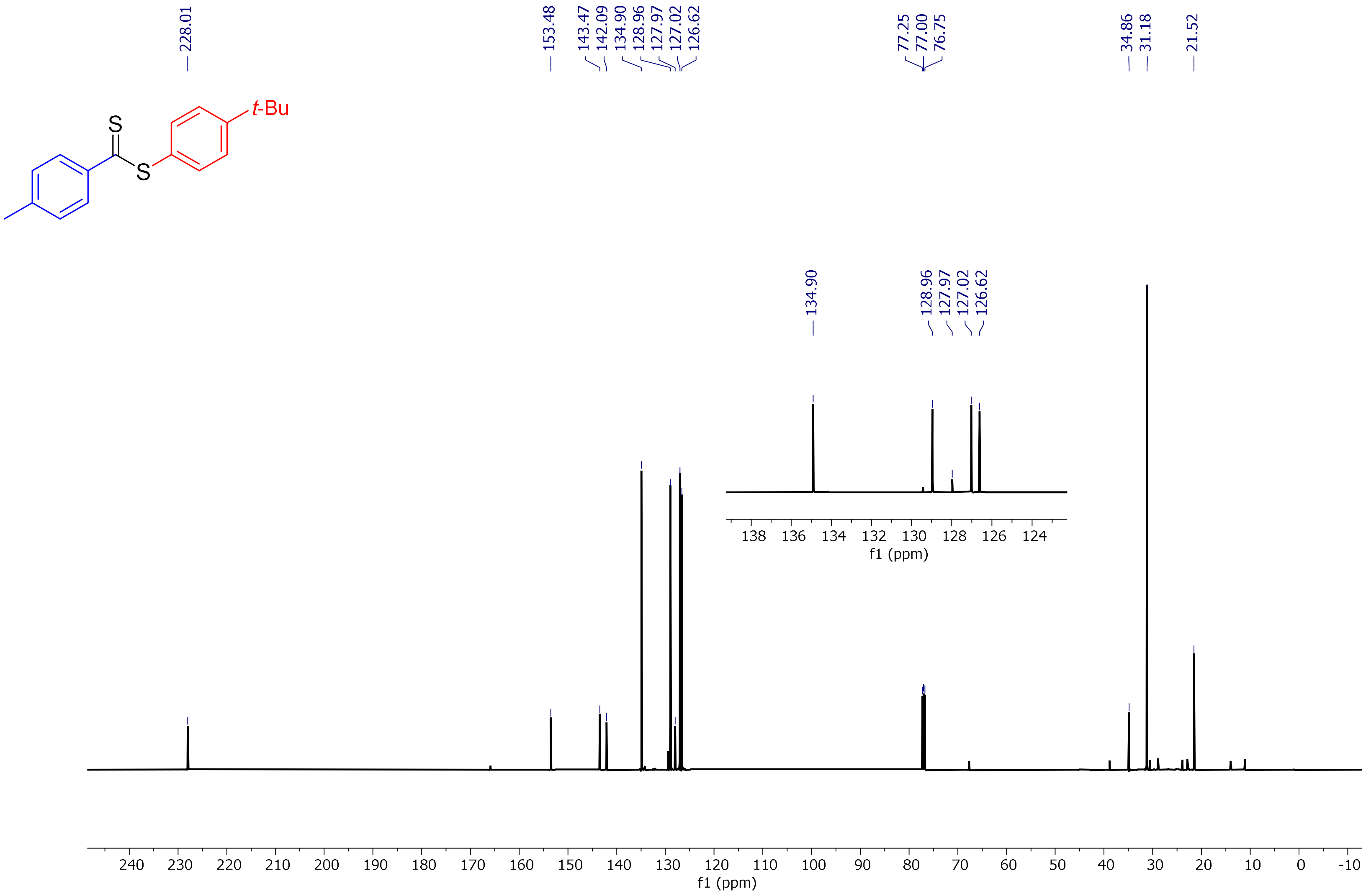

Figure S126. ^{1}H NMR (500 MHz, $CDCl_3$) spectrum for **36a**

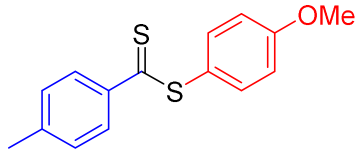

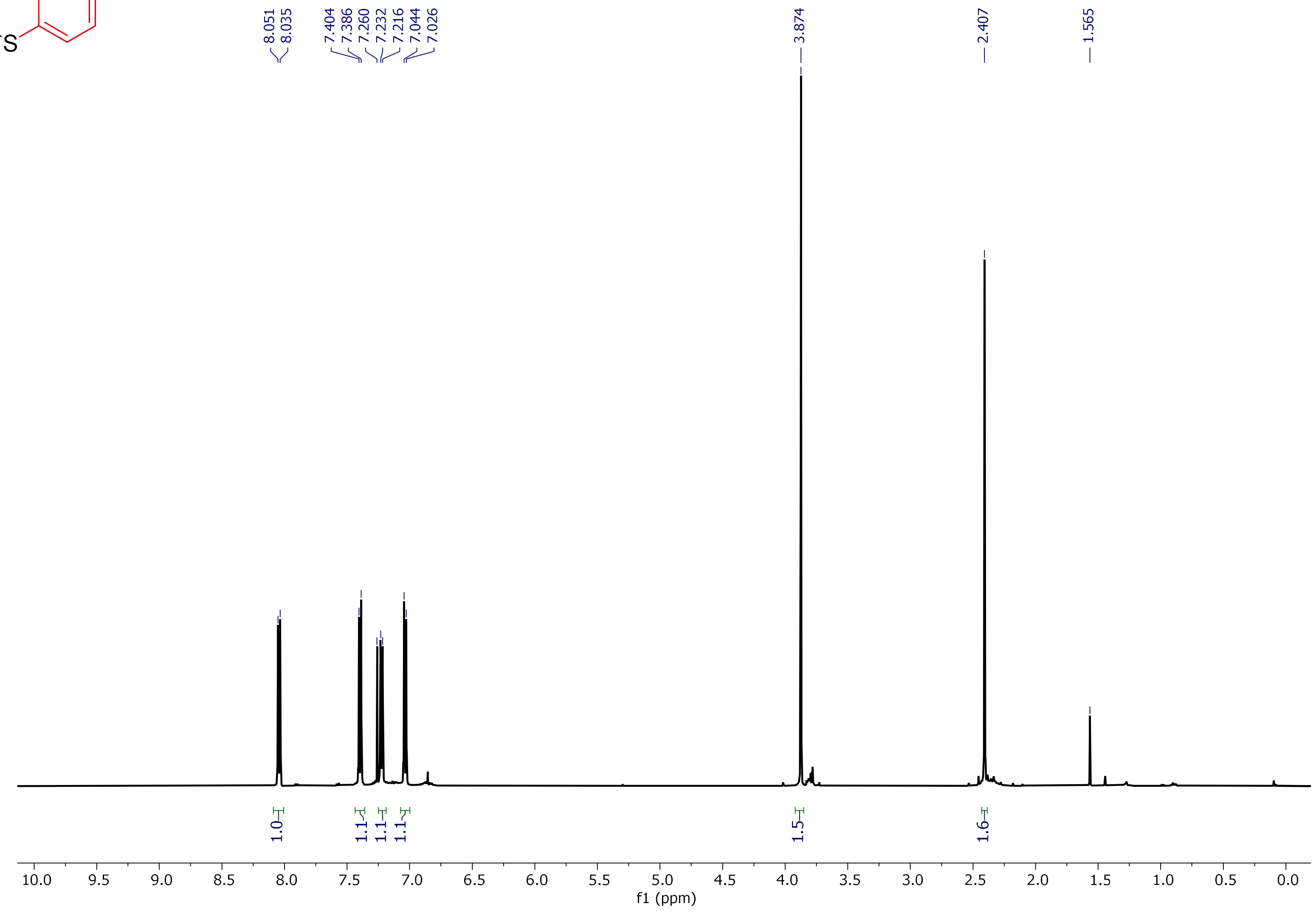

Figure S127. ^{13}C NMR (125 MHz, $CDCl_3$) spectrum for **36a**

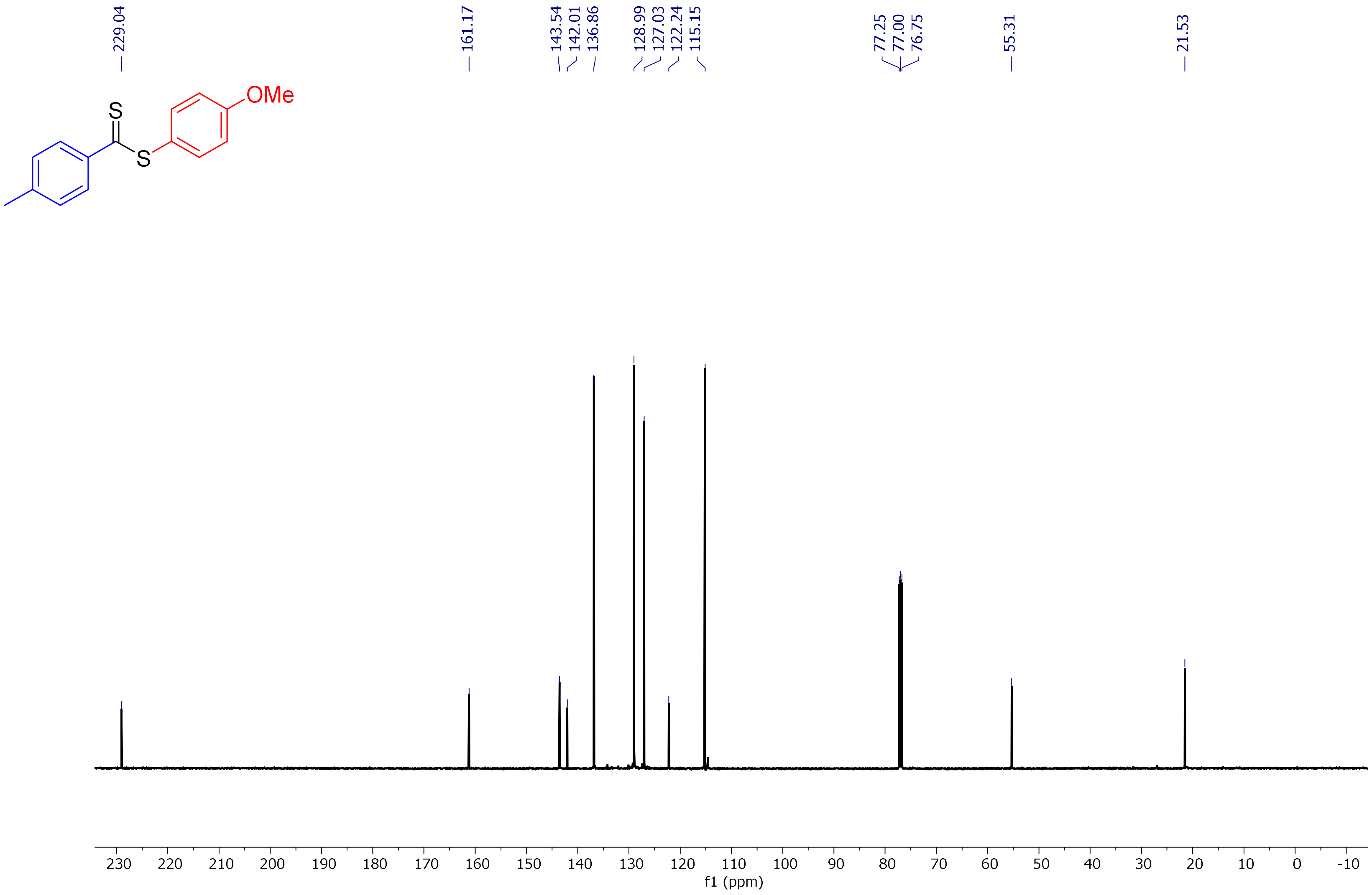

Figure S128. ^{1}H NMR (500 MHz, $CDCl_3$) spectrum for **37a**

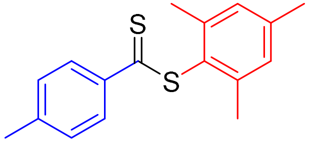

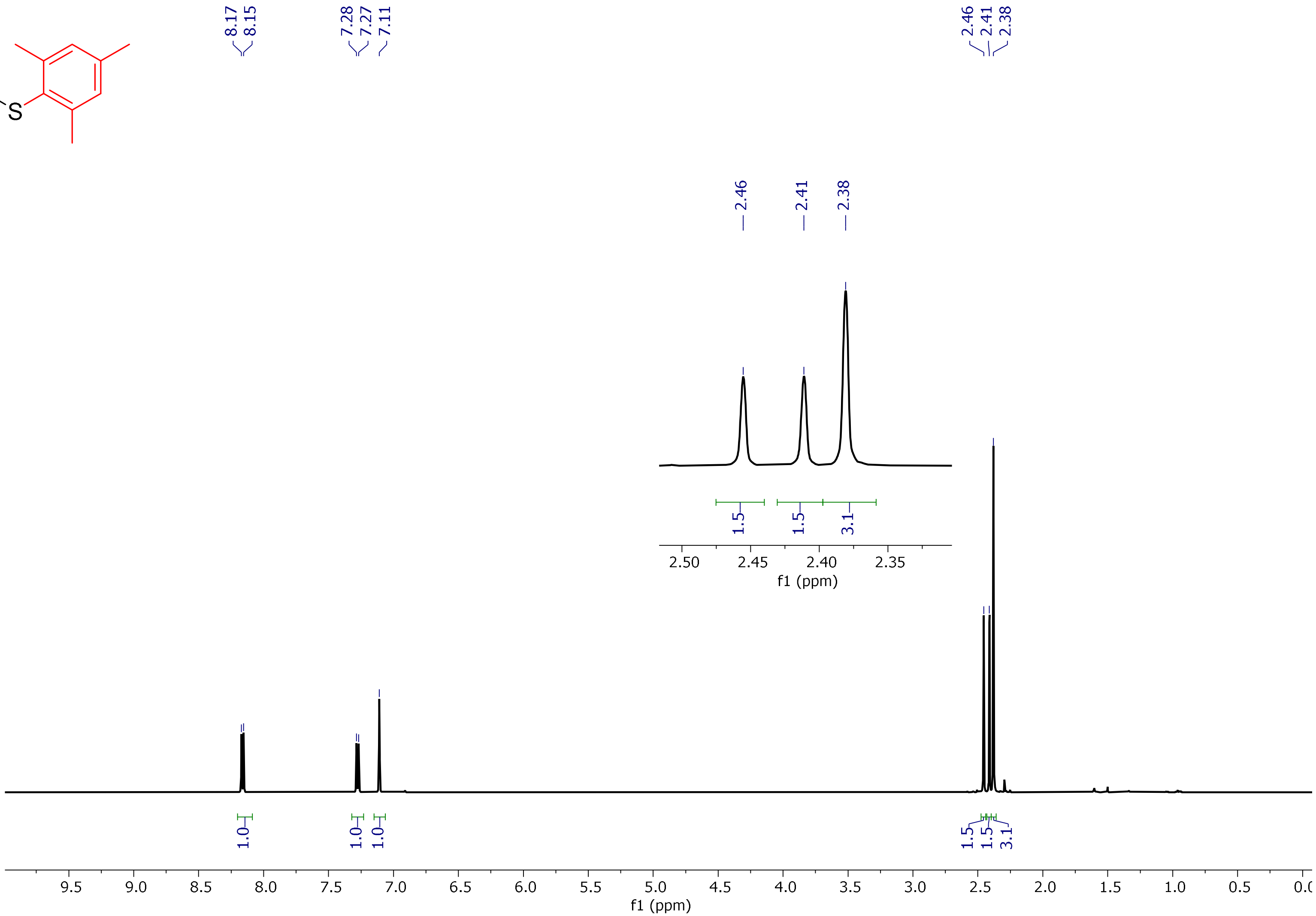

Figure S129. ^{13}C NMR (125 MHz, $CDCl_3$) spectrum for **37a**

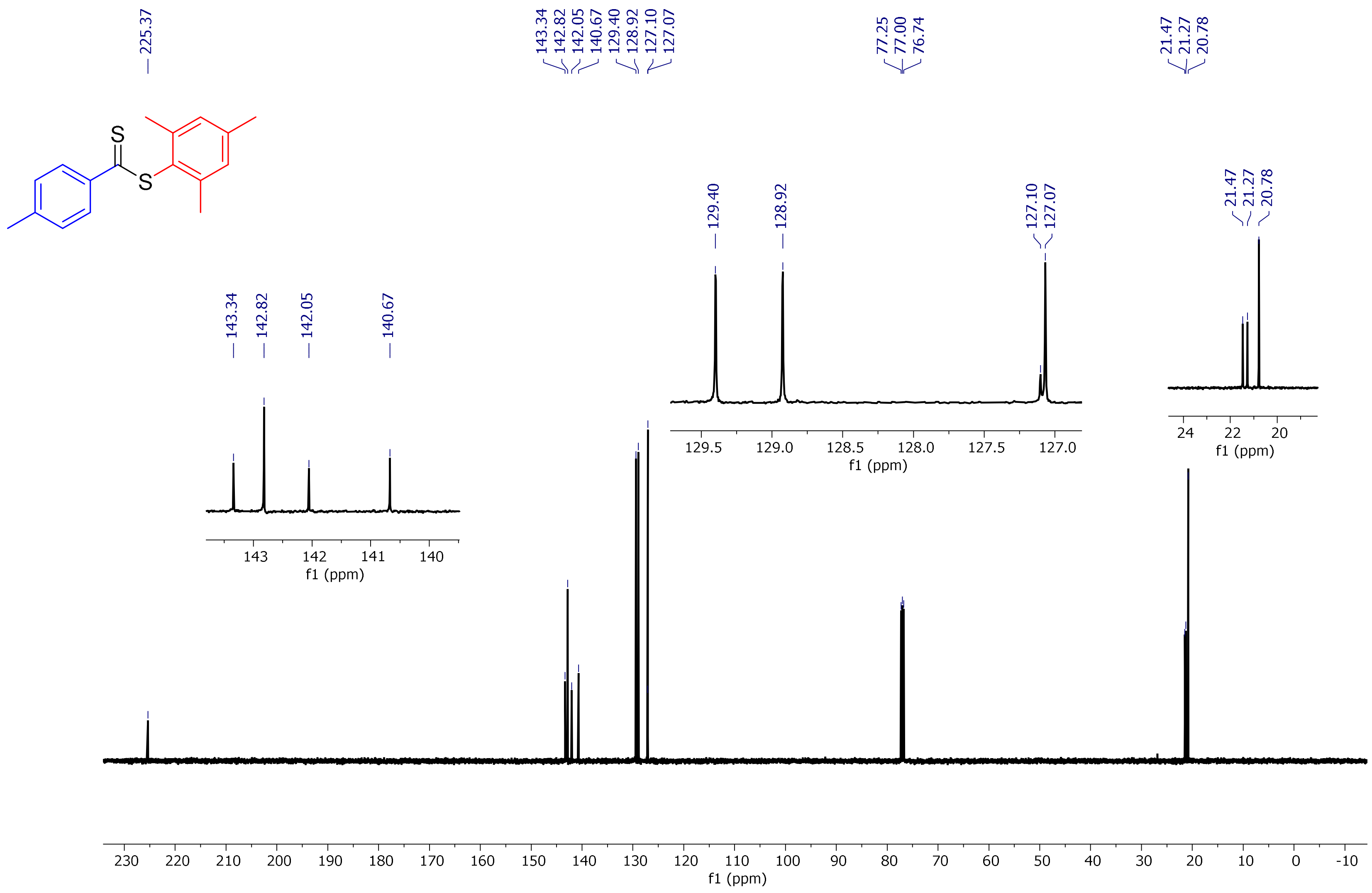

Figure S130. ^{1}H NMR (500 MHz, $CDCl_3$) spectrum for **37e**

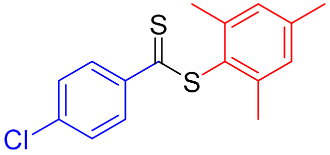

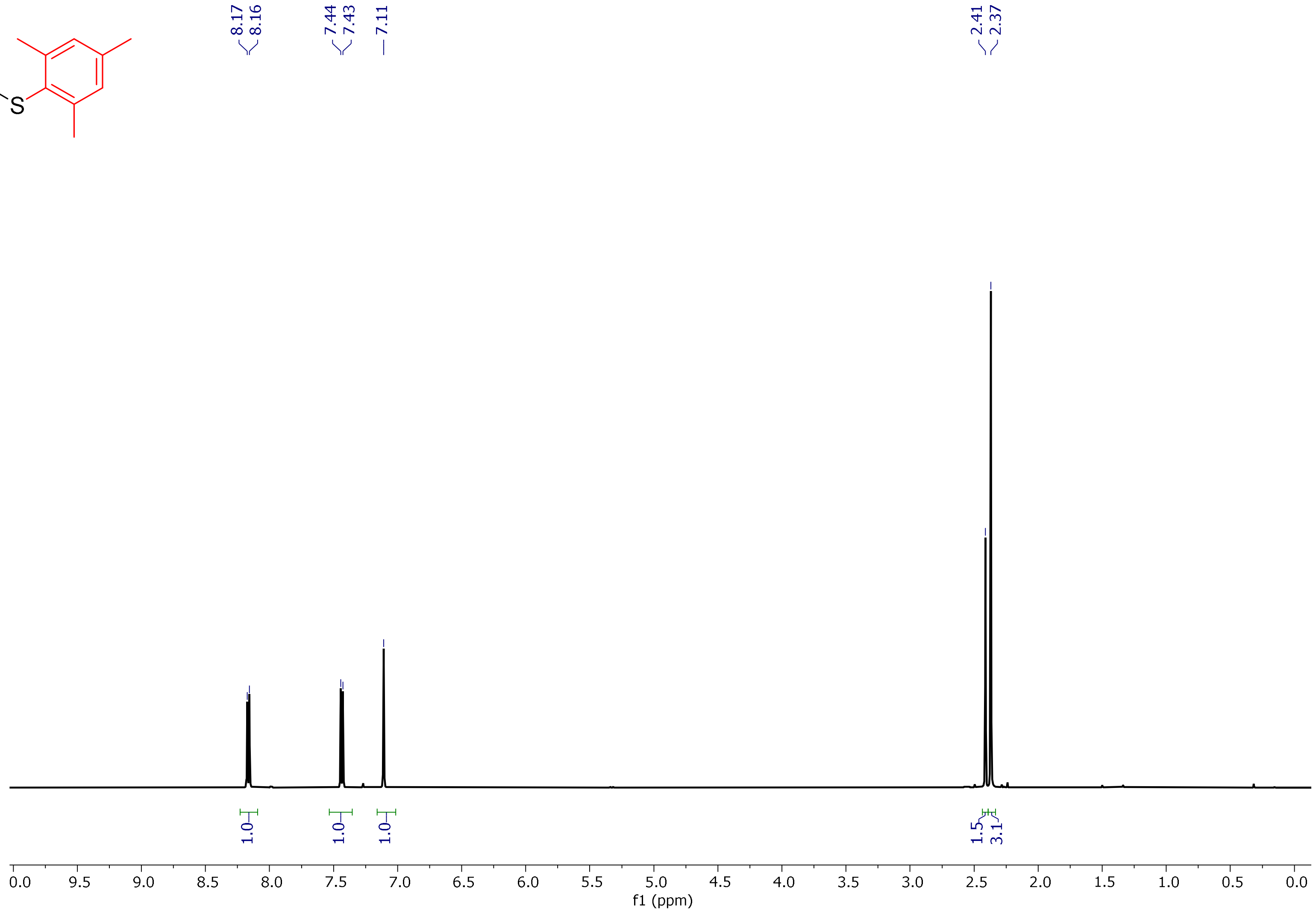

Figure S131. ^{13}C NMR (125 MHz, $CDCl_3$) spectrum for **37e**

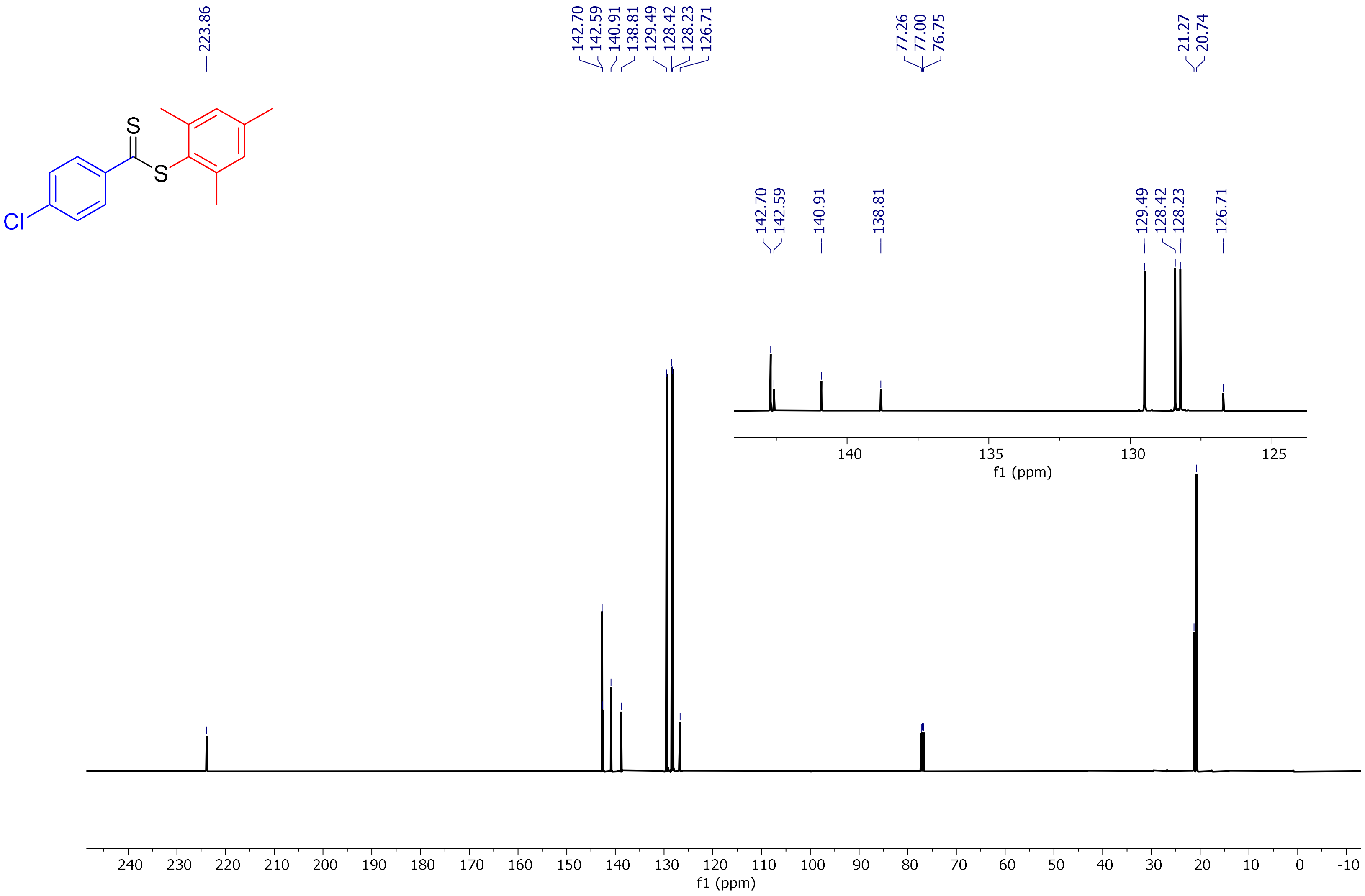

Figure S132. ^{1}H NMR (500 MHz, $CDCl_3$) spectrum for **37f**

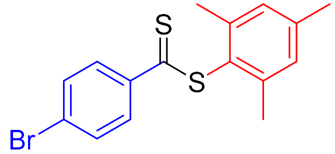

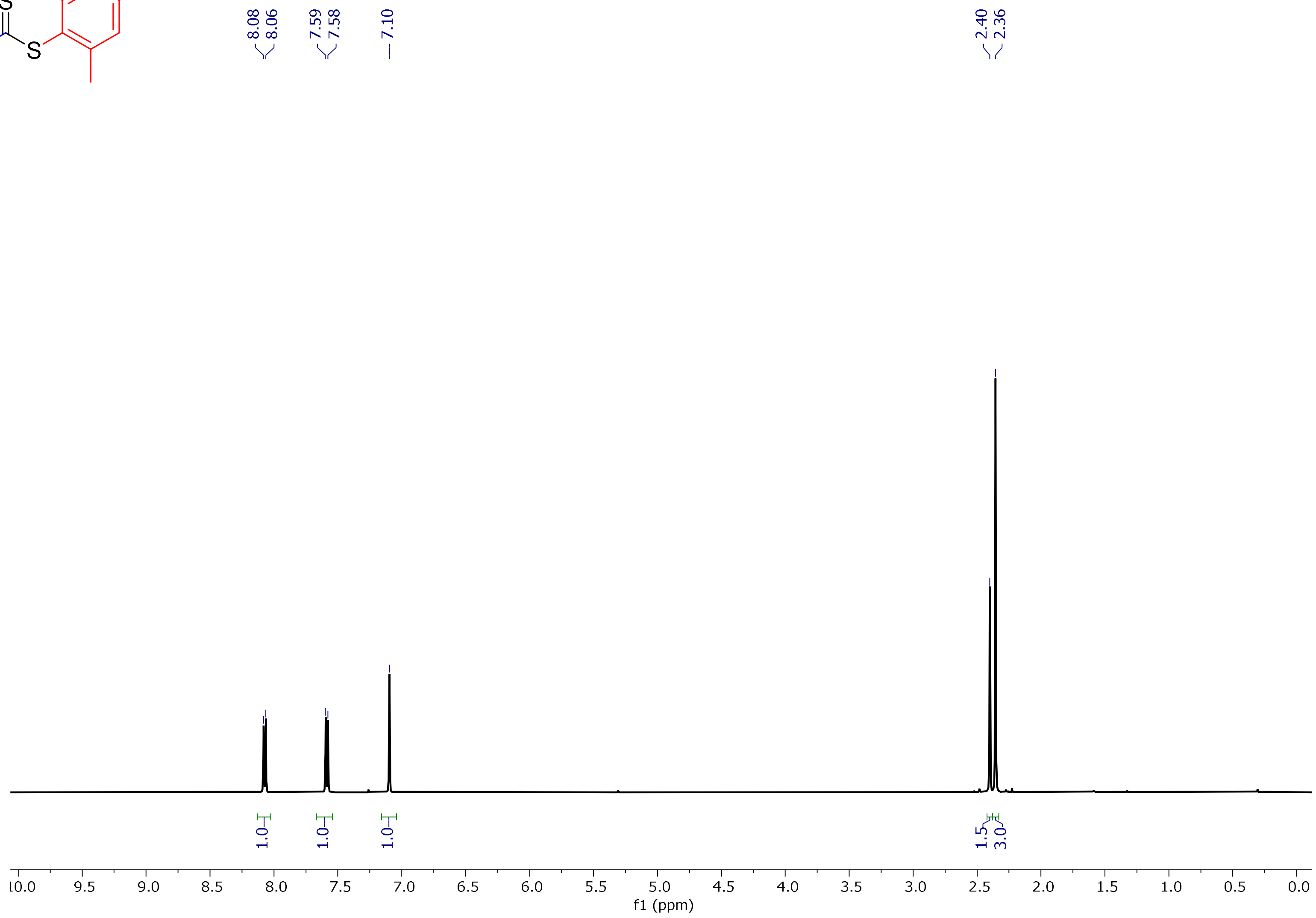

Figure S133. ^{13}C NMR (125 MHz, $CDCl_3$) spectrum for **37f**

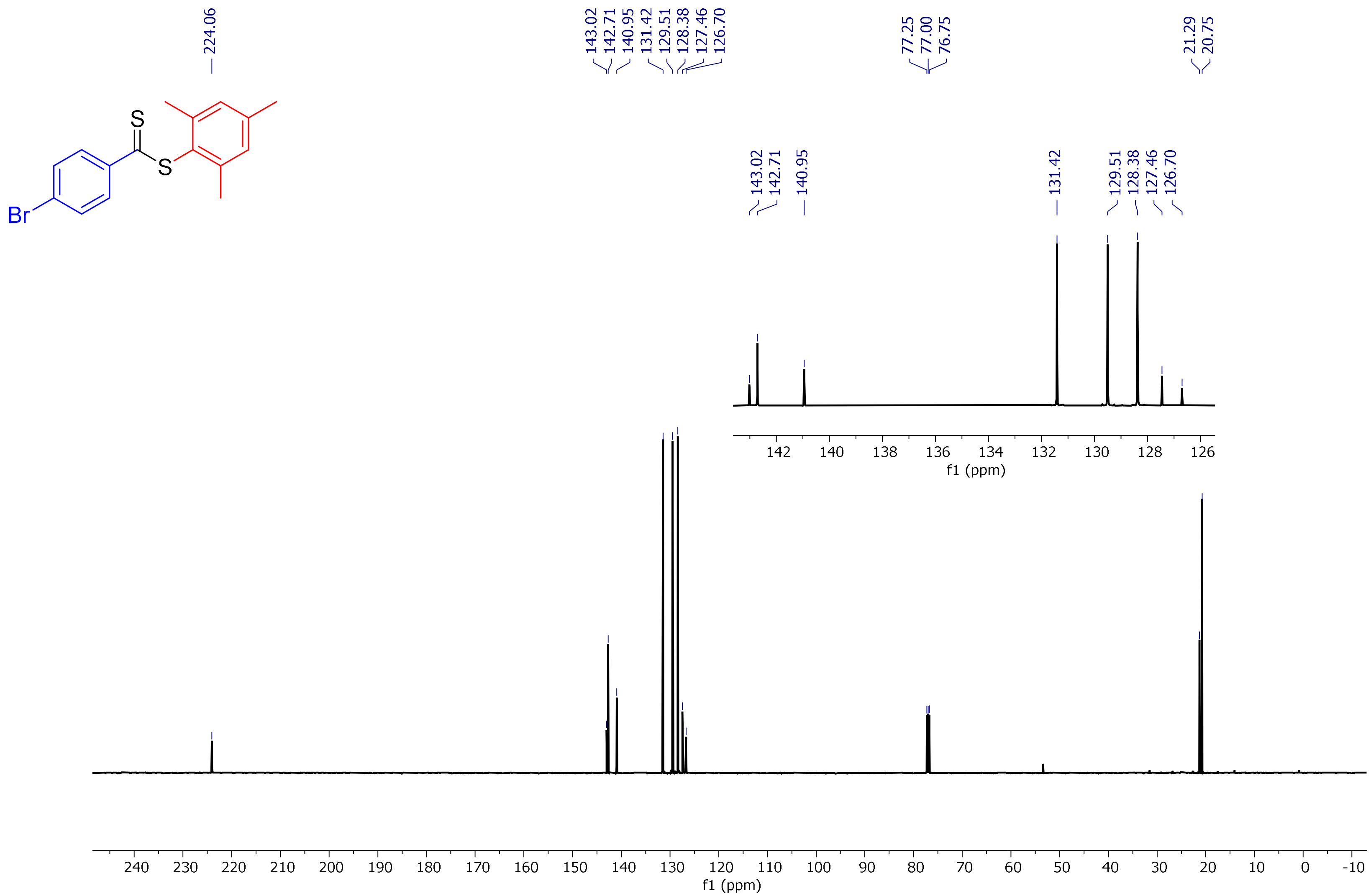

Figure S134. ^{1}H NMR (500 MHz, $CDCl_3$) spectrum for **2m'**

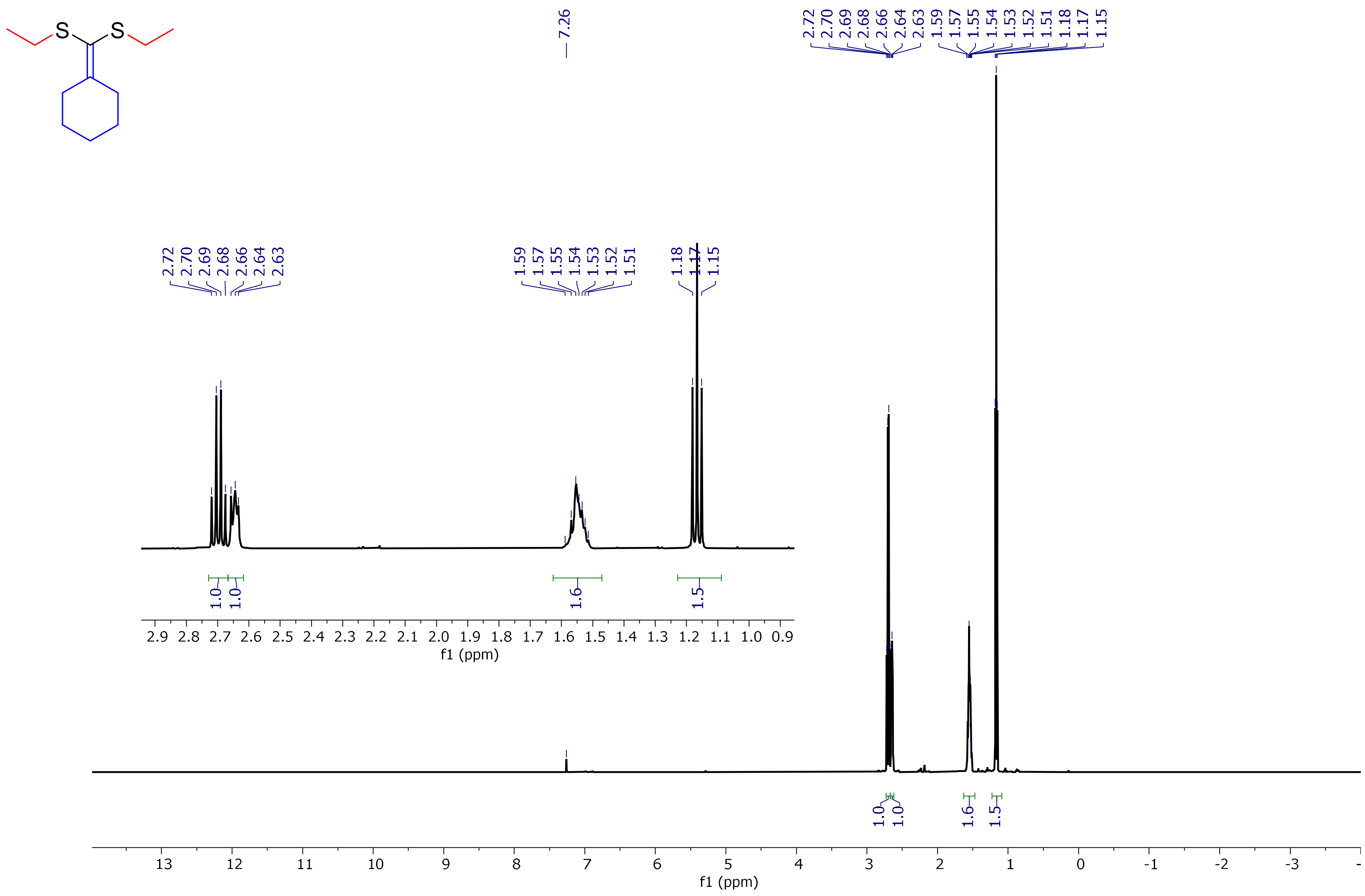

Figure S135. ^{13}C NMR (125 MHz, $CDCl_3$) spectrum for **2m'**

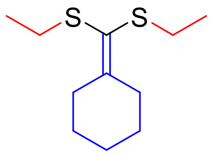

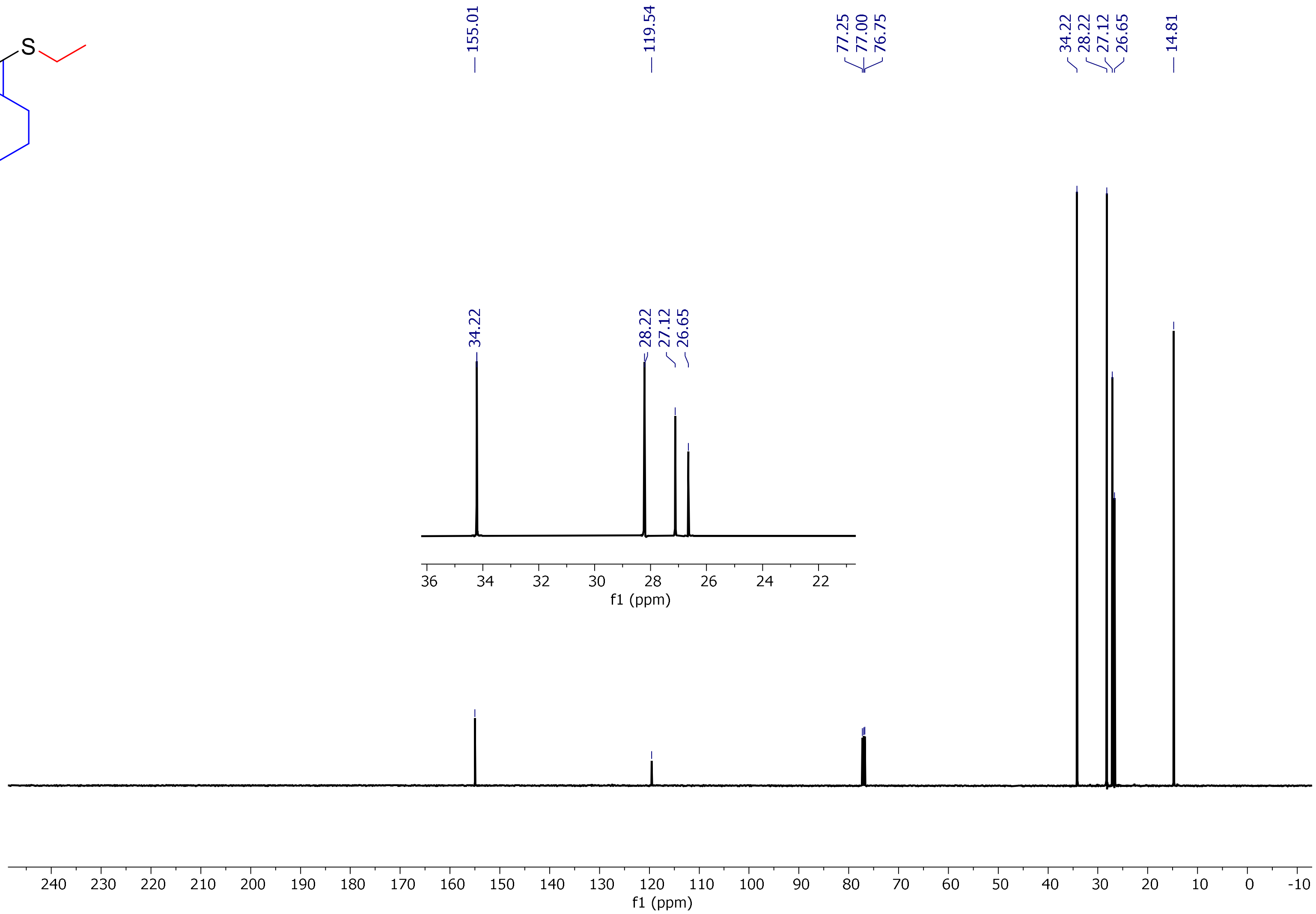

Figure S136. ^{1}H NMR (500 MHz, $CDCl_3$) spectrum for **2-bromoethyl 3-(4,5-diphenyloxazol-2-yl)propanoate**

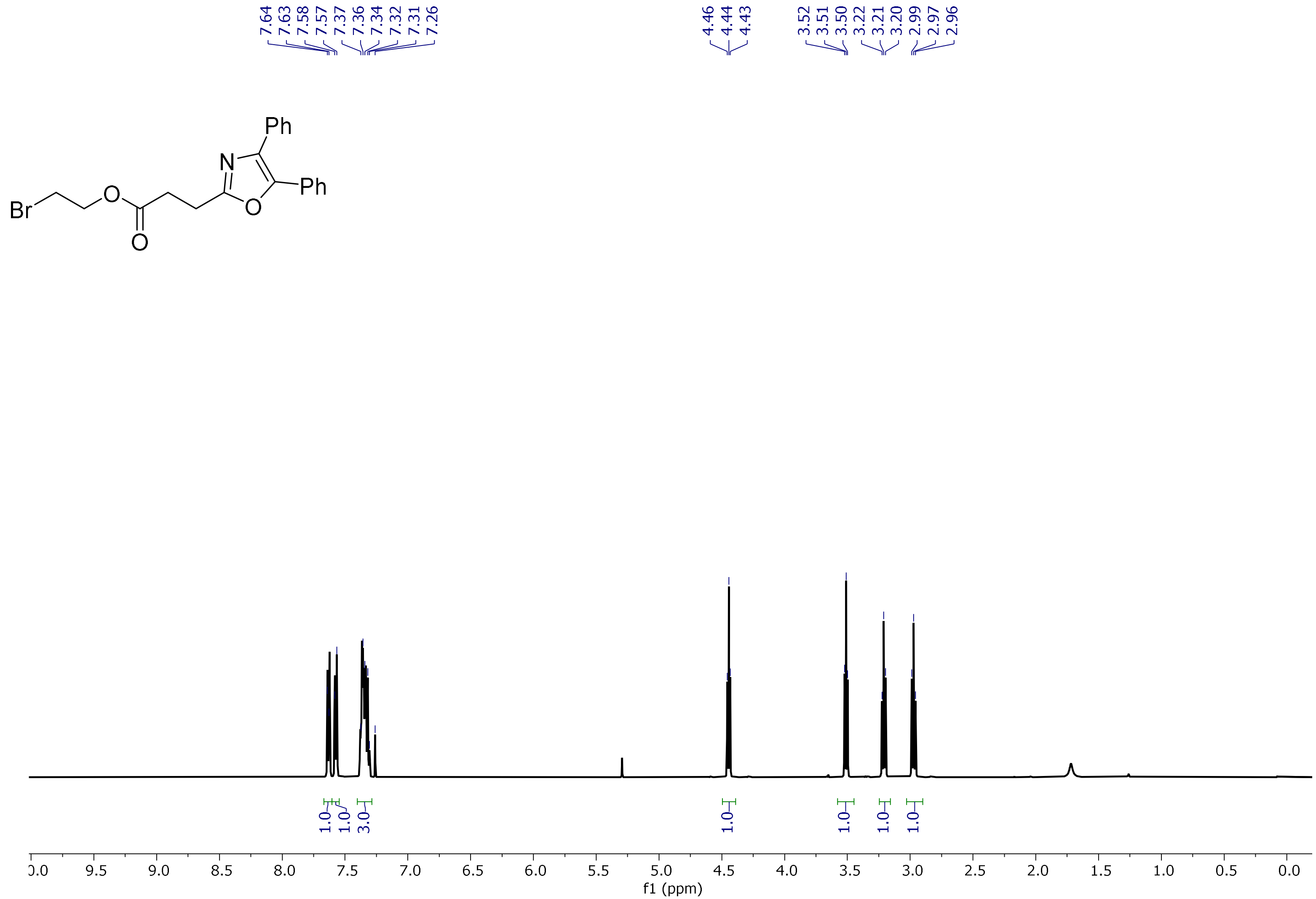

Figure S137. ^{13}C NMR (125 MHz, $CDCl_3$) spectrum for **2-bromoethyl 3-(4,5-diphenyloxazol-2-yl)propanoate**

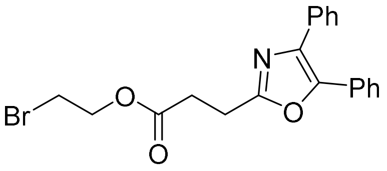

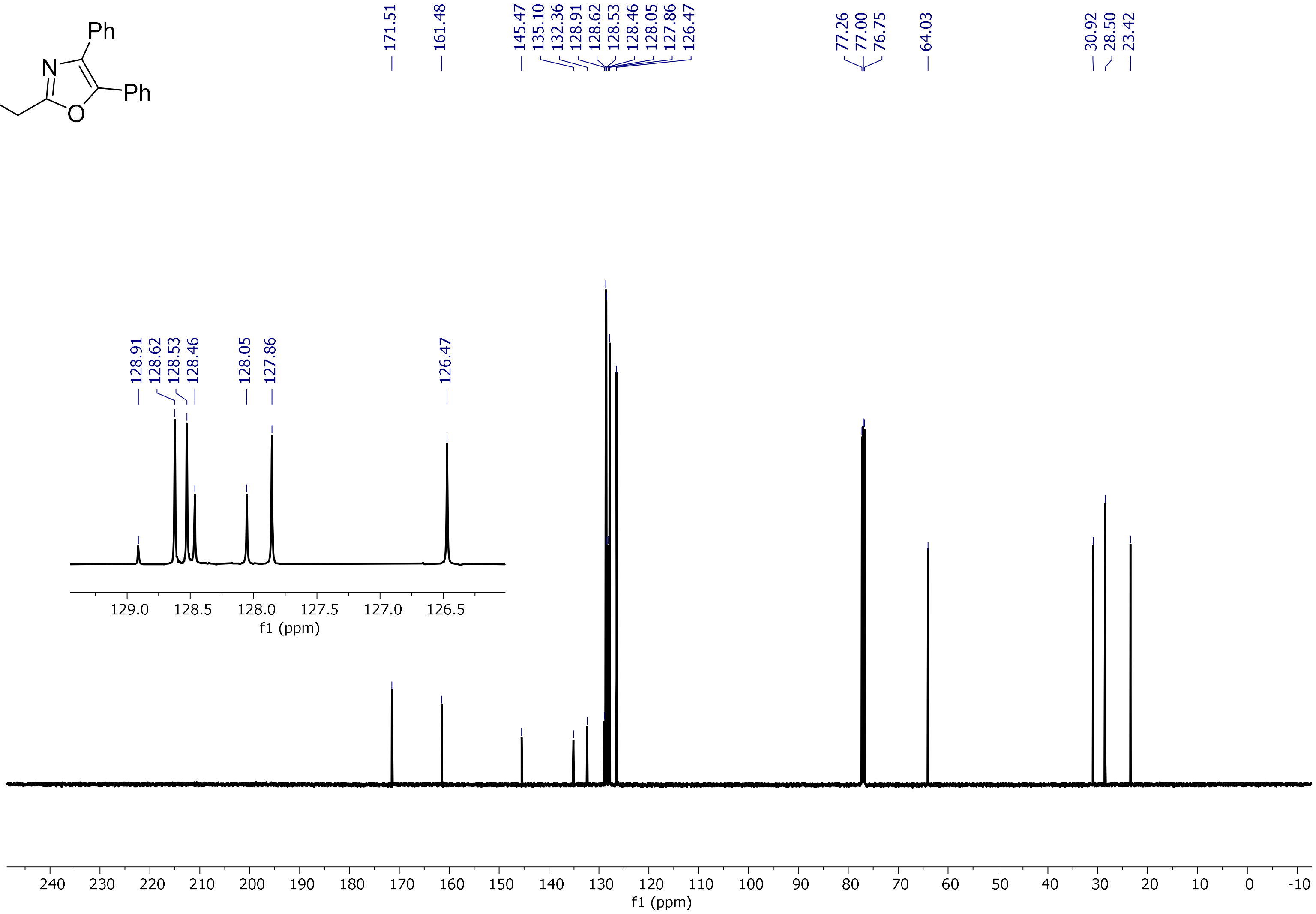

Figure S138. ^{1}H NMR (500 MHz, $CDCl_3$) spectrum for **2-bromoethyl 5-(2,5-dimethylphenoxy)-2,2-dimethylpentanoate**

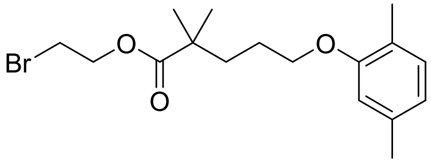

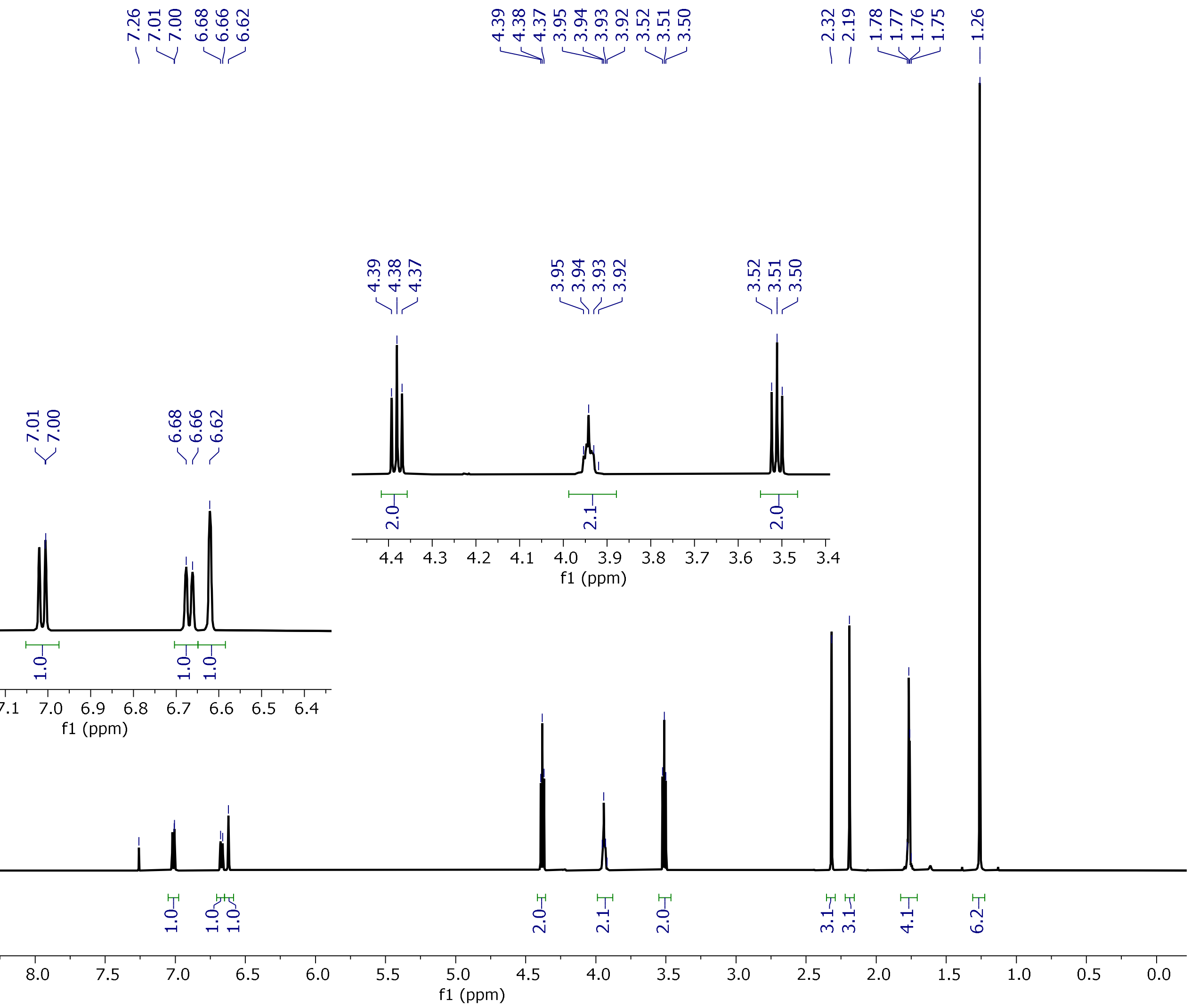

Figure S139. ^{13}C NMR (125 MHz, $CDCl_3$) spectrum for **2-bromoethyl 5-(2,5-dimethylphenoxy)-2,2-dimethylpentanoate**

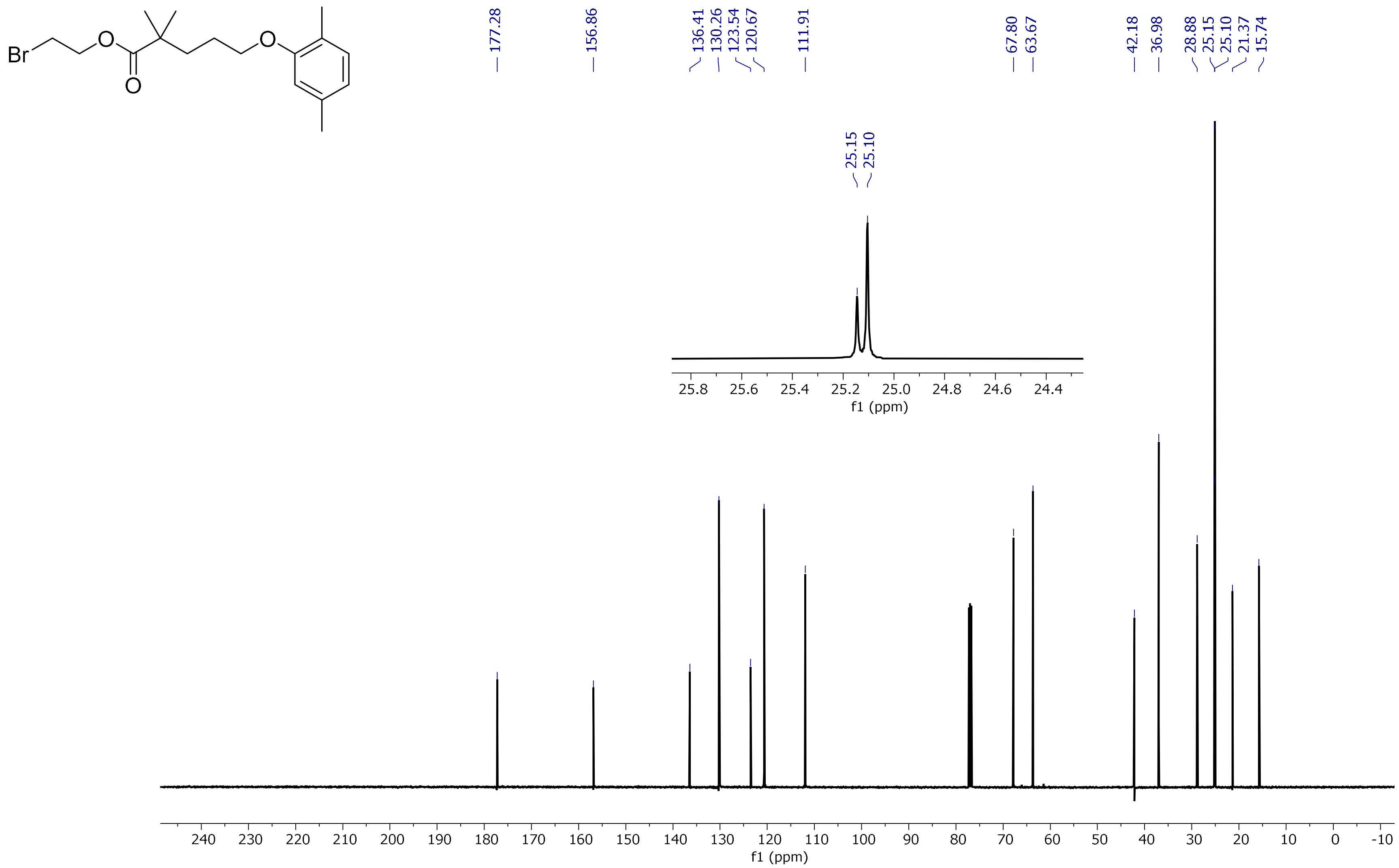

Supplement: Supplementary file 1 — ol5c00666_si_001.pdf [file ol5c00666_si_001.pdf]
